# Sulfoxide-directed metal-free cross-couplings in the expedient synthesis of benzothiophene-based components of materials†
†Electronic supplementary information (ESI) available: Full experimental details, NMR spectra, CCDC numbers for X-ray structures, and details of preliminary materials evaluation and device preparation. CCDC 1415330–1415334. For ESI and crystallographic data in CIF or other electronic format see DOI: 10.1039/c5sc03823e


**DOI:** 10.1039/c5sc03823e

**Published:** 2015-11-09

**Authors:** Andrew J. Eberhart, Harry Shrives, Yuntong Zhang, Amandine Carrër, Adam V. S. Parry, Daniel J. Tate, Michael L. Turner, David J. Procter

**Affiliations:** a School of Chemistry , University of Manchester , Oxford Rd , Manchester , M13 9PL , UK . Email: david.j.procter@manchester.ac.uk

## Abstract

A metal-free approach combining sulfoxide-directed metal-free C–H cross-couplings with tuneable electrophile-mediated heterocyclizations and carbocyclative dimerizations, allows expedient access to benzothiophene-based systems that are components of important materials or are proven organic materials in their own right. As benzothiophene-based materials are typically prepared using Pd-catalyzed cross-coupling processes, our approach allows potential issues of metal cost and supply, and metal-contamination of products, to be avoided.

## Abstract 2

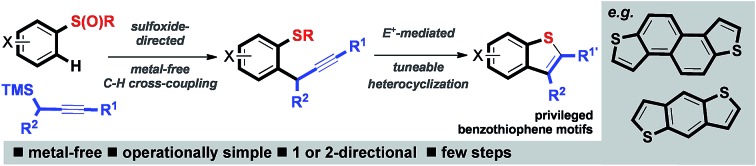
A metal-free approach combining sulfoxide-directed metal-free C–H cross-couplings with tuneable heterocyclizations and dimerizations allows expedient access to important organic materials.

## Introduction

The selective formation of carbon–carbon bonds to aromatic rings is a critical goal in science.^1^ Although the use of first row metals is an important and growing field (*e.g.* Fe, Co, Ni, Cu), modern cross-coupling technology still typically involves the use of platinum group metals (*e.g.* Pd, Ru, Rh, Pt),^2^ and the development of cross-coupling reactions that do not involve the use of an expensive and/or supply-risk metal is highly desirable.^3^ Such processes have additional benefits as trace metal contamination in products arising from metal-catalyzed processes is a concern in industry, particularly the pharmaceutical and organic electronic sectors.^4^ In the latter field, even ‘undetectable’ levels of palladium contaminant can have a detrimental effect on the electrical properties of materials and thin film device performance.4*d*

**Scheme 1:**
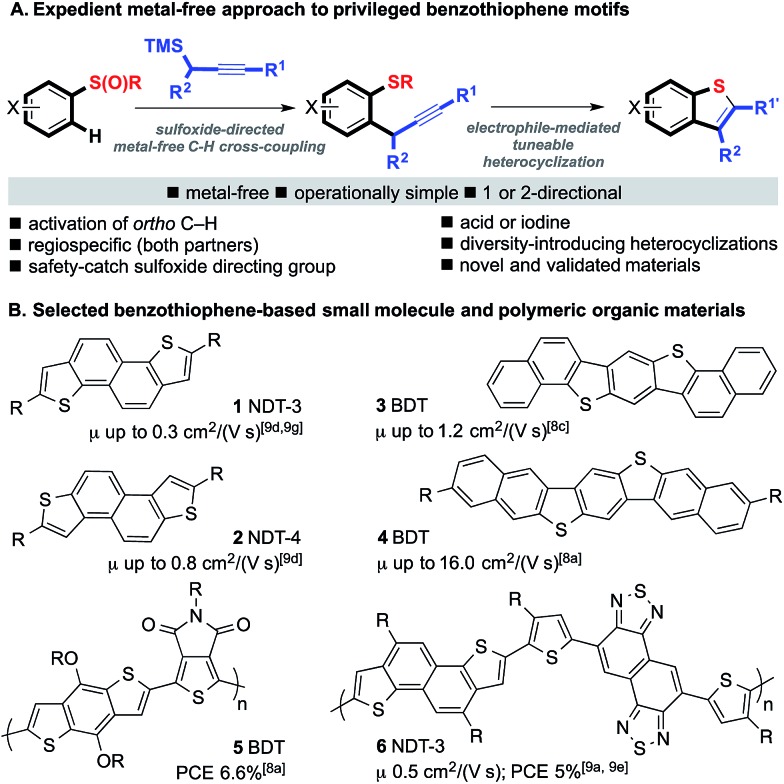
(A) Short metal-free approach to privileged benzothiophenes. (B) Benzothiophene-based organic materials. NDT = napthodithiophene; BDT = benzodithiophene; μ = electron mobility; PCE = Power Conversion Efficiency.

Here we describe an expedient metal-free approach to important benzothiophene-based architectures, including motifs that are crucial components in valuable organic small molecule and polymeric materials (Scheme 1A).^5^ Organic materials are increasingly prepared using C–H activation methods mediated by platinum group metals^6^ in addition to cross-couplings using classical Pd-catalyzed methods.^7^ Benzodithiophenes (BDTs)^8^ and napthodithiophenes (NDTs),^9^ in particular, have been exploited in high-performance small-molecule/oligomeric semi-conducting materials (*e.g.***1–4**), polymeric semiconductors,^8^,^9^ and in co-polymers for solar cells^8^,^9^ (*e.g.***5** and **6**) (Scheme 1B).

The crucial benzothiophene-based motifs are typically prepared using Pd-catalyzed cross-coupling processes in one or more key steps. Our approach utilizes sulfoxide-directed metal-free C–H cross-coupling processes,^10^ allowing established Pd-catalyzed couplings and associated metal-contamination to be avoided, and new diversity-introducing hetero- and carbocyclizations mediated by electrophiles. The novel approach has been used in the metal-free synthesis of a range of benzothiophenes including components of materials, and both validated organic materials and previously unknown organic materials for evaluation.

## Results and discussion

### A two-directional, metal-free route to NDT materials

**Scheme 2:**
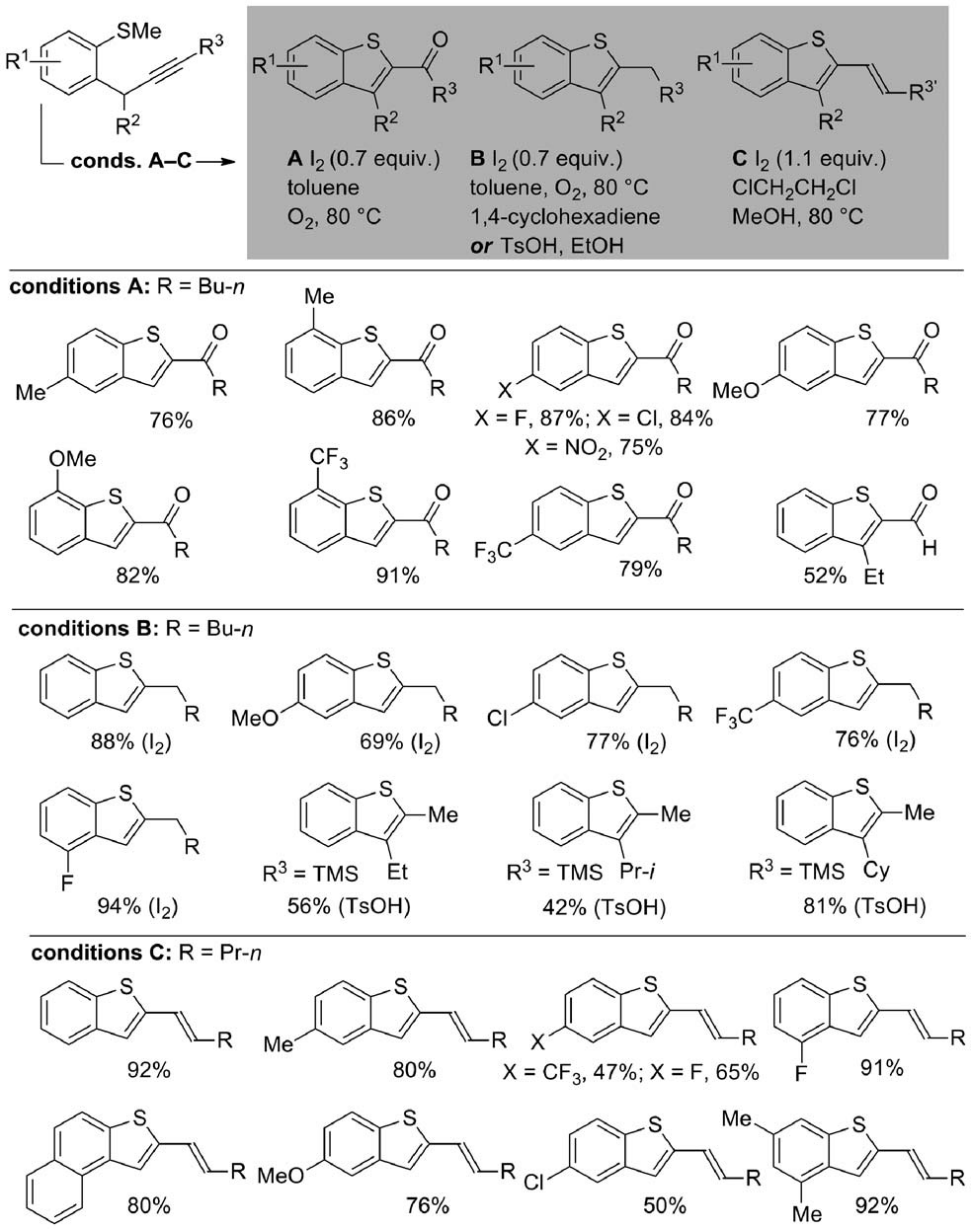
Tuneable electrophile-mediated, heterocyclization to give decorated benzothiophenes (isolated yields after purification).

We began by exploring the conversion of the products of our sulfoxide-directed, metal-free, propargylative C–H cross-coupling^10^ to benzothiophenes. Pleasingly, straight-forward variation of iodine or acid-mediated conditions (A–C) allowed access to a wide range of decorated benzothiophene motifs bearing different levels of oxidation at the carbon adjacent to the benzothiophene ring (Scheme 2).^11^ In addition, a wide range of substituents on the aromatic ring are tolerated thus allowing the electronic properties of target materials to be tuned (*vide infra*).

**Scheme 3:**
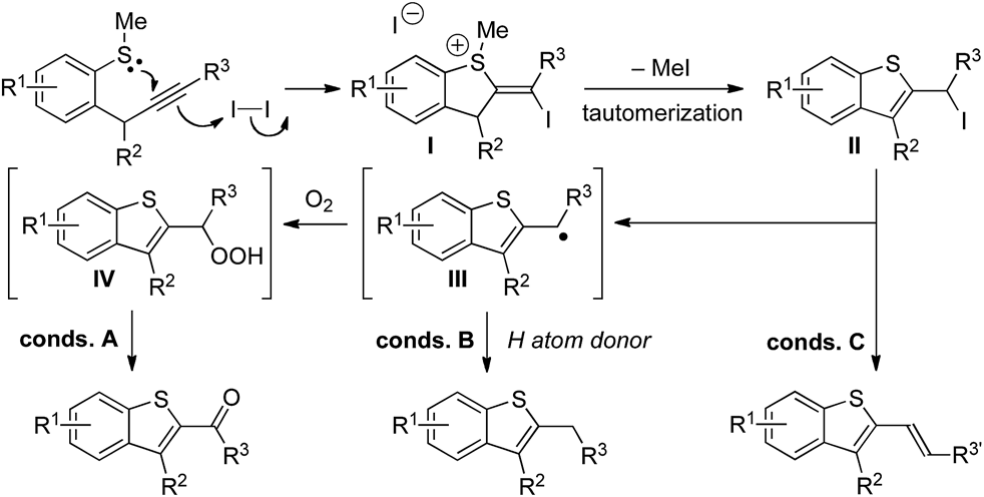
Proposed mechanism for the tuneable iodine-mediated, heterocyclization to give decorated benzothiophenes.

A proposed mechanism for the formation of benzothiophenes under iodine-mediated conditions A–C is set out in Scheme 3. Upon treatment with iodine, cyclization gives sulfonium salts **I** that undergo demethylation and tautomerization to form common iodide intermediates **II**. Upon heating, homolysis gives radicals **III** that either undergo quenching with O_2_ (conditions A) to give ketone products, possibly *via* alkylhydroperoxide intermediates **IV**, or with 1,4-cyclohexadiene (conditions B) to give alkyl substituted benzothiophene products. Under conditions C, elimination of iodides **II** gives alkenyl benzothiophene products. Acid-mediated cyclization (alternative conditions B) gives alkyl substituted benzothiophene products directly after demethylation of sulfonium salt intermediates.

**Scheme 4:**
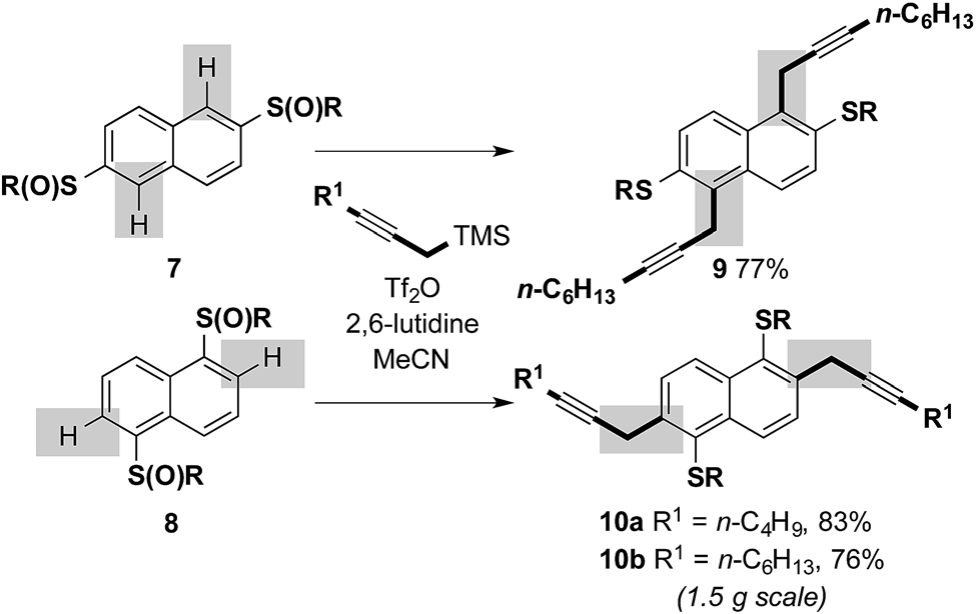
Two-directional, sulfoxide-directed, metal-free cross-couplings involving substrates derived from dihydroxynaphthalenes; R = *n*-C_6_H_13_ (isolated yields after purification).

We next investigated the feasibility of combining sulfoxide-directed cross-coupling with electrophile-mediated heterocyclizations in metal-free approaches to benzothiophene-based components of organic materials. We first examined a metal-free, two-directional synthesis of naphthodithiophene (NDT) motifs, typically prepared using Pd-catalyzed Sonogashira couplings,9d,g and found in high performance small molecule and polymeric organic materials.^9^ Bis-sulfoxide starter units **7** and **8**, readily-prepared from 2,6-dihydroxynapthalene (82%, 2 steps) and 1,5-dihydroxynapthalene (66%, 2 steps) (*n*-C_6_H_13_SH, toluene, Dean–Stark; *m*CPBA, CH_2_Cl_2_),^12^ underwent two-directional metal-free C–H propargylation, under our previously reported conditions,^10^ to deliver adducts **9** and **10** in high yield (Scheme 4). In the cross-coupling to give **9**, complete regiocontrol is observed. The process proved scalable and the cross-coupling of **8** gave **10b** in 76% on a 1.5 g scale.

**Scheme 5:**
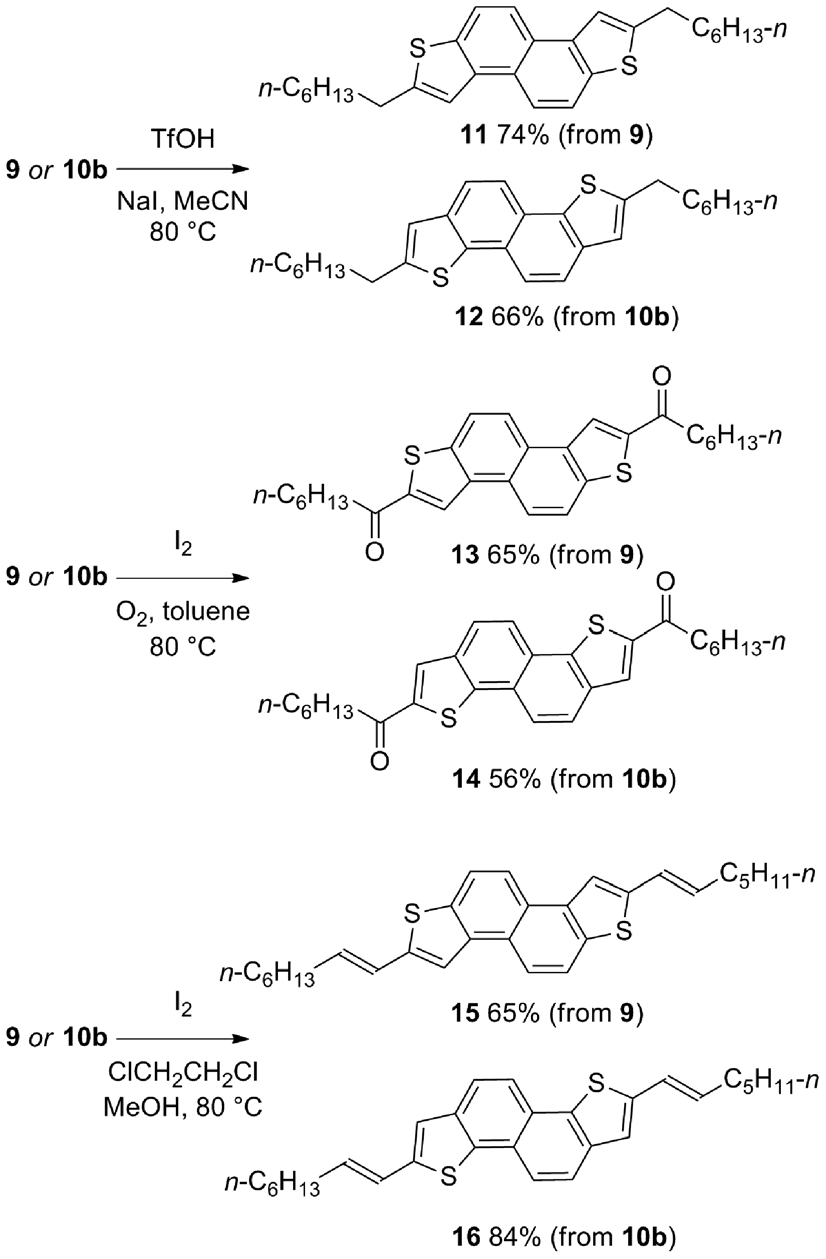
‘Tuneable’ 2-directional heterocyclizations for the metal-free synthesis of NDT materials (isolated yields after purification).

**Fig. 1:**
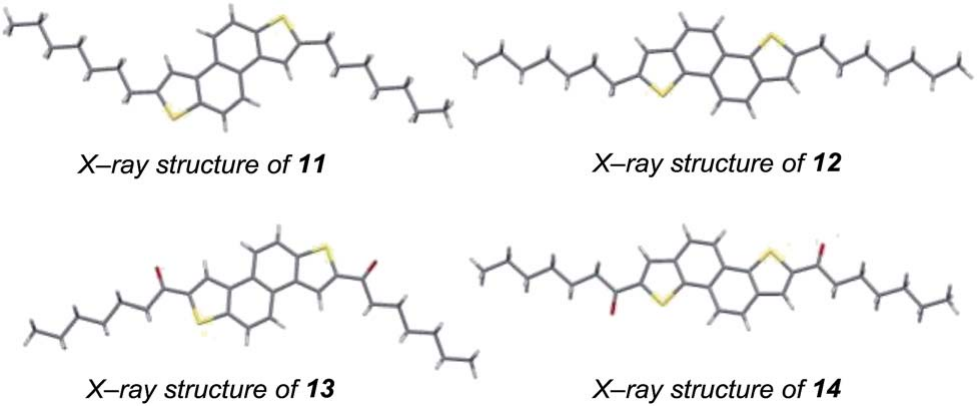
X-ray crystallographic analysis of materials **11–14**.

Further diversity can be introduced by tuning the conditions of the subsequent two-directional heterocyclization. For example, while acid-mediated cyclization of **9** and **10b** (variant of conditions B; the use of NaI facilitates demethylation of sulfur in an intermediate sulfonium salt; Schemes 2 and 3) delivers alkyl-substituted materials **11** and **12**, respectively, iodine-mediated cyclization delivers acyl-substituted materials **13** and **14** (all 2 steps from sulfoxide). Furthermore, iodine-mediated cyclization of **9** and **10b** under eliminative conditions gave alkenylated products **15** and **16** (Scheme 5). NDT products **11–16** are potential small molecule organic materials in their own right (*vide infra*)9d,g and **11–14** were characterized by X-ray crystallographic analysis (Fig. 1).^13^ Thus, sulfoxide-directed cross-couplings allow palladium-mediated couplings to be avoided in the four-step synthesis of validated (**11** and **12**) and unexplored (**13–16**) types of organic material.

### A metal-free route to BDT materials

**Scheme 6:**
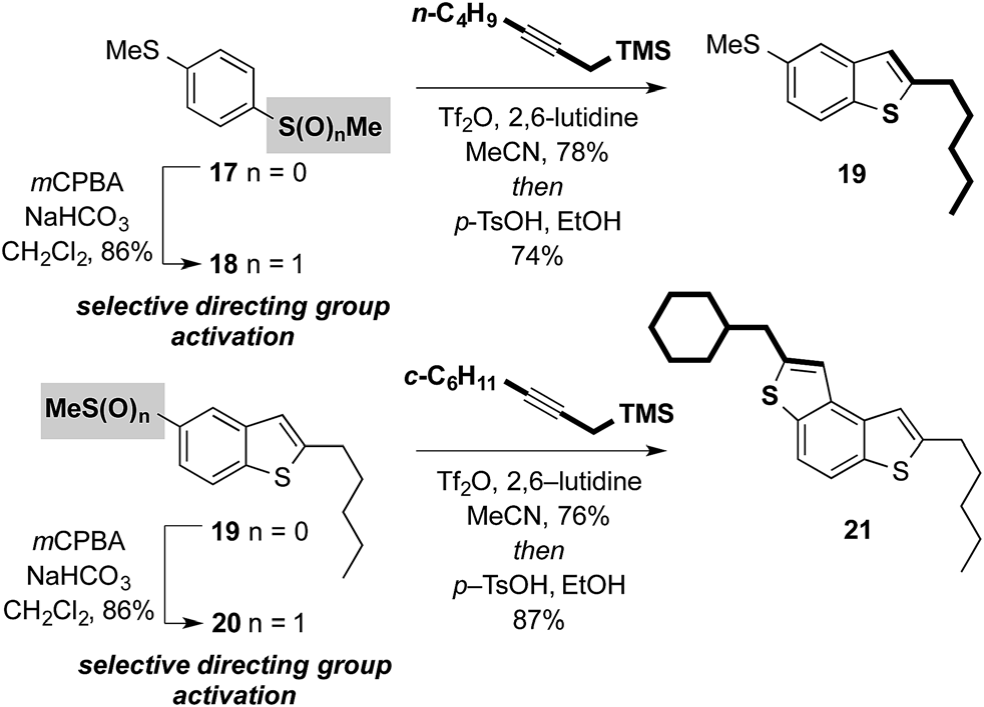
‘Safety-catch’ sulfoxide directing groups in metal-free cross-couplings: potential for the selective synthesis of unsymmetrical benzothiophene-based components.

Attractively, the sulfoxide moiety in our approach can be viewed as a ‘safety-catch’ directing group: only upon oxidation to the sulfoxide is the substrate receptive to cross-coupling. Furthermore, the directing group is reduced during the coupling, the directing effect is ‘switched-off’, and over-alkylation is impossible.10*b* Selective coupling in bis-sulfide substrates bearing latent directing groups is therefore possible provided selective oxidation can be achieved. For example, symmetrical bis-sulfide starter unit **17** and intermediate **19** can be selectively activated and thus undergo selective metal-free cross-coupling. This allows the controlled metal-free construction of unsymmetrical targets such as BDT **21** using iterative sulfoxide-directed cross-coupling (Scheme 6). The ability to prepare unsymmetrical small-molecule materials is crucial for several applications including the immobilization of materials on surfaces in the exploitation of self-assembled monolayers (SAM) in organic electronic devices.^14^

**Scheme 7:**
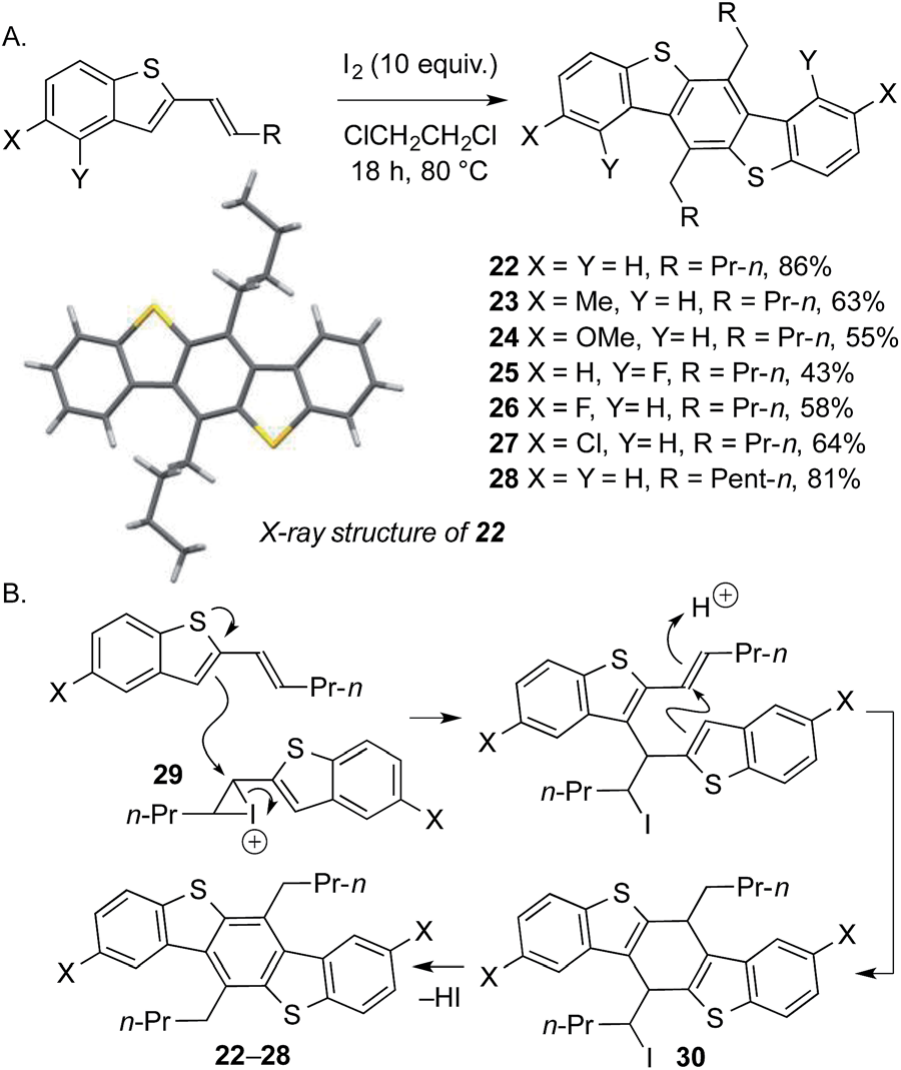
(A) Carbocyclative dimerization in a metal-free approach to novel dialkyl-substituted benzodithiophenes (BDTs). (B) Proposed mechanism for the dimerization.

We next addressed the challenge of developing a short, metal-free synthesis of extended BDT motifs. Such motifs are typically prepared by Pd-catalyzed Sonogashira, Suzuki, or Negishi couplings.^8^ Tuning the heterocyclization of the products from metal-free cross-coupling delivers benzothiophenes bearing conjugated alkenes (Scheme 2, conditions C). Subsequent, unprecedented iodine-mediated, carbocyclative dimerization conveniently delivers novel, substituted benzodithiophenes **22–28** in moderate to excellent yield (3 steps overall from sulfoxide). The structure of **22** was confirmed by X-ray crystallography.^13^ Crucially, the alkyl substituents on the central benzene ring, and the electronic properties of the flanking benzene rings, can be readily varied through judicial choice of propargylsilane and sulfoxide coupling partners in the metal-free approach (Scheme 7A). A proposed mechanism for the novel dimerization is shown in Scheme 7B: homocoupling of the activated alkene **29** followed by acid-mediated cyclization gives intermediate **30***en route* to dimers **22–28**.

We have carried out preliminary evaluation of the materials prepared. Cyclic voltammetry and UV/Vis spectroscopy were used to determine the HOMO/LUMO levels and energy gaps for compounds **11–16** and **22–28**. In particular, compounds **11–14** showed a low lying HOMO of <–5.5 eV and energy band gap greater than 3 eV, consistent with previous reports suggesting the *orientation* of the thiophene ring has little effect on the electronic structure of the core.9*h* As expected, a lowering of the HOMO/LUMO was observed for the acyl-substituted derivatives.

Thin films of compounds **11–14** were characterised using X-ray diffraction and atomic force microscopy, and organic field effect transistors (OFETs) were prepared and analysed using the conventional thin film transistor techniques.^15^ Consistent with previous observations,9d,g,h novel materials **11** and **13** formed smooth thin films and demonstrated p-type (hole transporting) behaviour in a field effect transistor. In particular, compound **11** demonstrated a mobility of 0.2 cm^2^ V^–1^ s^–1^, high current on/off ratio of 10^7^ and a threshold voltage of –22 V over an average of 9 devices. This performance is consistent with those reported for fused benzothiophene semiconductors.9*h*

## Conclusions

In conclusion, a metal-free approach allows expedient access to benzothiophene-based systems that are components of important materials or are proven organic materials in their own right. The approach combines sulfoxide-directed metal-free C–H cross-couplings with novel tuneable electrophile-mediated heterocyclizations and carbocyclative dimerizations. As benzothiophene-based materials are typically prepared using Pd-catalyzed cross-coupling processes, our approach allows potential issues of metal cost and supply, and the metal-contamination of products, to be avoided.

## Supplementary Material

Supplementary information

Crystal structure data
