# Supplementary material for: Sulfoxide-directed metal-free cross-couplings in the expedient synthesis of benzothiophene-based components of materials
Source: Chem Sci. 2015 Nov 9;7(2):1281–5. doi: 10.1039/c5sc03823e (PMC5975836; doi:10.1039/c5sc03823e)

Electronic Supplementary Material (ESI) for Chemical Science.
This journal is © The Royal Society of Chemistry 2015

Supporting Information

Sulfoxide-directed metal-free cross-couplings in the expedient synthesis of benzothiophene-based components of materials

A. J. Eberhart, H. Shrives, Y. Zhang, A. Carrër, A. Parry, D. Tate, M. L. Turner, D. J. Procter*

School of Chemistry, University of Manchester, Oxford Road, Manchester, M13 9PL, UK.

david.j.procter@manchester.ac.uk

Contents

General Procedure A – Condition A 3

General Procedure B – Conditions B1 8

Gerenal Procedure C – Conditions B2 11

General Procedure D – Conditions C 13

General procedure E: Oxidation to bis-sulfoxide 19

General Procedure F- 2D Propargylation... 20

General Procedure G – Cyclisation to Alkane 22

General Procedure H- Cyclisation to Ketone 23

General Procedure I- Cyclisation to Alkene 25

General Procedure J- Alkene Dimerisation.. 30

^{1}H and ^{13}C NMR Spectra.. 36

Field Effect Transistors 85

UV/Vis Spectra and Cyclic Voltammetry... 91

X-Ray Structures and CCDC Numbers.. 110

1. Experimental Section

General Procedure A – Condition A

1-(5-Methylbenzo[*b*]thiophen-2-yl)pentan-1-one S1

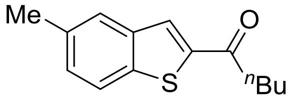

As described in general procedure A, (2-(hept-2-yn-1-yl)-4-methylphenyl)(methyl)sulfide (11.7 mg, 0.050 mmol), iodine (8.9 mg, 0.035 mmol) in toluene (2.0 mL), after purification by preparative thin-layer chromatography on silica gel eluting with 10% EtOAc in *n*-hexane, gave the product (8.8 mg, 38.0 μmol 76% yield) as a yellow solid (122 - 124 °C); ν_{max} (neat)/cm^{-1} 2959, 2932, 2862, 1661, 1520, 1465, 1452, 1441, 1406, 1277, 1188, 1178, 1109, 904, 861, 799, 736, 726; δ_H (400 MHz, $CDCl_3$) 0.98 (3H, t, *J* = 7.3, C*H*$_3$), 1.45 (2H, sxt, *J* = 7.4, C*H*$_2$CH$_3$), 1.78 (2H, quin, *J* = 7.5, CCH$_2$C*H*$_2$), 2.48 (3H, s, Ar-C*H*$_3$), 3.00 (2H, t, *J* = 7.5, CC*H*$_2$CH$_2$), 7.30 (1H, d, *J* 8.3, Ar-*H*), 7.68 (1H, s, Ar-*H*), 7.75 (1H, d, *J* 8.3, Ar-*H*), 7.88 (1H, s, Ar-*H*); δ_C (100 MHz, $CDCl_3$) 13.9 (CH$_2$*C*H$_3$), 21.3 (Ar-*C*H$_3$), 22.5 (*C*H$_2$CH$_3$), 26.9 (CCH$_2$*C*H$_2$), 39.0 (C*C*H$_2$CH$_2$), 122.6 (Ar-*C*H), 125.5 (Ar-*C*H), 128.5 (Ar-*C*H), 129.4 (Ar-*C*H), 135.0 (Ar-*C*), 139.5 (Ar-*C*), 139.7 (Ar-*C*), 144.2 (Ar-*C*), 195.3 (*C*=O); *m/z* (EI) M, 232; (Found: M, 232.0918. $C_{14}H_{16}OS$ requires M, 232.0916).

1-(7-Methylbenzo[*b*]thiophen-2-yl)pentan-1-one S2

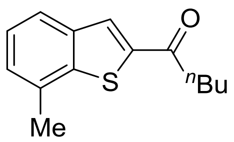

As described in general procedure A, (2-(hept-2-yn-1-yl)-6-methylphenyl)(methyl)sulfide (11.7 mg, 0.050 mmol), iodine (8.9 mg, 0.035 mmol) in toluene (2.0 mL), after purification by preparative thin-layer chromatography on silica gel eluting with 10% EtOAc in *n*-hexane, gave the product (10.0 mg, 43.0 μmol, 86% yield) as a white solid (mp: 74 - 75 °C); ν_{max} (neat)/cm^{-1} 2950, 2925, 2868, 1657, 1523, 1465, 1406, 1371, 1277, 1181, 1107, 870, 771, 722; δ_H (400 MHz, $CDCl_3$) 0.98 (3H, t, *J* = 7.3, C*H*$_3$), 1.46 (2H, sxt, *J* = 7.4, C*H*$_2$CH$_3$), 1.79 (2H,

quin, J = 7.5, CCH_2CH_2), 2.58 (3H, s, Ar-CH_3), 3.02 (2H, t, J = 7.5, CCH_2CH_2), 7.24 - 7.30 (1H, m, Ar-H), 7.35 (1H, t, J = 7.3, Ar-H), 7.75 (1H, d, J = 7.9, Ar-H), 7.99 (1H, s, Ar-H); δ_C (100 MHz, $CDCl_3$) 13.9 (CH_2CH_3), 20.2 (Ar-CH_3), 22.5 (CH_2CH_3), 26.9 (CCH_2CH_2), 39.0 (CCH_2CH_2), 123.5 (Ar-CH), 125.4 (Ar-CH), 127.3 (Ar-CH), 129.4 (Ar-CH), 132.6 (Ar-C), 139.0 (Ar-C), 142.8 (Ar-C), 143.4 (Ar-C), 195.1 (C=O); *m/z* (EI) M, 232; (Found: M, 232.0929. $C_{14}H_{16}OS$ requires M, 232.0916).

1-(5-Fluorobenzo[*b*]thiophen-2-yl)pentan-1-one S3

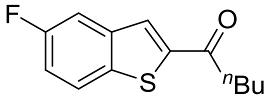

As described in general procedure A, (4-fluoro-2-(hept-2-yn-1-yl)phenyl)(methyl)sulfide (11.8 mg, 0.050 mmol), iodine (8.9 mg, 0.035 mmol) in toluene (2.0 mL), after purification by preparative thin-layer chromatography on silica gel eluting with 10% EtOAc in *n*-hexane, gave the product (10.3 mg, 43.5 µmol, 87% yield) as a colourless solid (mp: 118 - 121°C); ν_{max} (neat)/cm^{-1} 2954, 2926, 2854, 1670, 1521, 1437, 1274, 1264, 1191, 1182, 895, 806, 764, 736, 718, 705, 668; δ_H (400 MHz, $CDCl_3$) 0.98 (3H, t, J = 7.3, CH_3), 1.45 (2H, sxt, J = 7.4, CH_2CH_3), 1.78 (2H, quin, J = 7.5, CCH_2CH_2), 3.01 (2H, t, J = 7.4, CCH_2CH_2), 7.24 (1H, td, J = 8.8, 2.5, Ar-H), 7.55 (1H, dd, J = 9.0, 2.4, Ar-H), 7.82 (1H, dd, J = 8.9, 4.7, Ar-H), 7.90 (1H, s, Ar-H); δ_C (100 MHz, $CDCl_3$) 13.9 (CH_2CH_3), 22.4 (CH_2CH_3), 26.7 (CCH_2CH_2), 39.1 (CCH_2CH_2), 110.8 (d, J = 22.7, Ar-CH), 116.5 (d, J = 24.9, Ar-CH), 124.3 (d, J = 9.5, Ar-CH), 128.0 (d, J 4.4, Ar-CH), 137.9 (d, J = 1.5, Ar-C), 140.0 (d, J = 9.5, Ar-C), 146.2 (Ar-C), 160.9 (d, J = 243.6, Ar-CF), 194.8 (C=O); *m/z* (EI) M, 236; (Found: M, 236.0671. $C_{13}H_{13}OFS$ requires M, 236.0666).

1-(5-Chlorobenzo[*b*]thiophen-2-yl)pentan-1-one S4

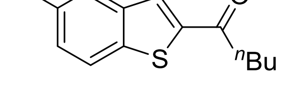

As described in general procedure A, (4-chloro-2-(hept-2-yn-1-yl)phenyl)(methyl)sulfide (12.6 mg, 0.050 mmol), iodine (8.9 mg, 0.035 mmol) in toluene (2.0 mL), after purification by preparative thin-layer chromatography on silica gel eluting with 10% EtOAc in *n*-hexane, gave the product (10.6 mg, 42.0 µmol, 84% yield) as a yellow solid (mp: 132 - 136 °C); ν_{max}

(neat)/cm^{-1} 2957, 2922, 2853, 1659, 1512, 1465, 1405, 1274, 1267, 1261, 1079, 902, 802, 764, 750, 737, 720; δ_H (400 MHz, $CDCl_3$) 0.98 (3H, t, *J* = 7.3, C*H*$_3$), 1.45 (2H, sxt, *J* = 7.4, C*H*$_2$CH$_3$), 1.78 (2H, quin, *J* = 7.5, CCH$_2$C*H*$_2$), 3.00 (2H, t, *J* = 7.4, CC*H*$_2$CH$_2$), 7.42 (1H, dd, *J* = 8.7, 2.0, Ar-*H*), 7.80 (1H, d, *J* = 8.7, Ar-*H*), 7.84 - 7.89 (1H, m, Ar-*H*); δ_C (100 MHz, $CDCl_3$) 14.1 (CH$_2$*C*H$_3$), 22.7 (*C*H$_2$CH$_3$), 26.9 (CCH$_2$*C*H$_2$), 39.3 (C*C*H$_2$CH$_2$), 124.3 (Ar-*C*H), 125.3 (Ar-*C*H), 127.8 (Ar-*C*H), 128.0 (Ar-*C*H), 131.4 (Ar-*C*), 140.4 (Ar-*C*), 140.6 (Ar-*C*), 146.0 (Ar-*C*), 195.0 (*C*=O); *m/z* (EI) M, 252 ^{35}Cl, 254 ^{37}Cl; (Found: M, 252.0377. $C_{13}H_{13}OClS$ requires M, 252.0370).

1-(5-Nitrobenzo[*b*]thiophen-2-yl)pentan-1-one S5

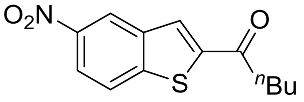

As described in general procedure A, (2-(hept-2-yn-1-yl)-4-nitrophenyl)(methyl)sulfide (13.2 mg, 0.050 mmol), iodine (8.9 mg, 0.035 mmol) in toluene (2.0 mL), after purification by preparative thin-layer chromatography on silica gel eluting with 10% EtOAc in *n*-hexane, gave the product (9.9 mg, 37.5 µmol, 75% yield) as a yellow solid (mp: 116 - 119 °C); ν_{max} (neat)/cm^{-1} 3071, 2967, 2947, 2930, 2850, 1659, 1599, 1576, 1531, 1517, 1467, 1405, 1349, 1268, 1177, 924, 826, 740, 735; δ_H (400 MHz, $CDCl_3$) 0.99 (3H, t, *J* = 7.4, C*H*$_3$), 1.47 (2H, sxt, *J* = 7.5, C*H*$_2$CH$_3$), 1.80 (2H, quin, *J* = 7.5, CCH$_2$C*H*$_2$), 3.05 (2H, t, *J* = 7.4, CC*H*$_2$CH$_2$), 8.01 (1H, d, *J* 9.0, Ar-*H*), 8.08 (1H, s, Ar-*H*), 8.31 (1H, dd, *J* = 8.9, 2.1, Ar-*H*), 8.81 (1H, d, *J* = 2.0, Ar-*H*); δ_C (100 MHz, $CDCl_3$) 14.1 (CH$_2$*C*H$_3$), 22.6 (*C*H$_2$CH$_3$), 26.8 (CCH$_2$*C*H$_2$), 39.3 (C*C*H$_2$CH$_2$), 121.4 (Ar-*C*H), 121.7 (Ar-*C*H), 124.0 (Ar-*C*H), 128.9 (Ar-*C*H), 139.1 (Ar-*C*), 146.1 (Ar-*C*), 147.7 (Ar-*C*), 147.8 (Ar-*C*), 194.7 (*C*=O); *m/z* (EI) M, 263; (Found: M, 263.0612. $C_{13}H_{13}O_3NS$ requires M, 263.0616).

1-(5-Methoxybenzo[*b*]thiophen-2-yl)pentan-1-one S6

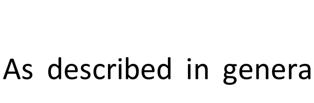

As described in general procedure A, (2-(hept-2-yn-1-yl)-4-methoxyphenyl)(methyl)sulfide (12.4 mg, 0.050 mmol), iodine (8.9 mg, 0.035 mmol) in toluene (2.0 mL), after purification by

preparative thin-layer chromatography on silica gel eluting with 15% EtOAc in *n*-hexane, gave the product (9.6 mg, 38.5 µmol, 77% yield) as a yellow solid (mp: 103 - 107 °C); ν_{max} (neat)/cm^{-1} 2963, 2953, 2864, 1657, 1598, 1515, 1456, 1441, 1407, 1374, 1336, 1275, 1268, 1232, 1201, 1178, 1158, 1148, 1071, 1031, 879, 812, 764, 749, 720; δ_H (400 MHz, $CDCl_3$) 0.98 (3H, t, *J* = 7.4, C*H*$_3$), 1.45 (2H, sxt, *J* = 7.3, C*H*$_2$CH$_3$), 1.78 (2H, quin, *J* = 7.5, CCH$_2$C*H*$_2$), 2.99 (2H, t, *J* = 7.5, CC*H*$_2$CH$_2$), 3.89 (3H, s, OC*H*$_3$), 7.13 (1H, dd, *J* = 8.8, 2.5, Ar-*H*), 7.30 (1H, d, *J* 2.3, Ar-*H*), 7.74 (1H, d, *J* = 9.0, Ar-*H*), 7.88 (1H, s, Ar-*H*); δ_C (100 MHz, $CDCl_3$) 14.1 (CH$_2$*C*H$_3$), 22.7 (*C*H$_2$CH$_3$), 27.1 (CCH$_2$*C*H$_2$), 39.3 (C*C*H$_2$CH$_2$), 55.8 (O*C*H$_3$), 107.0 (Ar-*C*H), 118.6 (Ar-*C*H), 123.9 (Ar-*C*H), 128.6 (Ar-*C*H), 135.4 (Ar-*C*), 140.4 (Ar-*C*), 145.1 (Ar-*C*), 158.0 (Ar-*C*), 195.2 (*C*=O); *m/z* (EI) M, 248; (Found: M, 248.0857. $C_{14}H_{16}O_2S$ requires M, 248.0866).

1-(7-Methoxybenzo[*b*]thiophen-2-yl)pentan-1-one S7

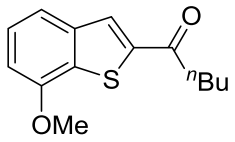

As described in general procedure A, (2-(hept-2-yn-1-yl)-6-methoxyphenyl)(methyl)sulfide (12.4 mg, 0.050 mmol), iodine (8.9 mg, 0.035 mmol) in toluene (2.0 mL), after purification by preparative thin-layer chromatography on silica gel eluting with 15% EtOAc in *n*-hexane, gave the product (10.1 mg, 41.0 µmol, 82% yield) as a yellow solid (mp: 67 - 69 °C); ν_{max} (neat)/cm^{-1} 2957, 2930, 2870, 1661, 1567, 1526, 1469, 1438, 1404, 1347, 1322, 1260, 1218, 1191, 1167, 1093, 967, 918, 846, 800, 771, 716, 658, 602; δ_H (400 MHz, $CDCl_3$) 0.97 (3H, t, *J* = 7.3, C*H*$_3$), 1.45 (2H, sxt, *J* = 7.4, C*H*$_2$CH$_3$), 1.78 (2H, quin, *J* = 7.5, CCH$_2$C*H*$_2$), 3.00 (2H, t, *J* = 7.4, CC*H*$_2$CH$_2$), 4.01 (3H, s, OC*H*$_3$), 6.87 (1H, d, *J* = 7.8, Ar-*H*), 7.36 (1H, t, *J* = 7.9, Ar-*H*), 7.50 (1H, d, *J* = 8.0, Ar-*H*), 7.95 (1H, s, Ar-*H*); δ_C (100 MHz, $CDCl_3$) 13.9 (CH$_2$*C*H$_3$), 22.5 (*C*H$_2$CH$_3$), 26.8 (CCH$_2$*C*H$_2$), 39.1 (C*C*H$_2$CH$_2$), 55.7 (O*C*H$_3$), 106.3 (Ar-*C*H), 118.1 (Ar-*C*H), 126.2 (Ar-*C*H), 128.9 (Ar-*C*H), 131.8 (Ar-*C*), 140.7 (Ar-*C*), 144.2 (Ar-*C*), 154.6 (Ar-*C*), 195.0 (*C*=O); *m/z* (EI) M, 248; (Found: M, 248.0865. $C_{14}H_{16}O_2S$ requires M, 248.0866).

1-(7-(Trifluoromethyl)benzo[*b*]thiophen-2-yl)pentan-1-one S8

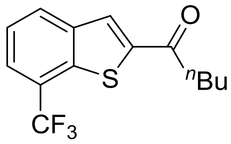

As described in general procedure A, (2-(hept-2-yn-1-yl)-6-(trifluoromethyl)phenyl)(methyl)sulfide (14.3 mg, 0.050 mmol), iodine (8.9 mg, 0.035 mmol) in toluene (2.0 mL), after purification by preparative thin-layer chromatography on silica gel eluting with 10% EtOAc in *n*-hexane, gave the product (13.0 mg, 45.5 µmol, 91% yield) as a yellow solid (mp: 57 - 59 °C); ν_{max} (neat)/cm^{-1} 2970, 2945, 1662, 1341, 1300, 1275, 1261, 1203, 1172, 1131, 1116, 1089, 949, 873, 764, 750, 725; δ_H (400 MHz, $CDCl_3$) 0.98 (3H, t, *J* = 7.3, C*H*$_3$), 1.45 (2H, sxt, *J* = 7.6, C*H*$_2$CH$_3$), 1.79 (2H, quin, *J* = 7.6, CCH$_2$C*H*$_2$), 3.03 (2H, t, *J* = 7.3, CC*H*$_2$CH$_2$), 7.52 (1H, t, *J* = 8.1, Ar-*H*), 7.77 (1H, d, *J* = 7.6, Ar-*H*), 8.01 (1H, s, Ar-*H*), 8.07 (1H, t, *J* = 8.1, Ar-*H*); δ_C (100 MHz, $CDCl_3$) 13.9 (CH$_2$*C*H$_3$), 22.4 (*C*H$_2$CH$_3$), 26.7 (CCH$_2$*C*H$_2$), 39.3 (C*C*H$_2$CH$_2$), 123.8 (q, *J* = 272.9, *C*F$_3$), 124.7 (Ar-*C*H), 124.9 (q, *J* = 4.4, Ar-*C*H), 125.4 (q, *J* 33.7, Ar-*C*), 127.8 (Ar-*C*H), 129.3 (Ar-*C*H), 138.3 - 138.4 (m, Ar-*C*), 140.8 (Ar-*C*), 145.1 (Ar-*C*), 194.6 (*C*=O); *m/z* (EI) M, 286; (Found: M, 286.0632. $C_{14}H_{13}OF_3S$ requires M, 286.0639).

1-(5-(Trifluoromethyl)benzo[*b*]thiophen-2-yl)pentan-1-one S9

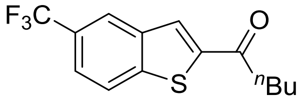

As described in general procedure A, (2-(hept-2-yn-1-yl)-4-(trifluoromethyl)phenyl)(methyl)sulfide (28.6 mg, 0.100 mmol), iodine (17.8 mg, 0.070 mmol) heated in toluene (4.0 mL) at 80 °C for 36 h, after purification by preparative thin-layer chromatography on silica gel eluting with 10% EtOAc in *n*-hexane, gave the product (22.6 mg, 79.0 µmol, 79% yield) as a white solid (mp: 99 - 101 °C); ν_{max} (neat)/cm^{-1} 2982, 2937, 2856, 1663, 1607, 1525, 1470, 1456, 1446, 1406, 1338, 1326, 1276, 1186, 1112, 1078, 1053, 920, 862, 821, 732; δ_H (500 MHz, $CDCl_3$) 0.98 (3H, t, *J* = 7.4, C*H*$_3$), 1.46 (2H, sxt, *J* = 6.9, C*H*$_2$CH$_3$), 1.80 (2H, quin, *J* = 7.6, CCH$_2$C*H*$_2$), 3.03 (2H, t, *J* = 7.6, CC*H*$_2$CH$_2$), 7.68 (1H, dd, *J* = 8.5, 1.3, Ar-*H*), 7.96 - 8.03 (2H, m, 2 × Ar-*H*), 8.18 (1H, s, Ar-*H*); δ_C (125 MHz, $CDCl_3$) 13.9 (CH$_2$*C*H$_3$), 22.4 (*C*H$_2$CH$_3$), 26.7 (CCH$_2$*C*H$_2$), 39.1 (C*C*H$_2$CH$_2$), 123.0 (q, *J* = 4.3, Ar-*C*H), 123.3 (q, *J*

= 3.4, Ar-*C*H), 124.2 (q, *J* = 272.5, CF_3), 123.7 (Ar-*C*H), 127.6 (q, *J* = 32.7, Ar-*C*), 128.3 (Ar-*C*H), 138.7 (Ar-*C*), 145.1 (Ar-*C*), 146.0 (Ar-*C*), 194.7 (*C*=O); *m/z* (EI) M, 286; (Found: M, 286.0627. $C_{14}H_{13}OF_3S$ requires M, 286.0634).

3-Ethylbenzo[*b*]thiophene-2-carbaldehyde S10

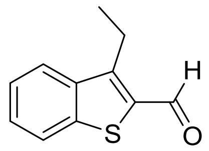

Methyl(2-(pent-1-yn-3-yl)phenyl)sulfide (0.02 g, 0.1 mmol), O_2 sparged toluene (2 mL) and I_2 (0.017 g, 0.07 mmol) was added to an oven-dried tube fitted with a magnetic stirrer bar and under a nitrogen atmosphere. The resulting mixture was sparged with O_2 for a further 10 min before heating to 80 ^{o}C for 18 h. Saturated sodium thiosulfate solution (4 mL) was then added, the organic layer was separated and the aqueous layer extracted with Et_2O (3 x 2 ml). The combined organic layers were then washed with brine (2 mL), dried ($MgSO_4$), filtered and the solvent removed *in vacuo.* The resulting mixture was purified by column chromatography using hexanes to give the product as a white solid (0.009 g, 0.052 mmol, 52%). ν_{max} (neat)/cm^{-1} 2967, 2918, 2832, 1651, 1560, 1524, 1448, 1350, 1247, 1163, 1088, 972, 850, 762, 709, 614, 544; δ_H (400 MHz, $CDCl_3$) 1.42 (3 H, t, *J* = 7.6 Hz, C*H*$_3$), 3.30 (2 H, q, *J* = 7.6 Hz, C*H*$_2$), 7.45 (1 H, t, *J* = 7.7 Hz, ArC*H*), 7.52 (1 H, t, *J* = 7.2 Hz, ArC*H*), 7.89 (1 H, d, *J* = 8.1 Hz, ArC*H*), 7.93 (1 H, d, *J* = 8.1 Hz, ArC*H*) 10.34 (1 H, s, C*H*O); δ_C (100 MHz, $CHCl_3$) 16.2 (*C*H$_3$), 20.0 (*C*H$_2$), 123.5 (Ar*C*H), 123.9 (Ar*C*H), 124.8 (Ar*C*H), 128.3 (Ar*C*H), 137.0 (Ar*C*), 139.1 (Ar*C*), 142.6 (Ar*C*), 149.7 (Ar*C*), 183.8 (*C*HO); *m/z* (GCMS) 190.0; (Found: M, 190.0446, $C_{11}H_{10}O_1S_1$ requires M, 190.0447).

General Procedure B – Conditions B1

2-Pentylbenzo[*b*]thiophene S11

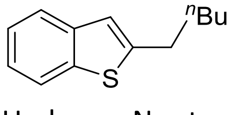

Under an N_2 atmosphere a solution of iodine (17.8 mg, 0.07 mmol) in toluene (2.0 mL) was added to a solution of (2-(hept-2-yn-1-yl)phenyl)(methyl)sulfide (21.8 mg, 0.100 mmol) and

1,4-cycloheaxadiene (20.0 mg, 0.25 mmol) in toluene (2.0 mL) at room temperature. The reaction mixture was stirred for 18 h at 80 °C before diluting with Et_2O (5 mL) and quenching with saturated aqueous $Na_2S_2O_3$ (5 mL). The aqueous layer was then extracted with Et_2O (2 × 5 mL) and the combined organic layers washed with brine (5 mL), dried (Na_2SO_4) and concentrated *in vacuo*. The crude product was purified by preparative thin-layer chromatography on silica gel eluting with *n*-hexane to yield the product (17.9 mg, 88% yield) as a yellow oil; ν_{max} (neat)/cm^{-1} 2955, 2928, 2856, 1457, 1436, 1275, 1261, 1067, 1015, 855, 818, 764, 746, 726; δ_H (500 MHz, $CDCl_3$) 0.89 - 0.95 (3H, m, C*H*$_3$), 1.34 - 1.43 (4H, m, 2 × CH_2), 1.73 - 1.81 (2H, m, CCH$_2$C*H*$_2$), 2.88 - 2.94 (2H, m, CC*H*$_2$CH$_2$), 7.00 - 7.02 (1H, m, Ar-*H*), 7.23 - 7.28 (1H, m, Ar-*H*), 7.31 (1H, td, *J* = 7.6, 1.1, Ar-*H*), 7.67 (1H, d, *J* = 7.6, Ar-*H*), 7.78 (1H, d, *J* = 7.6, Ar-*H*); δ_C (125 MHz, $CDCl_3$) 14.0 (*C*H$_3$), 22.4 (*C*H$_2$), 30.7 (C*C*H$_2$CH$_2$), 30.8 (CCH$_2$*C*H$_2$), 31.3 (*C*H$_2$), 120.4 (Ar-*C*H), 122.1 (Ar-*C*H), 122.6 (Ar-*C*H), 123.3 (Ar-*C*H), 124.0 (Ar-*C*H), 139.3 (Ar-*C*), 140.2 (Ar-*C*), 146.9 (Ar-*C*); *m/z* (EI) M, 204; (Found: M, 204.0970. $C_{13}H_{16}S$ requires M, 204.0967).

5-Methoxy-2-pentylbenzo[*b*]thiophene S12

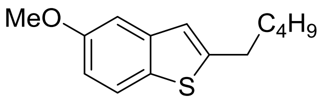

As described in general procedure B. (2-(hept-2-yn-1-yl)-4-methoxyphenyl)(methyl)sulfide (24.0 mg, 0.1 mmol), toluene (4 mL), I_2 (17.0 mg, 0.07 mmol) and 1,4-cyclohexadiene (0.014 mL, 0.15 mmol) were added to an oven-dried tube and the resulting mixture was heated at 80 ^{o}C for 18 h. The resulting mixture was purified by column chromatography using 10% EtOAc in hexanes to give the product as a yellow solid (14.0 mg, 0.069 mmol, 69%); ν_{max} (neat)/cm^{-1} 2955, 2918, 2849, 1596, 1568, 1457, 1377, 1269, 1198, 1154, 1069, 938, 854, 773, 694, 675, 579; δ_H (400 MHz, $CDCl_3$) 0.92 (3 H, t, *J* = 6.8 Hz, C*H*$_3$), 1.35 - 1.42 (4 H, m, 2 x C*H*$_2$), 1.75 (2 H, quin, *J* = 7.3 Hz, C*H*$_2$), 2.88 (2 H, t, *J* = 7.5 Hz, C*H*$_2$), 3.86 (3 H, s, OC*H*$_3$), 6.88 - 6.95 (2 H, m, 2 x ArC-*H*), 7.15 (1 H, d, *J* = 2.2 Hz, ArC-*H*), 7.62 (1 H, d, *J* = 8.8 Hz, ArC-*H*); δ_C (100 MHz, $CDCl_3$) 14.0 (*C*H$_3$), 22.4 (*C*H$_2$), 30.8 (*C*H$_2$), 30.8 (*C*H$_2$), 31.2 (*C*H$_2$), 55.5 (O*C*H$_3$),. 105.2 (Ar*C*H), 113.2 (Ar*C*H), 120.3 (Ar*C*H), 122.7 (Ar*C*H), 131.5 (Ar*C*), 141.2 (Ar*C*), 148.3 (Ar*C*), 157.3 (Ar*C*); m/z (GCMS) 234.1; (Found: M, 234.1074, $C_{14}H_{18}O_1S_1$ requires M, 234.1073).

5-Chloro-2-pentylbenzo[*b*]thiophene S13

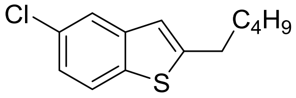

To an oven dried tube fitted with a magnetic stirrer bar and under a nitrogen atmosphere was added (2-(hept-2-yn-1-yl)-4-chlorophenyl)(methyl)sulfide (25.0 mg, 0.1 mmol), toluene (4 mL), I_2 (17.0 mg, 0.07 mmol) and 1,4-cyclohexadiene (0.014 mL, 0.15 mmol). The resulting mixture was heated at 80 ^{o}C for 18 h before the addition of saturated sodium thiosulfate solution (4 mL). The organic layer was separated and the aqueous layer extracted with Et_2O (3 x 2 mL). The combined organic layers were then washed with brine (2 mL), dried with $MgSO_4$, filtered and the solvent removed *in vacuo.* The resulting mixture was purified by column chromatography using 10% EtOAc in hexanes to give the product as a yellow solid (17.5 mg, 0.075 mmol, 75%). ν_{max} (neat)/cm^{-1} 2952, 2925, 2857, 1580, 1560, 1465, 1417, 1375, 1206, 1180, 1074, 903, 882, 803, 734, 667 597, 574; δ_H (400 MHz, $CDCl_3$) 0.9 (3 H, t, *J* = 6.8 Hz, C*H*$_3$), 1.3 - 1.4 (4 H, m, 2 x C*H*$_2$), 1.8 (2 H, quin, *J* = 7.3 Hz, C*H*$_2$), 2.9 (2 H, t, *J* = 7.5 Hz, C*H*$_2$), 6.9 (1 H, s, ArC*H*), 7.2 (1 H, dd, *J* = 8.6, 2.0 Hz, ArC*H*), 7.6 - 7.7 (2 H, m, 2 x ArC*H*); δ_C (100 MHz, $CDCl_3$) 14.0 (*C*H$_3$), 22.4 (*C*H$_2$), 30.7 (*C*H$_2$), 30.8 (*C*H$_2$), 31.2 (*C*H$_2$), 119.8 (Ar*C*H), 122.2 (Ar*C*H), 123.0 (Ar*C*H), 123.7 (Ar*C*H), 130.2 (Ar*C*), 137.3 (Ar*C*), 141.3 (Ar*C*), 149.1 (Ar*C*); *m/z* (GCMS) ; (Found: M, , $C_{13}H_{15}F_1S_1$ requires M,).

2-Pentyl-5-(trifluoromethyl)benzo[*b*]thiophene S14

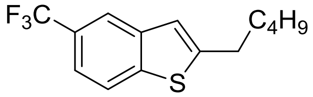

As described in general procedure B. (2-(hept-2-yn-1-yl)-4-trifluoromethylphenyl)(methyl)sulfide (28.0 mg, 0.1 mmol), toluene (4 mL), I_2 (17.0 mg, 0.07 mmol) and 1,4-cyclohexadiene (0.014 mL, 0.15 mmol) were added to an oven-dried tube and the resulting mixture was heated at 80 ^{o}C for 18 h. The resulting mixture was purified by column chromatography using 10% EtOAc in hexanes to give the product as a yellow solid (20.7 mg, 0.076 mmol, 76%); ν_{max} (neat)/cm^{-1} 2930, 1436, 1332, 1263, 1147, 1073, 911, 810, 761, 677, 609; δ_H (400 MHz, $CDCl_3$) 0.9 (1 H, t, *J* = 8.1 Hz, C*H*$_3$), 1.3 - 1.4 (4 H, m, 2 x C*H*$_2$), 1.8

(2 H, quin, *J* = 7.3 Hz, C*H*$_2$), 2.9 (2 H, t, *J* = 7.6 Hz, C*H*$_2$), 7.1 (1 H, s, ArC*H*), 7.5 (1 H, d, *J* = 8.3 Hz, ArC*H*), 7.9 (1 H, d, *J* = 8.4 Hz, ArC*H*), 7.9 (1 H, s, ArC*H*); δ_C (100 MHz, CDCl$_3$) 14.0 (*C*H$_3$), 22.4 (*C*H$_2$), 30.8 (*C*H$_2$), 30.8 (*C*H$_2$), 31.3 (C*C*H$_2$), 119.5 - 119.8 (m, 2 x Ar*C*-H), 120.5 (Ar*C*-H), 122.5 (Ar*C*-H), 126.6 (q, *J* = 31.5 Hz, Ar*C*-CF$_3$), 127.0 (q, J = 271.2, *C*F$_3$), 139.8 (Ar*C*), 142.4 (Ar*C*), 149.3 (Ar*C*); *m/z* (GCMS) 272.1; (Found: M, 272.0844, $C_{14}H_{15}F_3S_1$ requires M, 272.0841).

7-Fluoro-2-pentylbenzo[*b*]thiophene S15

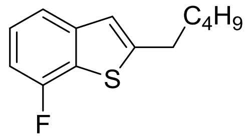

As described in general procedure B. (2-(hept-2-yn-1-yl)-6-fluorophenyl)(methyl)sulfide (24.0 mg, 0.2 mmol), toluene (4 mL), I_2 (17.0 mg, 0.07 mmol) and 1,4-cyclohexadiene (0.014 mL, 0.15 mmol) were added to an oven-dried tube and the resulting mixture was heated at 80 ^oC for 18 h. The resulting mixture was purified by column chromatography using 10% EtOAc in hexanes to give the product as a yellow solid (14.0 mg, 0.069 mmol, 69%); ν_{max} (neat)/cm^{-1} 2929, 2857, 1603, 1573, 1542, 1467, 1249, 1214, 1193, 913, 847, 828, 731, 585; δ_H (500 MHz, CDCl$_3$) 0.93 (3 H, t, *J* = 6.0 Hz, C*H*$_3$), 1.39 (4 H, d, *J* = 2.4 Hz, 2 x C*H*$_2$), 1.75 (2 H, quin, *J* = 6.8 Hz, C*H*$_2$), 2.88 (2 H, t, *J* = 7.6 Hz, CC*H*$_2$), 6.96 (1 H, s, ArC-*H*), 7.07 (1 H, t, *J* = 8.9 Hz, ArC-*H*), 7.46 (1 H, d, *J* = 8.9 Hz, ArC-*H*), 7.59 (1 H, dd, *J* = 8.4, 5.3 Hz, ArC-*H*); δ_C (100 MHz, CDCl$_3$) 13.7 (*C*H$_3$), 22.1 (*C*H$_2$), 30.4 (*C*H$_2$), 30.5 (*C*H$_2$), 31.0 (C*C*H$_2$), 108.0 (dd, *J* = 25.4, 1.0 Hz, Ar*C*-H), 112.5 (d, *J* = 24.5 Hz, Ar*C*-H), 119.5 (Ar*C*-H), 123.2 (d, *J* = 9.1 Hz, Ar*C*-H), 136.4 (d, *J* = 1.8 Hz, Ar*C*), 139.8 (d, *J* = 10.0 Hz, Ar*C*), 146.2 (d, *J* = 3.6 Hz, Ar*C*), 159.6 (d, *J* = 245.2 Hz, Ar*C*-F);); *m/z* (GCMS) 222.1; (Found: M, 222.0872, $C_{13}H_{15}F_1S_1$ requires M, 222.0873).

Gerenal Procedure C – Conditions B2

2-Methyl-3-ethylbenzothiophene S16

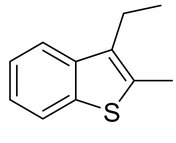

A microwave vial equipped with a magnetic stirrer, was charged with trimethyl(3-(2-methylsulfanyl)pent-1-yn-yl)silane (0.131 g, 0.5 mmol) and *para*-toluene sulfonic acid (95.1 mg, 0.5 mmol) and the mixture dissolved in EtOH (2.5 ml). The solution was then heated at 150 ^{o}C for 1 h 45 min in a microwave. The solution was quenched with aqueous saturated $NaHCO_3$ (6 mL) and the aqueous layer was extracted with Et_2O (3 x 5 mL). The combined organic layer was dried ($MgSO_4$) and concentrated *in vacuo.* The crude product was purified by column chromatography on silica gel eluting with 100% *n*-hexane to yield the product (49.0 mg, 56%); ν_{max} (neat)/cm^{-1} 2964, 2869, 1456, 1432, 1374, 1319, 1174, 1152, 1134, 1104, 1062, 1021, 925, 907, 808, 757, 729, 709, 638, 573; δ_H (400 MHz, $CDCl_3$) 1.23 (3 H, t, *J* = 7.6 Hz, CH_2CH_3), 2.51 (3 H, s, CH_3), 2.82 (2 H, q, *J* = 7.6 Hz, CH_2CH_3), 7.28 (1 H, t, *J* = 8.2 Hz, Ar-*H*), 7.35 (1 H, t, *J* = 7.9 Hz, Ar-*H*), 7.64 (1 H, d, *J* = 7.9 Hz, Ar-*H*), 7.77 (1 H, d, *J* = 7.8 Hz, Ar-*H*); δ_C (100 MHz, $CDCl_3$) 13.5 (CH_3), 14.1 (CH_2 CH_3), 19.5 (CH_2CH_3), 121.0 (Ar*C*-H), 122.1 (Ar*C*-H), 123.3 (Ar*C*-H), 123.7 (Ar*C*-H), 133.5 (Ar*C*), 138.4 (Ar*C*), 140.1 (Ar*C*); *m/z* (GCMS) 176.1; (Found: M, 176.0655, $C_{14}H_{18}O_1S_1$ requires M, 176.0654).

2-Methyl-3-isopropylbenzothiophene S17

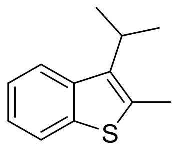

As in general procedure B2, Trimethyl(4-methyl-3-(2-(methylsulfanyl)phenyl)pent-1-yn-1-yl)silane (64.9 mg, 0.36 mmol) and *p*-TSA (68.4 mg, 0.36 mmol) in EtOH (2 ml) were heated at 150 ^{o}C in a microwave. The crude product was purified by column chromatography on silica gel eluting with 100% *n*-hexane to yield the product (28.4 mg, 0.15 mmol, 42%); ν_{max} (neat)/cm^{-1} 2961, 2952, 2870, 1456, 1432, 1384, 1363, 1189, 1173, 1106, 1066, 947, 760, 729, 674, 648; δ_H (400 MHz, $CDCl_3$) 1.46 (6 H, dd, J = 7.3, CH_3), 2.54 (3 H, s, CH_3), 3.42 (1 H, spt, *J* = 7.2 Hz, $CH(CH_3)_2$), 7.25 (1 H, t, *J* = 7.6 Hz, Ar-*H*), 7.32 (1 H, t, *J* = 8.1 Hz, Ar-*H*), 7.76 (1 H, d, *J* = 7.8 Hz, Ar-*H*), 7.83 (1 H, d, *J* = 8.1 Hz, Ar-*H*); δ_C (100 MHz, $CDCl_3$) 14.5 (CH_3), 21.5 ($CH(CH_3)_2$), 27.7 ($CH(CH_3)_2$), 122.1 (Ar*C*-H), 122.2 (Ar*C*-H), 122.9 (Ar*C*-H), 123.3 (Ar*C*-H), 132.9 (Ar*C*), 136.7 (Ar*C*), 138.5 (Ar*C*), 139.6 (Ar*C*); *m/z* (GCMS) 190.1; (Found: M, 190.0808, $C_{12}H_{14}S_1$ requires M, 190.0811).

2-Methyl-3-cyclohexylbenzothiophene S18

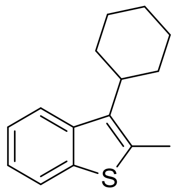

As in general procedure B2, (3-cyclohexyl-3-(2-methylsulfanyl)phenyl)prop-1-yn-1-yl)trimethylsilane (60.4 g, 0.2 mmol) and *p*-TSA (38.0 g, 0.2 mmol) in EtOH (2 ml) were heated at 150 ^{o}C in a microwave. The crude product was purified by column chromatography on silica gel eluting with 100% *n*-hexane to yield the product (37.2 mg, 0.16 mmol, 81%); ν_{max} (neat)/cm^{-1} 2923, 2849, 1447, 1431, 1173, 1150, 1136, 889, 757, 728, 641; δ_H (400 MHz, $CDCl_3$) 1.33 - 1.53 (4 H, m, 2 x C*H*$_2$), 1.76 - 2.07 (6 H, m, 3 x C*H*$_2$), 2.56 (3 H, s, C*H*$_3$), 3.01 (1H, tt, *J* = 12.4, 3.6 Hz, C*H*(CH_2)$_2$), 7.25 (1H, td, *J* = 7.0 Hz x 2, 1.26 Hz x 2, Ar-*H*), 7.32 (1H, td, J = 7.8 Hz x 2, 1.26 Hz x 2, Ar-*H*), 7.76 (1 H, d, *J* = 6.6 Hz), 7.88 (1 H, d, *J* = 8.1 Hz); δ_C (100 MHz, $CDCl_3$) 14.8 (*C*H$_3$), 26.3 (2 x *C*H$_2$), 27.3 (*C*H$_2$), 31.3 (2 x *C*H$_2$), 38.8 (*C*H), 122.1 (Ar*C*-H), 122.3 (Ar*C*-H), 122.8 (Ar*C*-H), 123.3 (Ar*C*-H), 133.2 (Ar*C*), 136.0 (Ar*C*), 138.4 (Ar*C*), 139.9 (Ar*C*); *m/z* 230.2; (Found: M, 230.1127, $C_{14}H_{18}O_1S_1$ requires M, 230.1124).

General Procedure D – Conditions C

(*E*)-2-(Pent-1-en-1-yl)benzo[*b*]thiophene S19

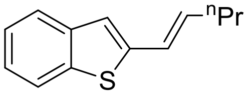

Under an Ar atmosphere, a solution of iodine (55.6 mg, 0.22 mmol) in Ar flushed 1,2-dichloroethane (2 mL) was added to a solution of (2-(hept-2-yn-1-yl)phenyl)(methyl)sulfide (43.6 mg, 0.2 mmol) in Ar flushed 1,2-dichloroethane (18 mL) at room temperature. The reaction mixture was stirred for 18 h at 80 °C before quenching with saturated aqueous $Na_2S_2O_3$ (5 mL). The aqueous layer was then extracted with EtOAc (3 x 5 mL) and the combined organic layers washed with brine (5 mL), dried (Na_2SO_4) and concentrated *in vacuo*. The crude product was purified by preparative thin-layer chromatography on silica

gel eluting with *n*-hexane to yield the product (37.5 mg, 0.18 mmol, 92 % yield) as a yellow solid (mp 38-40 °C); ν_{max} (neat)/cm^{-1} 2957, 2926, 2871, 1456, 1436, 1224, 1148, 1012, 950, 839, 839, 743, 725; δ_H (500 MHz, C_6D_6) 0.82 (3H, t, *J* = 7.3, C*H*$_3$), 1.29 (2H, sxt, *J* = 7.3, C*H*$_2$CH$_3$), 1.95 (2H, qd, *J* = 7.2, 1.4, CHC*H*$_2$), 6.14 (1H, dt, *J* = 15.7, 7.0, CCH=C*H*), 6.44 (1H, dt, *J* = 15.7, 1.2, CC*H*=CH), 6.81 (1H, s, Ar-*H*), 7.03 (1H, td, *J* = 7.6, 1.3, Ar-*H*), 7.12 (1H, td, *J* = 7.5, 1.2, Ar-*H*), 7.45 - 7.52 (2H, m, 2 × Ar-*H*); δ_C (125 MHz, C_6D_6) 14.1 (*C*H$_3$), 22.9 (*C*H$_2$CH$_3$), 35.6 (CH*C*H$_2$), 122.1 (Ar-*C*H), 122.8 (Ar-*C*H), 123.9 (Ar-*C*H), 124.9 (C*C*H=CH), 125.0 (2 × Ar-*C*H), 134.1 (CCH=*C*H), 139.5 (Ar-*C*), 141.2 (Ar-*C*), 143.9 (Ar-*C*); *m/z* (EI) M, 202; (Found: M, 202.0802. $C_{13}H_{14}S$ requires M, 202.0811).

(*E*)-5-Methyl-2-(pent-1-en-1-yl)benzo[*b*]thiophene S20

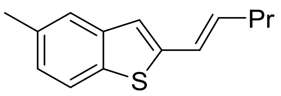

As described in general procedure C, (2-(hept-2-yn-1-yl)-4-methylphenyl)(methyl)sulfide (46.4 mg, 0.2 mmol), iodine (55.6 mg, 0.22 mmol) in 1,2-dichloroethane (20 mL), after purification by column chromatography on silica gel eluting with *n*-hexane, gave the product (34.7 mg, 0.16 mmol, 80 % yield) as a yellow solid (mp: 47-50 °C); ν_{max} (neat)/cm^{-1} 3012, 2954, 2924, 2867, 1443, 1378, 1301, 1259, 1230, 1209, 1169, 1138, 1065, 1044, 1008, 951, 889, 803, 744, 725, 694; δ_H (400 MHz, $CDCl_3$) 0.98 (3 H, t, *J* = 7.3 Hz, C*H*$_3$), 1.53 (2 H, sxt, *J* = 7.4 Hz, C*H*$_2$CH$_3$), 2.22 (2 H, qd, *J* = 7.2, 1.5 Hz, CHC*H*$_2$), 2.44 (3 H, s, C*H*$_3$), 6.15 (1 H, dt, *J* = 15.4, 7.0 Hz, CCH=C*H*), 6.60 (1 H, dd, *J* = 15.4, 0.5 Hz, CC*H*=CH), 6.98 (1 H, s, Ar-*H*), 7.10 (1 H, dd, *J* = 8.2, 1.1 Hz, Ar-*H*), 7.46 (1 H, s, Ar-*H*), 7.62 (1 H, d, *J* = 8.1 Hz, Ar-*H*); δ_C (100 MHz, $CDCl_3$) 13.4 (*C*H$_3$), 21.0 (*C*H$_2$CH$_3$), 22.0 (*C*H$_3$), 34.7 (CH*C*H$_2$), 120.6 (Ar-*C*H), 121.4 (Ar-*C*H), 122.8 (Ar-*C*H), 123.7 (C*C*H=CH), 125.6 (Ar-*C*H), 133.2 (Ar-*C*), 133.6 (CCH=*C*H), 135.2 (Ar-*C*), 140.2 (Ar-*C*), 143.1 (Ar-*C;*); *m/z* (GCMS) M, 216.1; (Found: M, 217.1051. $C_{14}H_{17}S$ requires M, 217.1050).

(E)-2-(3-Methylbut-1-en-1-yl)-5-(trifluoromethyl)benzo[b]thiophene S21

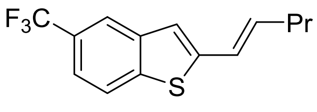

As described in general procedure C, (2-(hept-2-yn-1-yl)-4-trifluoromethylphenyl)(methyl)sulfide (57.2 mg, 0.2 mmol), iodine (55.6 mg, 0.22 mmol) in 1,2-dichloroethane (20 mL), after purification by column chromatography on silica gel eluting with 1% EtOAc in *n*-hexane, gave the product (18.0 mg, 0.09 mmol, 47 % yield) as a yellow oil; ν_{max} (neat)/cm^{-1} 2959, 2929, 1607, 1528, 1433, 1332, 1262, 1217, 1169, 1144, 1120, 1073, 1054 953, 906, 892, 812, 729, 709, 668, 650; δ_H (400 MHz, $CDCl_3$) 0.99 (3 H, t, *J* = 7.4 Hz, C*H*$_3$), 1.54 (2 H, sxt, *J* = 7.3 Hz, C*H*$_2$CH$_3$), 2.18 - 2.29 (2 H, q, *J* = 6.9, CHC*H*$_2$), 6.18 - 6.28 (1 H, m, CCH=C*H*), 6.62 (1 H, d, *J* = 15.8 Hz, CC*H*=CH), 7.10 (1 H, s, ArC-*H*), 7.48 (1 H, d, *J* = 8.5 Hz, ArC-*H*), 7.83 (1 H, d, *J* = 8.5 Hz, ArC-*H*), 7.91 (1 H, s, ArC-*H*); δ_C (100 MHz, $CDCl_3$13.8 (*C*H$_3$), 22.2 (*C*H$_2$CH$_3$), 35.1 (CH*C*H$_2$), 120.1 (q, *J* = 4.2 Hz, Ar*C*-H), 120.4 (q, *J* = 3.7 Hz, Ar*C*-H), 120.8 (Ar*C*-H), 122.5 (Ar*C*-H), 123.5 (C*C*H=CH), 124.5 (q, *J* = 272.9 Hz, *C*F$_3$), 126.9 (q, *J*=32.3 Hz, Ar*C*-CF$_3$), 135.3 (CCH=*C*H), 139.9 (Ar-*C*), 141.6 (Ar-*C*), 145.4 (Ar-*C*); *m/z* (GCMS) M, 270.0; (Found: M, 270.0681. $C_{14}H_{13}F_3S$ requires M, 270.0685).

(E)-5-Fluoro-2-(3-methylbut-1-en-1-yl)benzo[b]thiophene S22

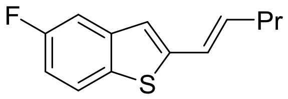

As described in general procedure C, (2-(hept-2-yn-1-yl)-4-fluorophenyl)(methyl)sulfide (47.2 mg, 0.2 mmol), iodine (55.6 mg, 0.22 mmol) in 1,2-dichloroethane (20 mL), after purification by column chromatography on silica gel eluting with *n*-hexane, gave the product (30.4 mg, 0.13 mmol, 65 % yield) as a yellow solid (mp 55-58 ^{0}C); ν_{max} (neat)/cm^{-1} 2954, 2925, 1595, 1443, 1379, 1230, 1209, 1170, 1121, 1066, 1045, 951,889,836, 803, 725, 715; δ_H (400 MHz, $CDCl_3$) 0.98 (3 H, t, *J* = 7.4 Hz, C*H*$_3$), 1.47 - 1.58 (2 H, m, C*H*$_2$CH$_3$), 2.18 - 2.26 (2 H, m, CHC*H*$_2$), 6.19 (1 H, dt, *J* = 15.5, 7.0 Hz, CCH=C*H*), 6.59 (1 H, d, *J* = 15.7 Hz, CC*H*=CH), 6.99 - 7.05 (2 H, m, ArC-*H*), 7.32 (1 H, dd, *J* = 9.5, 2.4 Hz, ArC-*H*), 7.65 (1 H, dd, *J* = 8.8, 4.9 Hz, ArC-*H*); δ_C (100 MHz, $CDCl_3$) 13.7 (*C*H$_3$), 22.2 (*C*H$_2$CH$_3$), 35.0 (CH*C*H$_2$), 108.6 (d, *J* = 23.5, Ar*C*-H), 112.7 (d, *J* = 29.3, Ar*C*-H), 120.7 (d, *J* = 4.4 Hz, Ar*C*-H), 123.1 (d, *J* = 9.5 Hz, Ar*C*), 123.7 (C*C*H=CH), 133.7 (Ar*C*), 134.6 (CCH=*C*H), 141.2 (d, *J* = 9.5 Hz, Ar*C*), 145.7 (Ar*C*), 160.8 (dd, *J* = 248.7, 1.0 Hz, Ar*C*-F); *m/z* (GCMS) M, 220.1; (Found: M, 220.0719. $C_{13}H_{13}F_1S_1$ requires M, 220.0717).

(E)-4-Fluoro-2-(3-methylbut-1-en-1-yl)benzo[b]thiophene S23

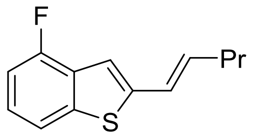

As described in general procedure C, (2-(hept-2-yn-1-yl)-5-fluorophenyl)(methyl)sulfide (47.2 mg, 0.2 mmol), iodine (55.6 mg, 0.22 mmol) in 1,2-dichloroethane (20 mL), after purification by column chromatography on silica gel eluting with *n*-hexane, gave the product (40.3 mg, 0.18 mmol, 92 % yield) as a yellow solid (mp 64-65 ^{o}C); ν_{max} (neat)/cm^{-1} 2958, 2929, 2872, 1589, 1565, 1520, 1464, 1401, 1378, 1248, 1233, 1188, 1146, 1111, 1046, 957, 938, 851, 819, 807, 718, 586; δ_H (400 MHz, $CDCl_3$) 0.97 (3 H, t, *J* = 7.4 Hz, CH_3), 1.45 - 1.58 (2 H, m, C$H_2$$CH_3$), 2.21 (2 H, qd, *J* = 7.2, 1.5 Hz, CHCH_2), 6.13 (1 H, dt, *J* = 15.6, 7.0 Hz, CCH=C*H*), 6.58 (1 H, d, *J* = 15.6 Hz, CC*H*=CH), 7.00 (1 H, s, ArC-*H*), 7.04 (1 H, td, *J* = 8.9, 2.4 Hz, ArC-*H*), 7.43 (1 H, dd, *J* = 8.8, 2.3 Hz, ArC-*H*), 7.58 (1 H, dd, *J* = 8.7, 5.2 Hz, ArC-*H*); δ_C (100 MHz, $CDCl_3$); 13.7 (*C*H_3), 22.3 (*C*$H_2$$CH_3$), 35.0 (*C*H$CH_2$), 108.3 (d, *J* = 24.9 Hz, Ar*C*-H), 113.1 (d, *J* = 23.5 Hz, Ar*C*-H), 120.3 (Ar*C*-H), 123.6 (CH=*C*HCH_2), 123.9 (d, *J* = 8.8 Hz, Ar*C*-H), 133.9 (d, *J* = 1.5 Hz, *C*H=CHCH_2), 136.7 (d, *J* = 1.5 Hz, Ar*C*), 139.4 (d, *J* = 10.3 Hz, Ar*C*), 143.0 (d, *J* = 3.7 Hz, Ar*C*), 160.5 (d, *J* = 243.6 Hz, Ar*C*-F); *m/z* (GCMS) M, 220.1; (Found: M, 220.0721. $C_{13}H_{13}F_1S_1$ requires M, 220.0717).

(E)-2-(3-Methylbut-1-en-1-yl)naphtho[2,1-b]thiophene S24

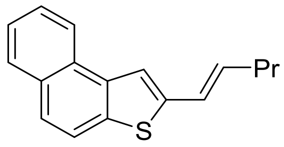

As described in general procedure C, (1-(hept-2-yn-1-yl)naphthalen-2-yl)(methyl)sulfide (53.6 mg, 0.2 mmol), iodine (55.6 mg, 0.22 mmol) in 1,2-dichloroethane (20 mL), after purification by column chromatography on silica gel eluting with *n*-hexane, gave the product (40.3 mg, 0.16 mmol 80 % yield) as a white solid (mp 83-85 ^{o}C); ν_{max} (neat)/cm^{-1} 2950, 2962, 2869, 2833, 1555, 1502, 1465, 1406, 1340, 1253, 1200, 1159, 1092, 1062, 1025, 955, 868, 843, 770, 712; δ_H (400 MHz, $CDCl_3$) 1.01 (3 H, t, *J* = 7.4 Hz, CH_3), 1.57 (2 H, m, C$H_2$$CH_3$), 2.26 (2 H, m, CHC$H_2$), 6.25 (1 H, dt, *J* = 15.5, 7.0 Hz, CCH=C*H*), 6.72 (1 H, d, *J* = 15.5Hz, CC*H*=CH),

7.52 (1 H, m, *J* = 6.8 Hz, ArC-*H*), 7.59 (1 H, t, *J* = 7.0 Hz, ArC-*H*), 7.73 (3 H, m, 3 x ArC-*H*), 7.92 (1 H, d, *J* = 7.9 Hz, ArC-*H*), 8.25 (1 H, d, *J* = 8.2 Hz, ArC-*H*); δ_C (100 MHz, $CDCl_3$) 13.8 (CH_3), 22.3 (CH_2CH_3), 35.0 ($CHCH_2$), 119.2 (Ar*C*-H), 120.5 (Ar*C*-H), 123.5 (Ar*C*-H), 123.9 (C*C*H=CH), 124.8 (Ar*C*-H), 125.1 (Ar*C*-H), 126.3 (Ar*C*-H), 128.5 (Ar*C*-H), 128.9 (Ar*C*), 131.0 (Ar*C*), 133.0 (CCH=*C*H), 135.8 (Ar*C*), 136.2 (Ar*C*), 143.1 (Ar*C*) ; *m/z* (GCMS) M, 252.1; (Found: M, 253.1056. $C_{17}H_{16}S$ requires M, 253.1050).

(*E*)-5-Methoxy-2-(pent-1-en-1-yl)benzo[*b*]thiophene S25

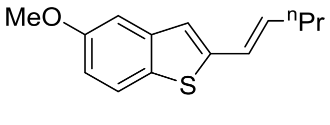

As described in general procedure C, (2-(hept-2-yn-1-yl)-4-methoxyphenyl)(methyl)sulfide (49.6 mg, 0.2 mmol), iodine (55.6 mg, 0.22 mmol) in 1,2-dichloroethane (20 mL), after purification by column chromatography on silica gel eluting with 5% EtOAc in *n*-hexane, gave the product (35.2 mg, 0.15 mmol, 76 % yield) as a yellow solid (mp 79-81 ^{o}C); ν_{max} (neat)/cm^{-1} 2957, 2921, 2872, 2850, 1571, 1599, 1519, 1456, 1331, 1258, 1216, 1203, 1171, 1151, 1098, 1069, 1024, 1001, 951, 854, 806, 764, 751, 719, 680; δ_H (400 MHz, C_6D_6) 0.83 (3H, t, *J* = 7.4, C*H*$_3$), 1.30 (2H, sxt, *J* = 7.3, C*H*$_2$CH$_3$), 1.97 (2H, qd, *J* = 7.2, 1.3, CHC*H*$_2$), 6.17 (1H, dt, *J* = 15.6, 7.0, CCH=C*H*), 6.48 (1H, dt, *J* = 15.5, 1.2, CC*H*=CH), 6.81 (1H, s, Ar-*H*), 6.87 (1H, dd, *J* = 8.6, 2.5, Ar-*H*), 7.01 (1H, d, *J* = 2.5, Ar-*H*), 7.34 (1H, d, *J* = 8.8, Ar-*H*); δ_C (100 MHz, C_6D_6) 14.4 (CH_3), 22.9 (CH_2CH_3), 35.6 ($CHCH_2$), 55.4 (OCH_3), 106.4 (Ar-*C*H), 115.0 (Ar-*C*H), 122.0 (Ar-*C*H), 123.5 (Ar-*C*H), 125.1 (C*C*H=CH), 131.7 (Ar-*C*), 133.9 (CCH=*C*H), 142.3 (Ar-*C*), 145.2 (Ar-*C*), 158.6 (Ar-*C*); *m/z* (EI) M, 232; (Found: M, 232.0915. $C_{14}H_{16}OS$ requires M, 232.0916).

(*E*)-5-Chloro-2-(pent-1-en-1-yl)benzo[*b*]thiophene S26

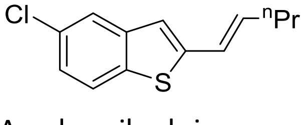

As described in general procedure C, (2-(hept-2-yn-1-yl)-4-chlorophenyl)(methyl)sulfide (49.7 mg, 0.2 mmol), iodine (55.6 mg, 0.22 mmol) in 1,2-dichloroethane (2 mL), after purification by column chromatography on silica gel eluting with *n*-hexane, gave the product

(19.0 mg, 0.08 mmol, 40 % yield) as a yellow solid (mp: 85 - 87 °C); ν_{max} (neat)/cm^{-1} 2960, 2928, 1582, 1438, 1416, 1275, 1262, 1145, 1077, 954, 906, 874, 799, 764, 749, 705; δ_H (400 MHz, C_6D_6) 0.81 (3H, t, *J* = 7.3, C*H*$_3$), 1.27 (2H, sxt, *J* = 7.3, C*H*$_2$CH$_3$), 1.93 (2H, qd, *J* = 7.2, 1.4, CHC*H*$_2$), 6.09 (1H, dt, *J* = 15.4, 7.1, CCH=C*H*), 6.34 (1H, dt, *J* = 15.6, 1.1, CC*H*=CH), 6.53 (1H, s, Ar-*H*), 6.98 (1H, dd, *J* = 8.6, 2.0, Ar-*H*), 7.10 (1H, d, *J* = 8.6, Ar-*H*), 7.42 (1H, d, *J* = 2.0, Ar-*H*); δ_C (100 MHz, C_6D_6) 14.1 (*C*H$_3$), 22.8 (*C*H$_2$CH$_3$), 35.5 (CH*C*H$_2$), 121.3 (Ar-*C*H), 123.4 (Ar-*C*H), 123.8 (Ar-*C*H), 124.6 (C*C*H=CH), 125.3 (Ar-*C*H), 131.3 (Ar-*C*), 134.9 (CCH=*C*H), 137.4 (Ar-*C*), 142.3 (Ar-*C*), 145.9 (Ar-*C*X); *m/z* (EI) [M-Cl], 202; (Found: M, 202.0802. $C_{13}H_{14}S$ requires M, 202.0811).

(E)-4,6-Dimethyl-2-(3-methylbut-1-en-1-yl)benzo[b]thiophene S27

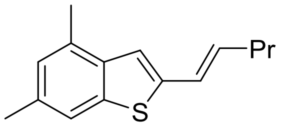

As described in general procedure C, (2-(hept-2-yn-1-yl)-3,5-dimethylphenyl)(methyl)sulfide (49.2 mg, 0.2 mmol), iodine (55.6 mg, 0.22 mmol) in 1,2-dichloroethane (20 mL), after purification by column chromatography on silica gel eluting in *n*-hexane, gave the product (42.4 mg, 0.18 mmol, 92 % yield) as a yellow oil; ν_{max} (neat)/cm^{-1} 2957, 2924, 2869, 1671, 1600, 1567, 1504, 1454, 1376, 1301, 1221, 1204, 1160, 1113, 1032, 950, 843, 757, 657; δ_H (400 MHz, CDCl$_3$) 1.00 (3 H, t, *J* = 7.4 Hz, C*H*$_3$), 1.54 (2 H, sxt, *J* = 7.4 Hz, C*H*$_2$CH$_3$), 2.23 (2 H, q, *J* = 6.9 Hz, CHC*H*$_2$), 2.43 (3 H, s, C*H*$_3$), 2.52 - 2.55 (3 H, s, C*H*$_3$), 6.14 (1 H, dt, *J* = 15.4, 7.0 Hz, CCH=C*H*), 6.63 (1 H, d, *J* = 15.5 Hz, CC*H*=CH), 6.94 (1 H, s, ArC-*H*), 7.08 (1 H, s, ArC-*H*), 7.40 (1 H, s, ArC-*H*); δ_C (100 MHz, CDCl$_3$) 13.8 (*C*H$_3$), 19.4 (*C*H$_2$CH$_3$), 21.5 (*C*H$_3$), 22.3 (*C*H$_3$), 35.0 (CH*C*H$_2$), 119.3 (Ar*C*-H), 119.5 (Ar*C*-H), 124.1 (Ar*C*H), 126.6 (C*C*H=CH), 132.0 (Ar*C*), 132.9 (CCH=*C*H), 134.3 (Ar*C*), 137.4 (Ar*C*), 138.7 (Ar*C*), 141.5 (Ar*C*); *m/z* (GCMS) M, 230.1; (Found: M, 230.1119. $C_{15}H_{18}S$ requires M, 230.1124).

General procedure E: Oxidation to bis-sulfoxide

1,5-*bis*(Hexylsulfinyl)naphthalene 8

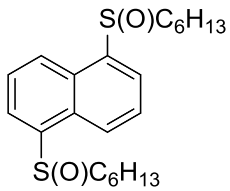

To a solution of 1,5-*bis*(hexylthio)naphthalene (3.0 g, 8.32 mmol) in CH_2Cl_2 (42.0 mL) a solution of *m*-CPBA (2.05 g, 9.15 mmol) in CH_2Cl_2 (183 mL) was added at -78 °C over 30 min. The reaction was warmed to room temperature over 1 h before adding a second portion of *m*-CPBA (2.05 g, 9.15 mmol) in CH_2Cl_2 (183 mL) over 30 min at -78 °C. After allowing the reaction mixture to reach room temperature over 1 h it was stirred for a further 1 h before quenching with aqueous $NaHCO_3$ (100 mL) and extraction with CH_2Cl_2 (2 × 75 mL). The combined organic layers were dried (Na_2SO_4) and concentrated *in vacuo*. The crude product was purified by column chromatography on silica gel (30% Et_2O in $CHCl_3$) to yield the product (2.98 g, 7.57 mmol, 91% yield) as a white solid (mp: 103 - 107 °C); ν_{max} (neat)/cm^{-1} 2949, 2921, 2856, 1498, 1466, 1403, 1390, 1338, 1275, 1261, 1193, 1155, 1113, 1074, 1036, 970, 791, 764, 750, 724; δ_H (500 MHz, $CDCl_3$) 0.79 - 0.91 (6H, m, 2 × C*H*$_3$), 1.19 - 1.31 (8H, m, 4 × C*H*$_2$), 1.32 - 1.53 (4H, m, 2 × C*H*$_2$), 1.59 - 1.74 (2H, m, 2 × $SCH_2CH_aCH_b$), 1.79 - 1.95 (2H, m, 2 × $SCH_2CH_aCH_b$), 2.75 - 2.88 (2H, m, 2 × SCH_aCH_b), 2.94 - 3.07 (2H, m, 2 × SCH_aCH_b), 7.71 - 7.82 (2H, m, 2 × Ar-*H*), 8.03 - 8.13 (2H, m, 2 × Ar-*H*), 8.16 - 8.27 (2H, m, 2 × Ar-*H*); δ_C (125 MHz, $CDCl_3$) 14.1 (2 × *C*H$_3$), 22.5 (2 × SCH_2CH_2, 2 × *C*H$_2$), 28.4 (2 × $SCH_2CH_2CH_2$), 31.5 (2 × *C*H$_2$), 56.4 (2 × S*C*H$_2$), 123.9 (2 × Ar-*C*H), 124.5 (2 × Ar-*C*H), 127.1 (2 × Ar-*C*H), 129.1 (2 × Ar-*C*), 141.8 (2 × Ar-*C*); *m/z* (ES+) M + H, 393; (Found: M + Na, 415.1741. $C_{22}H_{32}O_2S_2Na$ requires M, 415.1736).

2,6-*bis*(Hexylsulfinyl)naphthalene 7

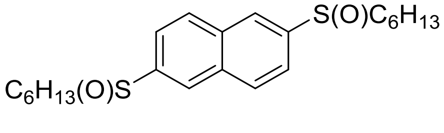

As described in general procedure E, 2,6-*bis*(hexylthio)naphthalene (6.70 g, 18.6 mmol), *m*-CPBA (9.25 g, 41.3 mmol) and CH_2Cl_2 (832 mL) after purification by column chromatography on silica gel (30% Et_2O in $CHCl_3$) gave the product (6.49 g, 16.5 mmol, 89% yield) as a white solid (mp: 155 - 157 °C); ν_{max} (neat)/cm^{-1} 2955, 2925, 2857, 1458, 1379, 1076, 1061, 1030, 970, 909, 824, 749, 728, 706, 646, 638; δ_H major diastereoisomer (400 MHz, $CDCl_3$) 0.87 (6H, t, *J* 7.1, 2 × C*H*$_3$), 1.21 - 1.34 (8H, m, 4 × C*H*$_2$), 1.36 - 1.53 (4H, m, 2 × C*H*$_2$), 1.58 - 1.71 (2H, m, 2 × $SCH_2CH_aCH_b$), 1.77 - 1.91 (2H, m, 2 × $SCH_2CH_aCH_b$), 2.80 - 2.97 (4H, m, 2 × SC*H*$_2$), 7.66 (2H, dd, *J* = 8.4, 1.4, 2 × Ar-*H*), 8.07 (2H, d, J = 8.6, 2 × Ar-*H*), 8.27 (2H, d, *J* = 1.5, 2 × Ar-*H*); δ_C (100 MHz, $CDCl_3$) 14.2 (2 × *C*H$_3$), 22.2 (2 × SCH_2CH_2), 22.6 (2 × *C*H$_2$), 28.6 (2 × $SCH_2CH_2CH_2$), 31.5 (2 × *C*H$_2$), 57.2 (2 × S*C*H$_2$), 121.6 (2 × Ar-*C*H), 124.8 (2 × Ar-*C*H), 130.0 (2 × Ar-*C*H), 134.1 (2 × Ar-*C*), 143.6 (2 × Ar-*C*); *m/z* (ES+) M + Na, 415; (Found: M + H, 393.1931. $C_{22}H_{33}O_2S_2$ requires M, 393.1916).

General Procedure F- 2D Propargylation

(2,6-Di(non-2-yn-1-yl)naphthalene-1,5-diyl)bis(hexylsulfide) 10b

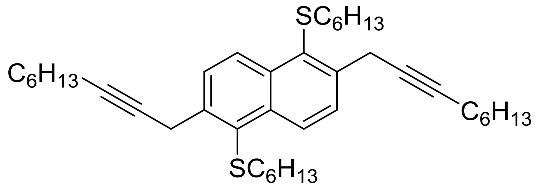

A solution containing 1,5-*bis*(hexylsulfinyl)naphthalene (1.50 g, 3.82 mmol) and trimethyl(non-2-yn-1-yl)silane (2.25 g, 11.5 mol) in MeCN (180 mL) was added to an oven dried tube flushed with N_2. Triflic anhydride (1.93 mL, 11.5 mmol) and 2,6-lutidine (1.55 mL, 13.4 mmol) were added sequentially at room temperature and the reaction mixture was then heated for 24 h at 80 °C. After cooling to room temperature, the solution was quenched with aqueous saturated $NaHCO_3$ (100 mL) and the aqueous layer was extracted with EtOAc (3 × 75 mL). The combined organic layer was washed successively with aqueous HCl 1.0 M (2 × 20 mL) and brine (100 mL), dried (Na_2SO_4) and concentrated *in vacuo*. The crude product was purified by column chromatography on silica gel eluting with 2% EtOAc in *n*-hexane to yield the product (1.76 g, 2.90 mmol, 76% yield) as a bown solid (mp: 39 - 41 °C); ν_{max} (neat)/cm^{-1} 2955, 2923, 2868, 2850, 1589, 1486, 1464, 1457, 1440, 1412, 1368,

1302, 1278, 1267, 1259, 1217, 1206, 1182, 956, 890, 813, 795, 764, 755, 726; δ_H (500 MHz, $CDCl_3$) 0.84 - 0.92 (12H, m, 4 × C*H*3), 1.18 - 1.45 (24H, m, 12 × C*H*2), 1.54 (8H, m, 4 × C*H*2), 2.23 (4H, tt, *J* = 7.1, 2.5, 2 × CC*H*2), 2.75 (4H, t, *J* = 7.6, 2 × SC*H*2), 4.14 (4H, t, *J* = 2.4, 2 × CC*H*2C), 7.85 (2H, d, *J* = 8.8, Ar-*H*), 8.74 (2H, d, *J* = 8.8, Ar-*H*); δ_C (125 MHz, $CDCl_3$) 14.0 (4 × *C*H3), 18.9 (2 × C*C*H2), 22.5 (2 × *C*H2), 22.6 (2 × *C*H2), 25.2 (2 × C*C*H2C), 28.6 (4 × *C*H2), 29.0 (2 × *C*H2), 29.8 (2 × *C*H2), 31.4 (4 × *C*H2), 36.9 (2 × S*C*H2), 78.1 (2 x *C*≡C), 82.7 (2 x C≡*C*), 127.7 (2 × Ar-*C*H), 128.0 (2 × Ar-*C*H), 131.0 (2 × Ar-*C*), 135.1 (2 × Ar-*C*), 140.7 (2 × Ar-*C*); *m/z* (ES+) M + H, 605; (Found: M, 604.4108. $C_{40}H_{60}S_2$ requires M, 604.4131).

(1,5-Di(non-2-yn-1-yl)naphthalene-2,6-diyl)bis(hexylsulfide) 9

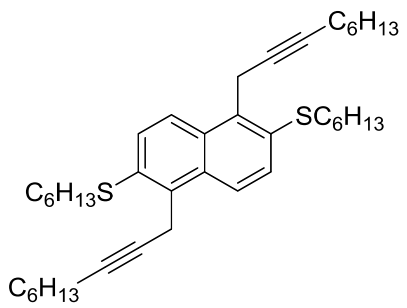

As described in general procedure F, 2,6-*bis*(hexylsulfinyl)naphthalene (29.4 mg, 0.075 mmol), trimethyl(non-2-yn-1-yl)silane (44.5 mg, 0.225 mol), triflic anhydride (38.0 µL, 0.225 mmol), 2,6-lutidine (31.0 µL, 0.263 mmol) and MeCN (7.50 mL), after purification by preparative thin-layer chromatography eluting with 2% EtOAc in *n*-hexane gave the product (34.8 mg, 0.17 mmol, 77% yield) as a yellow solid (mp: 56 - 57 °C); ν_{max} (neat)/cm^{-1} 2955, 2920, 2870, 2854, 1567, 1468, 1459, 1433, 1377, 1275, 1267, 1260, 1112, 941, 922, 798, 789, 764, 750, 722; δ_H (500 MHz, $CDCl_3$) 0.82 - 0.92 (12H, m, 4 × C*H*3), 1.18 - 1.35 (20H, m, 10 × C*H*2), 1.37 - 1.48 (8H, m, 2 × SCH2CH2C*H*2, 2 × CCH2C*H*2), 1.64 (4H, quin, *J* = 7.5, 2 × SCH2C*H*2), 2.09 (4H, tt, *J* = 7.1, 2.2, 2 × CC*H*2), 3.00 (4H, t, *J* = 7.3, 2 × SC*H*2), 4.22 (4H, t, *J* = 2.2, 2 × CC*H*2C), 7.62 (2H, d, *J* = 8.8, Ar-*H*), 8.06 (2H, d, *J* = 8.8, Ar-*H*); δ_C (125 MHz, $CDCl_3$) 14.0 (4 × *C*H3), 18.9 (2 × C*C*H2), 20.5 (2 × C*C*H2C), 22.5 (2 × *C*H2), 22.6 (2 × *C*H2), 28.5 (4 × *C*H2), 28.9 (2 × *C*H2), 29.6 (2 × SCH2*C*H2), 31.3 (2 × *C*H2), 31.4 (2 × *C*H2), 35.2 (2 × S*C*H2), 77.9 (2 x *C*≡C), 81.6 (2 x C≡*C*), 124.2 (2 × Ar-*C*H), 129.5 (2 × Ar-*C*H), 131.5 (2 × Ar-*C*), 132.6 (2 × Ar-

C), 135.4 (2 × Ar-*C*); *m/z* (ES-) M - C_6H_{13}, 519; (Found: M, 604.4112. $C_{40}H_{60}S_2$ requires M, 604.4131).

General Procedure G – Cyclisation to Alkane

2,7-Diheptylnaphtho[1,2-*b*:5,6-*b'*]dithiophene 12

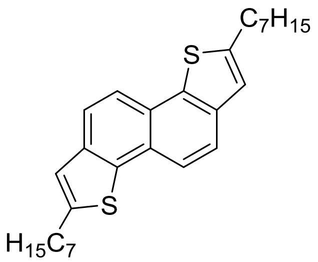

A solution containing 1,5-*bis*(hexylsulfinyl)naphthalene (29.4 mg, 0.075 mmol) and trimethyl(non-2-yn-1-yl)silane (44.2 mg, 0.225 mol) in MeCN (7.5 mL) was added An oven-dried tube flushed with N_2. Triflic anhydride (38.0 µL, 0.225 mmol) was added at room temperature and the reaction mixture was then heated for 24 h at 80 °C. After cooling to room temperature, NaI (33.8 mg, 0.225 mmol) was added and the reaction mixture was heated for a further 2 h at 80 °C, before quenching with aqueous saturated $NaHCO_3$ (5 mL) and extracting the aqueous layer with EtOAc (3 × 5 mL). The combined organic layer was washed with brine (10 mL), dried (Na_2SO_4) and concentrated *in vacuo*. The crude product was purified by thin-layer chromatography on silica gel eluting with 2% EtOAc in *n*-hexane to yield the product (18.3 mg, 0.12 mmol, 56% yield) as a white solid (mp 88 - 90 °C, toluene); ν_{max} (neat)/cm^{-1} 2950, 2921, 2849, 1541, 1466, 1460, 1363, 1306, 836, 818, 788, 688; δ_H (400 MHz, C_6D_6) 0.90 (6H, t, *J* 7.0, 2 × C*H*$_3$), 1.17 - 1.34 (16H, m, 8 × C*H*$_2$), 1.67 (4H, quin, *J* = 7.3, 2 × C*H*$_2$), 2.75 (4H, t, *J* = 7.4, 2 × C*H*$_2$), 6.91 (2H, s, 2 × Ar-*H*), 7.60 (2H, d, *J* = 8.5, 2 × Ar-*H*), 8.05 (2H, d, *J* = 8.5, 2 × Ar-*H*); δ_C (100 MHz, C_6D_6) 14.7 (2 × *C*H$_3$), 23.4 (2 × *C*H$_2$), 29.8 (4 × *C*H$_2$), 31.4 (2 × C*C*H$_2$), 32.1 (2 × CCH$_2$*C*H$_2$), 32.5 (2 × *C*H$_2$), 121.6 (2 × Ar-*C*H), 122.8 (2 × Ar-*C*H), 122.8 (2 × Ar-*C*H), 126.6 (2 × Ar-*C*), 138.3 (2 × Ar-*C*), 138.4 (2 × Ar-*C*), 145.9 (2 × Ar-*C*); *m/z* (ES+) M + H, 437; (Found: M, 436.2259. $C_{28}H_{36}S_2$ requires M, 436.2253).

2,7-Diheptylnaphtho[2,1-*b*:6,5-*b'*]dithiophene 11

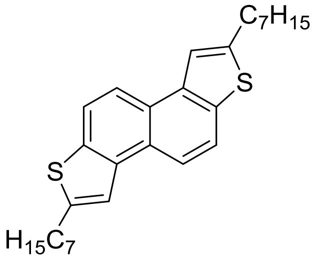

As described in general procedure G, 2,6-*bis*(hexylsulfinyl)naphthalene (29.4 mg, 0.075 mmol), trimethyl(non-2-yn-1-yl)silane (44.2 mg, 0.225 mol), triflic anhydride (38.0 µL, 0.225 mmol), NaI (33.8 mg, 0.225 mmol) and MeCN (7.5 mL), after purification thin-layer chromatography on silica gel eluting with 2% EtOAc in *n*-hexane gave the product (14.1 mg, 96.7 µmol, 43% yield) as a white solid (mp 145 - 148 °C, toluene); ν_{max} (neat)/cm^{-1} 2950, 2923, 2843, 1520, 1464, 1429, 1361, 1317, 1243, 1207, 1181, 1158, 1121, 858, 834, 743, 726; δ_H (400 MHz, C_6D_6) 0.90 (6H, t, *J* = 7.0, 2 × C*H*$_3$), 1.17 - 1.36 (16H, m, 8 × C*H*$_2$), 1.70 (4H, quin, *J* = 7.8, 2 × C*H*$_2$), 2.81 (4H, t, *J* = 7.5, 2 × C*H*$_2$), 7.52 (2H, s, 2 × Ar-*H*), 7.73 (2H, d, *J* = 8.5, 2 × Ar-*H*), 8.00 (2H, d, *J* = 8.8, 2 × Ar-*H*); δ_C (100 MHz, C_6D_6) 14.7 (2 × *C*H$_3$), 23.4 (2 × *C*H$_2$), 29.8 (2 × *C*H$_2$), 29.9 (2 × *C*H$_2$), 31.6 (2 × C*C*H$_2$), 32.2 (2 × CCH$_2$*C*H$_2$), 32.5 (2 × *C*H$_2$), 120.1 (2 × Ar-*C*H), 120.8 (2 × Ar-*C*H), 121.3 (2 × Ar-*C*H), 127.2 (2 × Ar-*C*), 136.7 (2 × Ar-*C*), 137.9 (2 × Ar-*C*), 147.3 (2 × Ar-*C*); *m/z* (ES+) M + H, 437; (Found: M, 436.2249. $C_{28}H_{36}S_2$ requires M, 436.2253).

General Procedure H- Cyclisation to Ketone

1,1'-(Naphtho[1,2-*b*:5,6-*b*']dithiophene-2,7-diyl)bis(heptan-1-one) 14

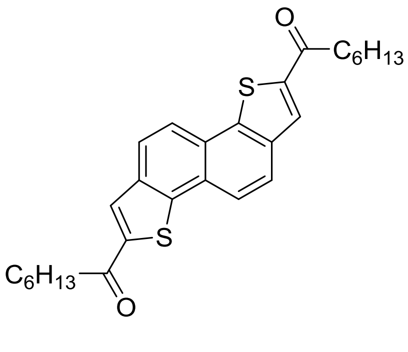

To a solution of (2,6-di(non-2-yn-1-yl)naphthalene-1,5-diyl)*bis*(hexylsulfide) (30.2 mg, 0.050 mmol) in O_2 flushed toluene (2.5 mL) was added a solution of iodine (17.8 mg, 0.070 mmol) in toluene (2.5 mL) at room temperature. The reaction mixture stirred for 18 h at 80 °C under an O_2 atmosphere before diluting with Et_2O (4 mL) and quenching with saturated aqueous $Na_2S_2O_3$ (4 mL). The aqueous layer was then extracted with Et_2O (2 × 2 mL) and the combined organic layers washed with brine (5 mL), dried (Na_2SO_4) and concentrated *in vacuo*. The crude product was purified by preparative thin-layer chromatography on silica gel eluting with CH_2Cl_2 to yield the product (13.1 mg, 28.0 µmol, 56% yield) as a yellow solid (mp 166 - 169 °C, toluene); ν_{max} (neat)/cm^{-1} 2930, 2850, 1658, 1531, 1467, 1405, 1372, 1303, 1240, 1219, 1206, 1163, 1134, 1116, 919, 889, 849, 799, 789, 710, 652; δ_H (400 MHz, C_6D_6) 0.91 (6H, t, *J* = 6.5, 2 × C*H*$_3$), 1.21 - 1.36 (12H, m, 6 × C*H*$_2$), 1.76 (4H, quin, *J* - 7.2, 2 × CH$_2$C*H*$_2$), 2.64 (4H, t, *J* = 7.3, 2 × CC*H*$_2$), 7.42 - 7.47 (4H, m, 4 × Ar-*H*), 7.87 (2H, d, *J* = 8.5, 2 × Ar-*H*); δ_C (100 MHz, C_6D_6) 14.7 (2 × *C*H$_3$), 23.3 (2 × *C*H$_2$), 25.0 (2 × CCH$_2$*C*H$_2$), 29.7 (2 × *C*H$_2$), 32.4 (2 × *C*H$_2$), 39.6 (2 × C*C*H$_2$), 122.7 (2 × Ar-*C*H), 124.7 (2 × Ar-*C*H), 127.8 (2 × Ar-*C*), 129.8 (2 × Ar-*C*H), 138.5 (2 × Ar-*C*), 142.8 (2 × Ar-*C*), 144.8 (2 × Ar-*C*), 193.7 (2 x *C*=O); *m/z* (ES+) M + H, 465; (Found: M, 464.1825. $C_{28}H_{32}O_2S_2$ requires M, 464.1838).

1,1'-(Naphtho[2,1-*b*:6,5-*b*']dithiophene-2,7-diyl)bis(heptan-1-one) 13

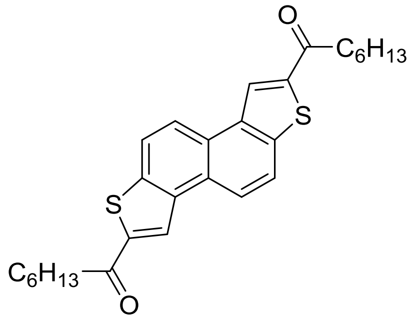

As described in general procedure H, (1,5-di(non-2-yn-1-yl)naphthalene-2,6-diyl)*bis*(hexylsulfide) (30.2 mg, 0.050 mmol), iodine (17.8 mg, 0.070 mmol) and toluene (5.0 mL) after purification by thin-layer chromatography on silica gel eluting with CH_2Cl_2 gave the product (15.1 mg, 32.5 µmol, 65% yield) as a yellow solid (mp 144 - 147 °C, toluene); ν_{max} (neat)/cm^{-1} 2957, 2934, 2912, 2892, 2852, 1664, 1559, 1480, 1468, 1453, 1406, 1373, 1340, 1296, 1233, 1190, 1182, 1167, 1143, 1112, 962, 932, 876, 843, 822, 804, 793, 761, 744, 728;

δ_H (400 MHz, C_6D_6) 0.92 (6H, t, *J* = 6.5, 2 × C*H*$_3$), 1.23 - 1.41 (12H, m, 6 × C*H*$_2$), 1.81 (4H, quin, *J* = 7.2, 2 × CCH$_2$C*H*$_2$), 2.74 (4H, t, *J* = 7.3, 2 × CC*H*$_2$), 7.51 (2H, d, *J* = 8.8, 2 × Ar-*H*), 7.81 (2H, d, *J* = 8.8, 2 × Ar-*H*), 8.17 (2H, s, 2 × Ar-*H*); δ_C (100 MHz, C_6D_6) 14.7 (2 × *C*H$_3$), 23.3 (2 × *C*H$_2$), 25.0 (2 × CCH$_2$*C*H$_2$), 29.7 (2 × *C*H$_2$), 32.4 (2 × *C*H$_2$), 39.7 (2 × C*C*H$_2$), 122.4 (2 × Ar-*C*H), 123.9 (2 × Ar-*C*H), 126.4 (2 × Ar-*C*H), 127.7 (2 × Ar-*C*), 137.1 (2 × Ar-*C*), 141.4 (2 × Ar-*C*), 145.3 (2 × Ar-*C*), 193.7 (*C*=O); *m/z* (ES+) M + H, 465; (Found: M + H, 465.1914. $C_{28}H_{33}O_2S_2$ requires M, 465.1916).

General Procedure I- Cyclisation to Alkene

2,7-Di((E)-hept-1-en-1-yl)naphtho[1,2-b:5,6-b']dithiophene 16

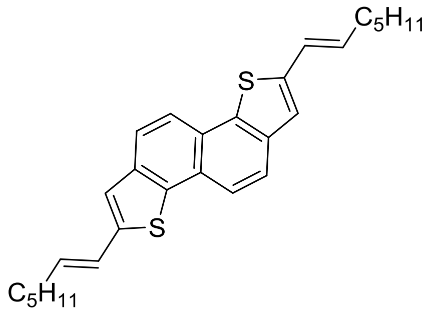

To a solution of (2,6-di(non-2-yn-1-yl)naphthalene-1,5-diyl)bis(hexylsulfide) (30.2 mg, 0.05 mmol) in Ar flushed 1,2-dichloroethane (8 mL) was added a solution of iodine (27.8 mg, 0.11 mmol) in 1,2-dichloroethane (2 mL) at room temperature with methanol (0.81 ml, 5 mmol) and stirred for 1 h at 80 °C before quenching with saturated aqueous $Na_2S_2O_3$ (10 mL). The aqueous layer was then extracted with CH_2Cl_2 (2 × 10 mL), dried ($MgSO_4$) and concentrated *in vacuo*. The crude product was purified by column chromatography on neutralised silica gel eluting with Hexane to yield the product (18.1 mg, 84% yield) as a white solid (mp 145-147 °C); ν_{max} (neat)/cm^{-1} 2960, 2930, 2483, 1332, 1263, 1169, 1145, 1121, 1074, 1055, 953, 907, 893, 812, 729, 709, 669, 651; δ_H (400 MHz, $CDCl_3$) 0.90 - 0.99 (m, 2 × CH$_3$), 1.29 - 1.45 (m, 2 × C*H*$_2$C*H*$_2$CH$_3$) 1.47 - 1.61 (m, 2 × C*H*$_2$CH$_2$CH$_2$) 2.27 (q, J = 6.89 Hz, 2 × CH=CHC*H*$_2$) 6.26 (dt, J = 15.47, 7.00 Hz, 2 × CH=C*H*) 6.65 (d, J = 15.65 Hz, 2 × C*H*=CH) 7.18 (s, 2 × Ar-*H*) 7.75 (d, J = 8.56 Hz, 2 × Ar-*H*) 7.90 (d, J = 8.68 Hz, 2 × Ar-*H*); δ_C (100 MHz, $CDCl_3$) 14.1 (2 × *C*H$_3$), 22.6 (2 × *C*H$_2$CH$_3$), 28.9 (2 × *C*H$_2$CH$_2$CH$_3$), 31.5 (2 × CHCH$_2$*C*H$_2$), 33.0 (2 × CH=CH*C*H$_2$), 121.1 (2 × Ar-*C*H), 122.2 (2 × Ar-*C*H), 122.3 (2 × Ar-*C*H), 123.6 (2 × Ar*C*H=CH),

125.8 (2 × Ar-C), 133.7 (2 × CCH=*C*H$_2$), 136.8 (2 × Ar-*C*), 137.6 (2 × Ar-*C*), 142.4 (2 × Ar-*C*); *m/z* (AP+) M + H, 433.5; (Found: M + H, 433.2023. $C_{28}H_{32}O_2S_2$ requires M, 432.1945).

2,7-Di((E)-hept-1-en-1-yl)naphtho[2,1-b:6,5-b']dithiophene 15

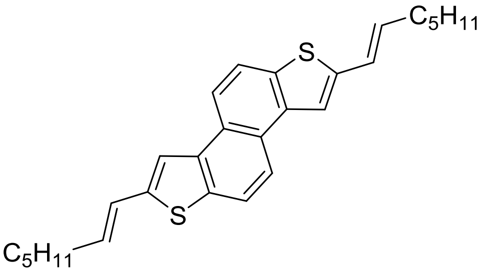

As described in general procedure I, 2,7-di((E) hept-1-en-1-yl)naphtho[2,1-b:6,5-b']dithiophene (40.9 mg, 0.088 mmol), I_2 (55.7 mg, 0.022 mmol), 1,2-dichloroethane (20.0 mL) and methanol (0.40 ml, 8.8 mmol were heated for 1 h at 80 ^{o}C. Purification by column chromatography on neutralised silica gel (1 % Et_2O in hexanes) gave the product (24.6 mg, 65% yield) as a white solid (decomp. T > 235 °C); ν_{max} (neat)/cm^{-1} 2952, 2922, 2849, 1465, 1455, 1362, 1190, 1171, 955, 876, 837, 806, 796, 725, 677; δ_H (400 MHz,CDCl$_3$) 0.91 - 0.96 (m, 2 × C*H*$_3$), 1.37 (dq, J = 7.27, 3.57 Hz, 2 × CH$_2$C*H*$_2$CH$_3$), 1.49 - 1.57 (m, 2 × CH$_2$C*H*$_2$CH$_2$), 2.27 (q, J = 6.93 Hz, 2 × CH=CHC*H*$_2$), 6.26 (dt, J = 15.44, 7.02 Hz, 2 × CH=C*H*), 6.72 (d, J = 15.53 Hz, 2 × C*H*=CH), 7.72 (s, 2 × Ar-*H*), 7.87 (d, J = 8.80 Hz, 2 × Ar-*H*), 8.12 (d, J = 8.68 Hz, 2 × Ar-*H*); δ_C (100 MHz, CDCl$_3$) 14.4 (2 × *C*H$_3$), 22.9 (2 × *C*H$_2$CH$_3$), 29.1 (2 × *C*H$_2$CH$_2$CH$_3$), 31.8 (2 × CHCH$_2$*C*H$_2$), 33.3 (2 × CH=CH*C*H$_2$), 119.7 (2 × Ar-*C*H), 120.8 (2 × Ar-*C*H), 121.0 (2 × Ar-*C*H), 124.1 (2 × C*C*H=CH$_2$), 126.6 (2 × Ar-*C*), 134.1 (2 × CCH=*C*H$_2$), 135.5 (2 × Ar-*C*), 137.3 (2 × Ar-*C*), 143.8 (2 × Ar-*C*); m/z (AP+) M + H, 432.9; (Found: M + H, 433.2009. $C_{28}H_{32}O_2S_2$ requires M, 432.1945).

Methyl(4-(methylsulfinyl)phenyl)sulfide[1] 18

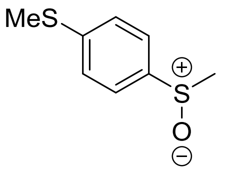

To a solution of 1,4-*bis*(methylthio)benzene (0.850 g, 5.0 mmol) in CH_2Cl_2 (60 mL) was added $NaHCO_3$ (0.460 g, 5.5 mmol) followed by a solution of m-CPBA (1.18 g, 5.25 mmol) in 15 mL CH_2Cl_2 at -78 °C. The reaction mixture was then allowed to warm to room temperature for 1 h before quenching with aqueous saturated $NaHCO_3$ (70 mL). The aqueous layer was then extracted with CH_2Cl_2 (3 x 70 mL) and the combined organic layers were washed with brine (70 mL), dried ($MgSO_4$) and concentrated *in vacuo*. The crude product was purified by column chromatography on silica gel (20% EtOAc in CH_2Cl_2) to yield the product (0.797 g, 4.3 mmol, 86%) as a white solid; δ_H (400 MHz, $CDCl_3$) 2.51 (3H, s, S*CH₃*), 2.70 (3H, s, S(O)*CH₃*), 7.35 (2H, d, *J* = 8.4, Ar-*H*), 7.55 (2H, d, *J* = 8.4, Ar-*H*); δ_C (100 MHz, $CDCl_3$) 15.3 (S*C*H₃), 44.1 (SO*C*H₃), 124.1 (2 x Ar*C*H), 126.5 (2 x Ar*C*H), 141.8 (Ar*C*), 143.3 (Ar*C*).

(2-(Hept-2-yn-1-yl)-1,4-phenylene)*bis*(methylsulfide) S28

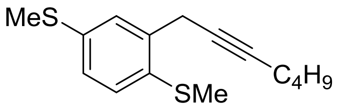

An oven-dried microwave tube was flushed with N_2 before adding a solution containing methyl(4-(methylsulfinyl)phenyl)sulfide (93.0 mg, 0.50 mmol) and hept-2-ynyltrimethylsilane (126.0 mg, 0.75 mol) in MeCN (2.5 mL). Triflic anhydride (100 µL, 0.60 mmol) and 2,6-lutidine (145 µL, 0.625 mmol) were added sequentially at room temperature and the reaction mixture was then heated in a microwave reactor for 15 min at 130 °C. After cooling to room temperature, the solution was quenched with aqueous saturated $NaHCO_3$ (10 mL) and the aqueous layers were extracted with EtOAc (3 x 10 mL). The combined organic layer was washed successively with aqueous HCl 1.0 M (10 mL) and brine (10 mL), dried ($MgSO_4$) and concentrated *in vacuo*. The crude product was purified by column

[1]K, Kobayashi; N, Furukawa, *J. Org. Chem.*, **1999**, *64* (9), pp 3190–3195

chromatography on silica gel (5% CH_2Cl_2 in hexane) to yield the product (0.103 g, 0.39 mmol, 78% yield) as a yellow oil; ν_{max} (neat)/cm^{-1} 2955, 1921, 1460, 1434, 1113, 804; δ_H (400 MHz, $CDCl_3$) 0.93 (3H, t, *J* = 7.6, *CH₃*), 1.57 - 1.40 (4H, m, 2 × *CH₂*), 2.26 (2H, tt, *J* = 6.8, 2.4, C*CH₂*), 2.44 (3H, s, S-*CH₃*), 2.49 (3H, s, S-*CH₃*), 3.61 (2H, t, *J* = 2.4, Ph-*CH₂*C), 7.17-7.12 (2H, m, ArH), 7.50 (1H, d, *J* = 0.8, ArH); δ_C (100 MHz, $CDCl_3$) 13.8 (*C*H₃), 16.4 (S*C*H₃), 16.6 (S*C*H₃), 18.7 (C*C*H₂), 22.2 (*C*H₂), 23.5 (Ph*C*H₂), 31.2 (*C*H₂), 76.6 (*C≡C*), 84.0 (*C≡C*), 125.9 (Ar*C*H), 127.0 (Ar*C*H), 127.2 (Ar*C*H), 133.6 (Ar*C*), 135.6 (Ar*C*), 136.9 (Ar*C*);); *m/z* (GCMS) 264.1; (Found: M, 265.1080, $C_{15}H_{21}S_2$ requires M, 265.1079).

5-(Methylsulfanyl)-2-pentylbenzo[b]thiophene 19

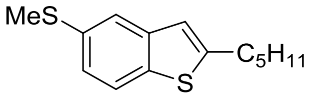

In a microwave tube was added (2-(hept-2-yn-1-yl)-1,4-phenylene)bis(methylsulfide) (396 mg, 1.5 mmol), *para*-toluene sulfonic acid (314 mg, 1.65 mmol) and EtOH (7.5 mL) and the reaction mixture was then heated in a microwave reactor for 105 min at 150 °C. After cooling to room temperature, the solution was concentrated *in vacuo*. The crude product was purified by column chromatography on silica gel (2% CH_2Cl_2 in hexane) to yield the product (0.278 g, 1.11 mmol, 74% yield) as a yellow oil; ν_{max} (neat)/cm^{-1} 2954, 2924, 1434, 1086, 796; δ_H (400 MHz, $CDCl_3$) 0.91 (3H, t, *J* = 6.8, *CH₃*), 1.39-1.36 (4H, m, 2 × *CH₂*), 1.78-1.71 (2H, m, HetAr-CH₂*CH₂*), 2.53 (3H, s, S*CH₃*), 2.88 (2H, t, *J* = 7.2, HetAr-*CH₂*), 6.93 (1H, s, ArH), 7.21 (1H, dd, *J* = 8.4, 1.6, ArH), 7.57 (1H, d, *J* = 1.6, ArH), 7.65 (1H, d, *J* = 8.4, ArH); δ_C (100 MHz, $CDCl_3$) 14.1 (*C*H₃), 17.2 (S*C*H₃), 22.6 (*C*H₂), 30.9 (*C*H₂), 31.4 (*C*H₂), 120.0 (Ar*C*H), 121.4 (Ar*C*H), 122.5 (Ar*C*H), 123.7 (Ar*C*H), 133.9 (Ar*C*), 136.8 (Ar*C*), 141.1 (Ar*C*), 148.2 (Ar*C*); m/z (ES+) (M), 250.2;); *m/z* (GCMS) 250.1; (Found: M, 250.0845, $C_{14}H_{18}S_2$ requires M, 250.0844).

5-(Methylsulfinyl)-2-pentylbenzo[b]thiophene 20

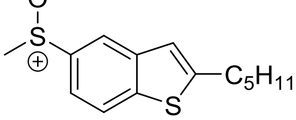

To a solution of 5-(methylthio)-2-pentylbenzo[b]thiophene (0.40 g, 1.6 mmol) in CH_2Cl_2 (20 mL) was added $NaHCO_3$ (0.147 g, 1.75 mmol) followed by a solution of *m*-CPBA (0.37 g, 1.68 mmol) in 5 mL CH_2Cl_2 at -78 °C. The reaction mixture was then allowed to warm to room temperature for 1 h before quenching with aqueous saturated $NaHCO_3$ (20 mL). The aqueous layer was then extracted with CH_2Cl_2 (3 x 20 mL) and the combined organic layers were washed with brine (20 mL), dried ($MgSO_4$) and concentrated *in vacuo*. The crude product was purified by column chromatography on silica gel (10% EtOAc in CH_2Cl_2) to yield the product (0.383 g, 1.44 mmol, 90% yield) as a pale yellow solid (mp 67-69 ^{o}C); ν_{max} (neat)/cm^{-1} 2955, 2925, 1434, 1039, 810, 736; δ_H (400 MHz, $CDCl_3$) 0.91 (3H, t, *J* = 7.2, *CH$_3$*), 1.40- 1.36 (4H, m, 2 × *CH$_2$*), 1.80-1.72 (2H, m, HetAr-CH_2*CH$_2$*), 2.76 (3H, s, S*CH$_3$*), 2.92 (2H, t, *J* = 7.6, HetAr-*CH$_2$*), 7.07 (1H, s, ArH), 7.44 (1H, dd, *J* 8.4, 1.2, ArH), 7.88 (1H, d, *J* 8.4, ArH), 8.00 (1H, d, *J* 1.2, ArH); δ_C (100 MHz, $CDCl_3$) 14.1 (*C*H$_3$), 22.5 (*C*H$_2$), 30.9 (*C*H$_2$), 31.0 (*C*H$_2$), 31.4 (*C*H$_2$), 44.5 (S(O)*C*H$_3$), 117.9 (Ar*C*H), 118.2 (Ar*C*H), 120.6 (Ar*C*H), 123.2 (Ar*C*H), 140.6 (Ar*C*), 141.6 (Ar*C*), 142.1 (Ar*C*), 149.8 (Ar*C*); m/z (ES+) (M + H), 267.2; (Found: M, 267.0877. $C_{14}H_{19}S_2O$ requires M, 267.0877).

4-(3-Cyclohexylprop-2-yn-1-yl)-5-(methylthio)-2-pentylbenzo[b]thiophene S29

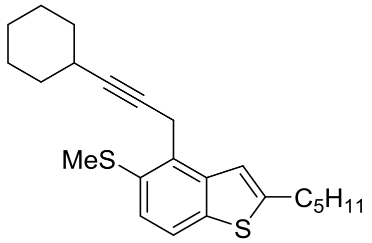

An oven-dried microwave tube was flushed with N_2, before adding a solution containing 5-(methylsulfinyl)-2-pentylbenzo[b]thiophene (133.0 mg, 0.50 mmol) and (3-cyclohexylprop-2-yn-1-yl)trimethylsilane (146.0 mg, 0.75 mol) in MeCN (2.5 mL). Triflic anhydride (100 µL, 0.60 mmol) and 2,6-lutidine (145 µL, 0.625 mmol) were added sequentially at room temperature and the reaction mixture was then heated in a microwave reactor for 15 min at 130 °C. After cooling to room temperature, the solution was quenched with aqueous saturated $NaHCO_3$ (10 mL) and the aqueous layer was extracted with EtOAc (3 x 10 mL). The combined organic layer was washed successively with aqueous HCl 1.0 M (10 mL) and brine (10 mL), dried ($MgSO_4$) and concentrated *in vacuo*. The crude product was purified by column chromatography on silica gel (5% CH_2Cl_2 in hexane) to yield the product (0.140 g,

0.38 mmol, 76% yield) as a white solid (mp 50-52 ^{O}C); vmax (neat)/cm^{-1} 2922, 2854, 1448, 1152, 945, 819; δ_H (400 MHz, $CDCl_3$) 0.91 (3H, t, *J* = 7.2, *CH_3*), 1.28-1.19 (2H, m, *CH_2*), 1.45-1.33 (8H, m, *CH_2*), 1.66-1.62 (2H, m, *CH_2*), 1.78-1.70 (4H, m, *CH_2*), 2.34-2.26 (1H, m, *CH*), 2.48 (3H, s, S*CH_3*), 2.91 (2H, t, *J* = 7.2, HetAr-*CH_2*), 4.04 (2H, d, *J* = 2.4, Ph-*CH_2*C), 7.24 (1H, s, ArH), 7.33 (1H, d, *J* = 8.4, ArH), 7.60 (1H, d, *J* = 8.4, ArH); δ_C (100 MHz, $CDCl_3$) 14.1 (*C*H_3), 19.2 (S*C*H_3), 21.4 (Ph*C*H_2), 22.6 (*C*H_2), 25.0 (*C*H_2), 26.1 (*C*H_2), 29.3 (*C*H), 31.0 (Ph*C*H_2), 31.1 (*C*H_2), 31.4 (*C*H_2), 33.0 (*C*H_2), 77.8 (*C≡C*), 85.6 (*C≡C*), 119.5 (Ar*C*H), 121.2 (Ar*C*H), 126.2 (Ar*C*H), 131.7 (Ar*C*), 132.7 (Ar*C*), 138.5 (Ar*C*), 140.1 (Ar*C*), 147.7 (Ar*C*); *m/z*

2-(Cyclohexylmethyl)-7-pentylbenzo[1,2-b:4,3-b']dithiophene 21

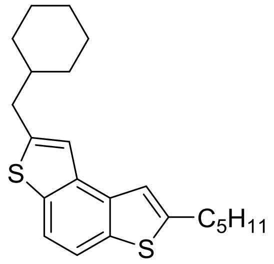

In a microwave tube was added 4-(3-cyclohexylprop-2-yn-1-yl)-5-(methylthio)-2-pentylbenzo[b]thiophene (148.0 mg, 0.4 mmol), *para*-toluene sulfonic acid (84.0 mg, 0.44 mmol) and EtOH (2.5 mL) and the reaction mixture was then heated in a microwave reactor for 105 min at 150 °C. After cooling to room temperature, the solution was concentrated *in vacuo*. The crude product was purified by column chromatography on silica gel (1% CH_2Cl_2 in hexane) to yield the product (0.124 g, 0.34 mmol, 87% yield) as a white solid (mp 67-68 ^{O}C); vmax (neat)/cm^{-1} 2921, 2849, 1448, 1124, 830, 802; δ_H (400 MHz, $CDCl_3$) 0.92 (3H, t, *J* = 6.8, *CH_3*), 1.05-0.97 (2H, m, *CH_2*), 1.30-1.15 (3H, m, *CH*+*CH_2*), 1.40-1.35 (4H, m, *CH_2*), 1.73-1.64 (4H, m, *CH_2*), 1.82-1.77 (4H, m, *CH_2*), 2.83 (2H, d, *J* = 7.2, HetAr-*CH_2*), 2.97 (2H, t, *J* = 7.6, HetAr-*CH_2*), 7.28 (1H, s, ArH), 7.31 (1H, s, ArH), 7.62 (2H, s, ArH); δ_C (100 MHz, $CDCl_3$) 14.2 (*C*H_3), 22.6 (*C*H_2), 26.3 (2 x *C*H_2), 26.6 (*C*H_2), 31.0 (HetAr*C*H_2), 31.2 (*C*H_2), 31.4 (*C*H), 33.2 (2 x *C*H_2), 38.9 (HetAr*C*H_2), 40.1 (*C*H_2), 117.7 (Ar*C*H), 117.7 (Ar*C*H), 118.8 (Ar*C*H), 119.7 (Ar*C*H), 134.5 (Ar*C*), 134.5 (Ar*C*), 135.6 (Ar*C*), 135.8 (Ar*C*), 145.4 (Ar*C*), 147.0 (Ar*C*); m/z (ES+) (M + H), 357; (Found: M, 357.1699. $C_{22}H_{29}S_2$ requires M, 357.1711).

General Procedure J- Alkene Dimerisation

6,12-Dibutylbenzo[1,2-*b*:4,5-*b'*]bis[*b*]benzothiophene 22

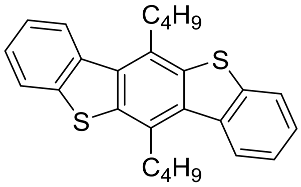

Under an Ar atmosphere, a solution of (*E*)-2-(pent-1-en-1-yl)benzo[*b*]thiophene) (40.4 mg, 0.2 mmol) in Ar sparged 1,2-dichloroethane (2 ml) was added to a solution of iodine (506 mg, 2 mmol) in Ar sparged 1,2-dichloroethane (18 mL). The reaction mixture was stirred for 18 h at 80 °C before quenching with saturated aqueous $Na_2S_2O_3$ (5 mL). The aqueous layer was then extracted with EtOAc (3 × 5 mL) and the combined organic layers washed with brine (5 mL), dried (Na_2SO_4) and concentrated *in vacuo*. The crude product was washed with cold ethanol (3 ml) to yield the product (34.8 mg, 86.0 µmol , 86 % yield) as a white solid (mp: 151 - 155 °C); ν_{max} (neat)/cm^{-1} 2953, 2923, 2850, 1468, 1424, 1364, 1275, 1267, 1164, 1106, 1074, 1043, 927, 764, 751, 724, 670; δ_H (400 MHz, $CDCl_3$) 1.09 (6 H, t, *J* 7.3, 2 × C*H*$_3$), 1.69 (4 H, sxt, *J* = 7.1, 2 × C*H*$_2$CH$_3$), 1.93 (4 H, quin, *J* = 7.5, 2 × CCH$_2$C*H*$_2$), 3.46 - 3.57 (4 H, m, 2 × CC*H*$_2$), 7.44 - 7.57 (4 H, m, 4 × ArC-*H*), 7.88 - 7.97 (2 H, m, 2 × ArC-*H*), 8.25 - 8.37 (2 H, m, 2 × ArC-*H*); δ_C (100 MHz, $CDCl_3$) 14.2 (2 × *C*H$_3$), 23.5 (2 × *C*H$_2$CH$_3$), 30.6 (2 × CCH$_2$*C*H$_2$), 33.9 (2 × C*C*H$_2$), 123.1 (2 × Ar-*C*H), 124.7 (2 × Ar-*C*H), 125.1 (2 × Ar-*C*H), 126.3 (2 × Ar-*C*H), 131.3 (4 × Ar-*C*), 136.2 (2 × Ar-*C*), 139.1 (2 × Ar-*C*), 140.2 (2 × Ar-*C*); *m/z* (EI) M, 402; (Found: M, 402.1483. $C_{26}H_{26}S_2$ requires M, 402.1470).

6,12-Dibutylbenzo[1,2-*b*:4,5-*b'*]bis[*b*]5-methylbenzothiophene 23

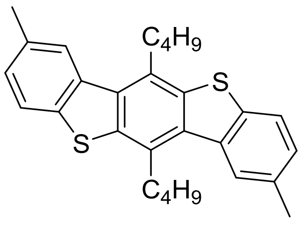

As for general procedure J, (*E*)-5-methyl-2-(pent-1-en-1-yl)benzo[*b*]thiophene) (43.2 mg, 0.2 mmol) in 1,2-dichloroethane (2 ml) was added to a solution of iodine (506 mg, 2 mmol) in 1,2-dichloroethane (18 ml). The reaction mixture was stirred for 18 h at 80 °C before quenching. The crude product was washed with cold ethanol (3 ml) to yield the product (27.1 mg, 63.0 µmol, 63 % yield) as a white solid (mp: 260-262 °C); ν_{max} (neat)/cm^{-1} 2961,

1568, 1412, 1258, 1017, 861, 793, 687, 626, 613, 567; δ_H (400 MHz, $CDCl_3$) 1.11 (6 H, t, *J* = 7.3 Hz, 2 x CH_2CH_3), 1.70 (4 H, sxt, *J* = 7.3 Hz, 2 x CH_2CH_3), 1.87 - 1.97 (4 H, m, 2 x CCH_2CH_2), 2.58 (6 H, s, 2 x CH_3), 3.45 - 3.54 (4 H, m, 2 x CCH_2CH_2), 7.32 (2 H, dd, *J* = 8.0, 0.6 Hz, 2 x ArC-*H*), 7.80 (2 H, d, *J* = 8.0 Hz, ArC-*H*), 8.13 (2 H, s, ArC-*H*); δ_C (100 MHz, $CDCl_3$) 14.0 (2 x CH_3), 22.0 (2 x CH_2CH_3), 23.2 (2 x ArC-CH_3), 30.2 (2 x CCH_2CH_2), 33.5 (2 x CCH_2CH_2), 122.4 (2 X Ar*C*-H), 125.3 (2 X Ar*C*-H), 127.4 (2 X Ar*C*-H), 130.9 (2 X Ar*C*), 131.0 (2 X Ar*C*), 133.9 (2 X Ar*C*), 136.2 (2 X Ar*C*), 136.8 (2 X Ar*C*), 139.2 (2 X Ar*C*); *m/z* (APCI) M+H, 431.6; (Found: M, 431.1858. $C_{28}H_{30}S_2$ requires M,).

6,12-Dibutylbenzo[1,2-*b*:4,5-*b'*]bis[*b*]5-methoxybenzothiophene 24

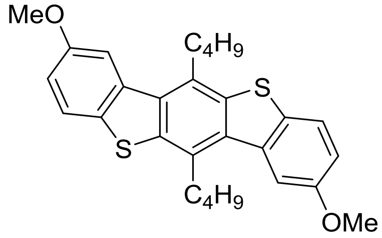

As in general procedure J, (*E*)-5-methoxy-2-(pent-1-en-1-yl)benzo[*b*]thiophene) (49.6 mg, 0.2 mmol) in 1,2-dichloroethane (2 ml) was added to a solution of iodine (506 mg, 2 mmol) in 1,2-dichloroethane (18 ml). The reaction mixture was stirred for 18 h at 80 °C before quenching. The crude product was washed with cold ethanol (3 ml) to yield the product (27.6 mg, 55.0 µmol, 55 % yield) as a white solid (mp: 175-177 °C); ν_{max} (neat)/cm^{-1} 2959, 2930, 2872, 1874, 1596, 1564, 1470, 1428, 1308, 1213, 1184, 1020, 894, 863, 794, 663; δ_H (500 MHz, $CDCl_3$) 1.09 (6 H, t, *J* = 7.3 Hz, 2 x CH_2CH_3), 1.70 (4 H, sxt, *J* = 7.4 Hz, 2 x CH_2CH_3), 1.90 - 1.98 (4 H, m, 2 x CCH_2CH_2), 3.46 - 3.50 (4 H, m, 2 x CCH_2CH_2), 3.97 (6 H, s, 2 x OCH_3), 7.14 (2 H, dd, *J* = 8.7, 2.3 Hz, 2 x ArC-*H*), 7.79 (2 H, d, *J* = 8.7 Hz, 2 x ArC-*H*), 7.84 (2 H, d, *J* = 2.3 Hz, 2 x ArC-*H*); δ_C (100 MHz, $CDCl_3$) 14.1 (2 x CH_3), 23.4 (2 x CH_2CH_3), 30.5 (2 x CCH_2CH_2), 33.7 (2 x CCH_2CH_2), 55.7 (2 x OCH_3), 109.2 (2 x Ar*C*-H), 114.6 (2 x Ar*C*-H), 123.2 (2 x Ar*C*-H), 131.1 (2 x Ar*C*), 131.1 (2 x Ar*C*), 131.8 (2 x Ar*C*), 136.9 (2 x Ar*C*), 139.9 (2 x Ar*C*), 157.5 (2 x Ar*C*-OCH_3); *m/z* (APCI) M+H, 463.1; (Found: M, 463.1751. $C_{28}H_{30}O_2S_2$ requires M, 463.1765).

6,12-Dibutylbenzo[1,2-*b*:4,5-*b'*]bis[*b*]5-fluorobenzothiophene 25

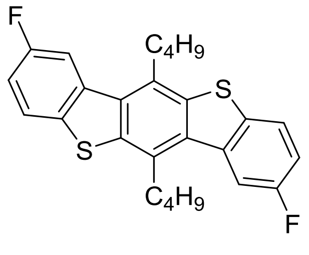

As for general procedure J, (*E*)-5-fluoro-2-(pent-1-en-1-yl)benzo[*b*]thiophene) (44.0 mg, 0.2 mmol) in 1,2-dichloroethane (2 ml) was added to a solution of iodine (506 mg, 2 mmol) in 1,2-dichloroethane (18 ml). The reaction mixture was stirred for 18 h at 80 °C before quenching. The crude product was washed with cold ethanol (3 ml) to yield the product (25.4 mg, 58.0 µmol, 58 % yield) as a white solid (mp: 209-211 °C); ν_{max} (neat)/cm^{-1} 2960, 2870, 1770, 1601, 1569, 1472, 1414, 1356, 1293, 1258, 1183, 1096, 1020, 939, 850, 793, 656, 613; δ_H (400 MHz, $CDCl_3$) 1.09 (6 H, t, *J* = 7.34 Hz, 2 x CH_2CH_3), 1.69 (4 H, sxt, *J* = 7.3 Hz, 2 x CH_2CH_3), 1.84 - 1.94 (4 H, m, 2 x CCH_2CH_2), 3.40 - 3.46 (4 H, m, 2 x CCH_2CH_2), 7.26 (2 H, td, *J* = 8.5, 2.3 Hz, 2 x ArC-*H*), 7.84 (2 H, dd, *J* = 8.7, 5.2 Hz, , ArC-*H*), 7.97 (2 H, dd, *J* = 11.0, 2.3 Hz, ArC-*H*); δ_C (100 MHz, $CDCl_3$) 13.9 (2 x CH_3), 23.1 (2 x CH_2CH_3), 30.3 (2 x CCH_2CH_2), 33.3 (2 x CCH_2CH_2), 111.0 (d, *J* = 28.2 Hz, 2 x Ar*C*-H), 114.1 (d, *J* = 24.5 Hz, 2 x Ar*C*-H), 123.6 (d, *J* = 9.1 Hz, 2 x Ar*C*-H), 131.0 (d, *J* = 3.6 Hz, 2 x Ar*C*) 131.4 (2 x Ar*C*), 135.1 (2 x Ar*C*), 136.9 (d, *J* = 9.1 Hz, 2 x Ar*C*), 140.1 (2 x Ar*C*), 158.9 (d, *J* = 240.7 Hz, 2 x Ar*C*-F); *m/z* (APCI) M+Na+H,463.3; (Found: M, 439.1356. $C_{26}H_{24}F_2S_2$ requires M, 439.1366).

6,12-Dibutylbenzo[1,2-*b*:4,5-*b*']bis[*b*]6-fluorobenzothiophene 26

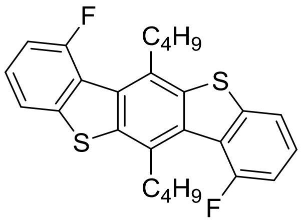

As in general procedure J, (*E*)-4-fluoro-2-(pent-1-en-1-yl)benzo[*b*]thiophene) (32.8 mg, 0.14 mmol) in 1,2-dichloroethane (2 ml) was added to a solution of iodine (374 mg, 1.48 mmol) in 1,2-dichloroethane (12 ml). The reaction mixture was stirred for 18 h at 80 °C before quenching. The crude product was washed with cold ethanol (3 ml) to yield the product (14.7 mg, 33.0 µmol, 43 % yield) as a white solid (mp: 213-215 °C); ν_{max} (neat)/cm^{-1} 2955, 2934, 2873, 2858, 1597, 1596, 1485, 1457, 1408, 1365, 1315, 1274, 1252, 1195, 1104, 1034,

925, 900, 842, 800, 767,735, 720; δ_H (500 MHz, $CDCl_3$) 1.07 (6 H, t, *J* = 7.4 Hz, 2 x CH_2CH_3), 1.67 (4 H, sxt, *J* = 7.4 Hz, 2 x CH_2CH_3), 1.82 - 1.93 (4 H, m, 2 x CCH_2CH_2), 3.38 - 3.48 (4 H, m, 2 x CCH_2CH_2), 7.23 (2 H, td, *J* = 8.8, 2.4 Hz, 2 x ArC-*H*), 7.59 (2 H, dd, *J* = 8.4, 2.4 Hz, 2 x ArC-*H*), 8.21 (2 H, dd, *J* = 9.0, 5.0 Hz, 2 x ArC-*H*); δ_C (100 MHz, $CDCl_3$) 13.9 (2 x CH_3), 23.1 (2 x CH_2CH_3), 30.2 (2 x CCH_2CH_2), 33.3 (2 x CCH_2CH_2), 109.1 (d, *J* = 22.7 Hz, 2 x Ar*C*-H), 112.7 (d, *J* = 22.7 Hz, 2 x Ar*C*-H), 125.8 (d, *J* = 9.1 Hz, 2 x Ar*C*-H), 130.1 (2 x Ar*C*), 130.4 (2 x Ar*C*), 132.3 (2 x Ar*C*), 138.9 (2 x Ar*C*), 141.4 (d, *J* = 10.0 Hz, 2 x Ar*C*), 161.1 (d, *J* = 248.0 Hz, 2 x Ar*C*-F); *m/z* (APCI) M 438.9, ; (Found: M, 439.1351. $C_{26}H_{25}S_2F_2$ requires M, 439.1366).

6,12-Dibutylbenzo[1,2-*b*:4,5-*b'*]bis[*b*]5-chlorobenzothiophene 27

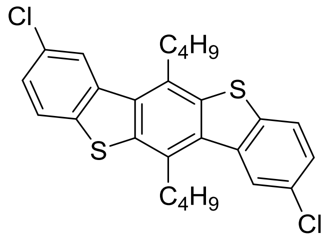

As in general procedure J, (*E*)-5-chloro-2-(pent-1-en-1-yl)benzo[*b*]thiophene) (33.7 mg, 0.14 mmol) in 1,2-dichloroethane (2 ml) was added to a solution of iodine (359 mg, 1.42 mmol) in 1,2-dichloroethane (12 ml). The reaction mixture was stirred for 18 h at 80 °C before quenching. The crude product was washed with cold ethanol (2 ml) to yield the product (19.0 mg, 46.0 µmol, 64 % yield) as a white solid (mp: 196-198 °C); ν_{max} (neat)/cm^{-1} 2957, 2928, 2870, 2851, 1873, 1583, 1548, 1474, 1431, 1409, 1375, 1320, 1289, 1143, 1102, 1040, 865, 855, 812, 800, 783, 732; δ_H (500 MHz, $CDCl_3$) 1.11 (6 H, t, *J* = 7.4 Hz, 2 x CH_2CH_3), 1.70 (4 H, sxt, *J* = 7.3 Hz, 2 x CH_2CH_3), 1.85 - 1.96 (4 H, m, 2 x CCH_2CH_2), 3.39 - 3.51 (4 H, m, 2 x CCH_2CH_2), 7.47 (2 H, dd, *J* = 8.4, 1.9 Hz, 2 x ArC-*H*), 7.83 (2 H, d, *J* = 8.3 Hz, 2 x ArC-*H*), 8.27 (2 H, d, *J* = 1.8 Hz, 2 x ArC-*H*); δ_C (100 MHz, $CDCl_3$) 13.9 (2 x CH_3), 23.1 (2 x CH_2CH_3), 30.1 (2 x CCH_2CH_2), 33.4 (2 x CCH_2CH_2), 123.6 (2 x Ar*C*-H), 124.8 (2 x Ar*C*-H), 126.4 (2 x Ar*C*-H), 130.6 (2 x Ar*C*), 130.6 (2 x Ar*C*), 131.4 (2 x Ar*C*), 137.0 (2 x Ar*C*), 138.1 (2 x Ar*C*), 139.7 (2 x Ar*C*-Cl); *m/z* (APCI) M+H, 471.5; (Found: M, 471.0789. $C_{26}H_{25}S_2Cl_2$ requires M, 471.0775).

6,12-Dihexylbenzo[1,2-*b*:4,5-*b*']bis[*b*]benzothiophene 28

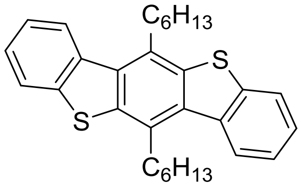

As in general procedure J, (*E*)-2-(hex-1-en-1-yl)benzo[*b*]thiophene) (41.2 mg, 0.17 mmol) in 1,2-dichloroethane (2 ml) was added to a solution of iodine (450 mg, 1.78 mmol) in 1,2-dichloroethane (15 ml). The reaction mixture was stirred for 18 h at 80 °C before quenching. The crude product was washed with cold ethanol (2 ml) to yield the product (30.7 mg, 69.0 µmol, 81 % yield) as a white solid (mp: 155-157 °C); ν_{max} (neat)/cm^{-1} 2948, 2923, 2854, 1725, 1467, 1424, 1365, 1260, 1163, 1103, 1072, 1049, 927, 843, 801, 756, 723, 700, 660; δ_H (500 MHz, $CDCl_3$) 0.96 (6 H, t, *J* = 7.1 Hz, 2 x CH_2CH_3), 1.36 - 1.49 (8 H, m, 4 x CH_2), 1.68 (4 H, quin, *J* = 7.4 Hz, 2 x CH_2), 1.89 - 1.97 (4 H, m, 2 x CCH_2CH_2), 3.47 - 3.52 (4 H, m, 2 x CCH_2CH_2), 7.50 (4 H, quin, *J* = 6.5 Hz, 4 x ArC-*H*), 7.93 (2 H, d, *J* = 8.7 Hz, 2 x ArC-*H*), 8.30 (2 H, d, *J* = 7.5 Hz, 2 x ArC-*H*); δ_C (100 MHz, $CDCl_3$) 14.1 (2 x CH_3), 22.6 (2 x CH_2CH_3), 28.1 (2 x CH_2), 29.7 (CH_2), 31.6 (2 x CCH_2CH_2), 33.9 (2 x CCH_2CH_2), 122.8 (2 x Ar*C*-H), 124.4 (2 x Ar*C*-H), 124.8 (2 x Ar*C*-H), 126.0 (2 x Ar*C*-H), 131.0 (2 x Ar*C*), 131.0 (2 x Ar*C*), 135.9 (2 x Ar*C*), 138.8 (2 x Ar*C*), 139.9 (2 x Ar*C*); *m/z* (APCI) M+H, 459.6; (Found: M, 459.2200. $C_{30}H_{35}S_2$ requires M, 459.2180).

^{1}H and ^{13}C NMR Spectra

1-(5-Methylbenzo[*b*]thiophen-2-yl)pentan-1-one S1

^{1}H NMR (400 MHz, $CDCl_3$)

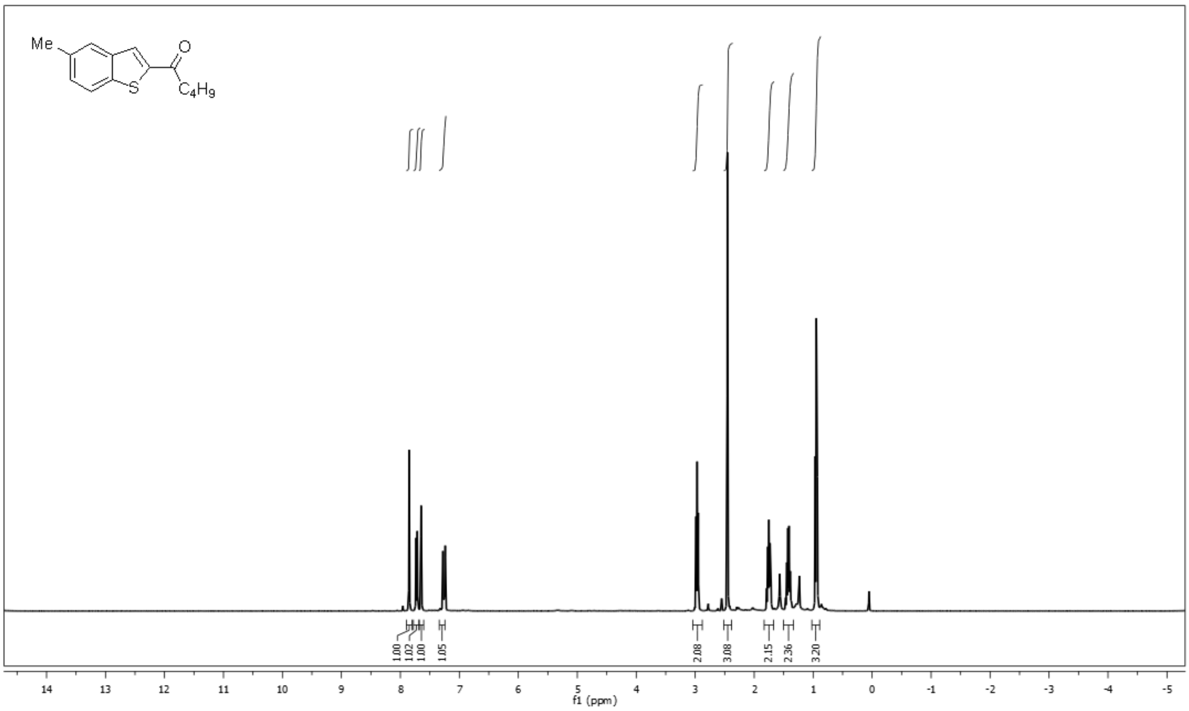

^{13}C NMR (100 MHz, $CDCl_3$)

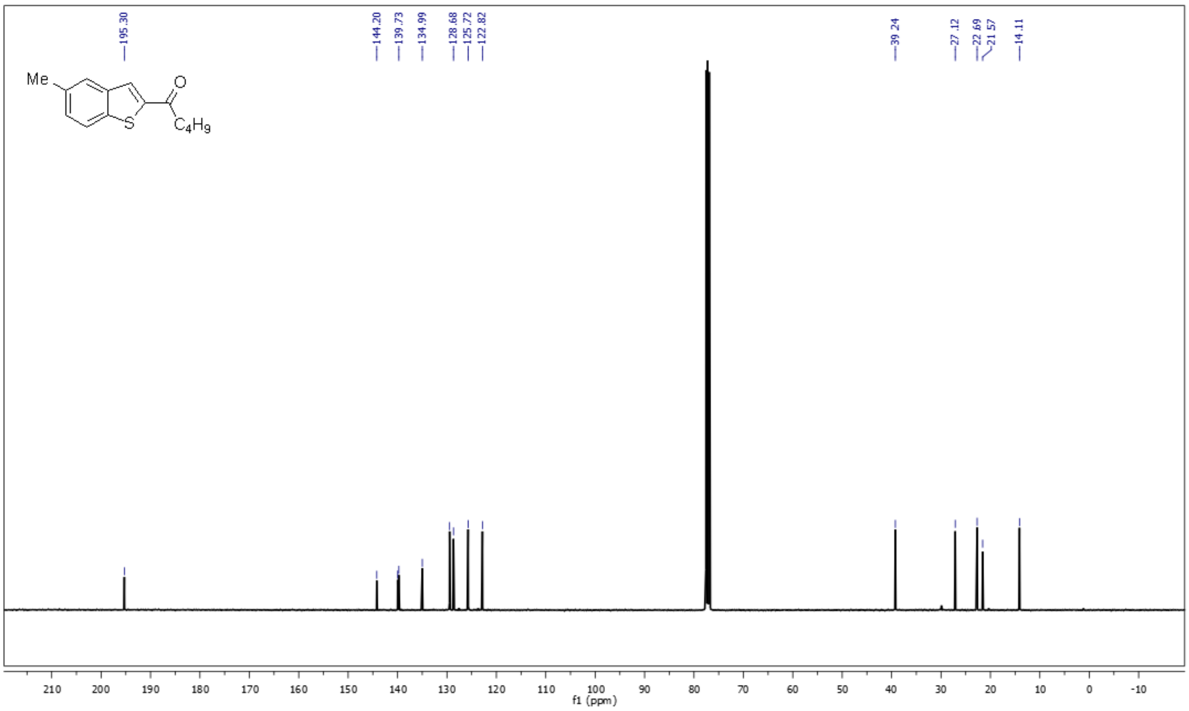

1-(7-Methylbenzo[*b*]thiophen-2-yl)pentan-1-one S2

^{1}H NMR (400 MHz, CDCl$_3$)

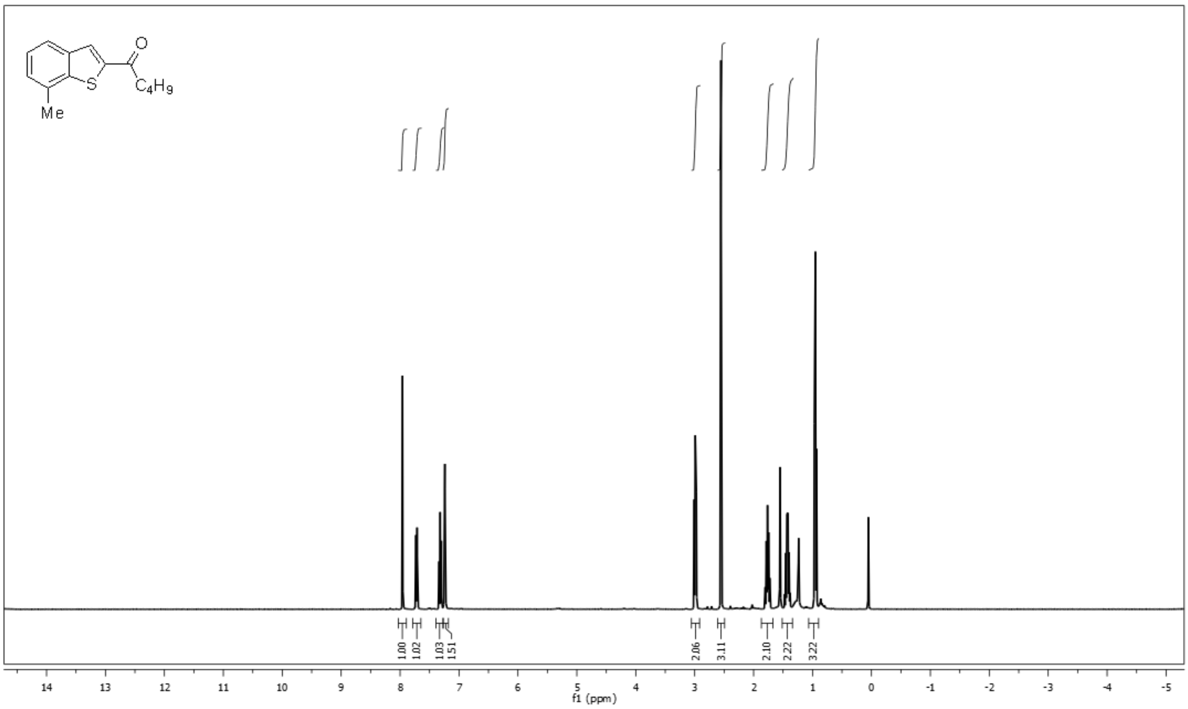

^{13}C NMR (100 MHz, CDCl$_3$)

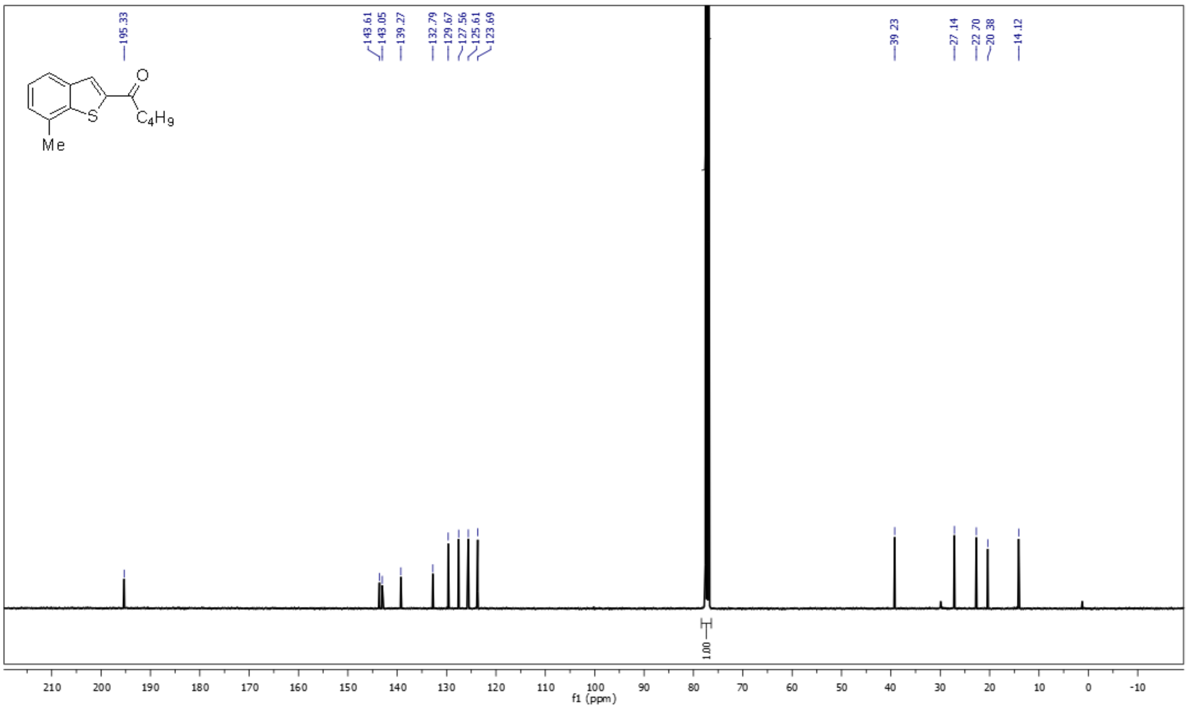

1-(5-Fluorobenzo[*b*]thiophen-2-yl)pentan-1-one S3

^{1}H NMR (400 MHz, $CDCl_3$)

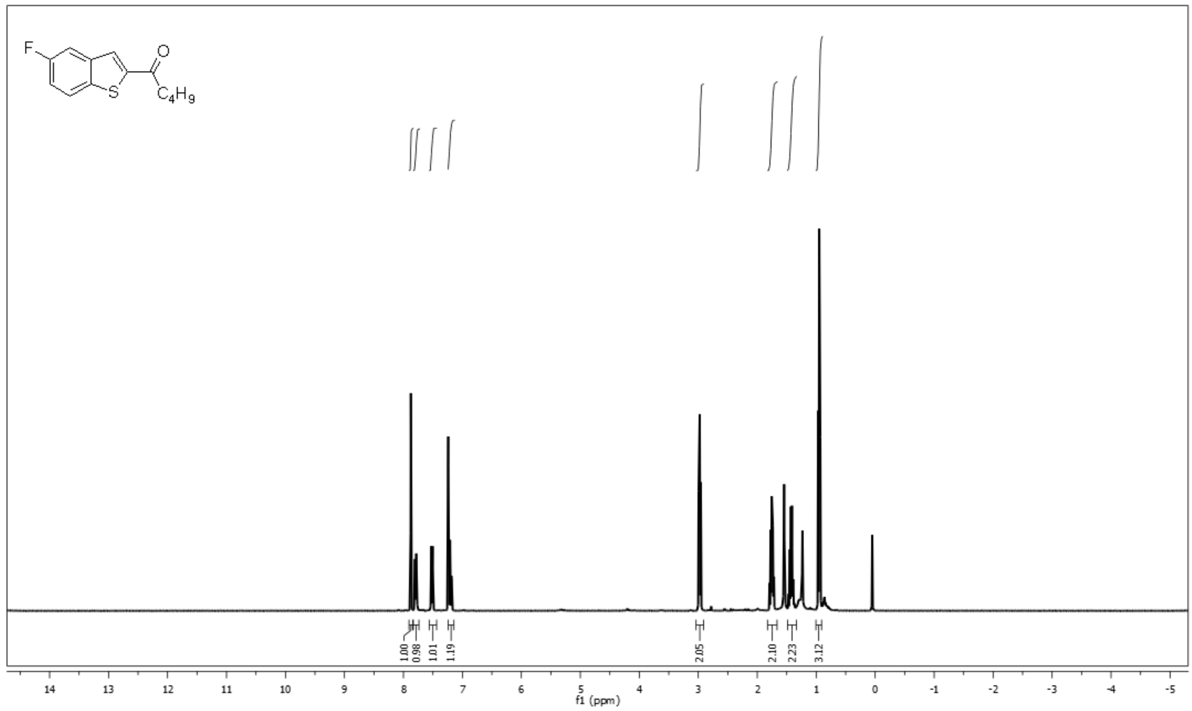

^{13}C NMR (100 MHz, $CDCl_3$)

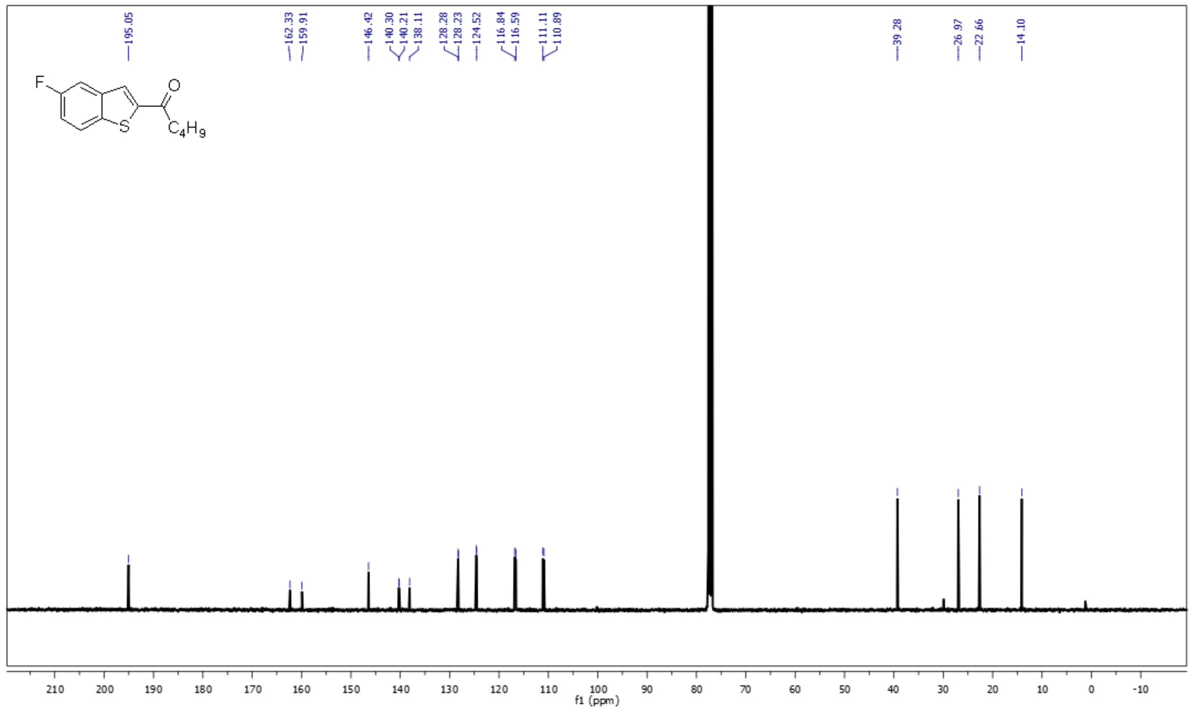

1-(5-Chlorobenzo[*b*]thiophen-2-yl)pentan-1-one S4

^{1}H NMR (400 MHz, $CDCl_3$)

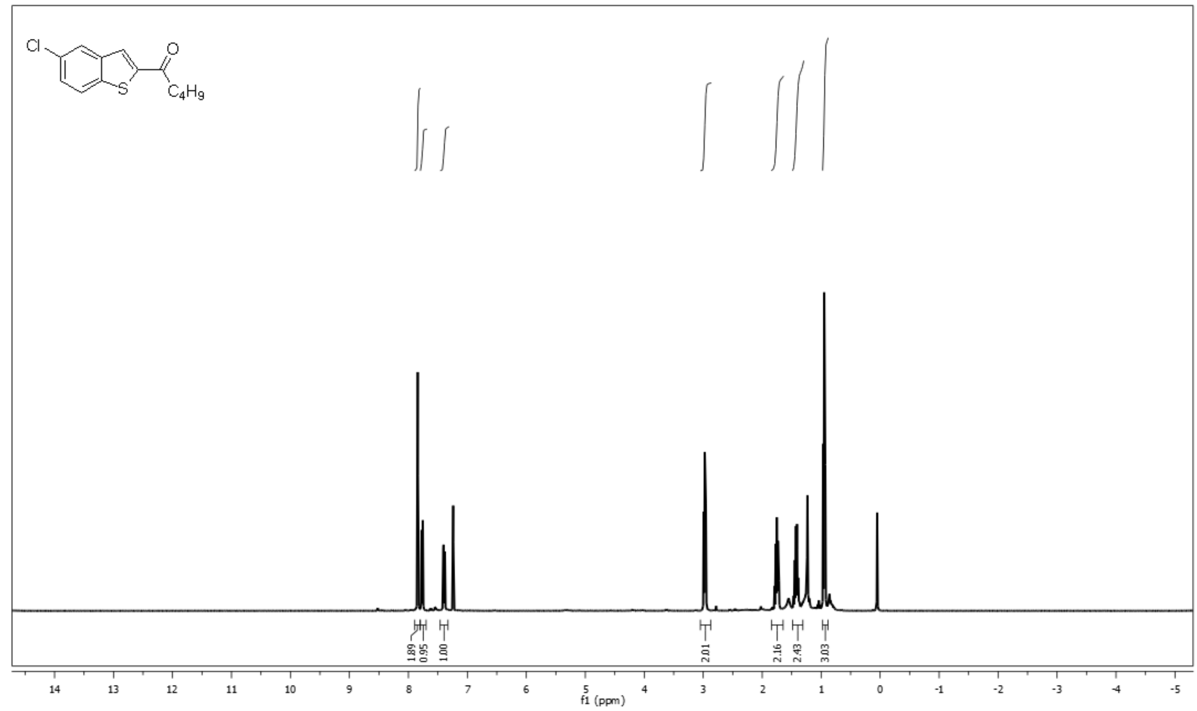

^{13}C NMR (100 MHz, $CDCl_3$)

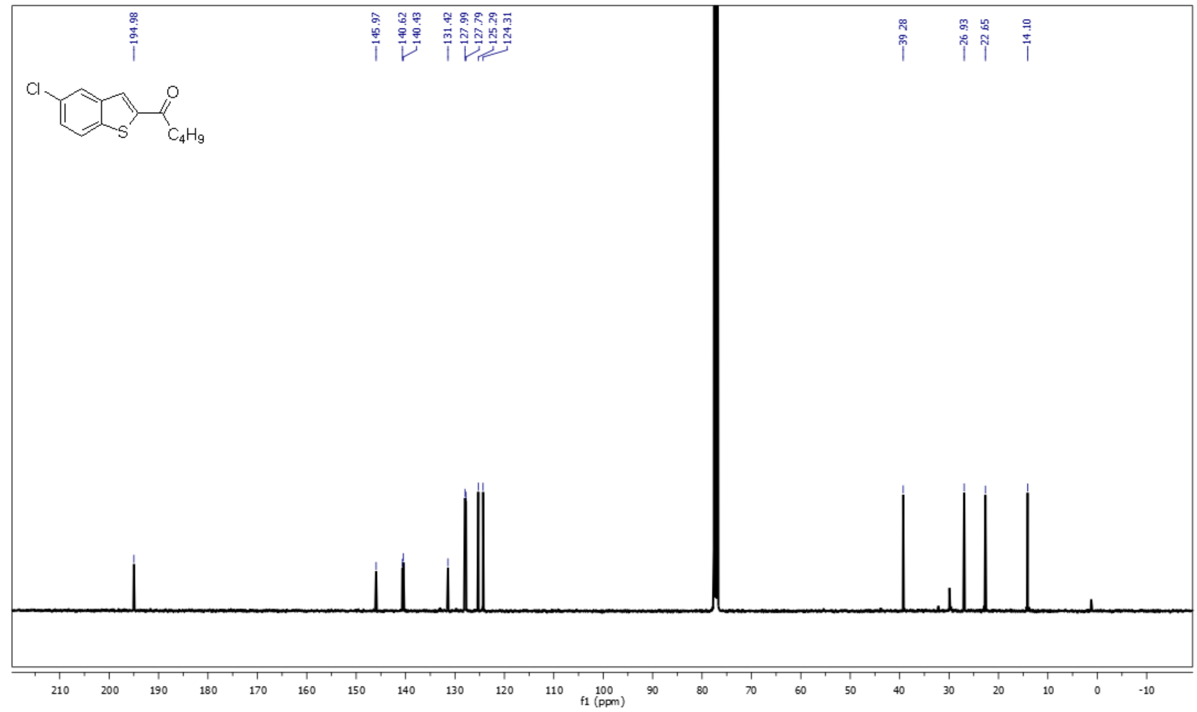

1-(5-Nitrobenzo[*b*]thiophen-2-yl)pentan-1-one S5

^{1}H NMR (400 MHz, $CDCl_3$)

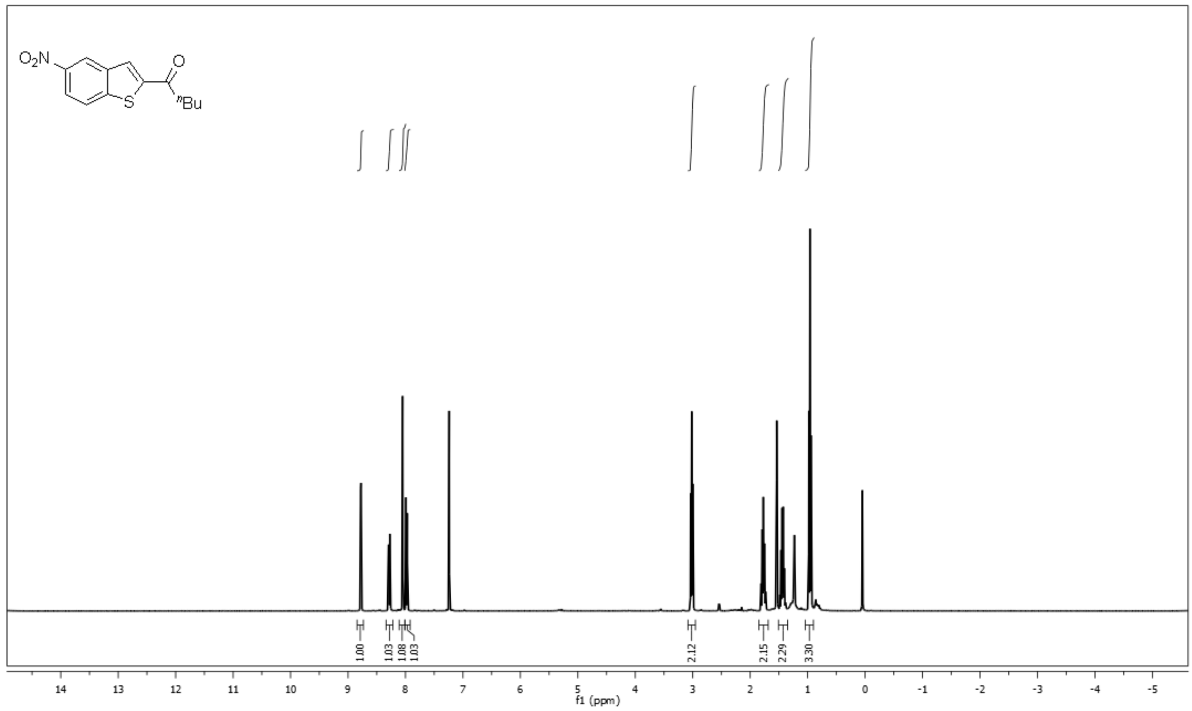

^{13}C NMR (100 MHz, $CDCl_3$)

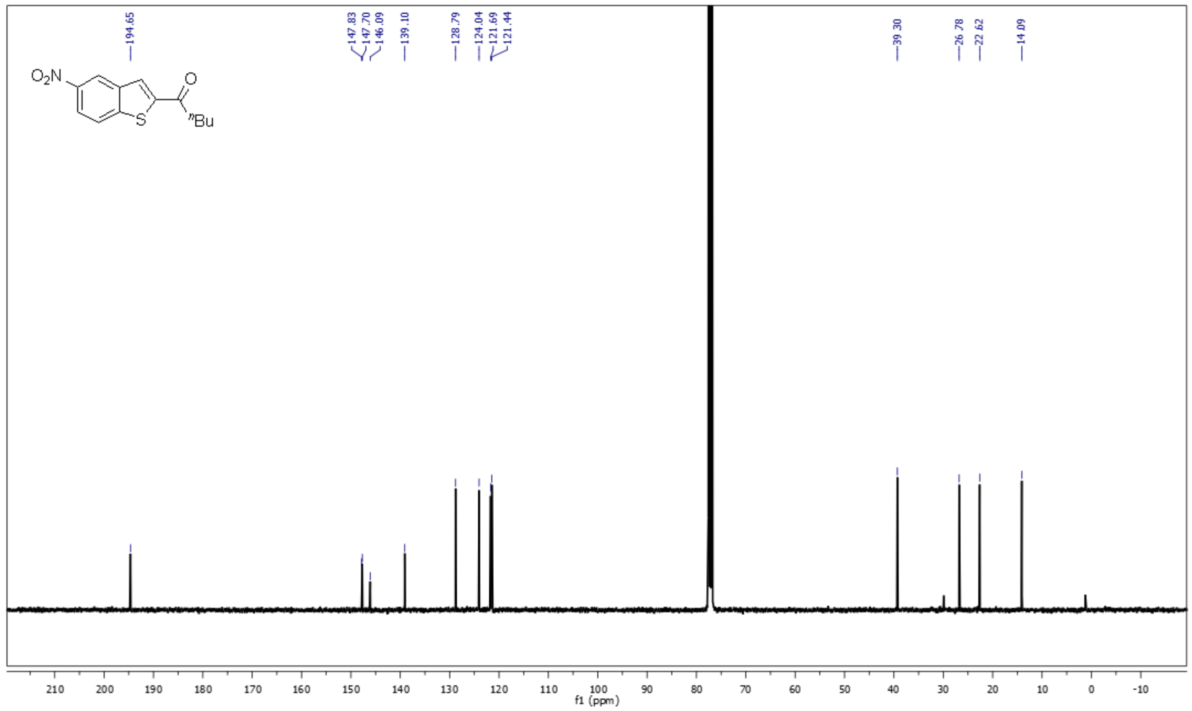

1-(5-Methoxybenzo[*b*]thiophen-2-yl)pentan-1-one S6

^{1}H NMR (400 MHz, $CDCl_3$)

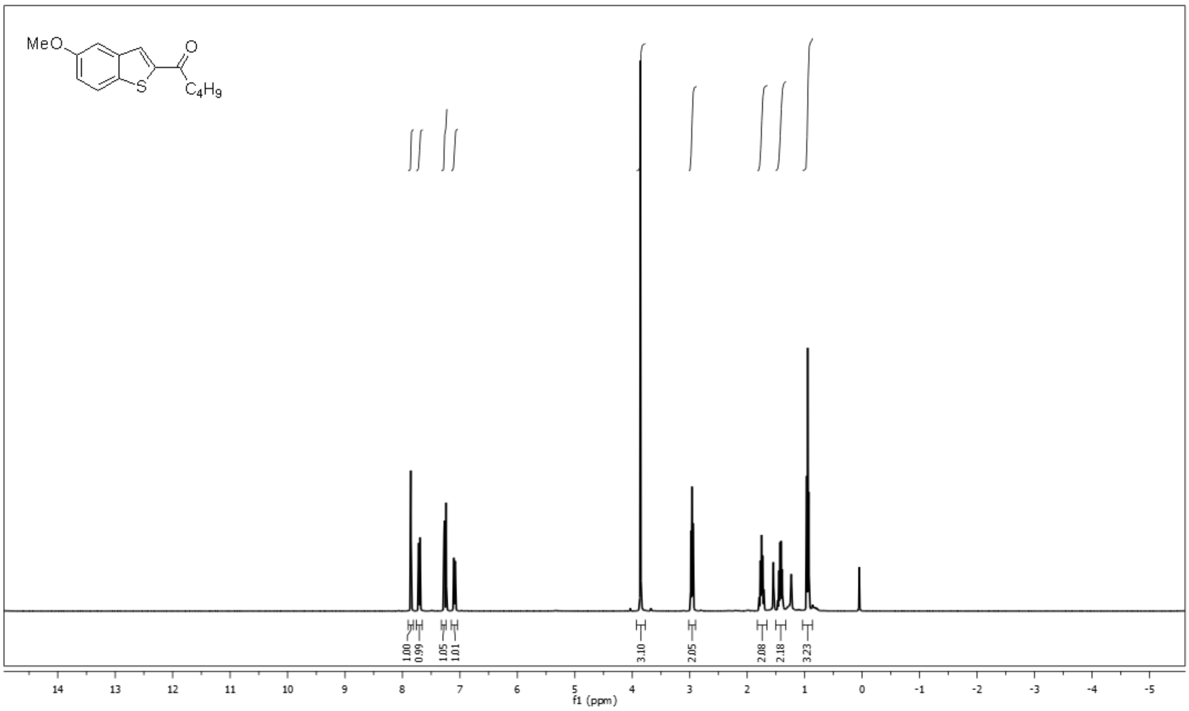

^{13}C NMR (100 MHz, $CDCl_3$)

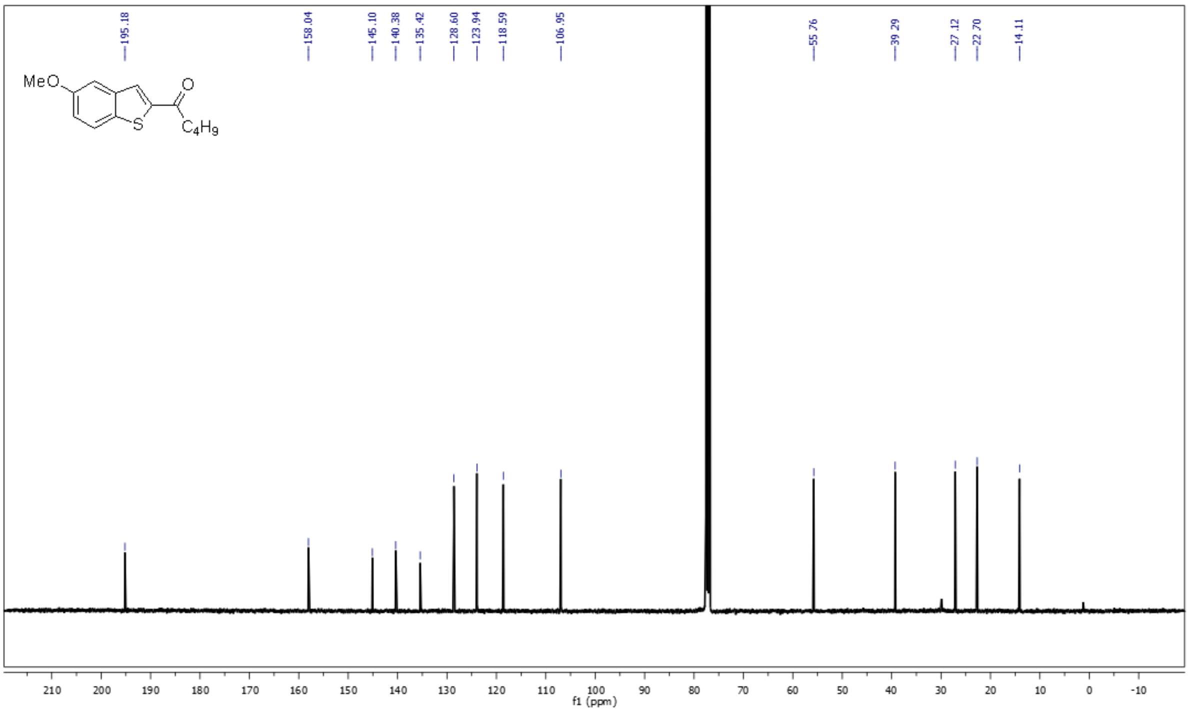

1-(7-Methoxybenzo[*b*]thiophen-2-yl)pentan-1-one S7

^{1}H NMR (400 MHz, $CDCl_3$)

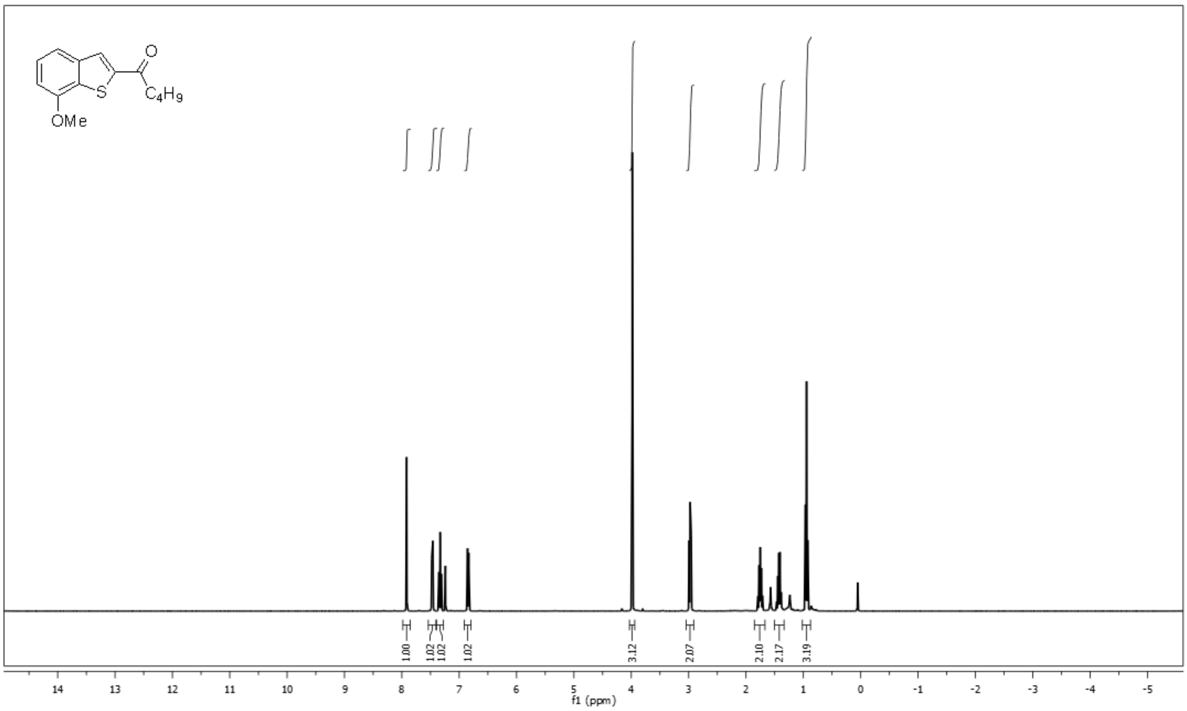

^{13}C NMR (100 MHz, $CDCl_3$)

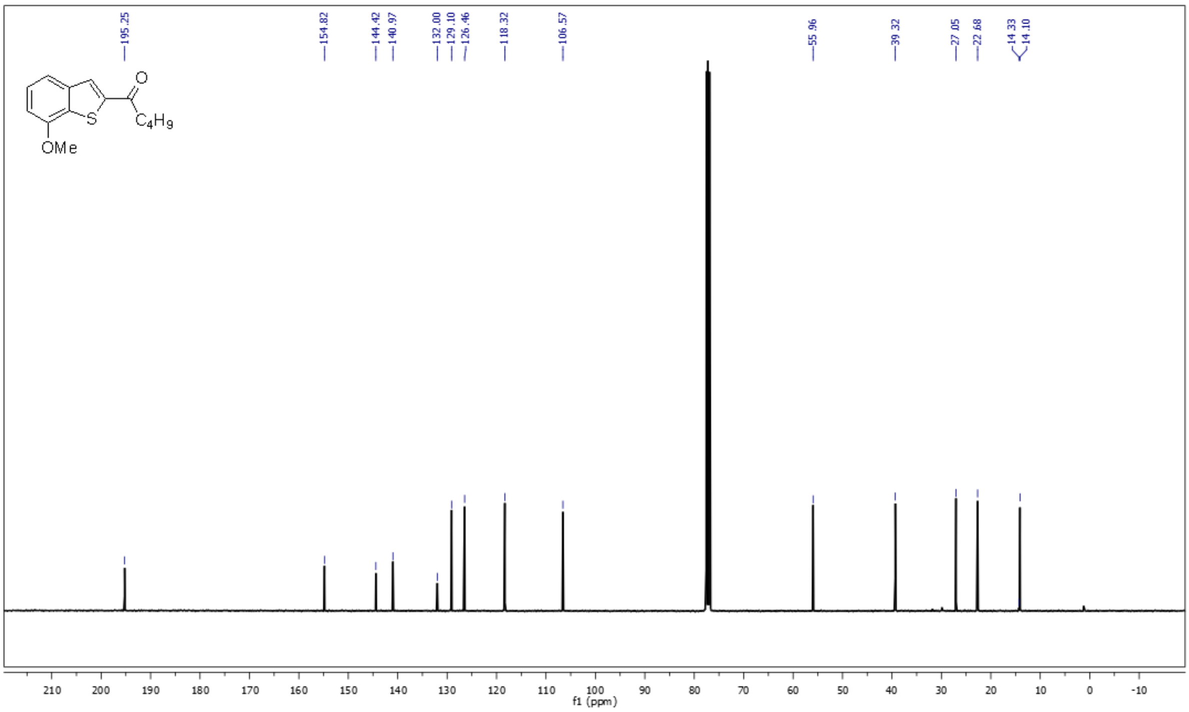

1-(7-(Trifluoromethyl)benzo[*b*]thiophen-2-yl)pentan-1-one S8

^{1}H NMR (400 MHz, $CDCl_3$)

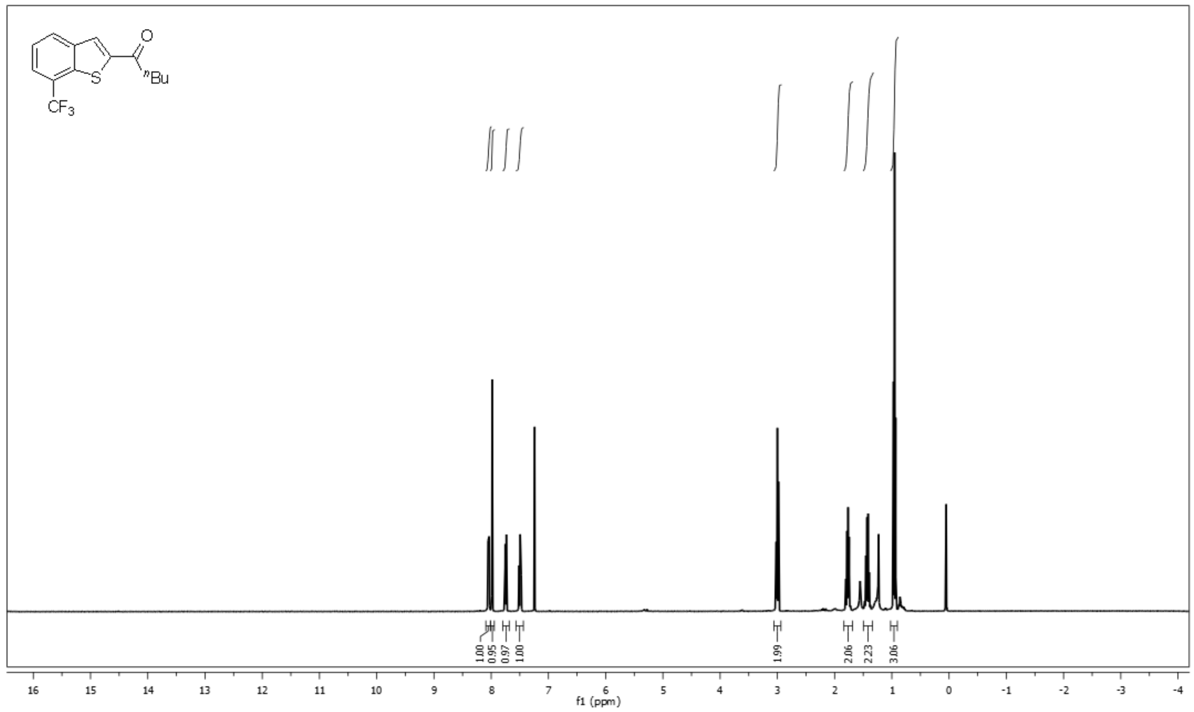

^{13}C NMR (100 MHz, $CDCl_3$)

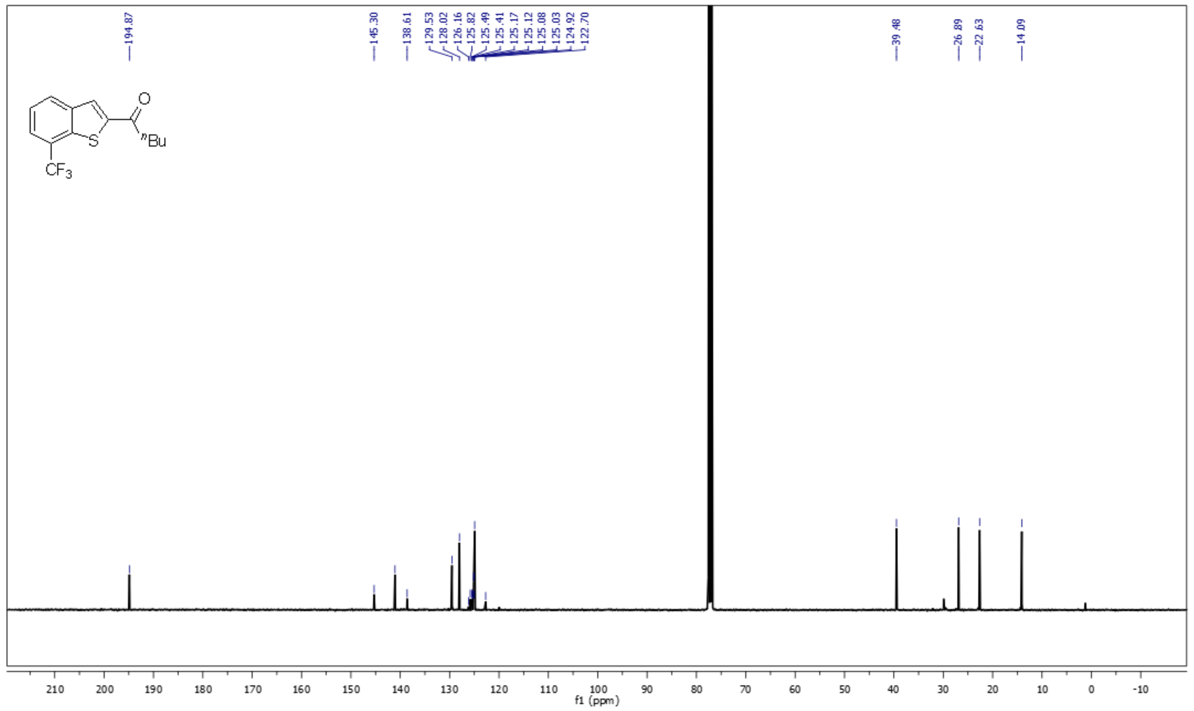

1-(5-(Trifluoromethyl)benzo[*b*]thiophen-2-yl)pentan-1-one S9

^{1}H NMR (500 MHz, $CDCl_3$)

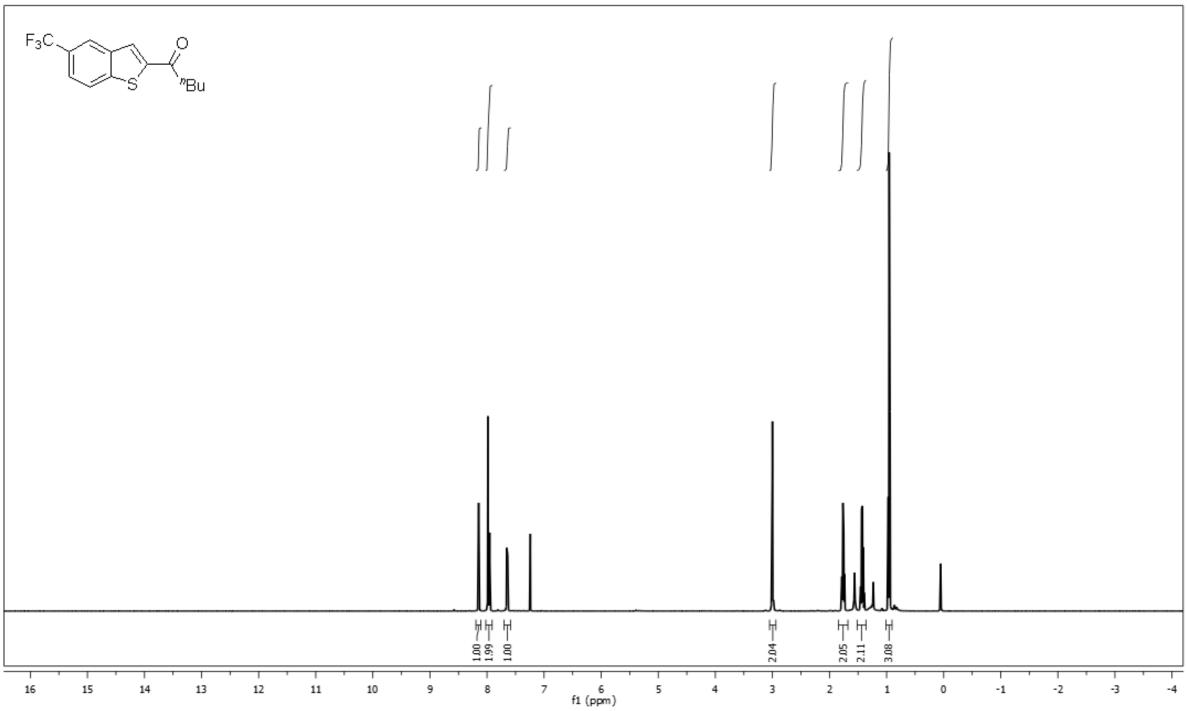

^{13}C NMR (125 MHz, $CDCl_3$)

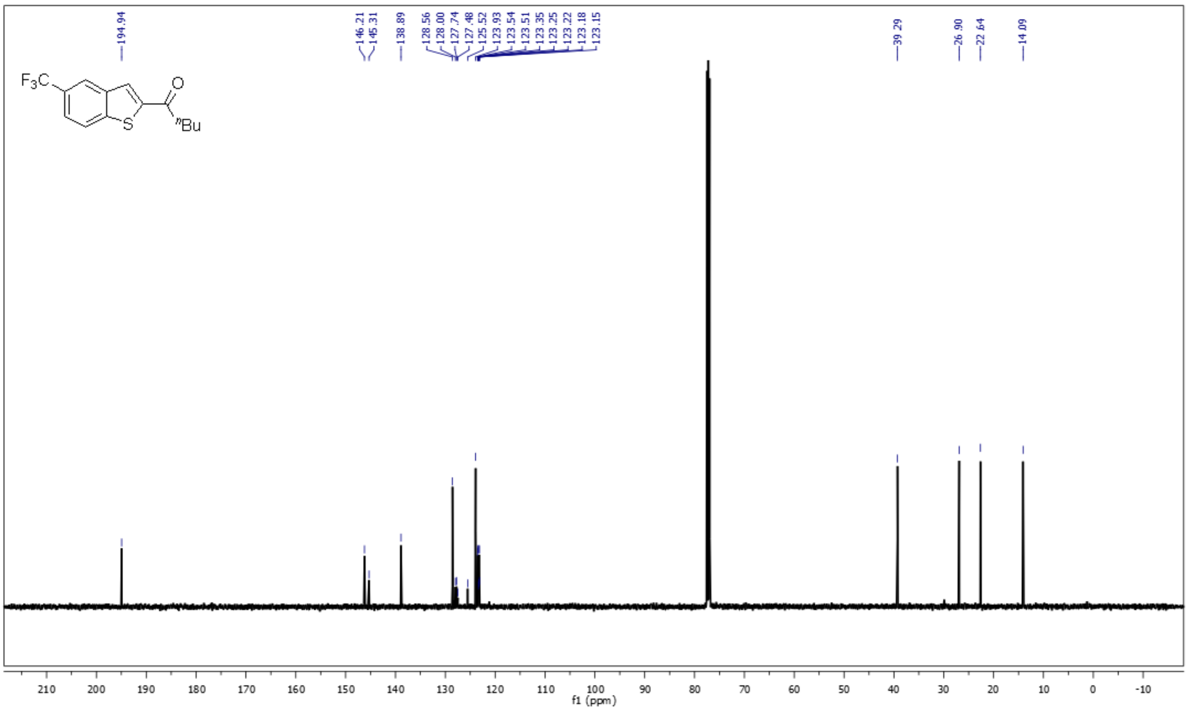

3-Ethylbenzo[*b*]thiophene-2-carbaldehyde S10

^{1}H NMR (500 MHz, $CDCl_3$)

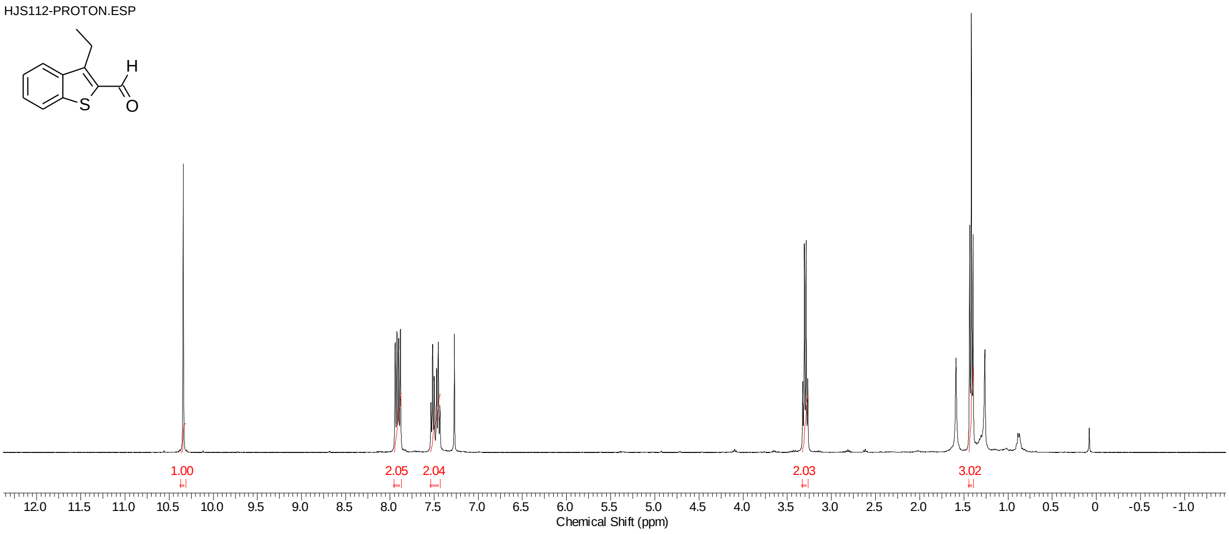

^{13}C NMR (125 MHz, $CDCl_3$)

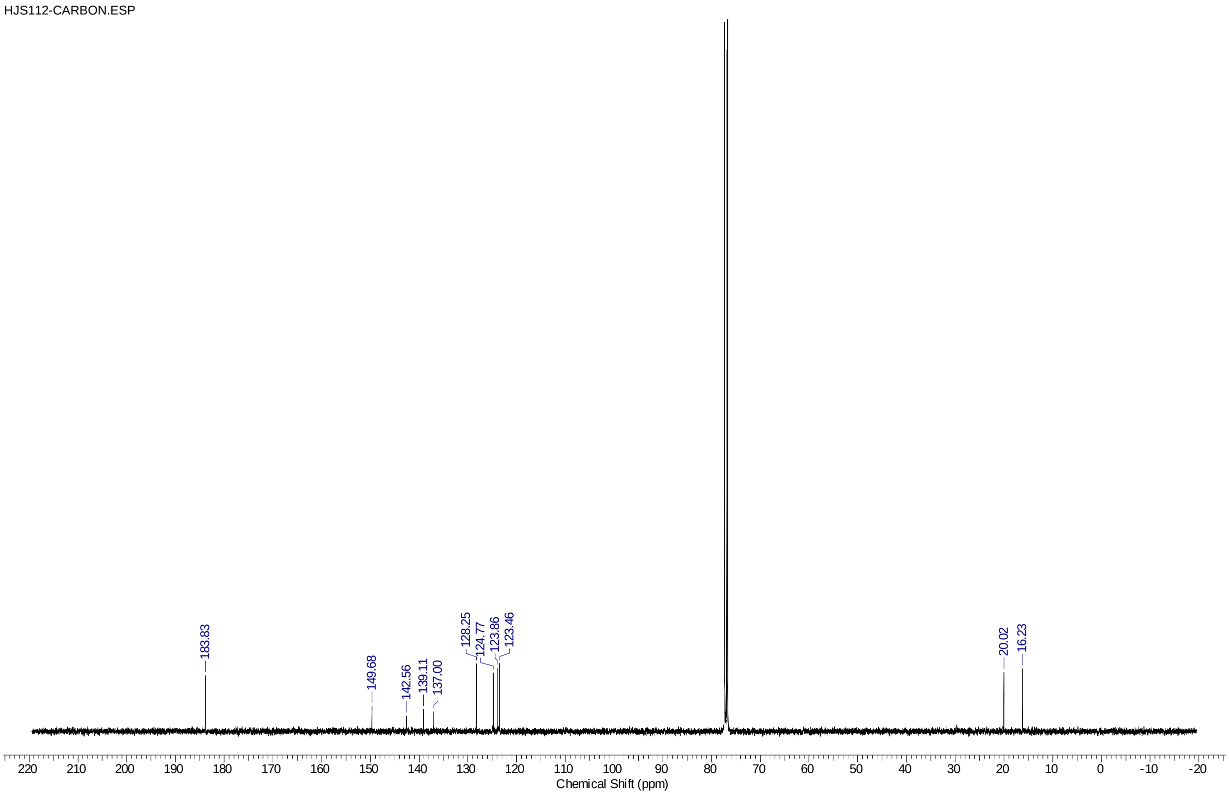

2-Pentylbenzo[*b*]thiophene S11

^{1}H NMR (500 MHz, $CDCl_3$)

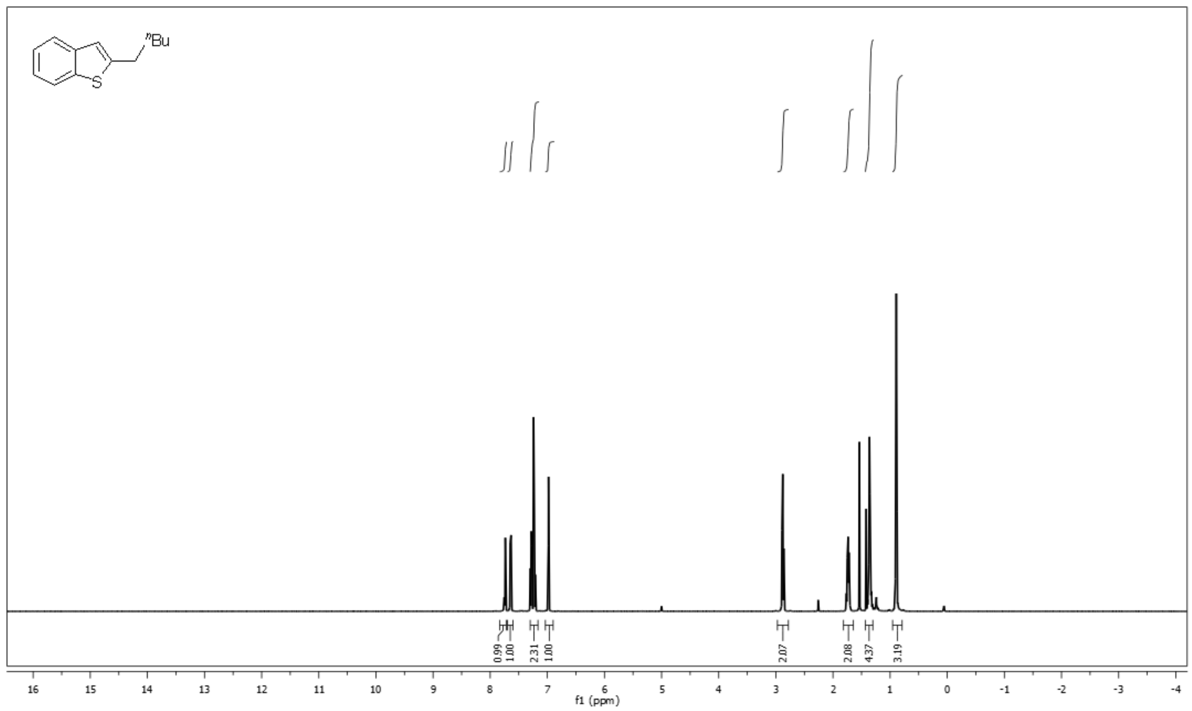

^{13}C NMR (125 MHz, $CDCl_3$)

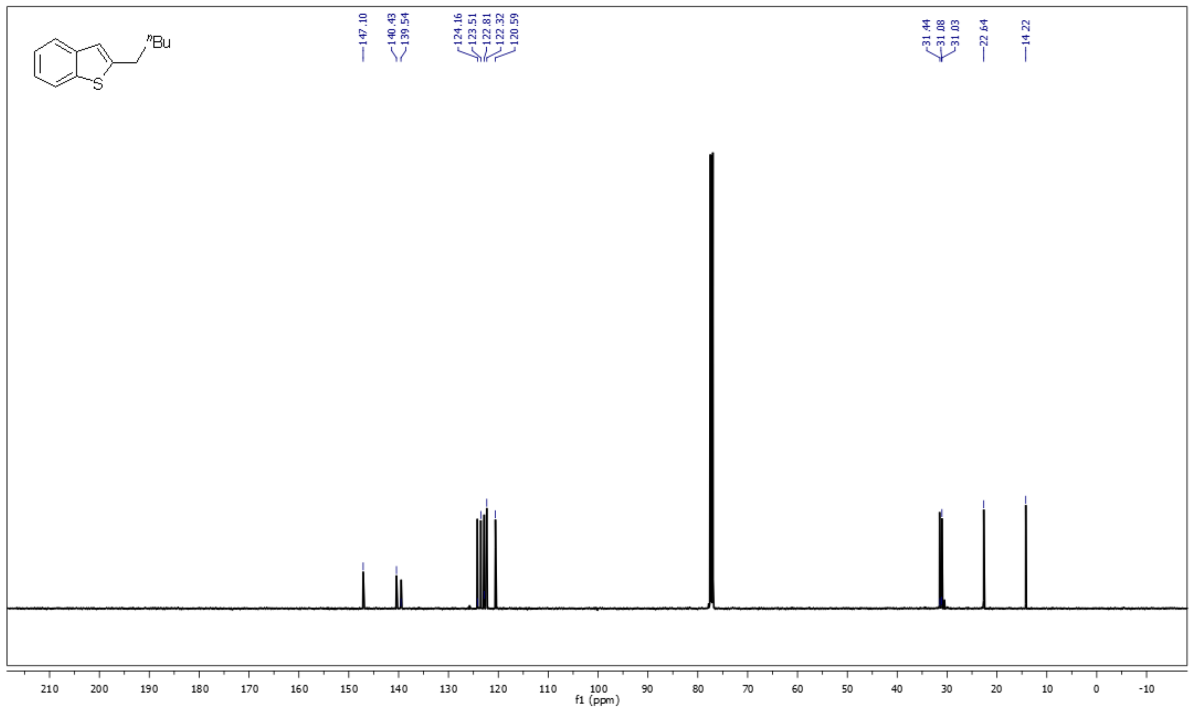

5-Methoxy-2-pentylbenzo[*b*]thiophene S12

^{1}H NMR (400 MHz, $CDCl_3$)

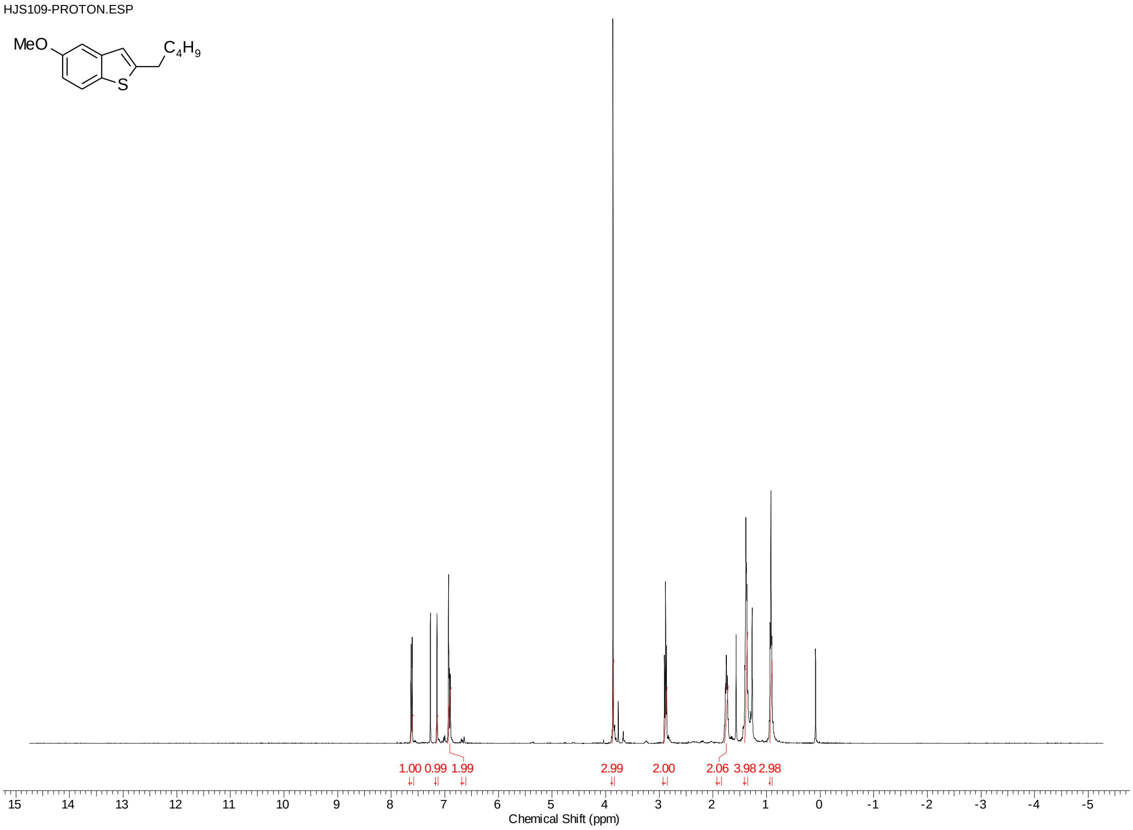

^{13}C NMR (100 MHz, $CDCl_3$)

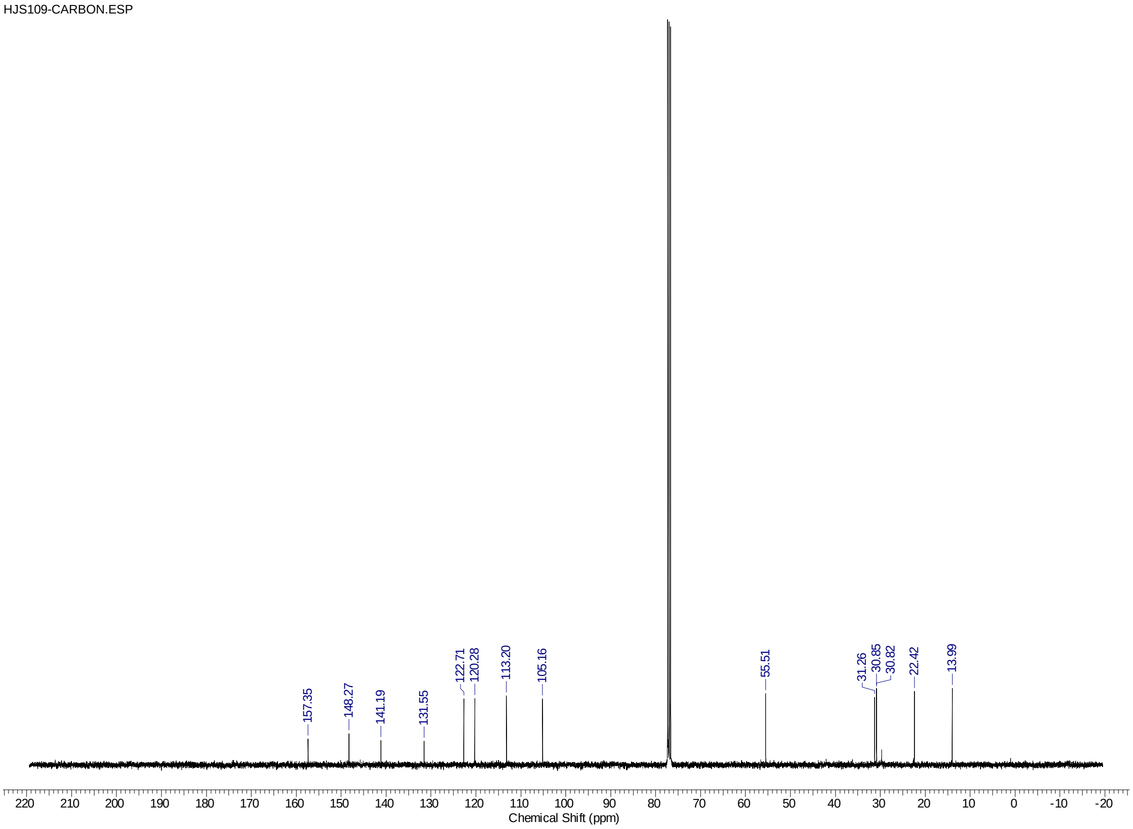

5-Chloro-2-pentylbenzo[*b*]thiophene S13

^{1}H NMR (400 MHz, $CDCl_3$)

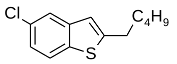

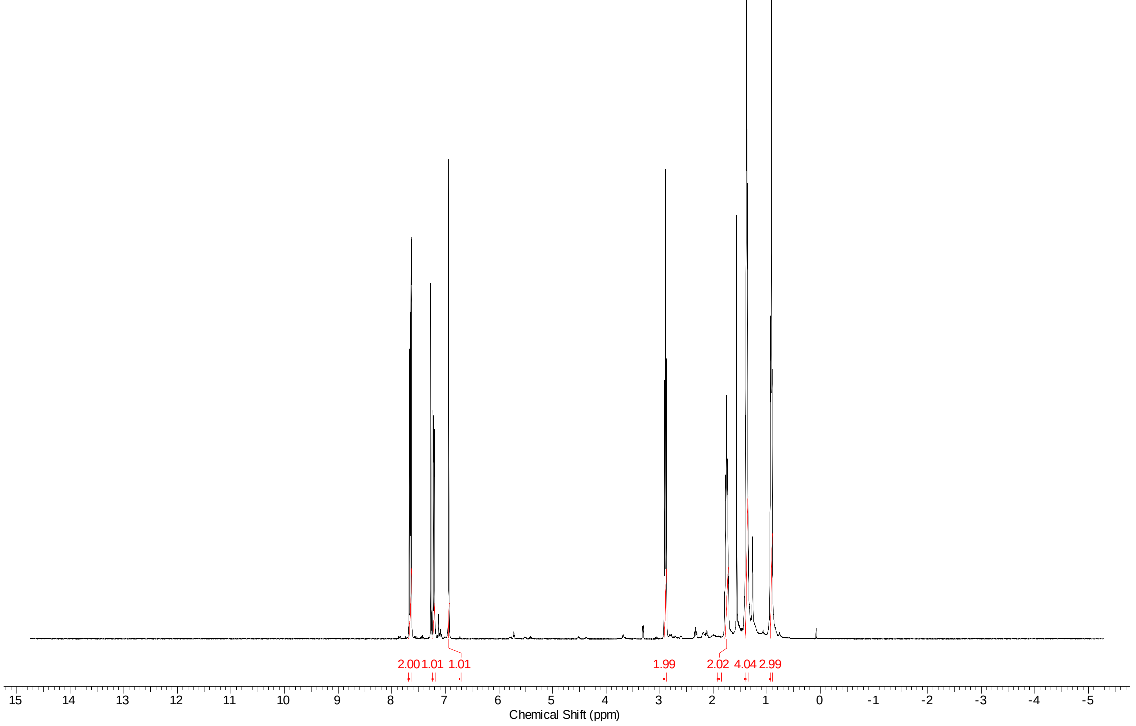

^{13}C NMR (100 MHz, $CDCl_3$)

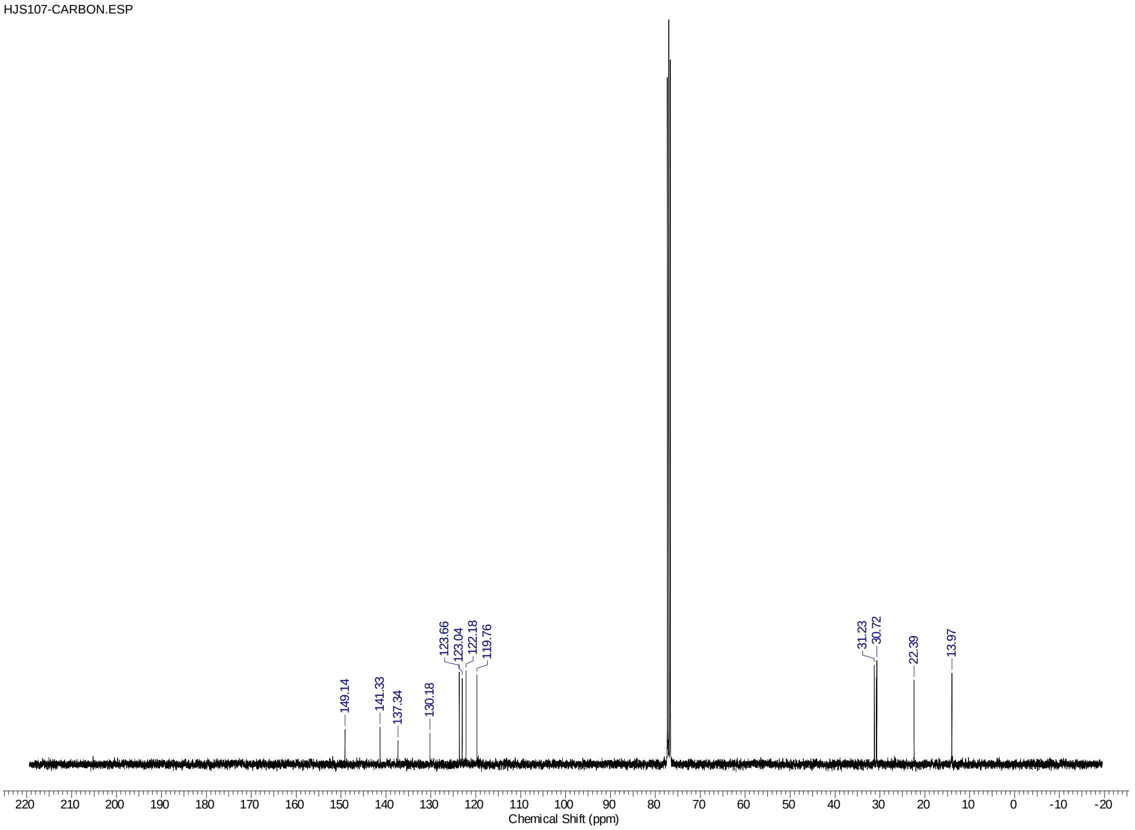

2-Pentyl-5-(trifluoromethyl)benzo[b]thiophenes S14

^{1}H NMR (400 MHz, $CDCl_3$)

2014-09-01-DJP-47.010.001.1R.esp

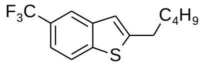

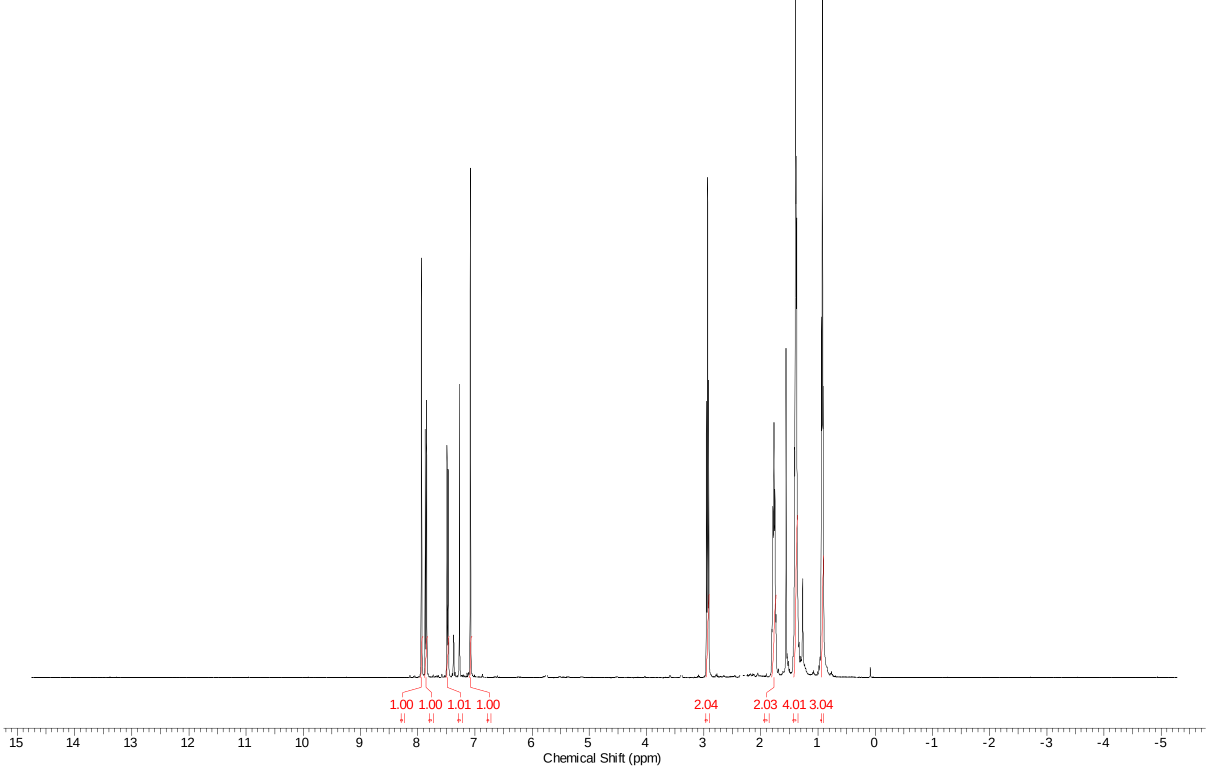

^{13}C NMR (100 MHz, $CDCl_3$)

2015-07-16-DJP-44.011.001.1R.esp

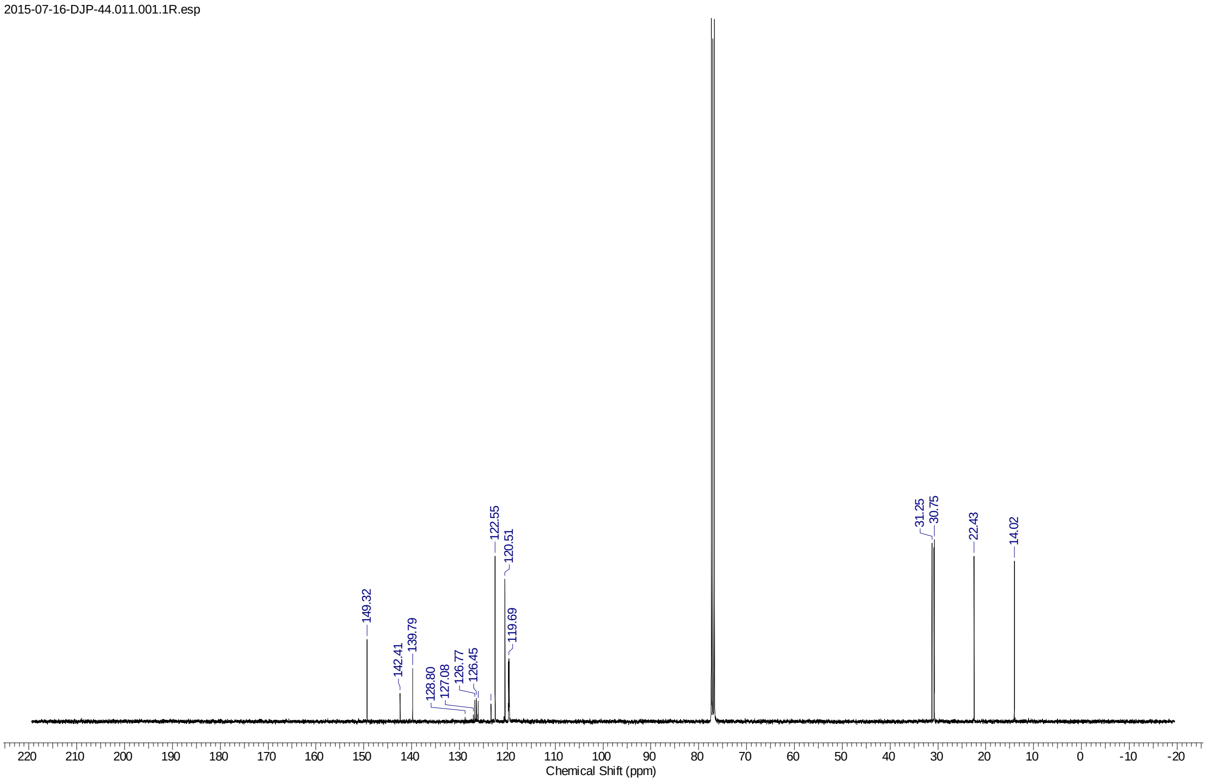

7-Fluoro-2-pentylbenzo[*b*]thiophene S15

^{1}H NMR (400 MHz, $CDCl_3$)

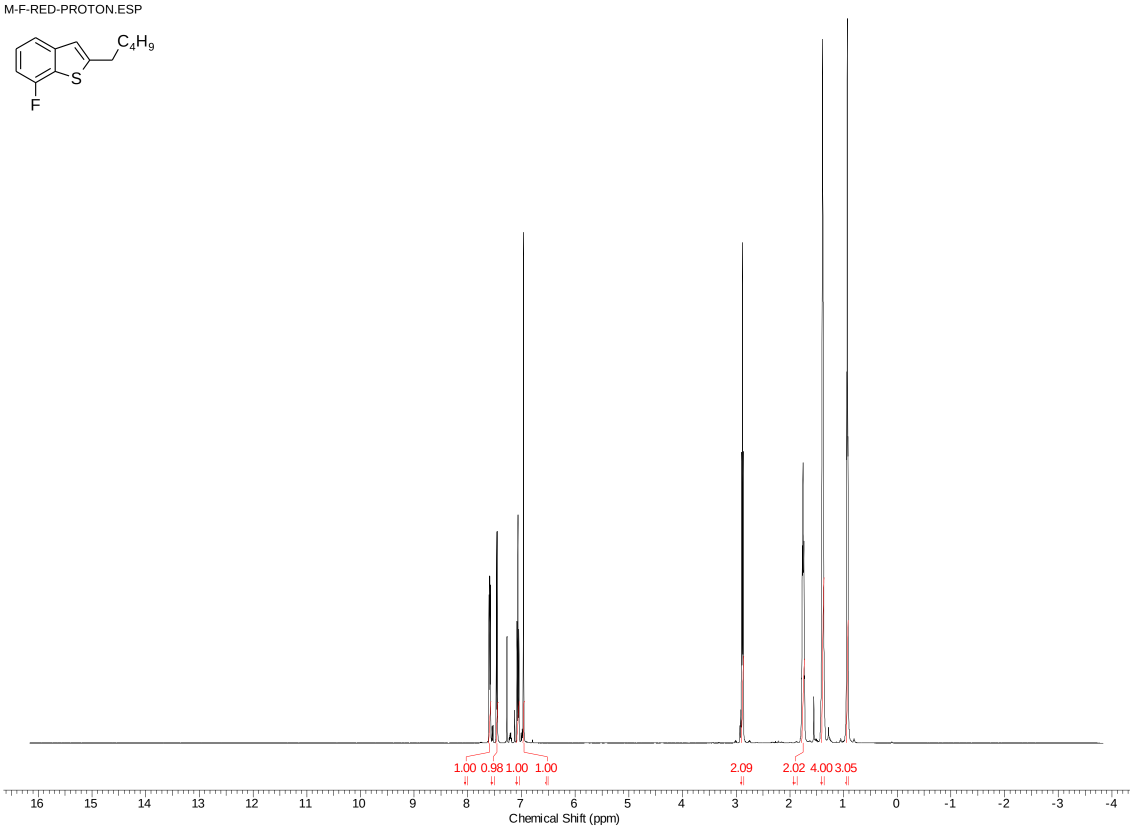

^{13}C NMR (100 MHz, $CDCl_3$)

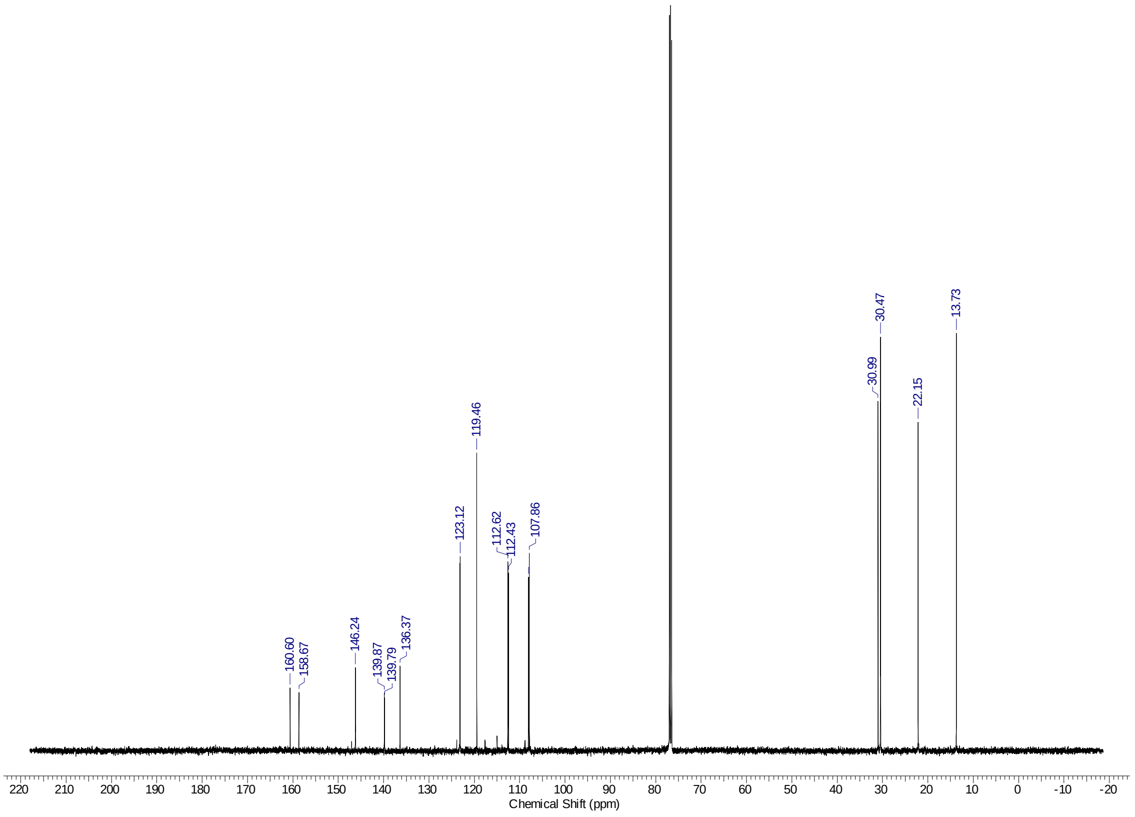

2-Methyl-3-ethylbenzothiophene S16

^{1}H NMR (400 MHz, $CDCl_3$)

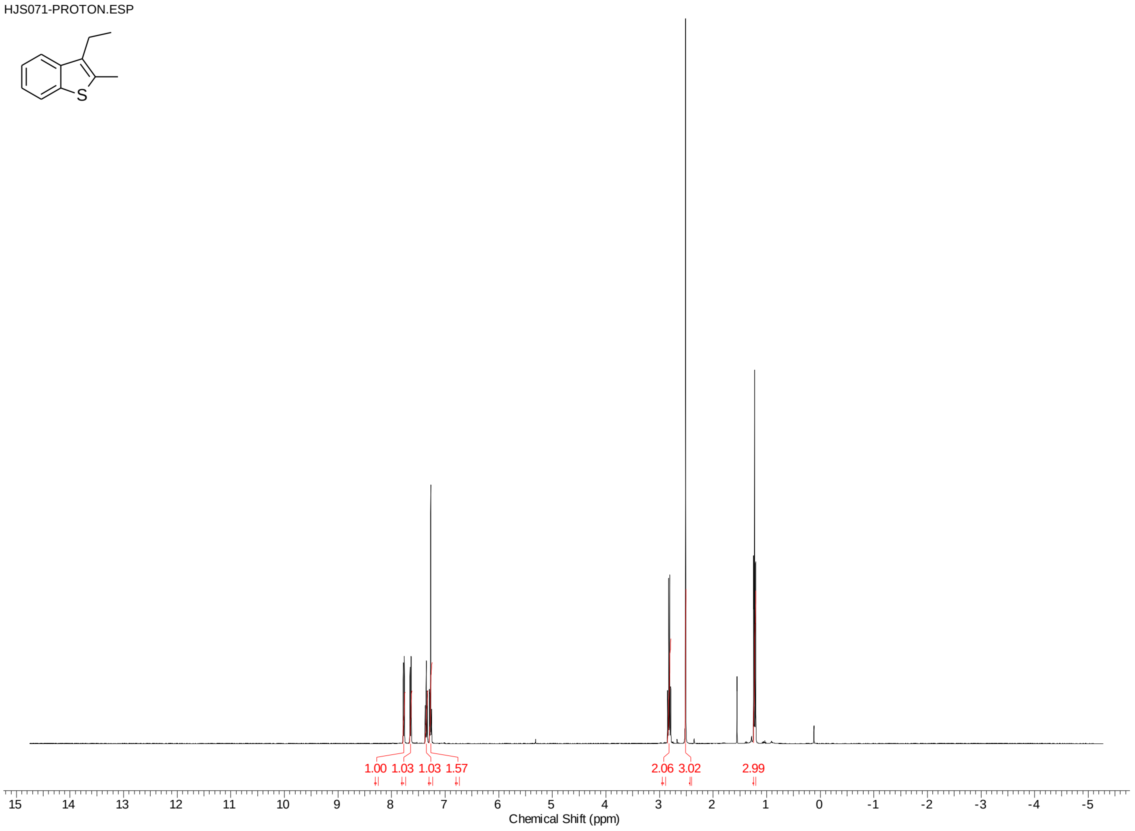

^{13}C NMR (100 MHz, $CDCl_3$)

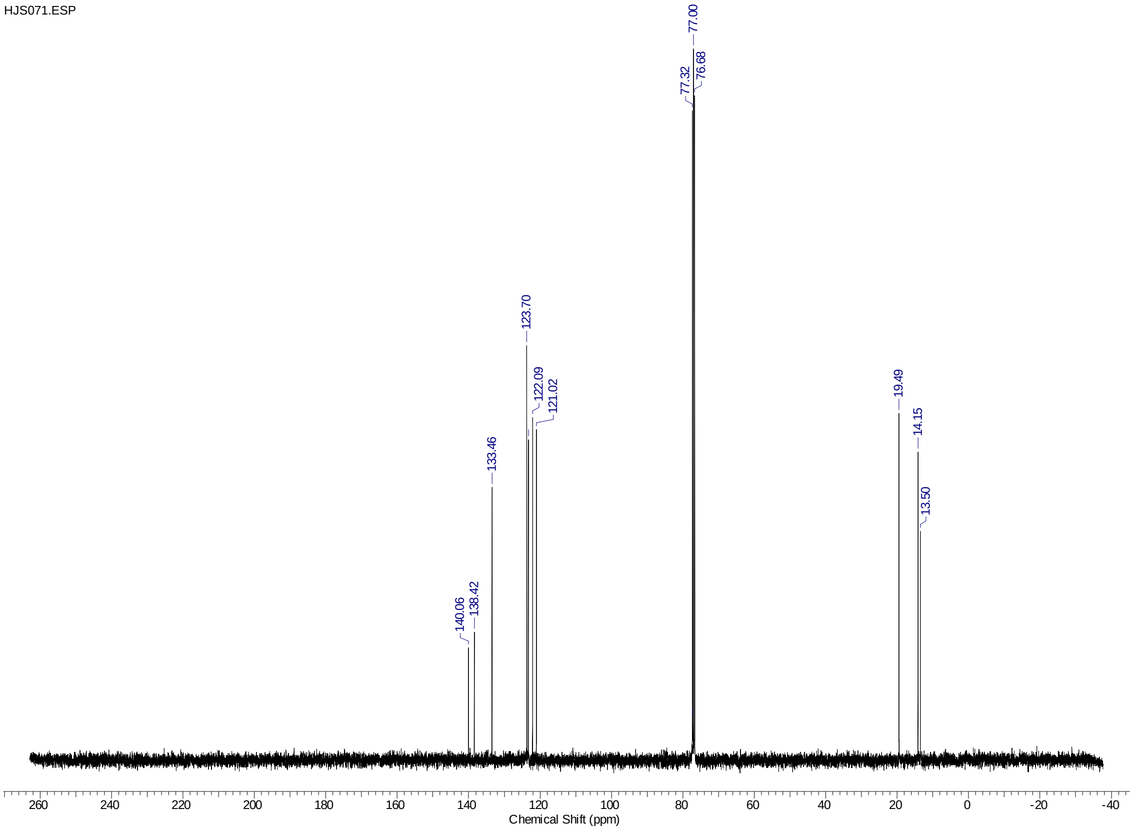

2-Methyl-3-isopropylbenzothiophene S17

^{1}H NMR (400 MHz, $CDCl_3$)

HJS073-PROTON.ESP

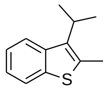

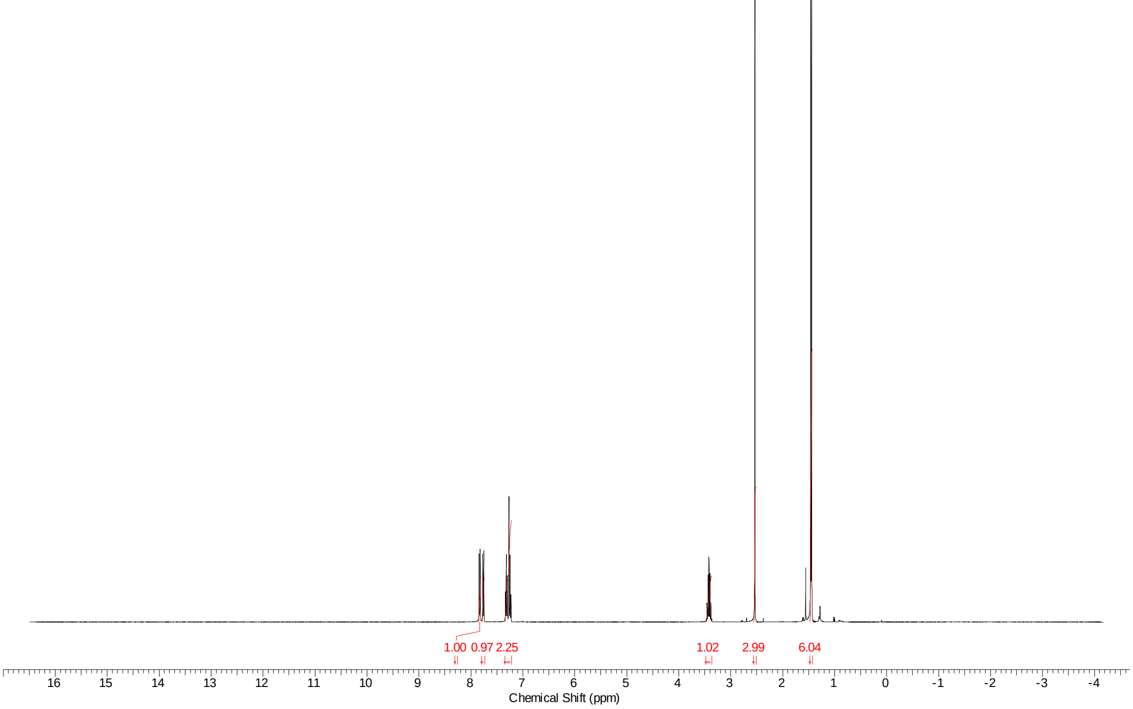

^{13}C NMR (100 MHz, $CDCl_3$)

HJS073-C.ESP

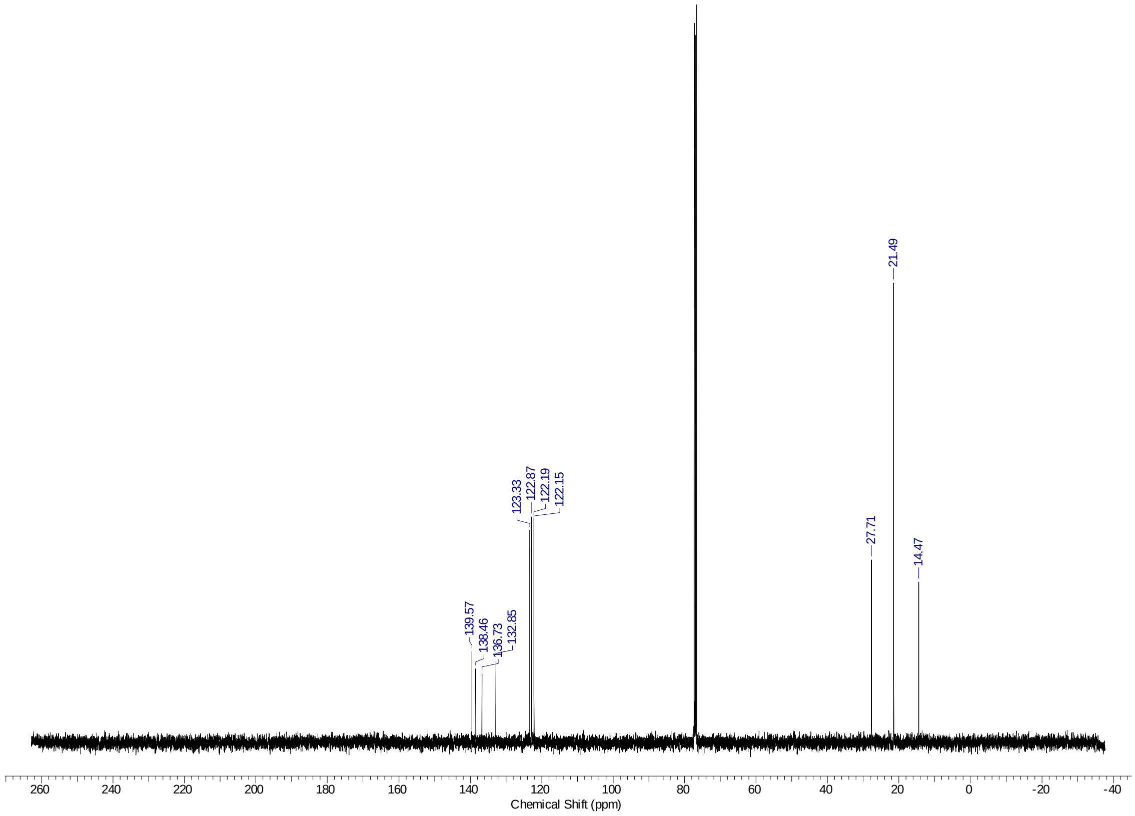

2-Methyl-3-cyclohexylbenzothiophene S18

^{1}H NMR (400 MHz, $CDCl_3$)

HJS072-PROTON.ESP

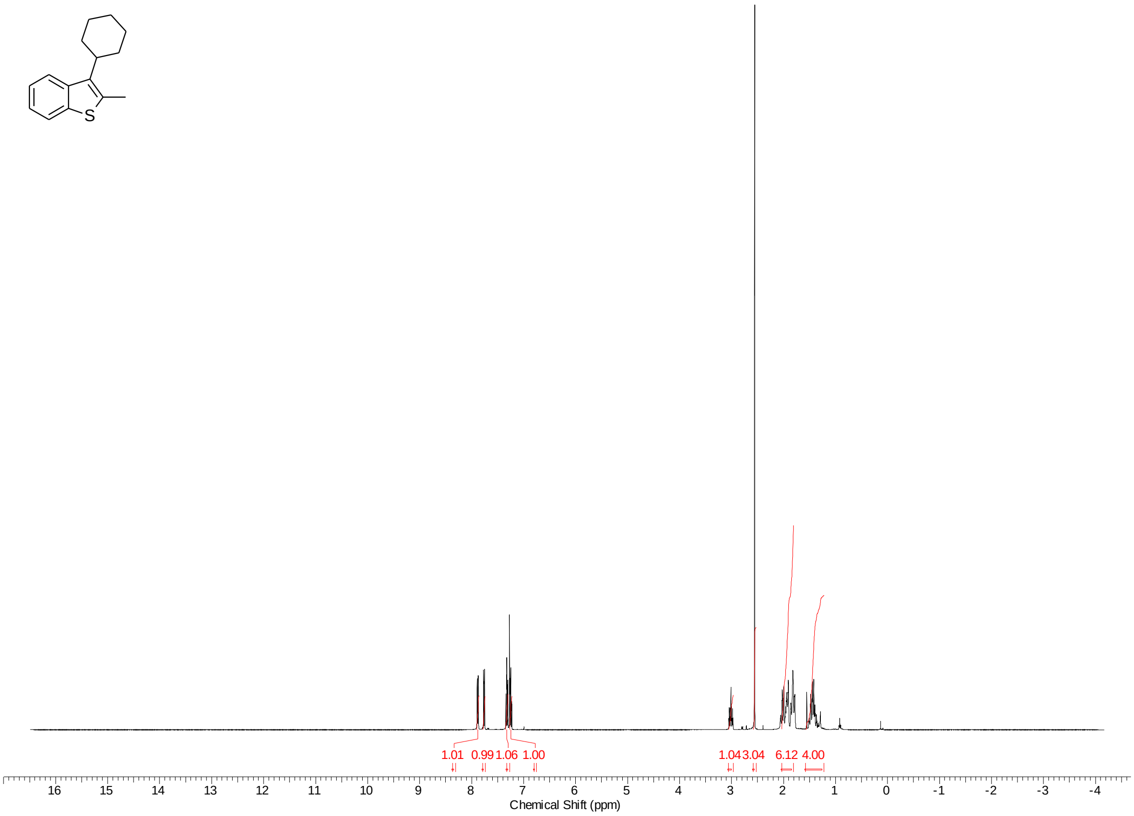

^{13}C NMR (100 MHz, $CDCl_3$)

HJS072-CARBON.ESP

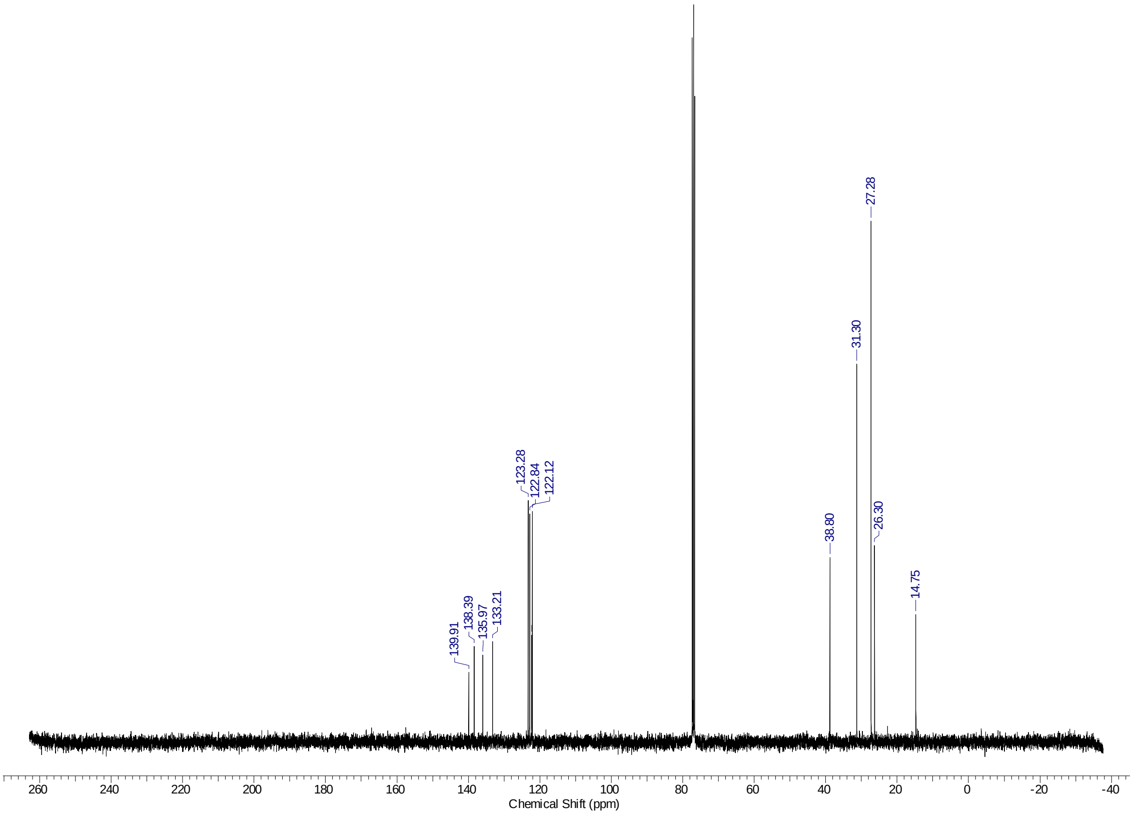

(E)-(Pent-1-en-1-yl)benzo[b]thiophenes S19

^{1}H NMR (500 MHz, C_6D_6)

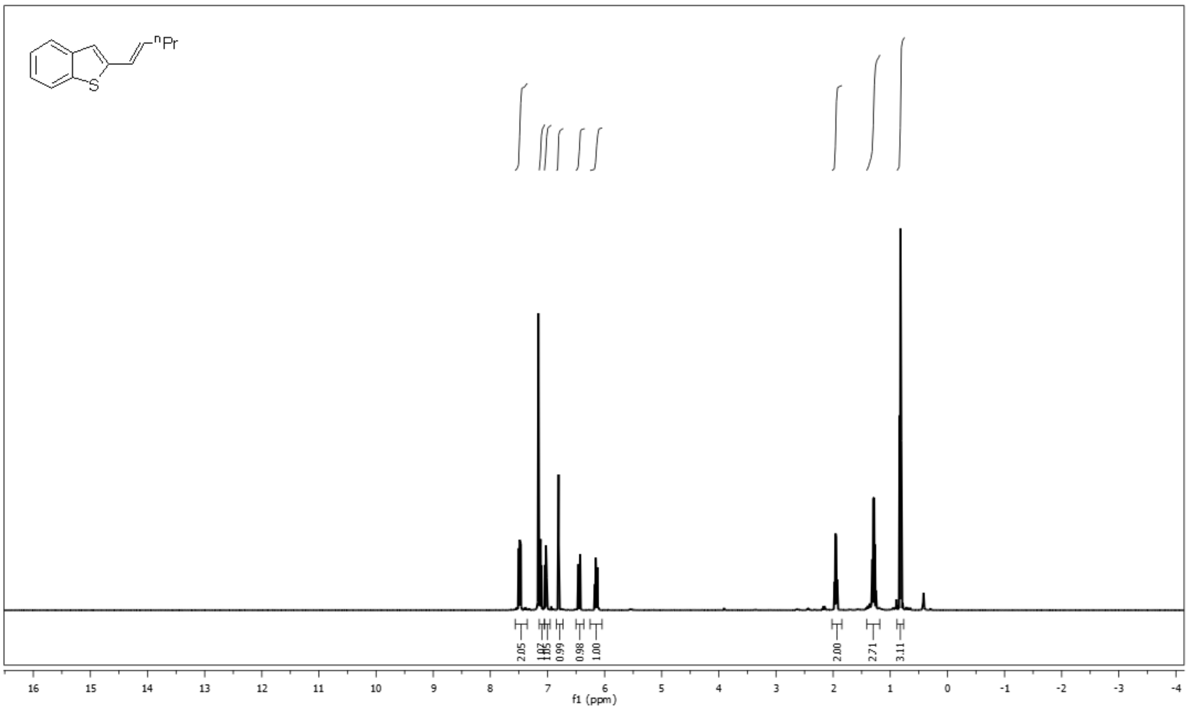

^{13}C NMR (125 MHz, C_6D_6)

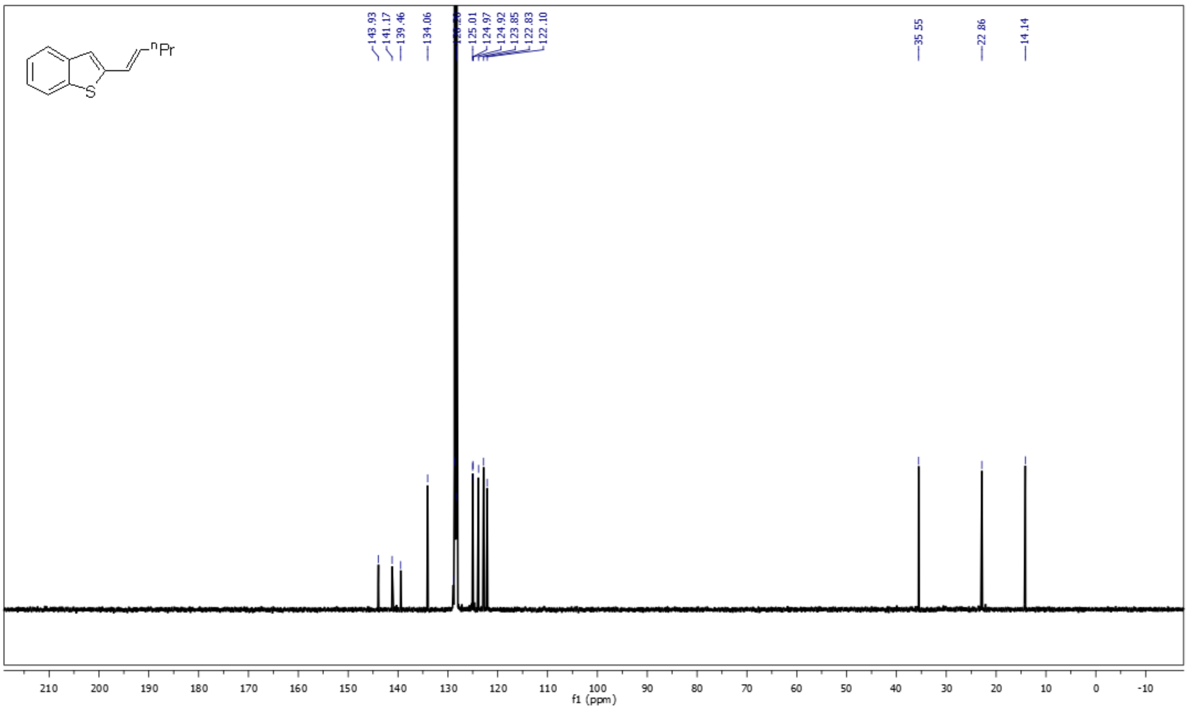

(E)-5-Methyl-2-(pent-1-en-1-yl)benzo[b]thiophene S20

^{1}H NMR (400 MHz, $CDCl_3$)

HJS006-02-PROTON.ESP

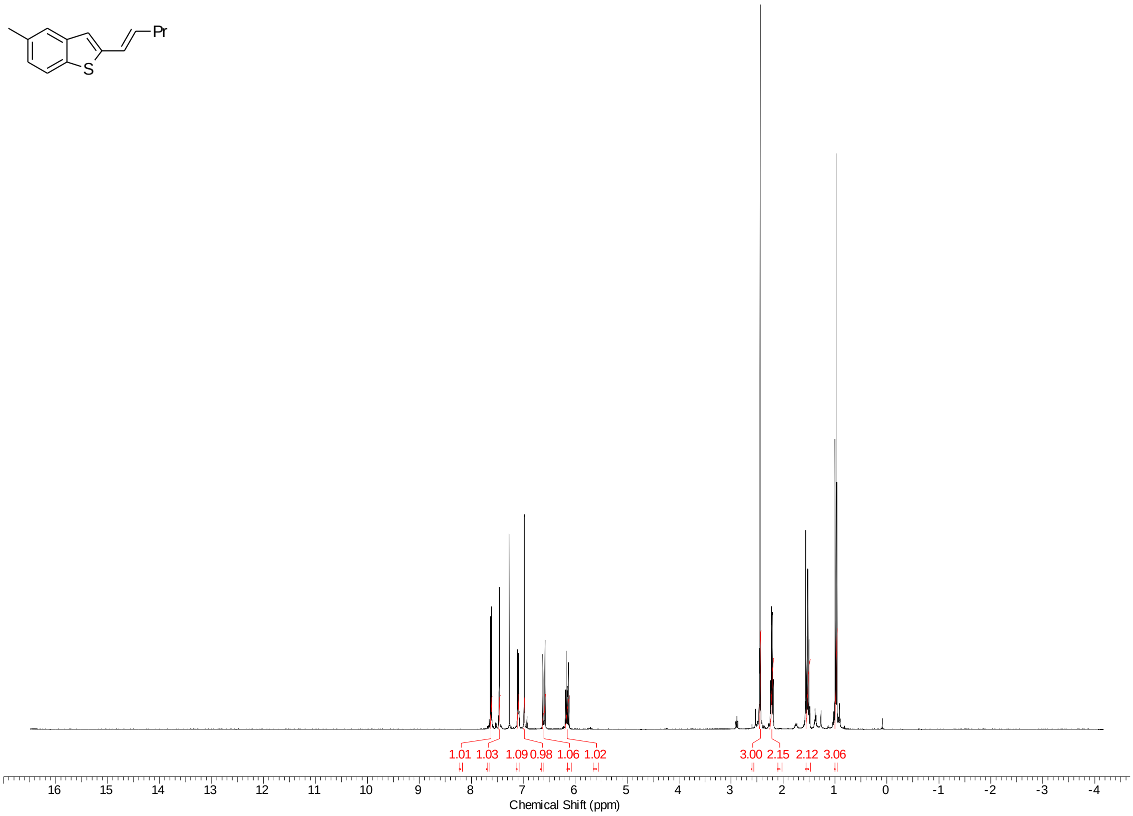

^{13}C NMR (100 MHz, $CDCl_3$)

HJS006P-ME-02-CARBON.ESP

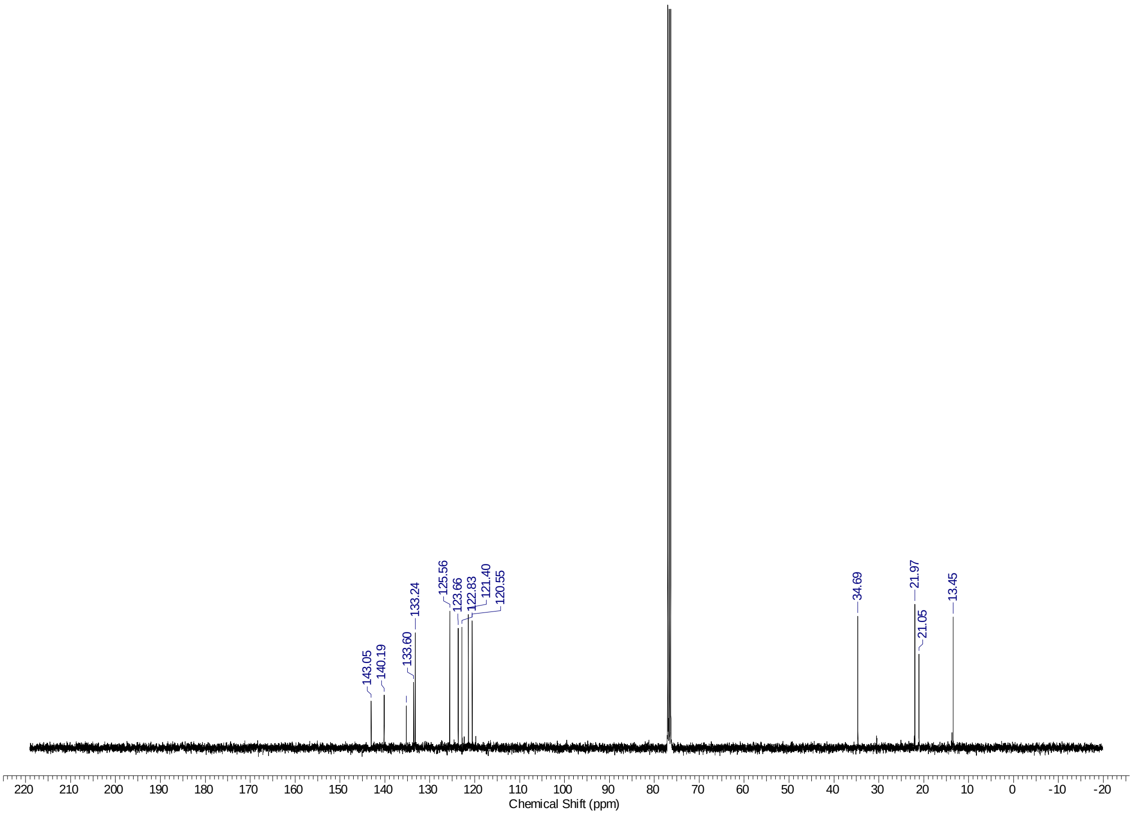

(E)-2-(Pent-1-en-1-yl)-5-(trifluoromethyl)benzo[b]thiophene S21

^{1}H NMR (400 MHz, $CDCl_3$)

P-CF3-PROTON.ESP

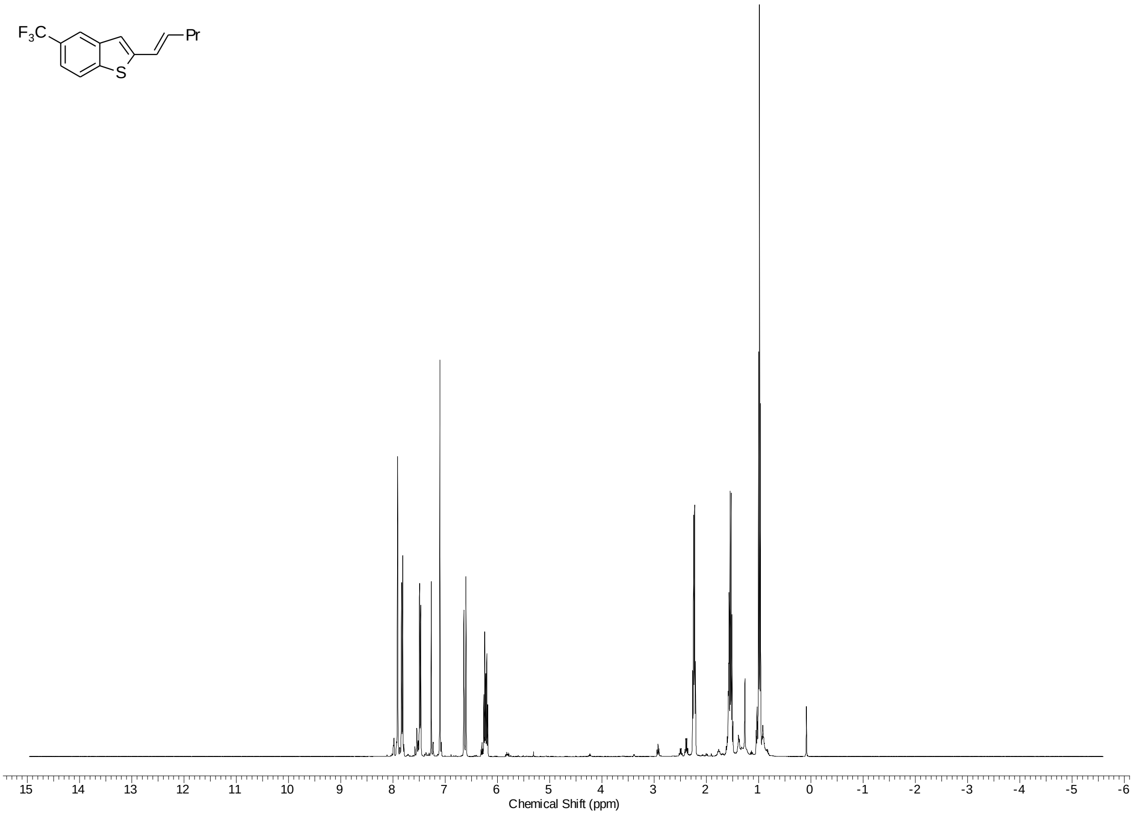

^{13}C NMR (100 MHz, $CDCl_3$)

2015-02-17-DJP-8.011.001.1R.esp

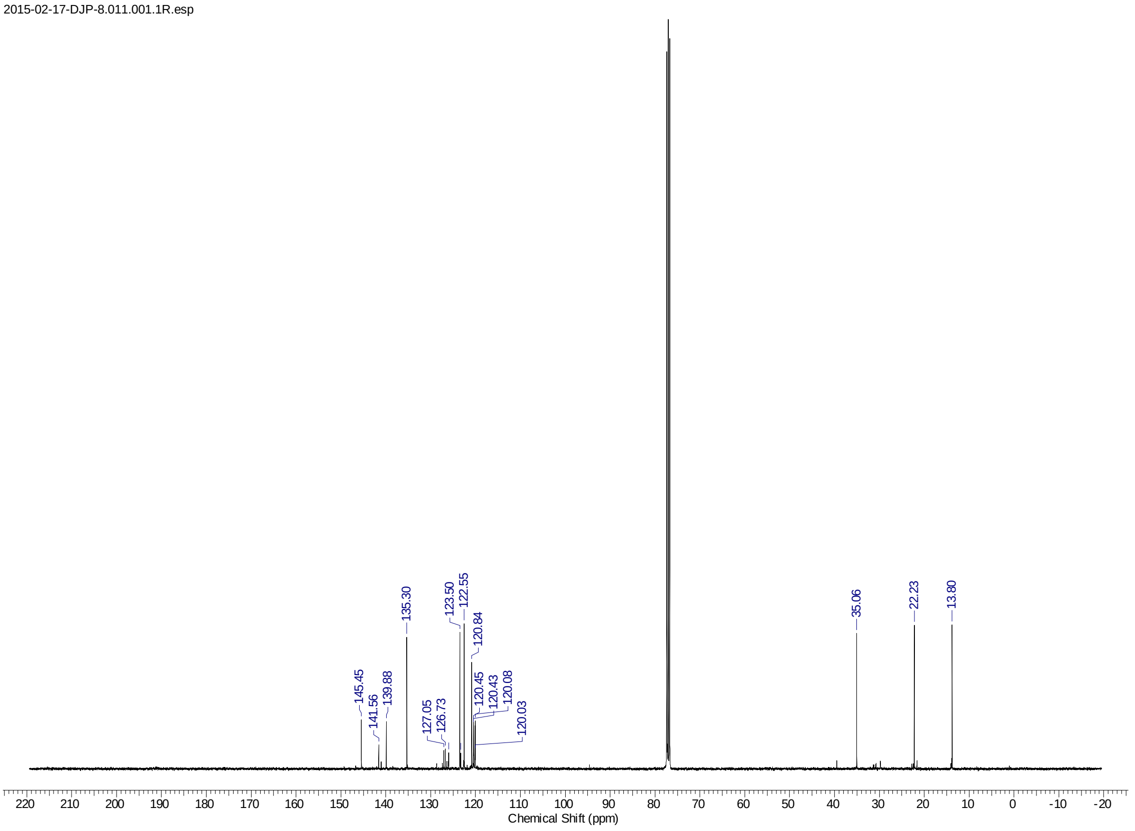

(E)-5-Fluoro-2-(pent-1-en-1-yl)benzo[b]thiophene S22

^{1}H NMR (400 MHz, $CDCl_3$)

20151102-1334-B400_B.12-22.010.001.1R.esp

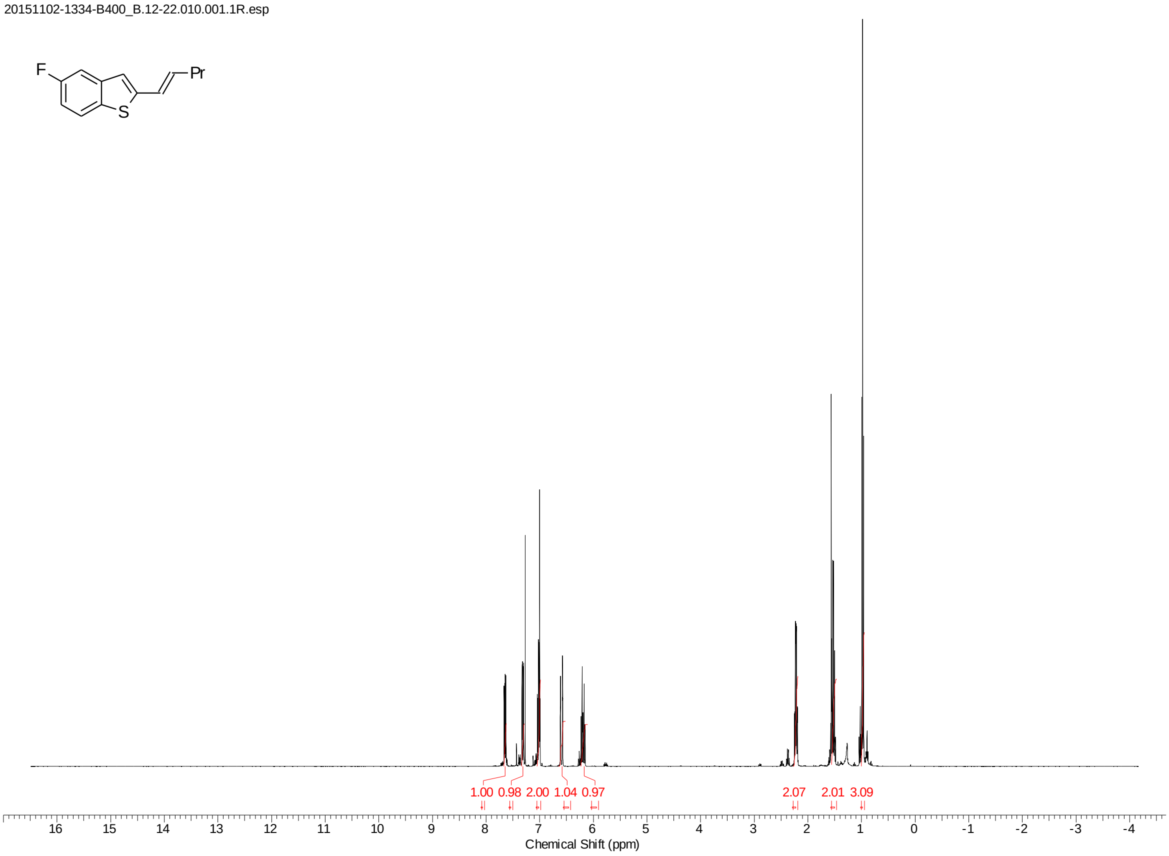

^{13}C NMR (100 MHz, $CDCl_3$)

P-F-CARBON.ESP

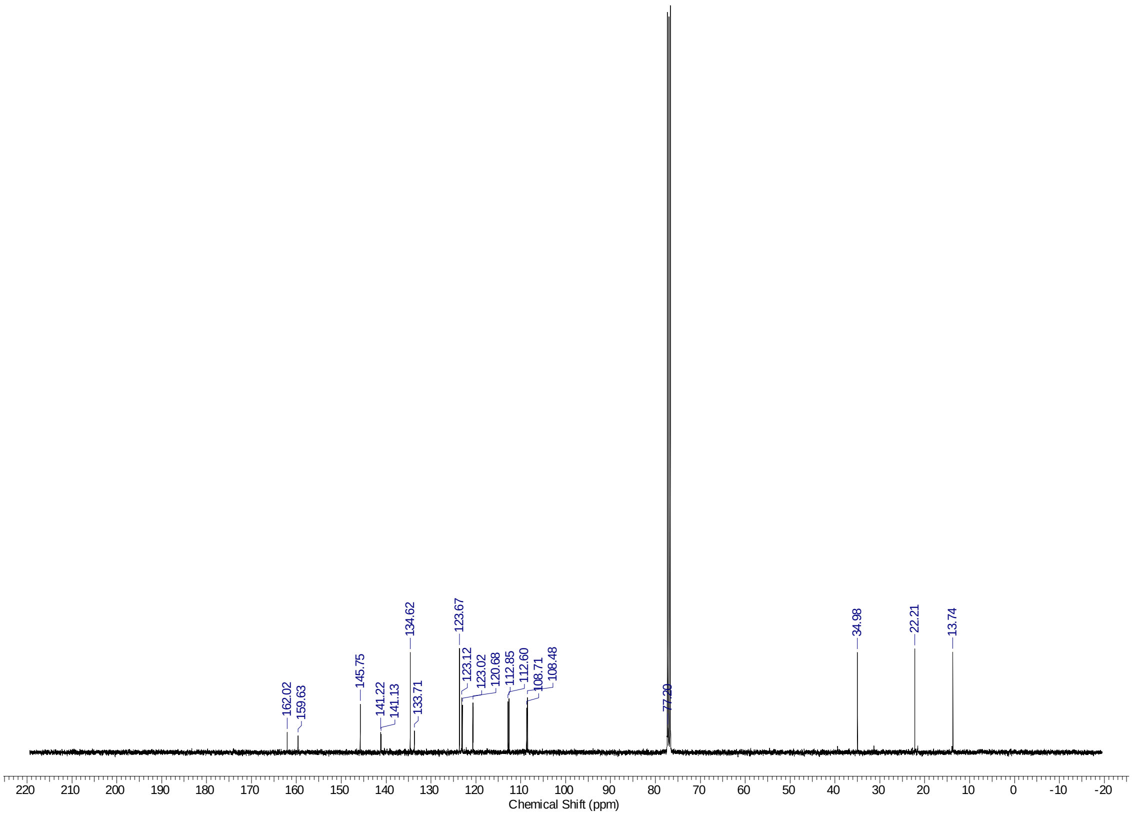

(E)-4-Fluoro-2-(pent-1-en-1-yl)benzo[b]thiophene S23

^{1}H NMR (400 MHz, $CDCl_3$)

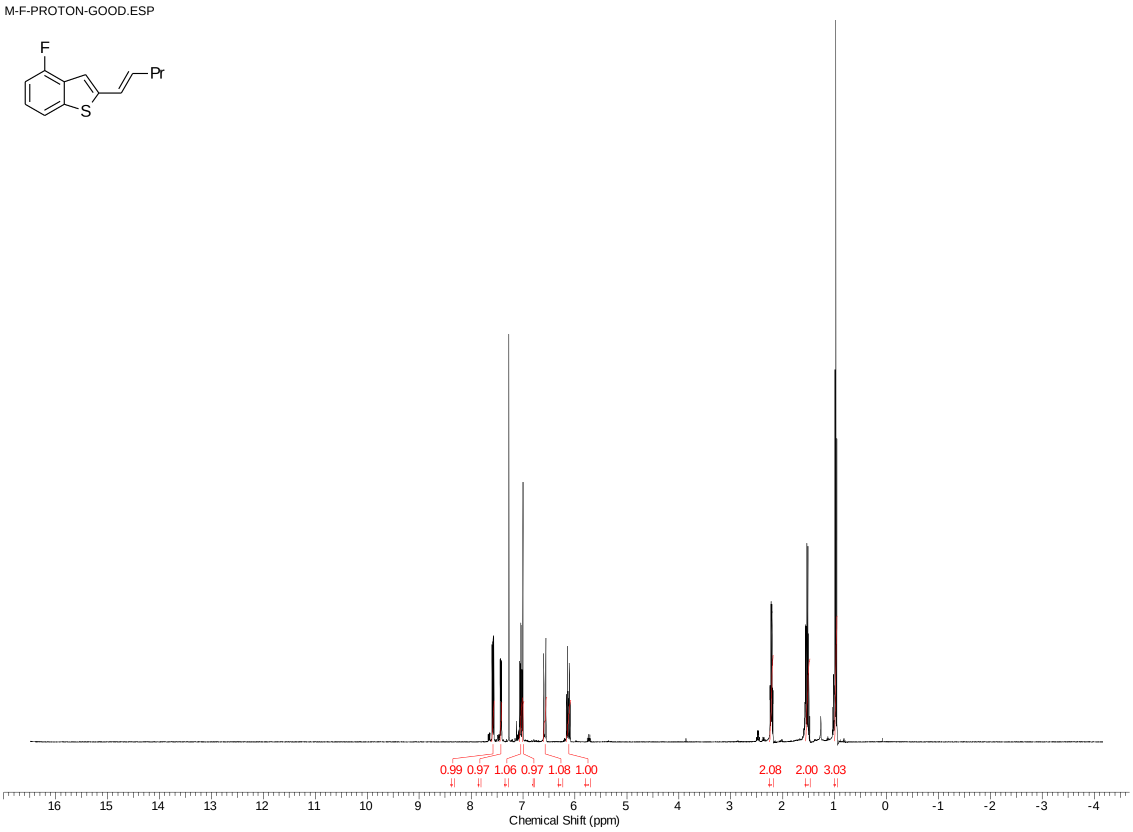

^{13}C NMR (100 MHz, $CDCl_3$)

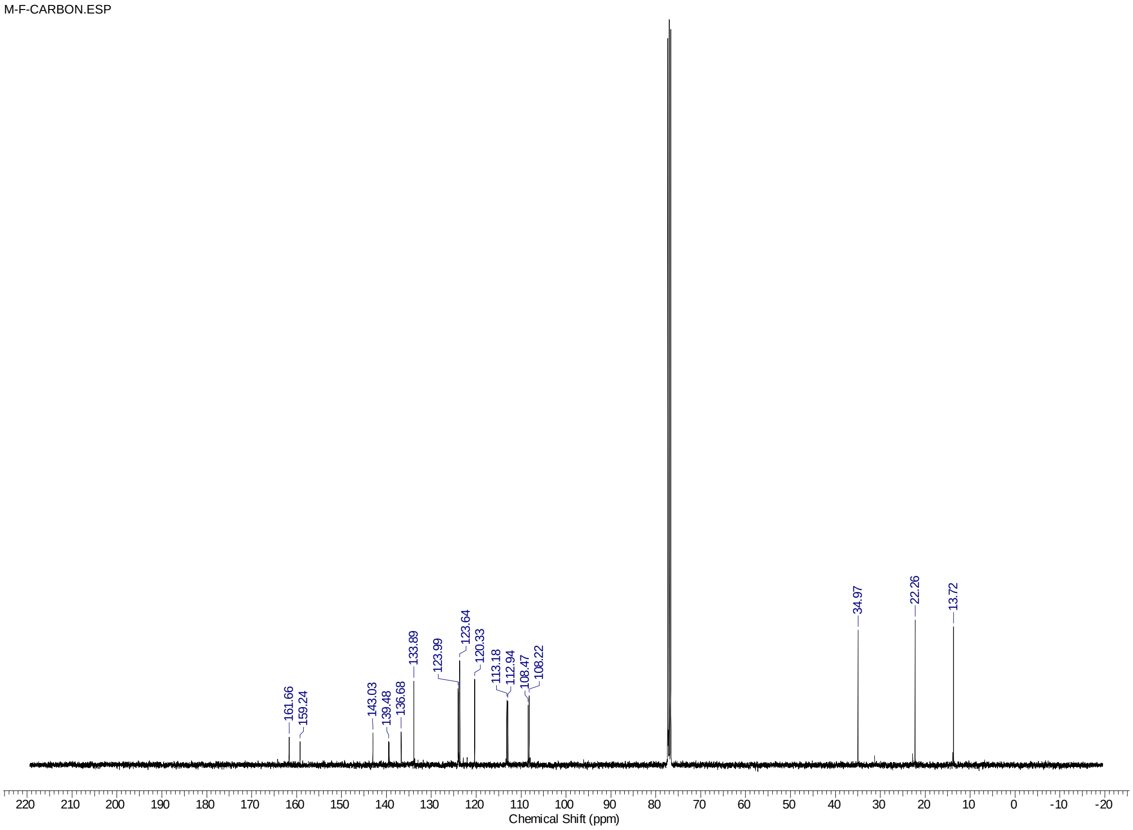

(E)-2-(Pent-1-en-1-yl)naphtho[2,1-b]thiophene S24

^{1}H NMR (400 MHz, $CDCl_3$)

20151102-1555-B400_B.11-29.010.001.1R.esp

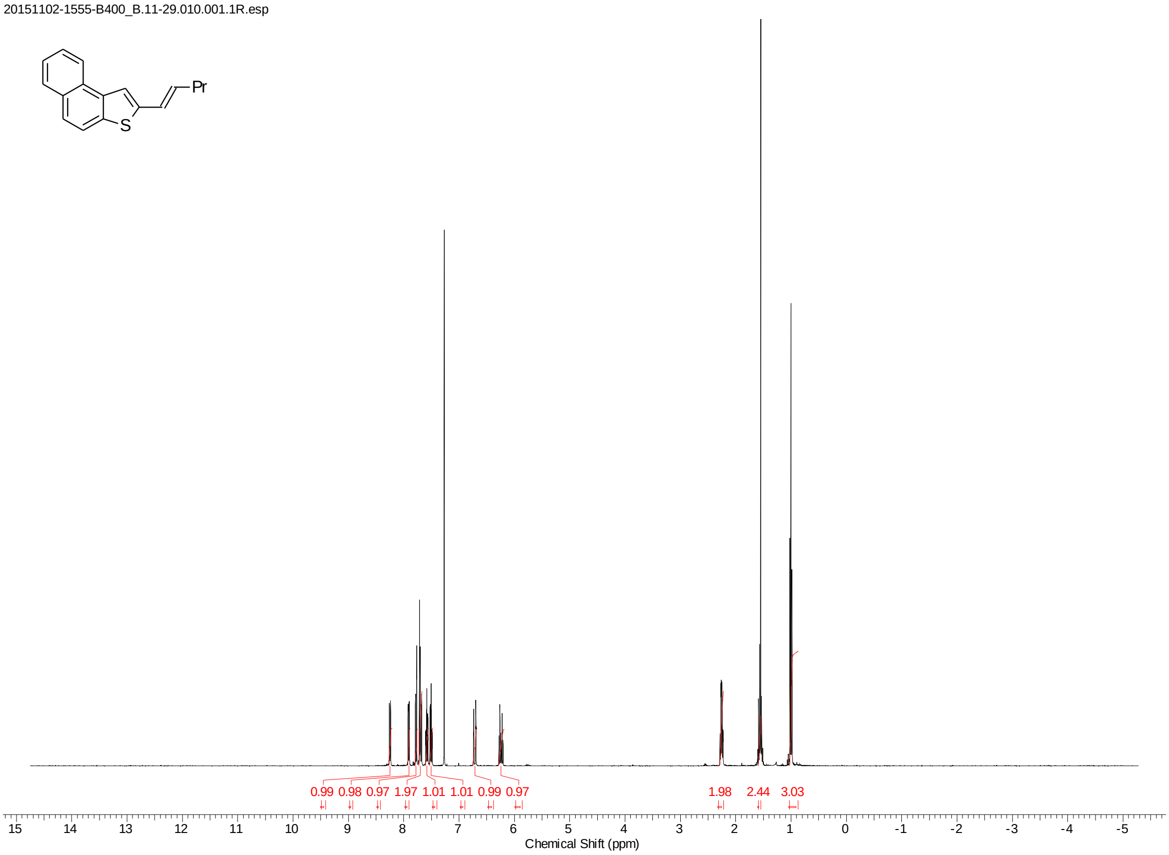

^{13}C NMR (100 MHz, $CDCl_3$)

NAPHTH-CARBON.ESP

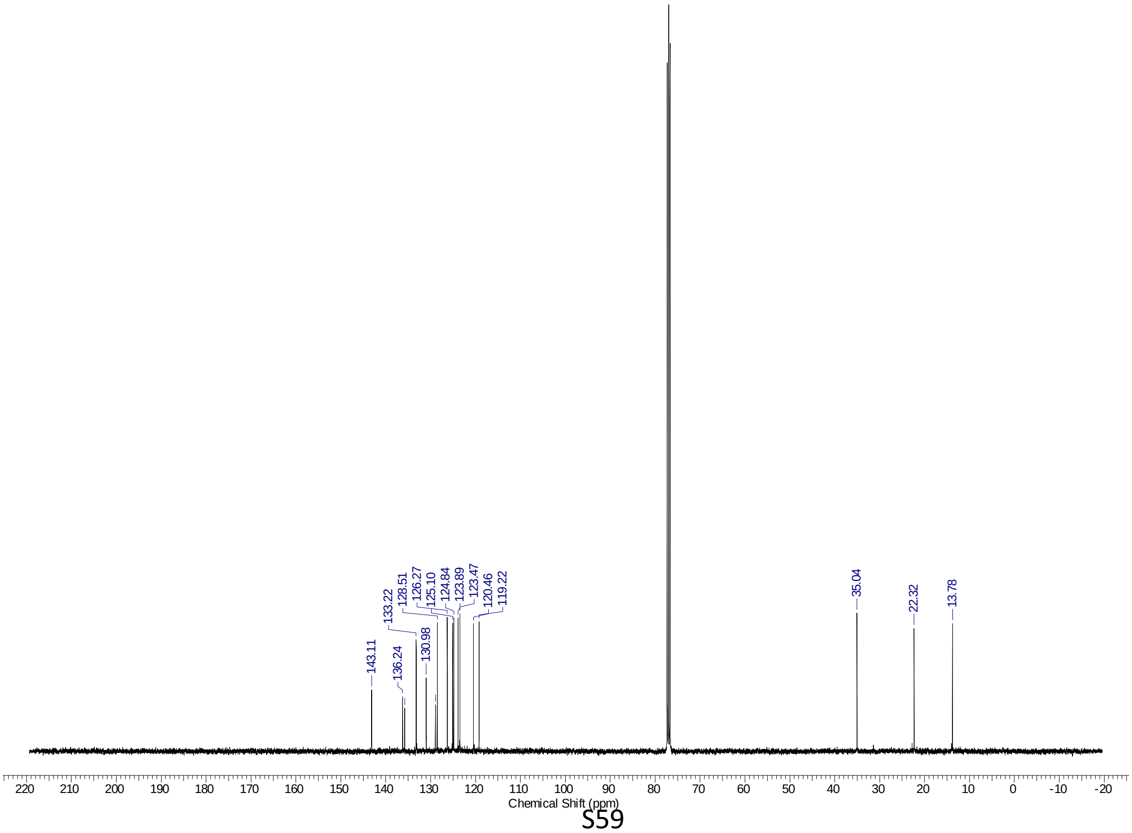

(E)-5-Methoxy-2-(pent-1-en-1-yl)benzo[b]thiophene S25

^{1}H NMR (400 MHz, $CDCl_3$)

20151102-1334-B400_B.12-21.010.001.1R.esp

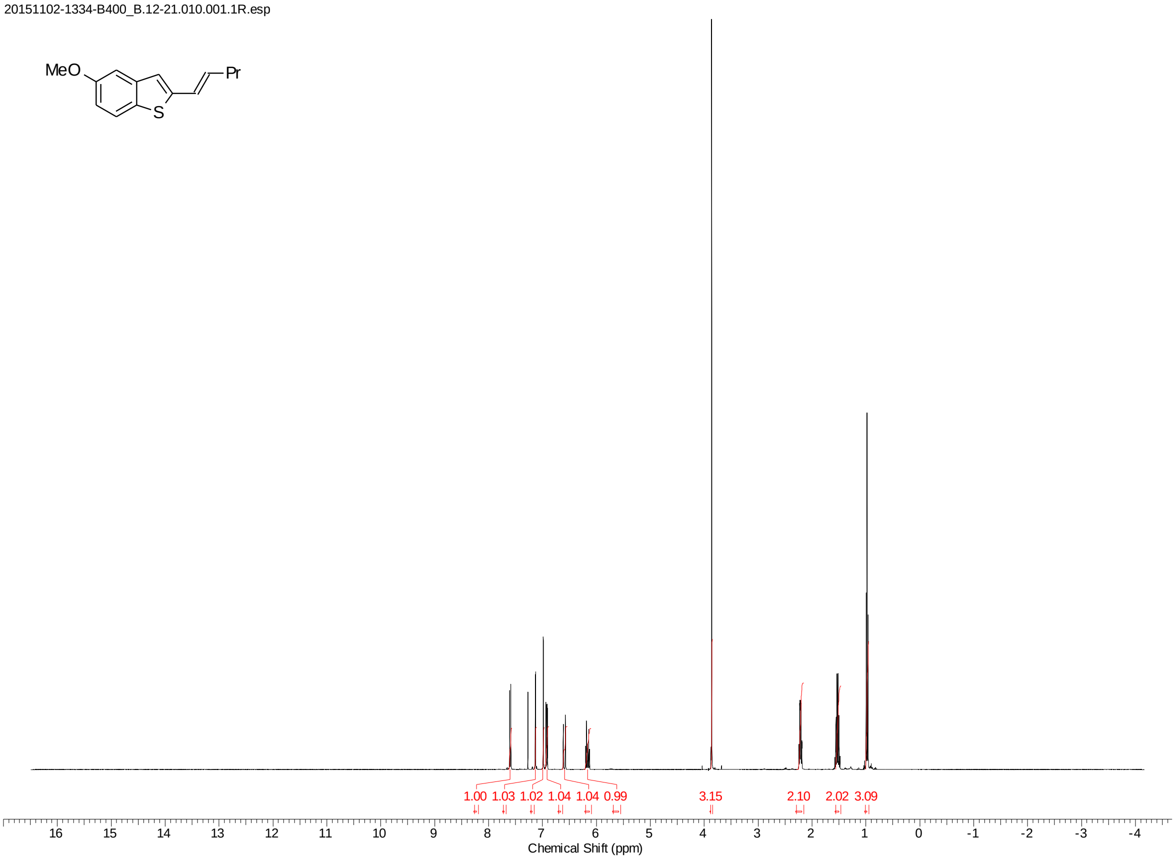

^{13}C NMR (100 MHz, $CDCl_3$)

HJS059-OME02 CARBON.ESP

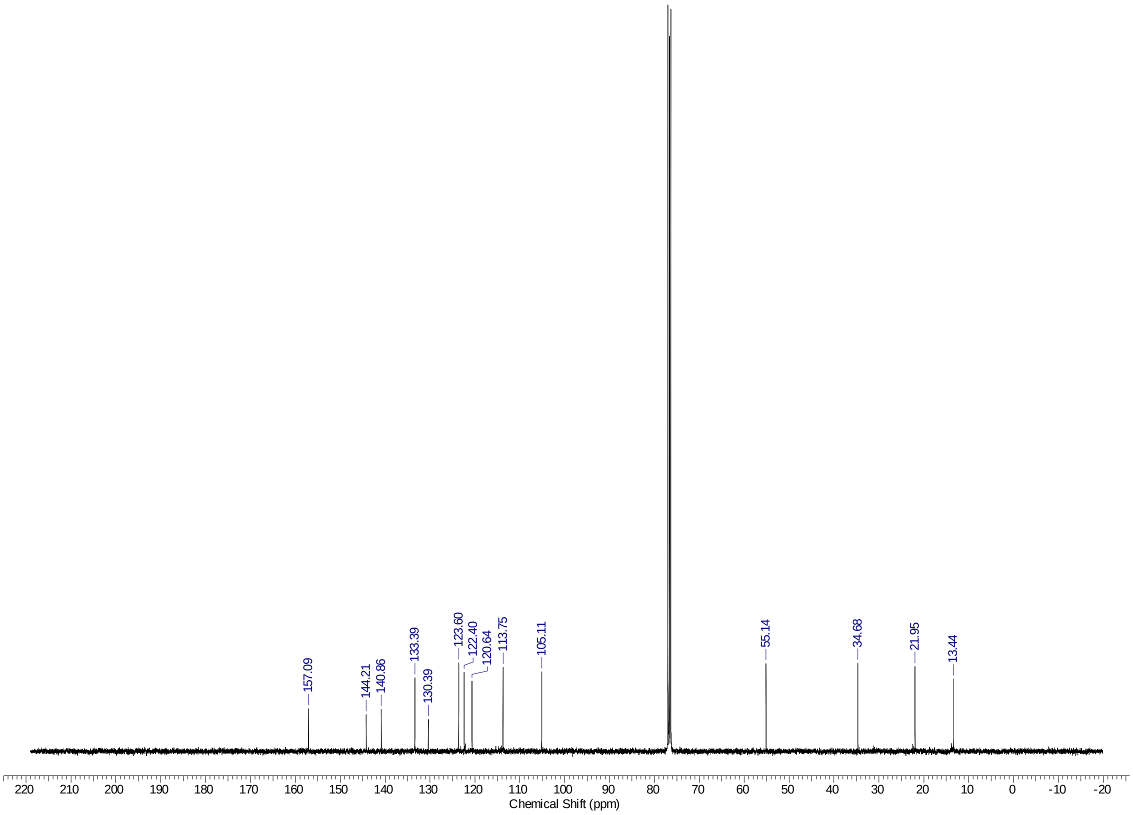

(E)-5-Chloro-2-(pent-1-en-1-yl)benzo[b]thiophene S26

^{1}H NMR (400 MHz, $CDCl_3$)

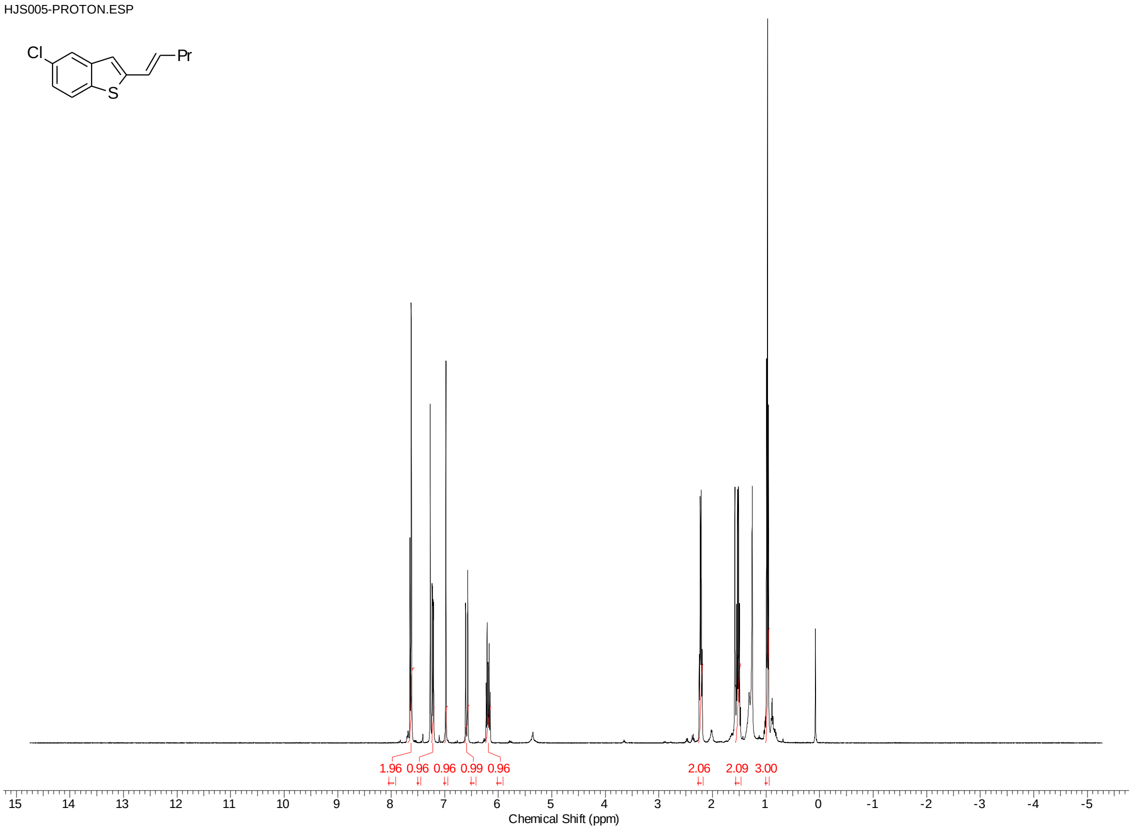

^{13}C NMR (100 MHz, $CDCl_3$)

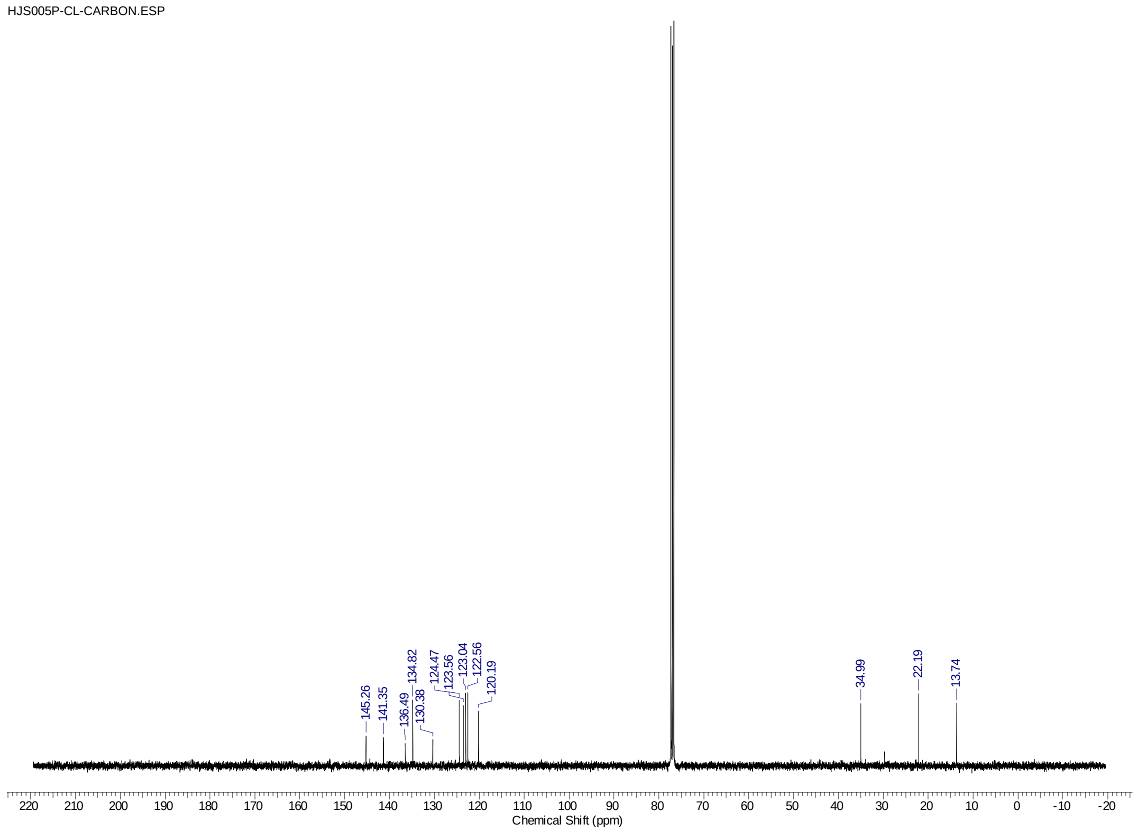

(E)-4,6-Dimethyl-2-(pent-1-en-1-yl)benzo[b]thiophene S27

^{1}H NMR (400 MHz, $CDCl_3$)

3,5-DIMETHYL-PROTON.ESP

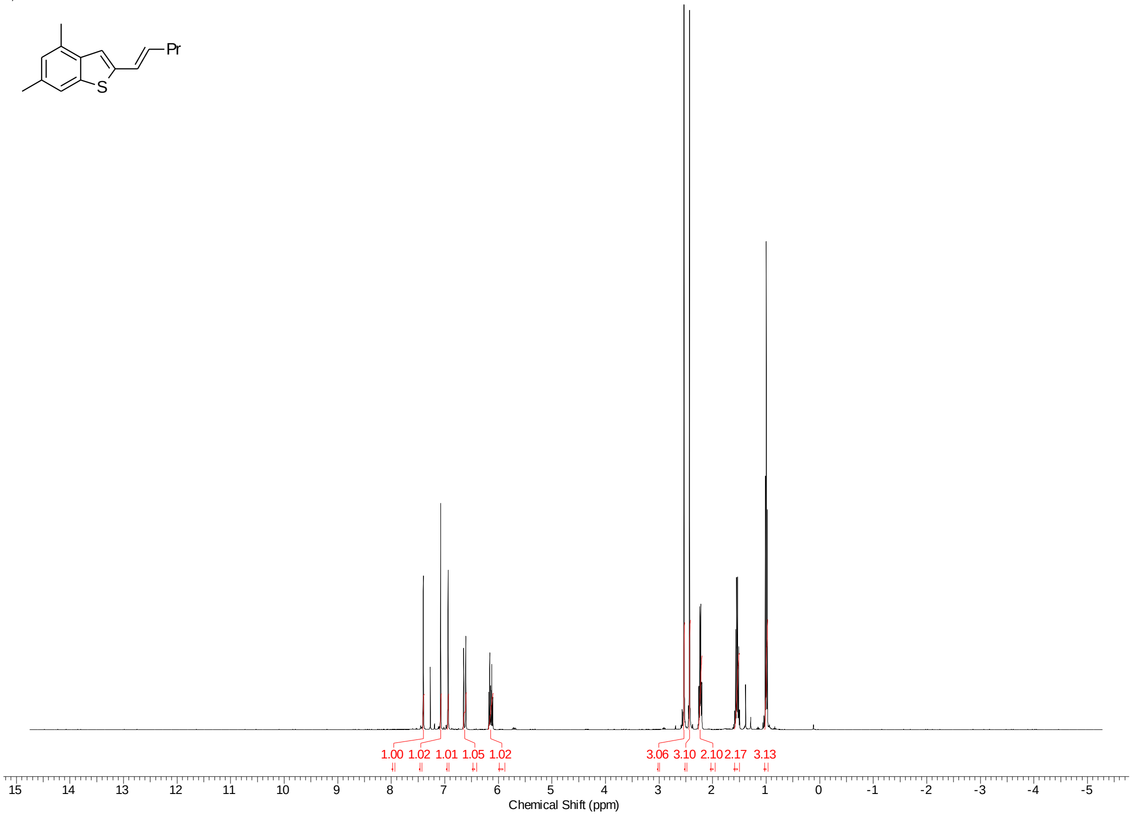

^{13}C NMR (100 MHz, $CDCl_3$)

3,5-DIMETHYL-CARBON.ESP

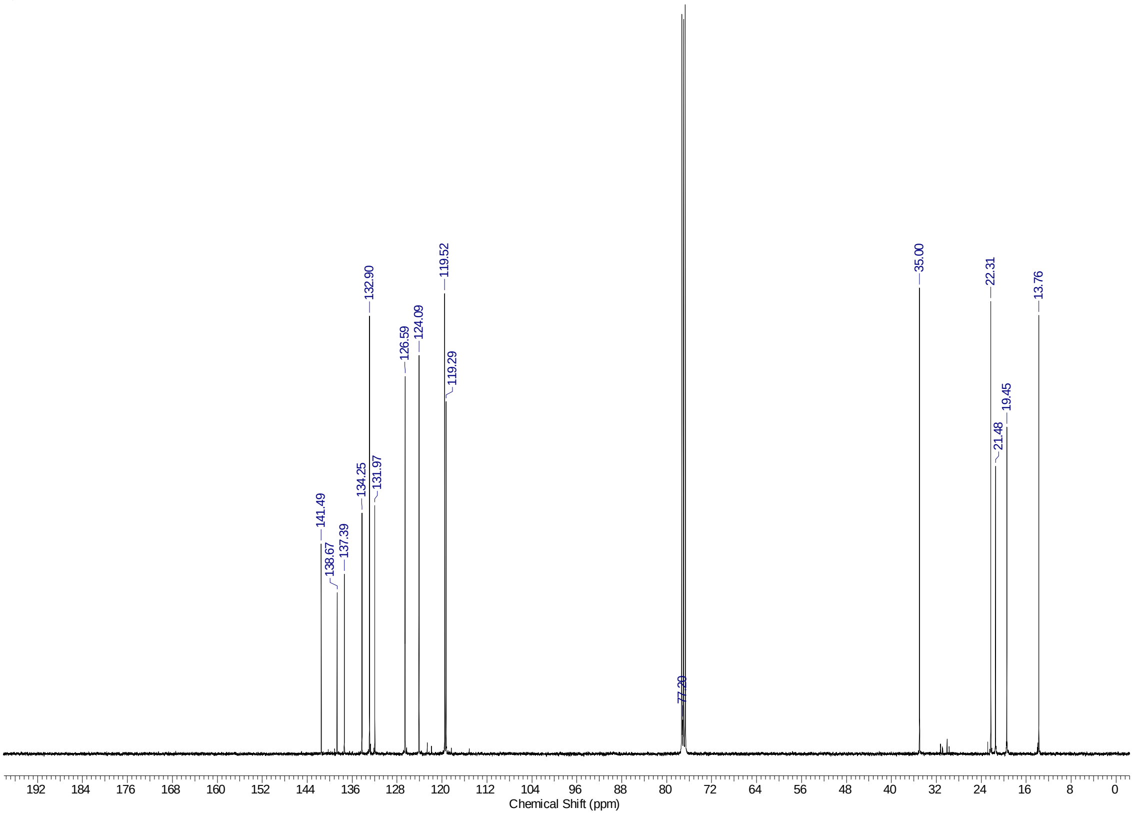

2,6-bis(Hexylsulfinyl)naphthalene 7

^{1}H NMR (400 MHz, $CDCl_3$)

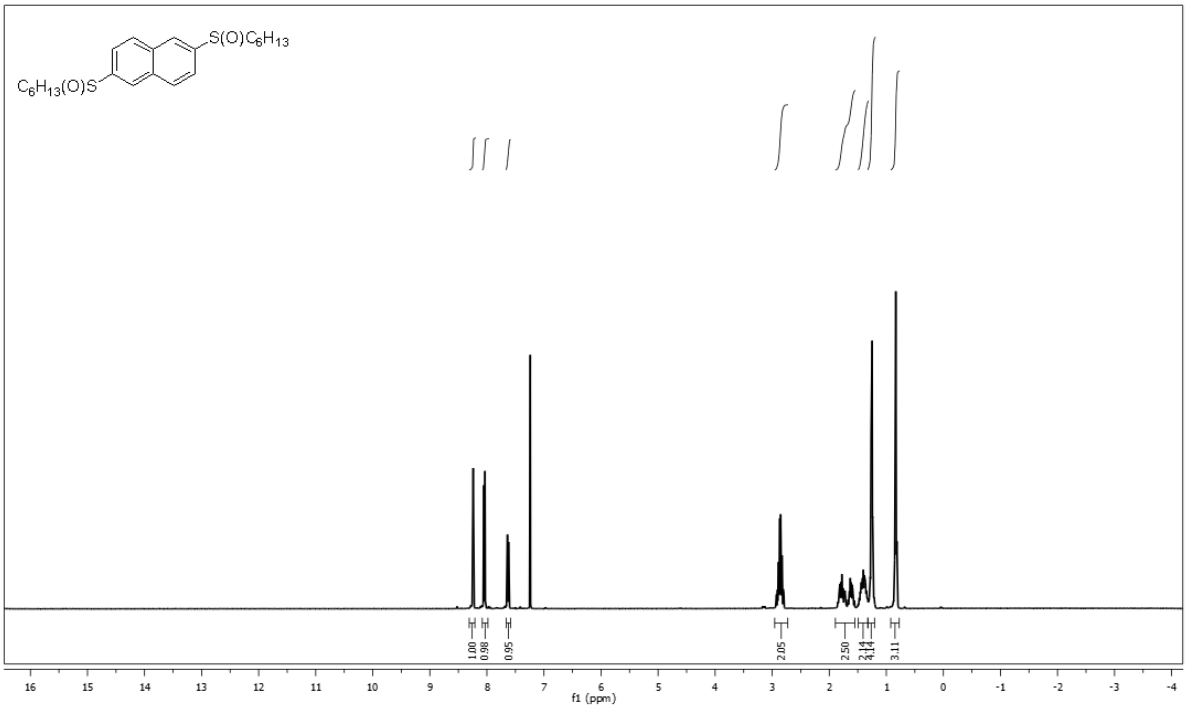

^{13}C NMR (100 MHz, $CDCl_3$)

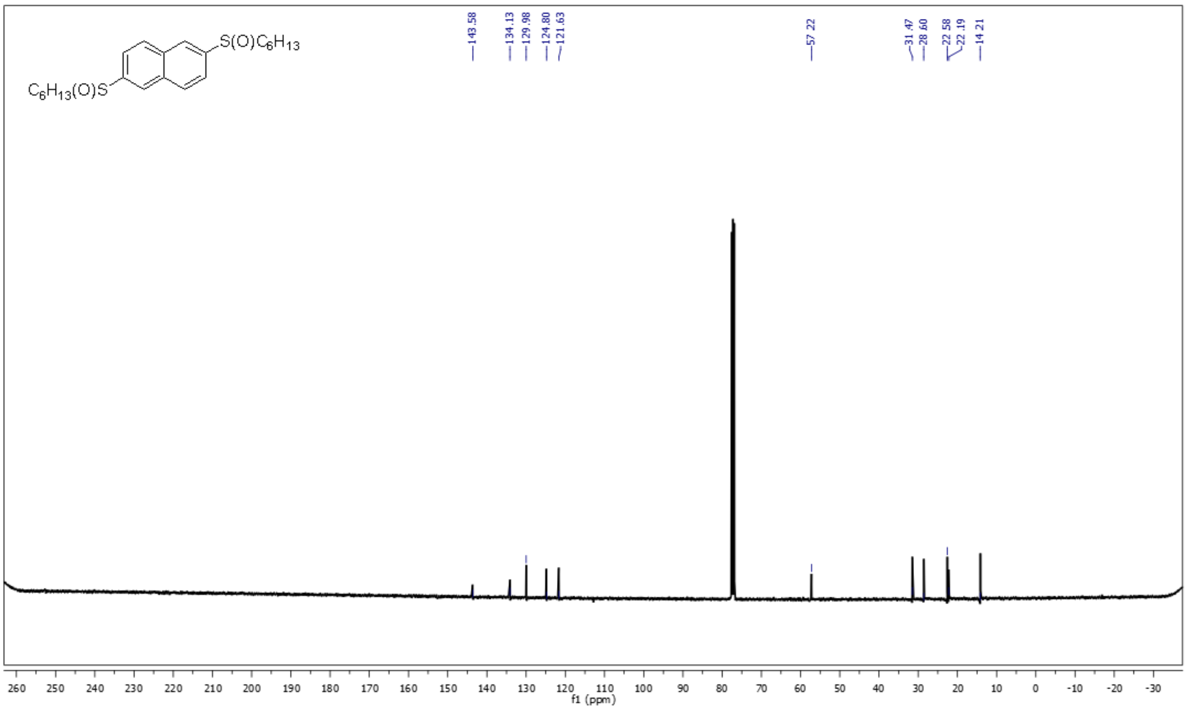

1,5-bis(Hexylsulfinyl)naphthalene 8

^{1}H NMR (500 MHz, $CDCl_3$)

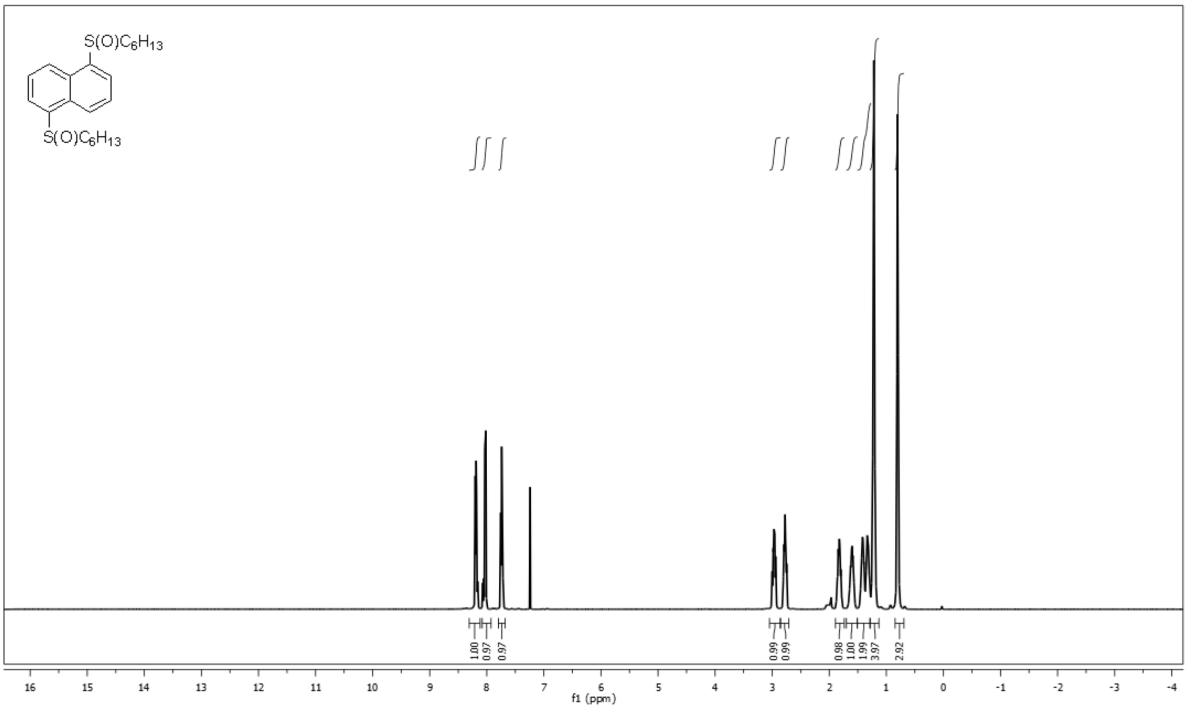

^{13}C NMR (125 MHz, $CDCl_3$)

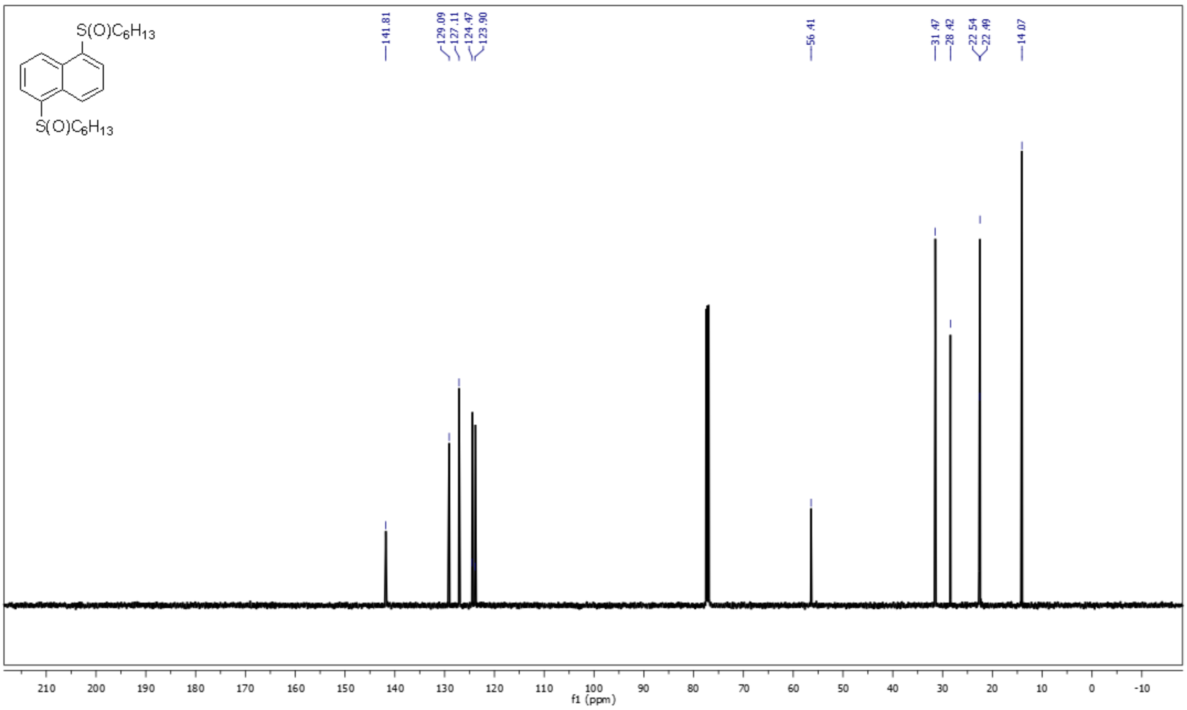

(2,6-Di(non-2-yn-1-yl)naphthalene-1,5-diyl)bis(hexylsulfide) 10b

^{1}H NMR (500 MHz, $CDCl_3$)

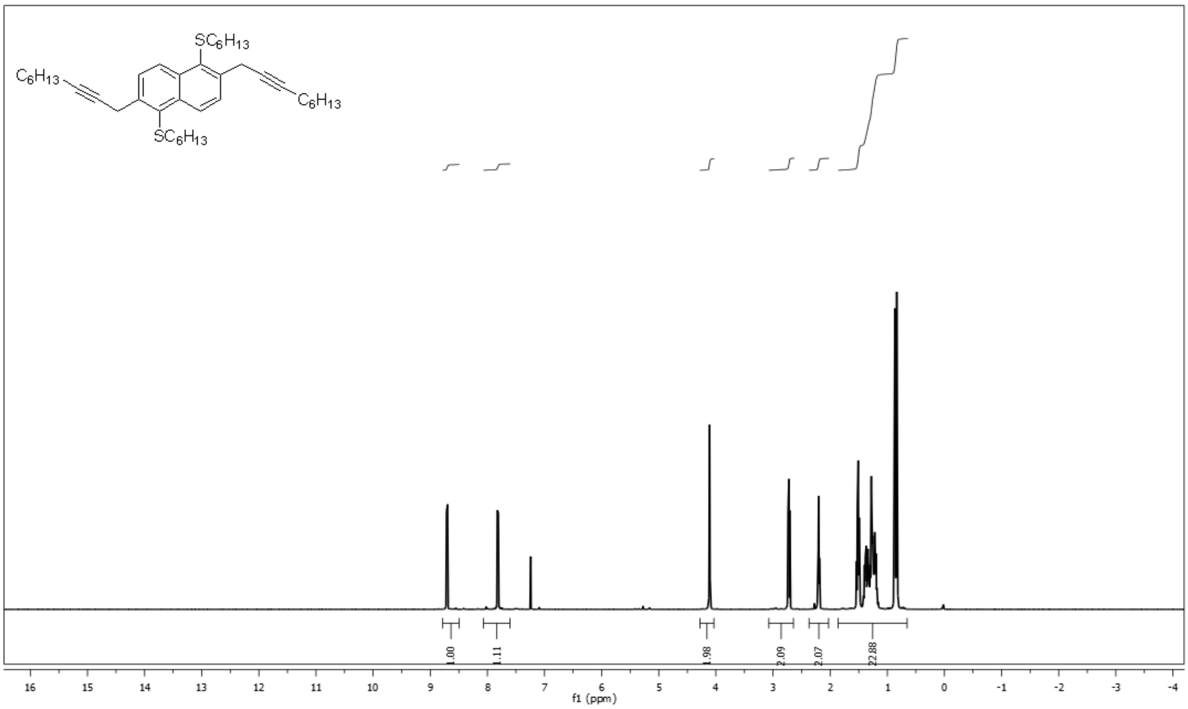

^{13}C NMR (125 MHz, $CDCl_3$)

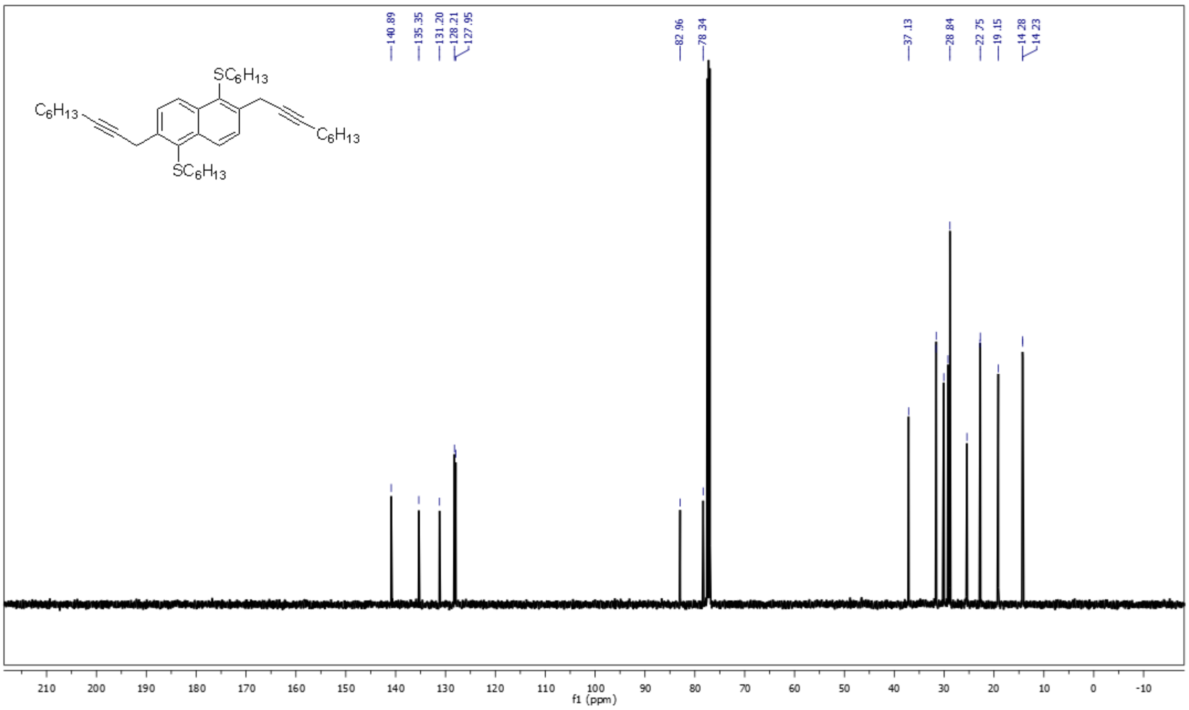

(1,5-Di(non-2-yn-1-yl)naphthalene-2,6-diyl)bis(hexylsulfide) 9

^{1}H NMR (500 MHz, $CDCl_3$)

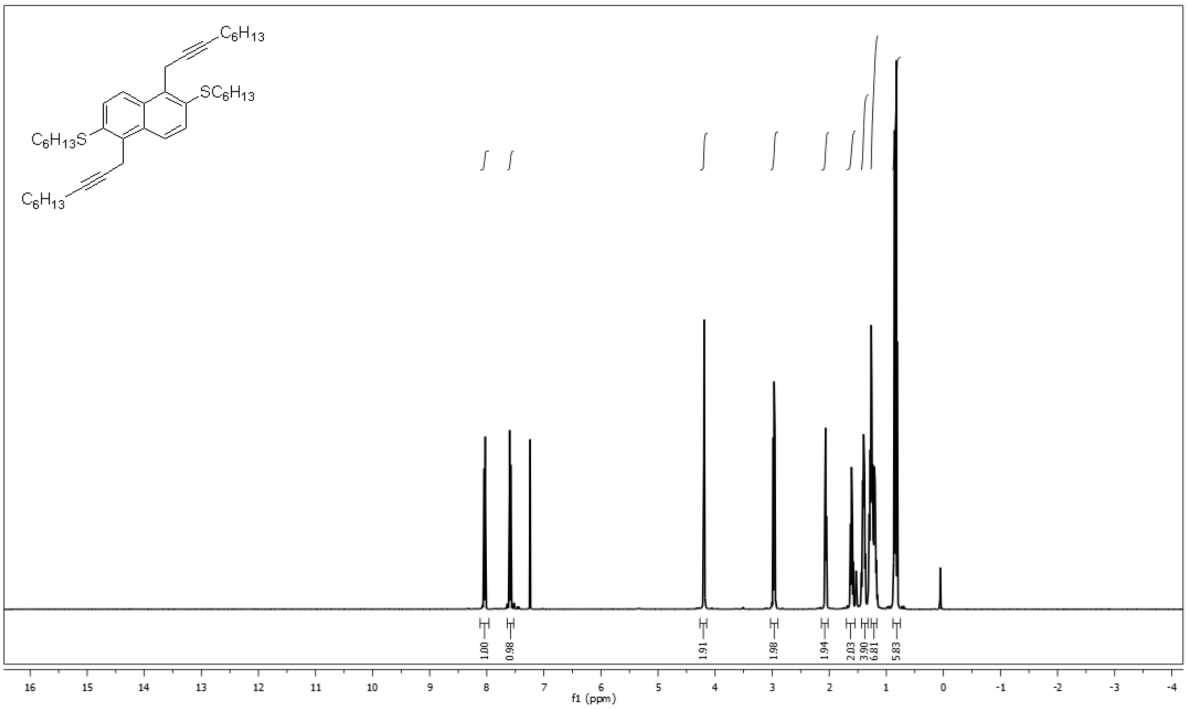

^{13}C NMR (125 MHz, $CDCl_3$)

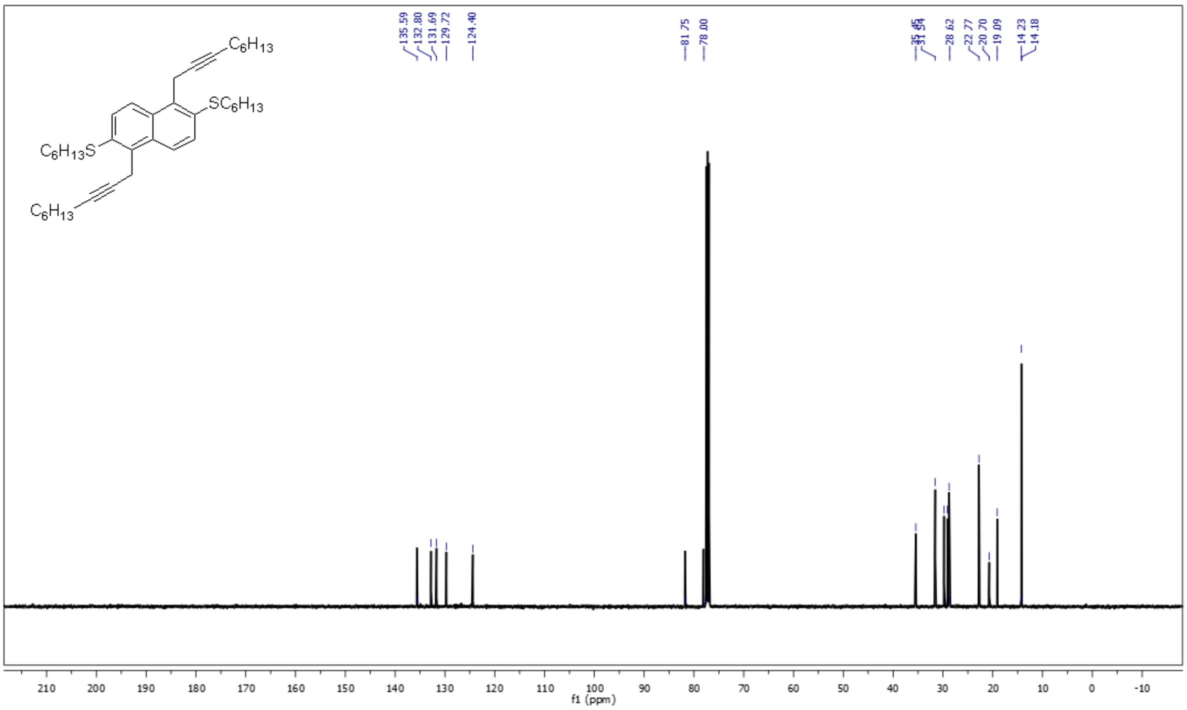

2,7-Diheptylnaphtho[1,2-*b*:5,6-*b*']dithiophene 12

^{1}H NMR (400 MHz, C_6D_6)

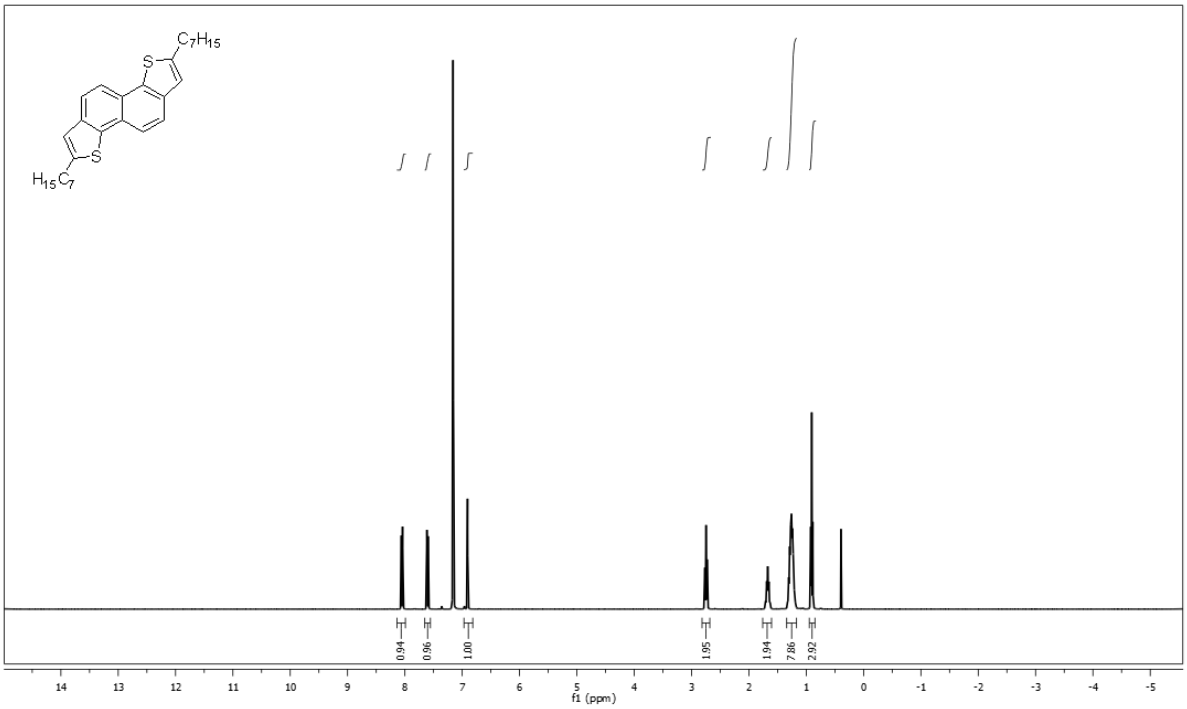

^{13}C NMR (100 MHz, C_6D_6)

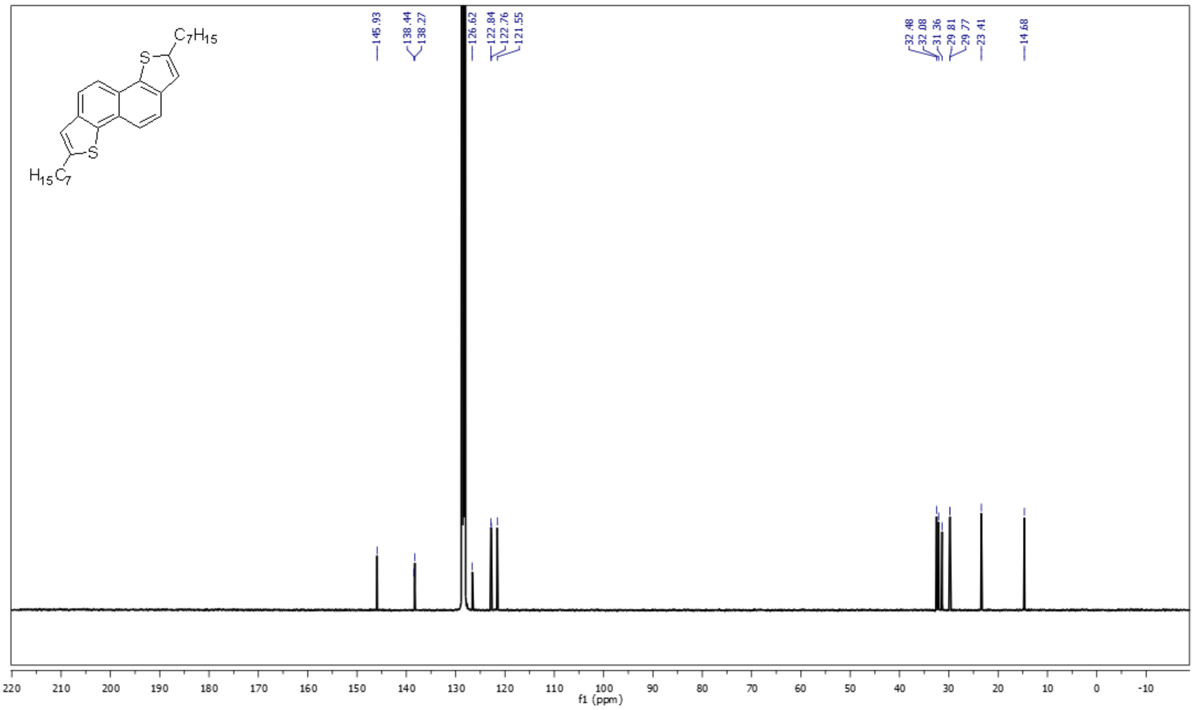

2,7-Diheptylnaphtho[2,1-*b*:6,5-*b*']dithiophene 11

^{1}H NMR (400 MHz, C_6D_6)

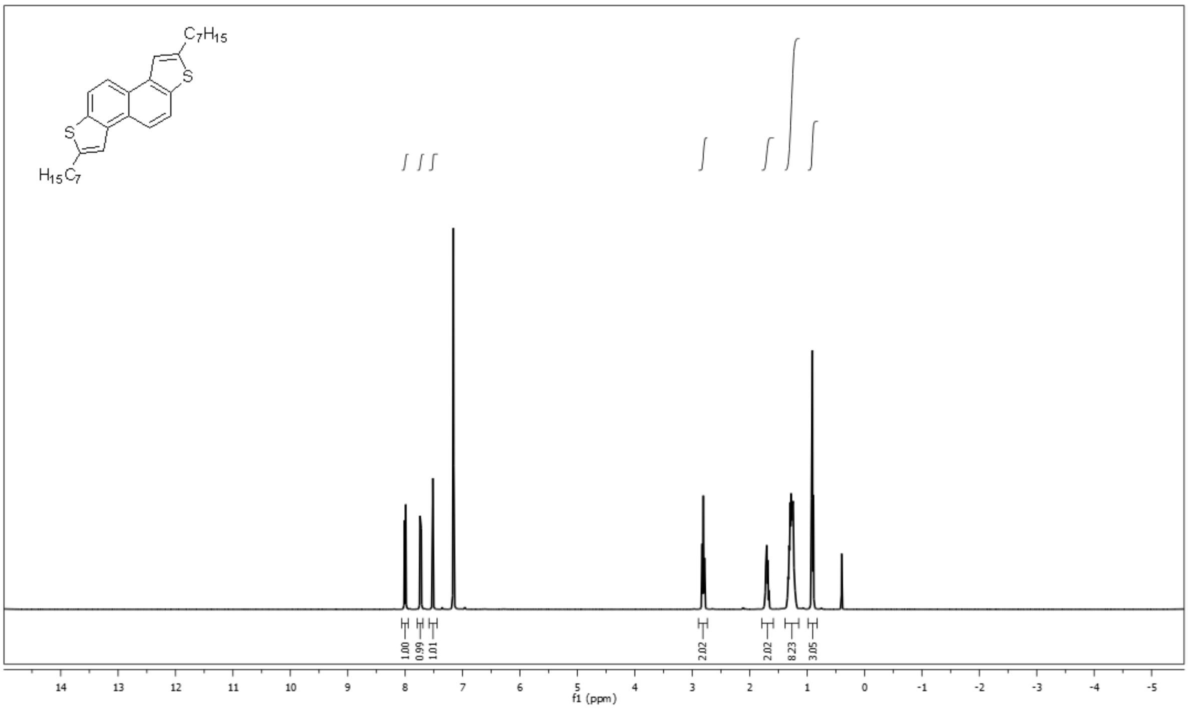

^{13}C NMR (100 MHz, C_6D_6)

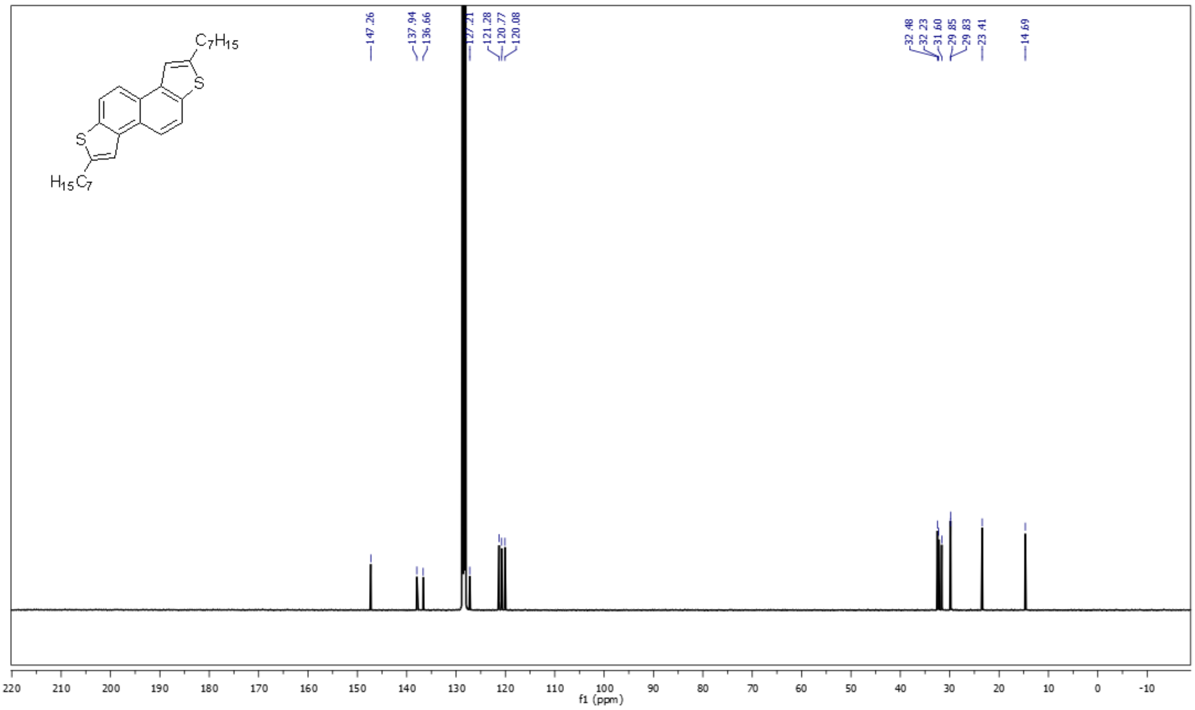

1,1'-(Naphtho[1,2-*b*:5,6-*b*']dithiophene-2,7-diyl)bis(heptan-1-one) 14

^{1}H NMR (400 MHz, C_6D_6)

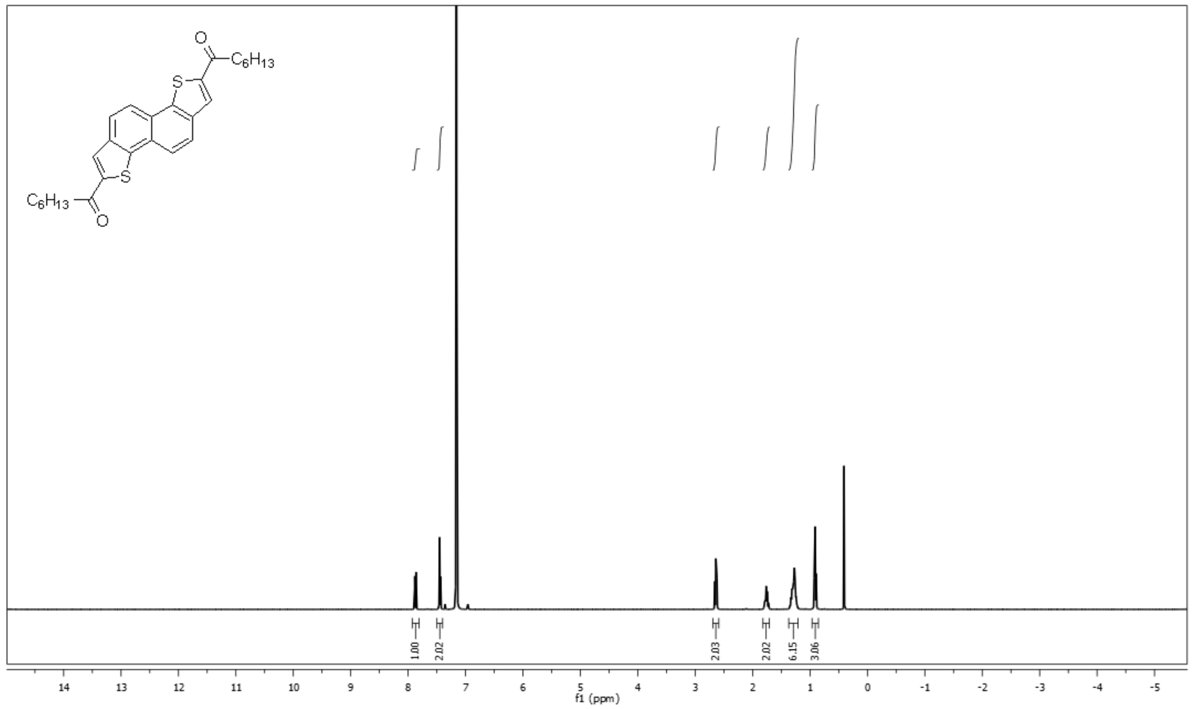

^{13}C NMR (100 MHz, C_6D_6)

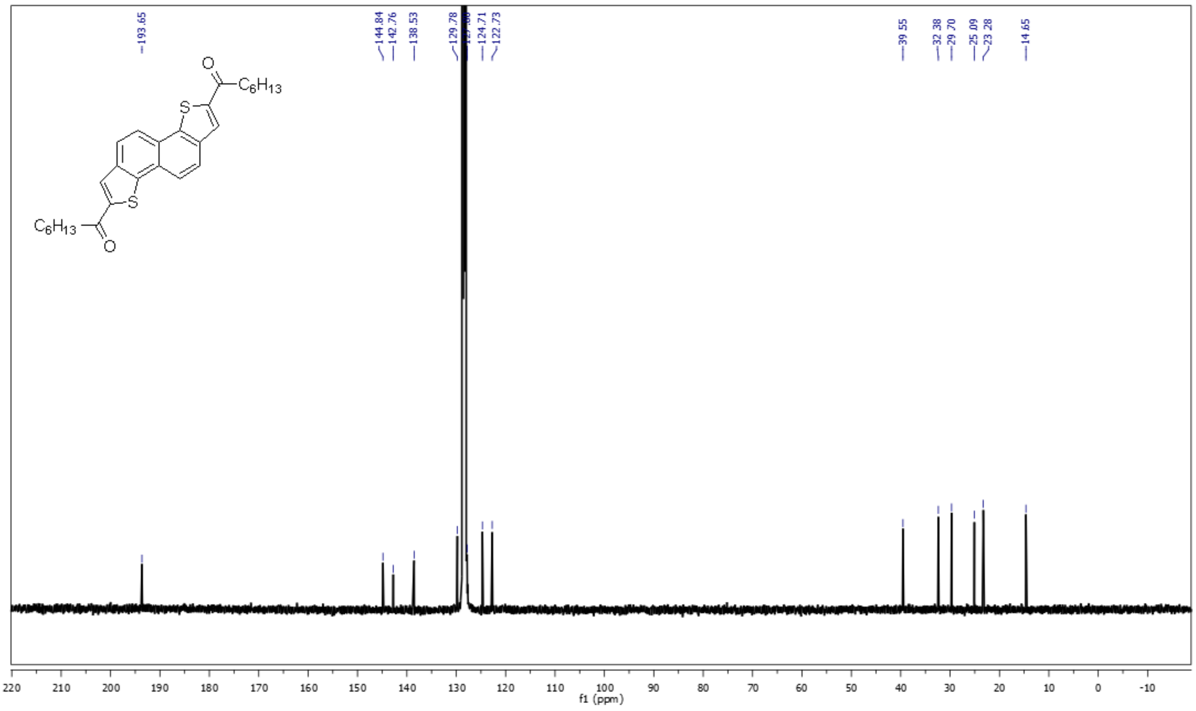

1,1'-(Naphtho[2,1-*b*:6,5-*b*']dithiophene-2,7-diyl)bis(heptan-1-one) 13

^{1}H NMR (400 MHz, C_6D_6)

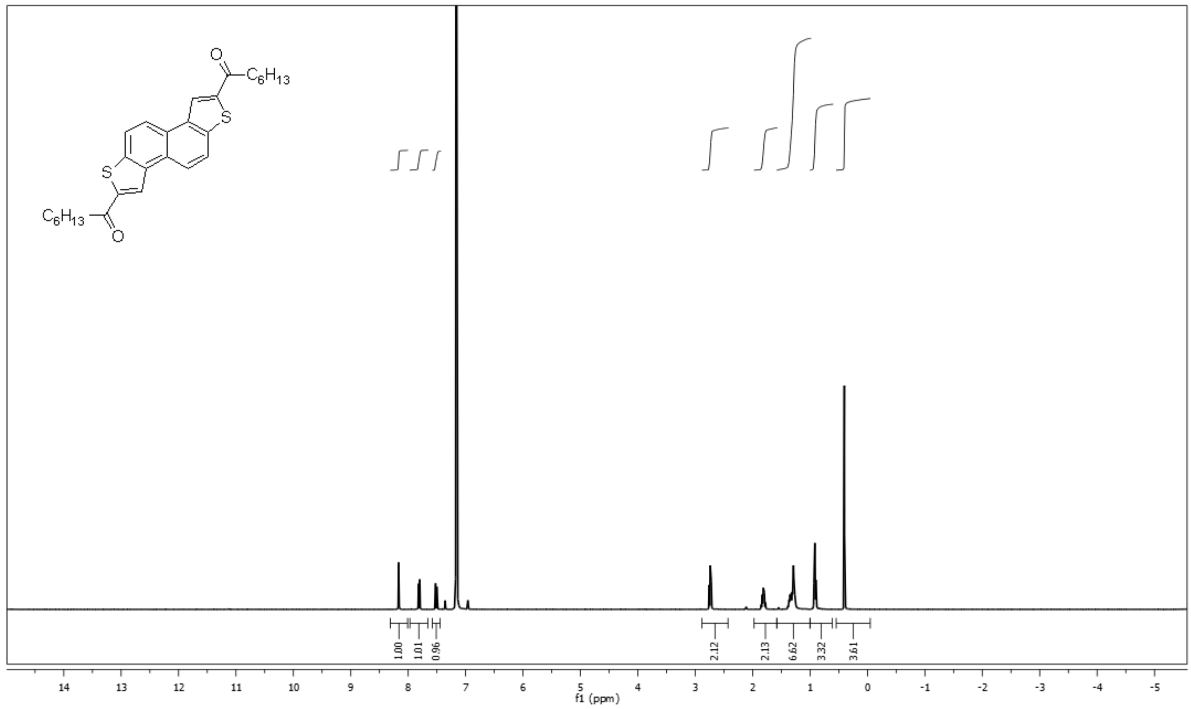

^{13}C NMR (100 MHz, C_6D_6)

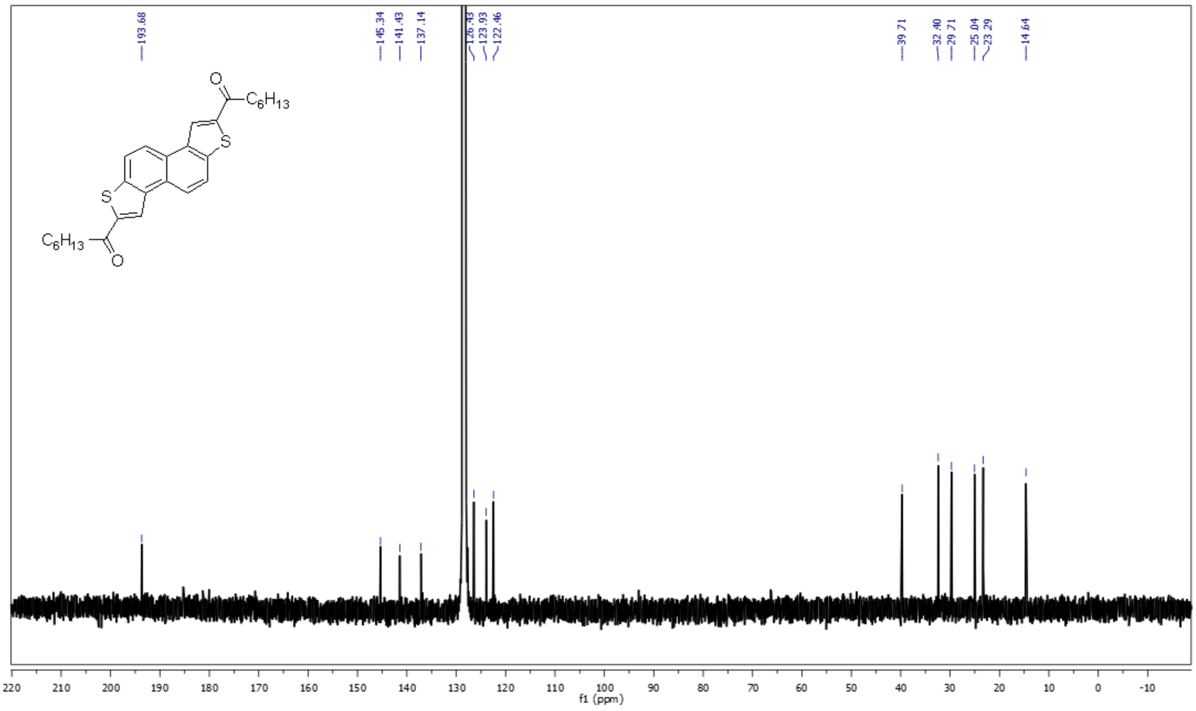

2,7-Di((E)-hept-1-en-1-yl)naphtho[2,1-b:6,5-b']dithiophene 15

^{1}H NMR (400 MHz, $CDCl_3$)

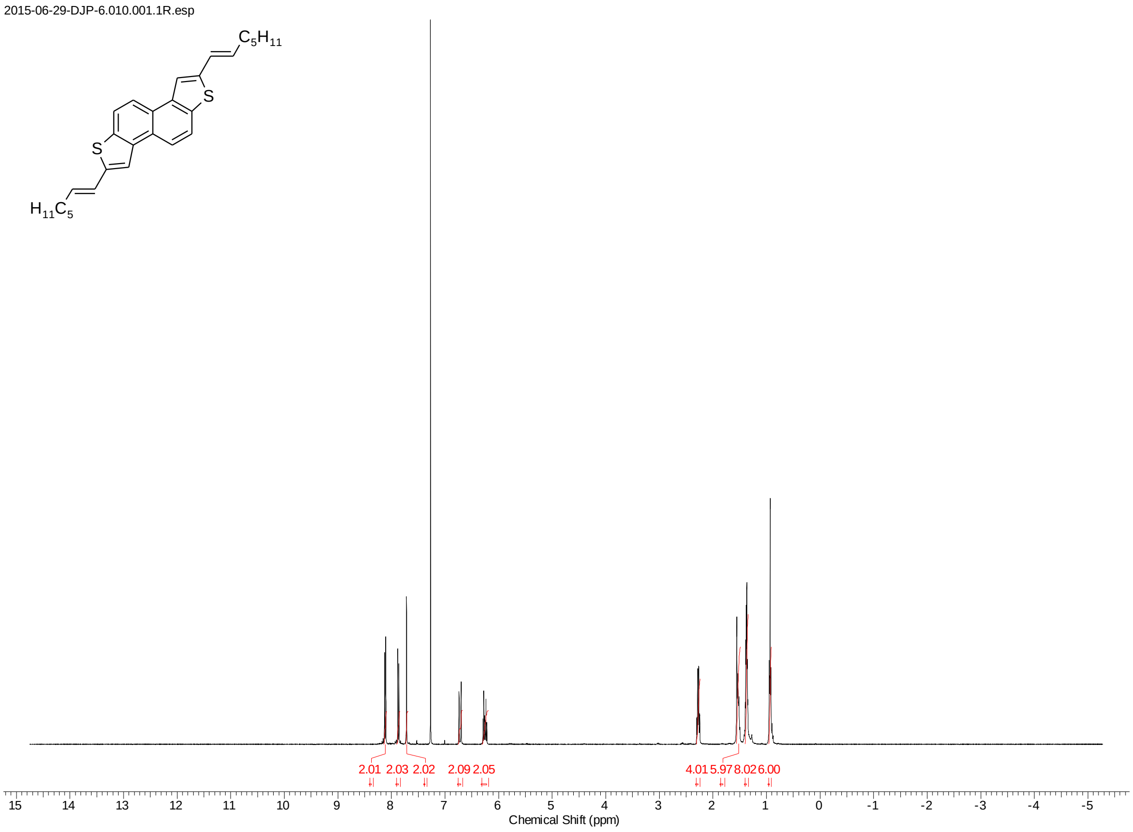

^{13}C NMR (100 MHz, $CDCl_3$)

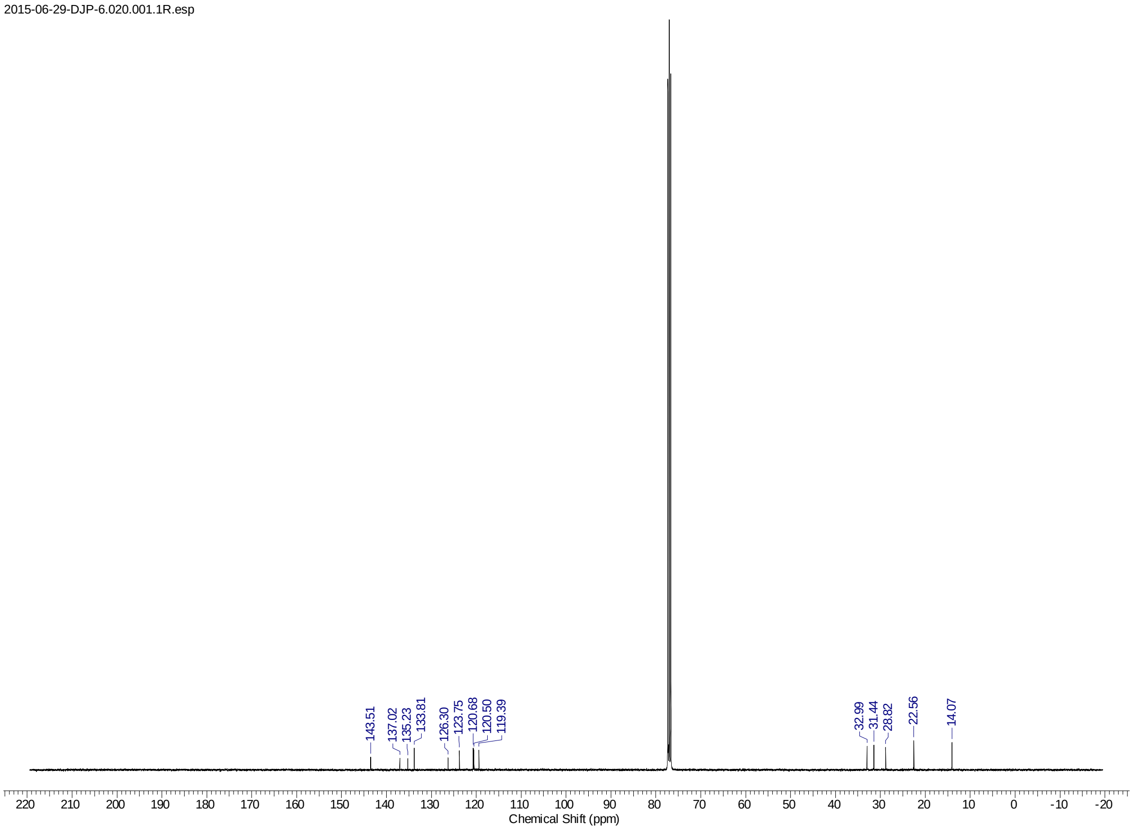

2,7-Di((E)-hept-1-en-1-yl)naphtho[1,2-b:5,6-b']dithiophene 16

^{1}H NMR (400 MHz, $CDCl_3$)

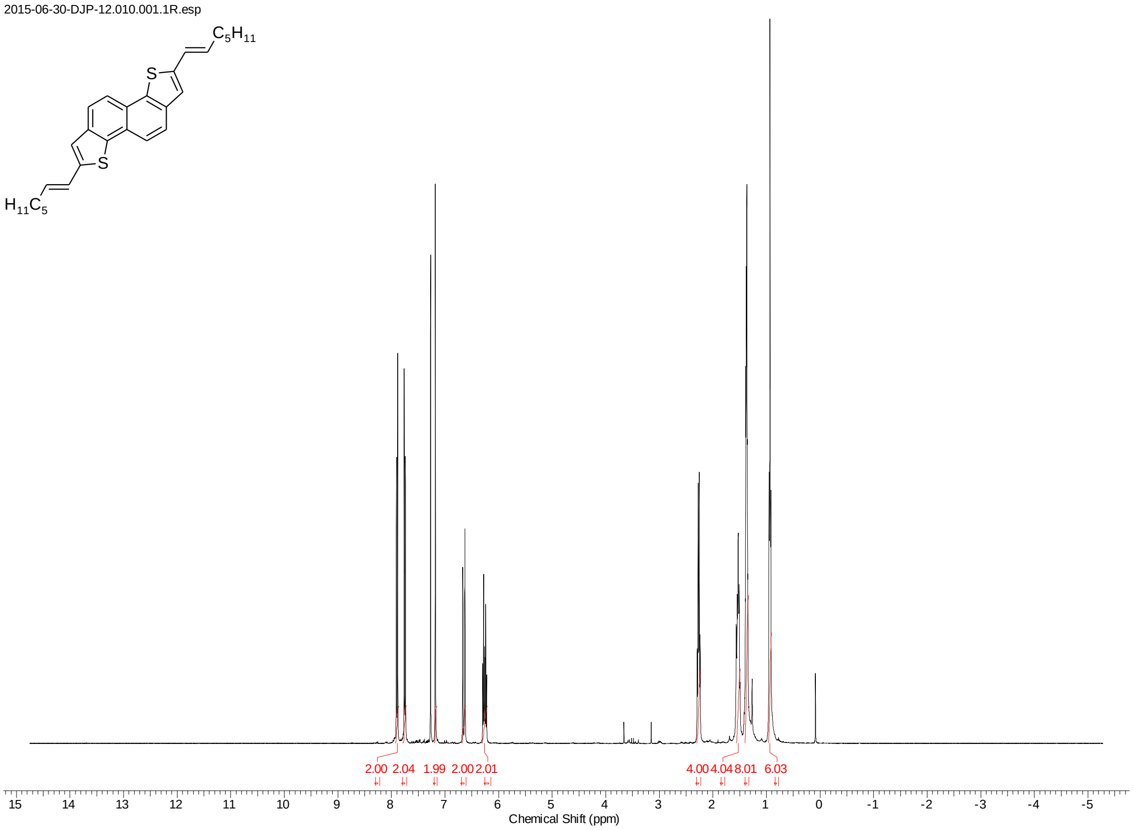

^{13}C NMR (100 MHz, $CDCl_3$)

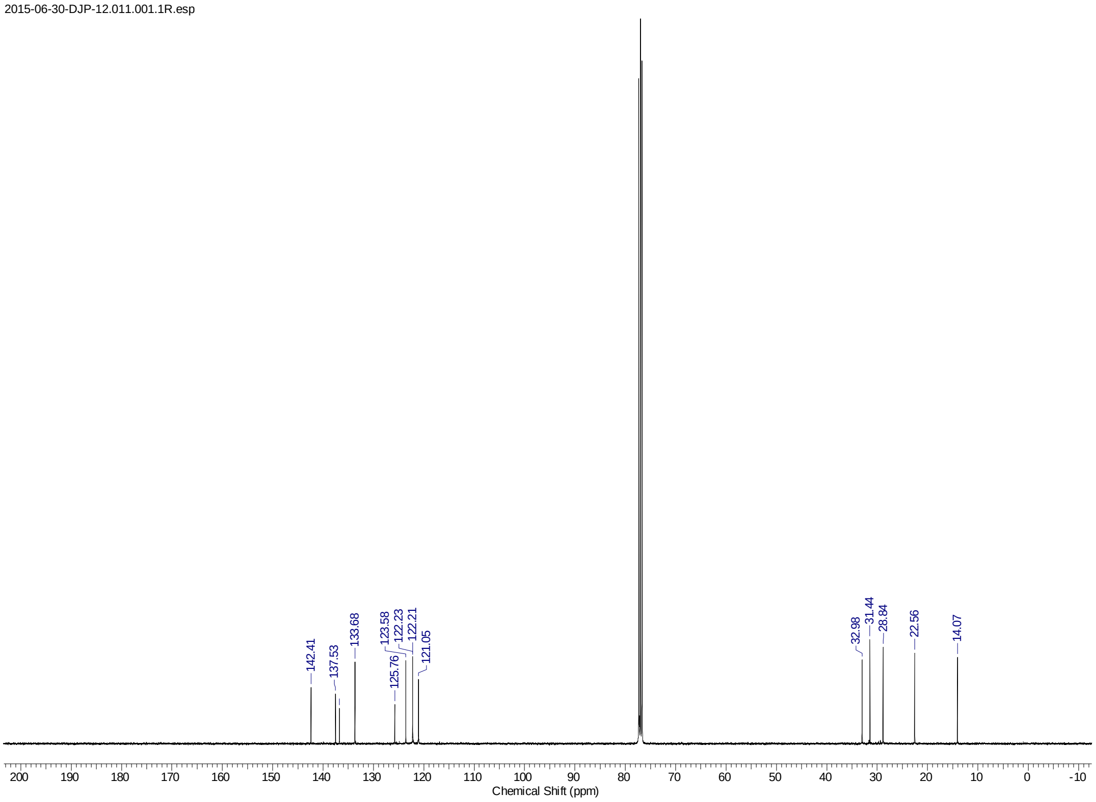

2-(Hept-2-yn-1-yl)-1,4-phenylene)*bis*(methylsulfide) S28

^{1}H NMR (400 MHz, $CDCl_3$)

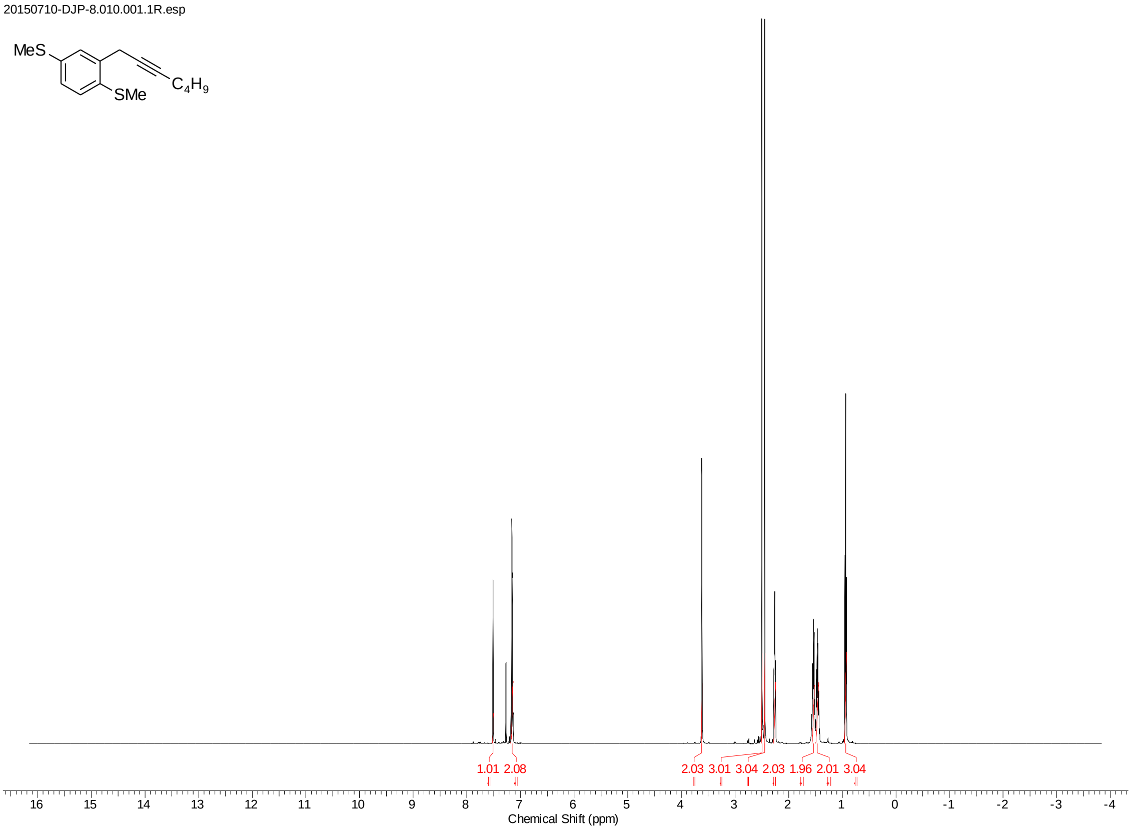

^{13}C NMR (100 MHz, $CDCl_3$)

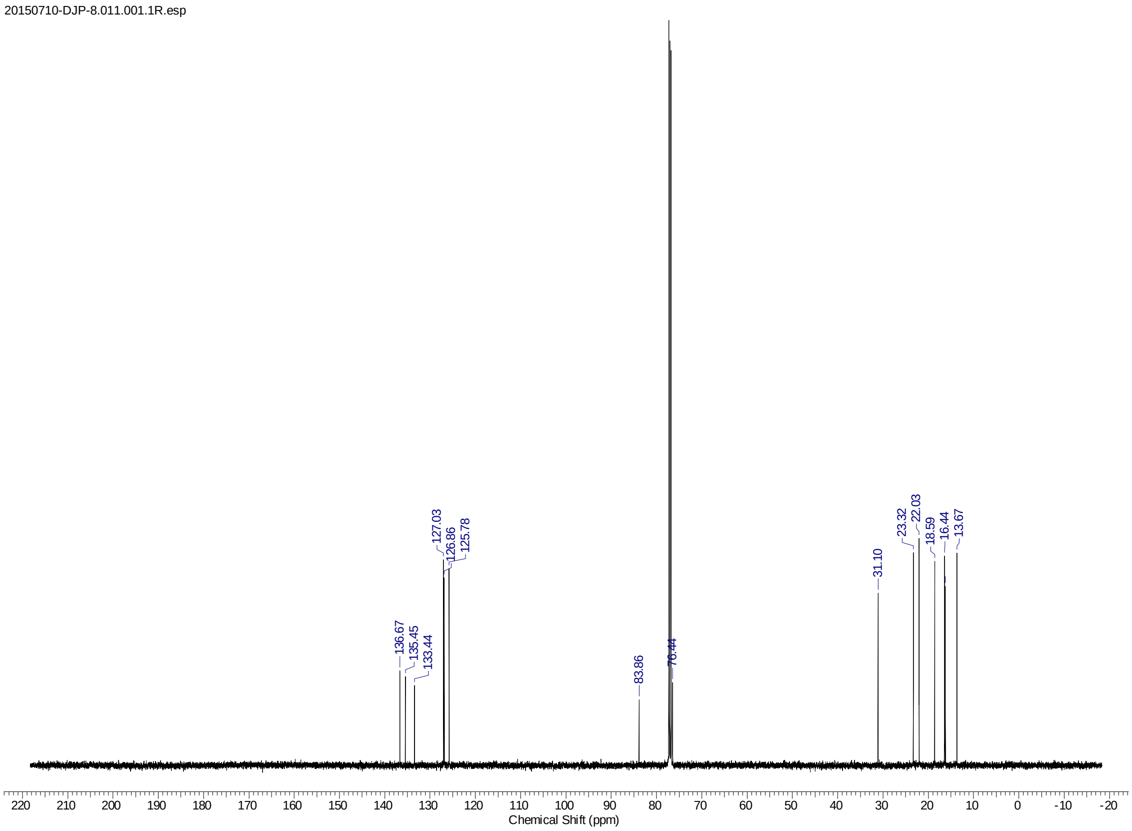

5-(Methylsulfanyl)-2-pentylbenzo[b]thiophene 19

^{1}H NMR (400 MHz, $CDCl_3$)

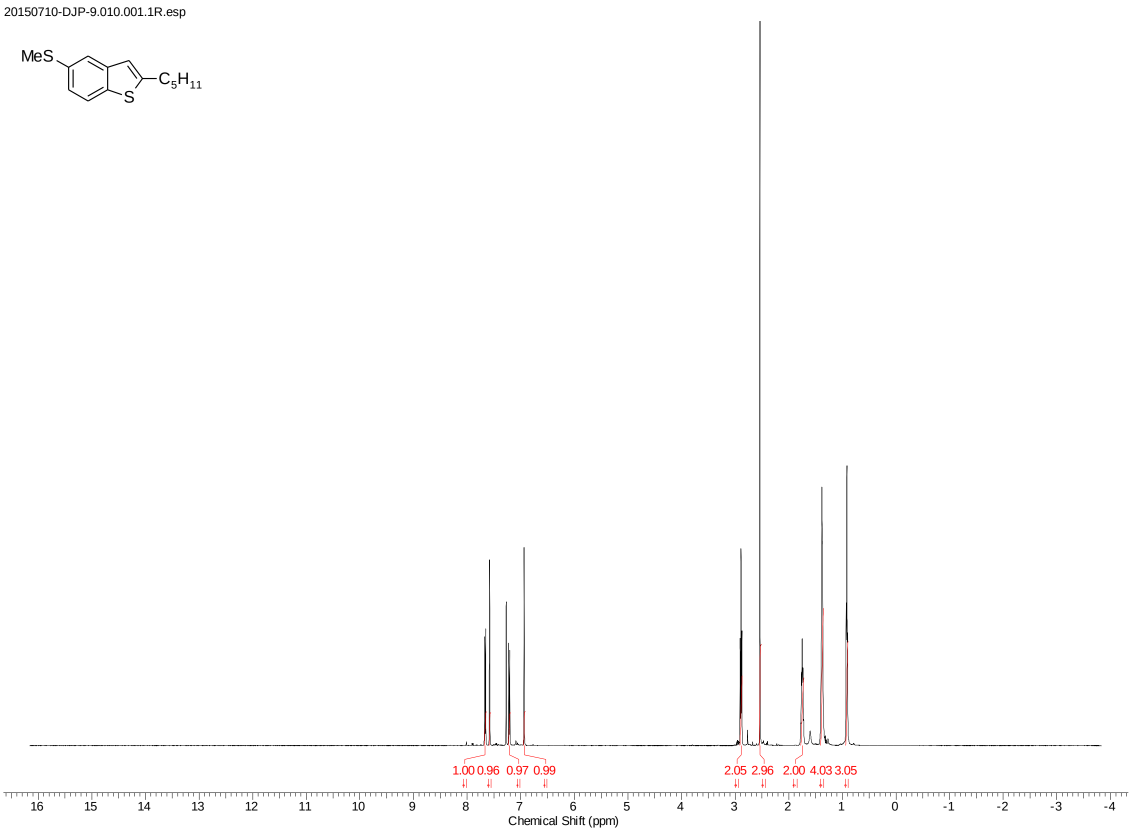

^{13}C NMR (100 MHz, $CDCl_3$)

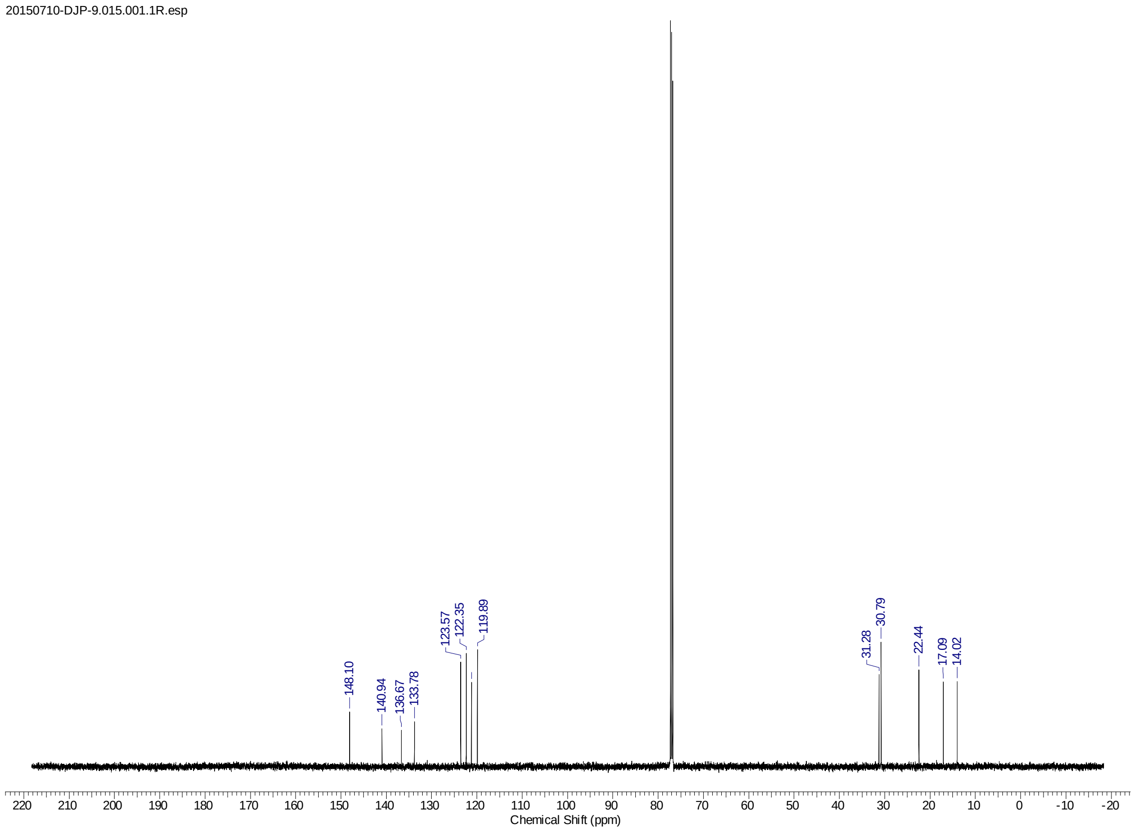

5-(Methylsulfinyl)-2-pentylbenzo[b]thiophene 20

^{1}H NMR (400 MHz, $CDCl_3$)

20150707-DJP-7.010.001.1R.esp

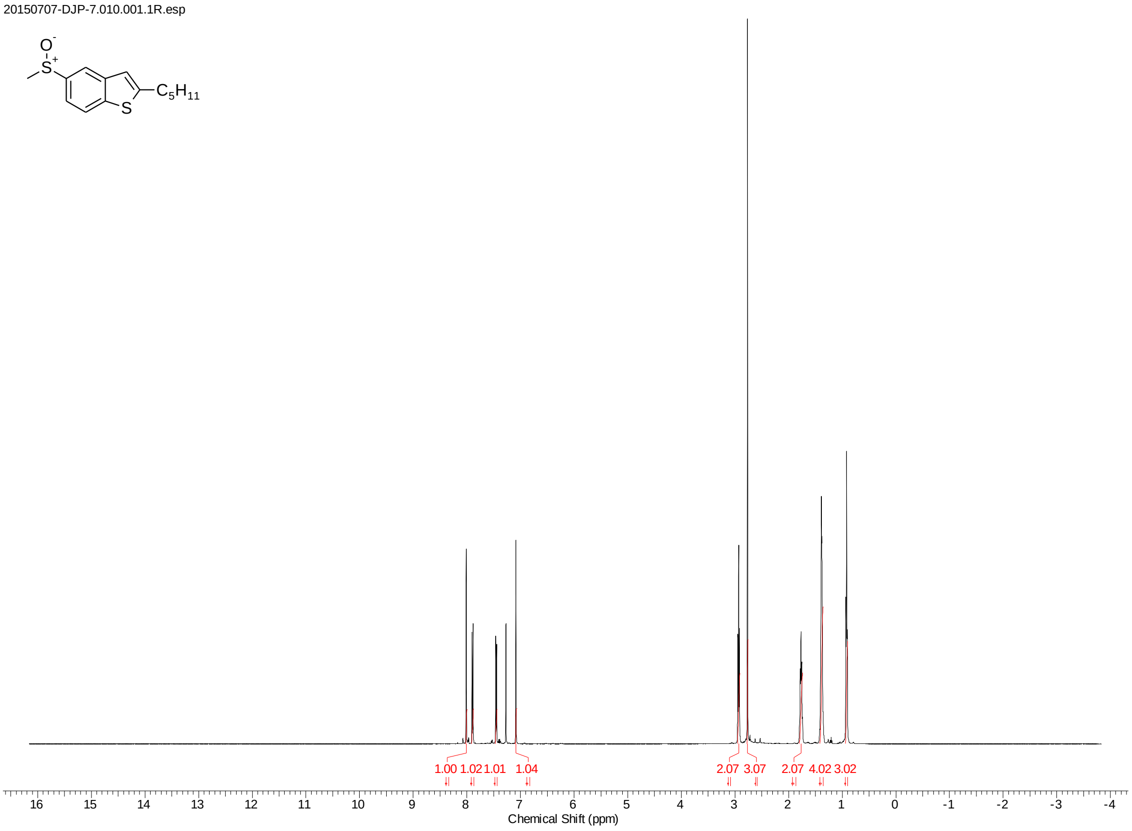

^{13}C NMR (100 MHz, $CDCl_3$)

20150707-DJP-7.011.001.1R.esp

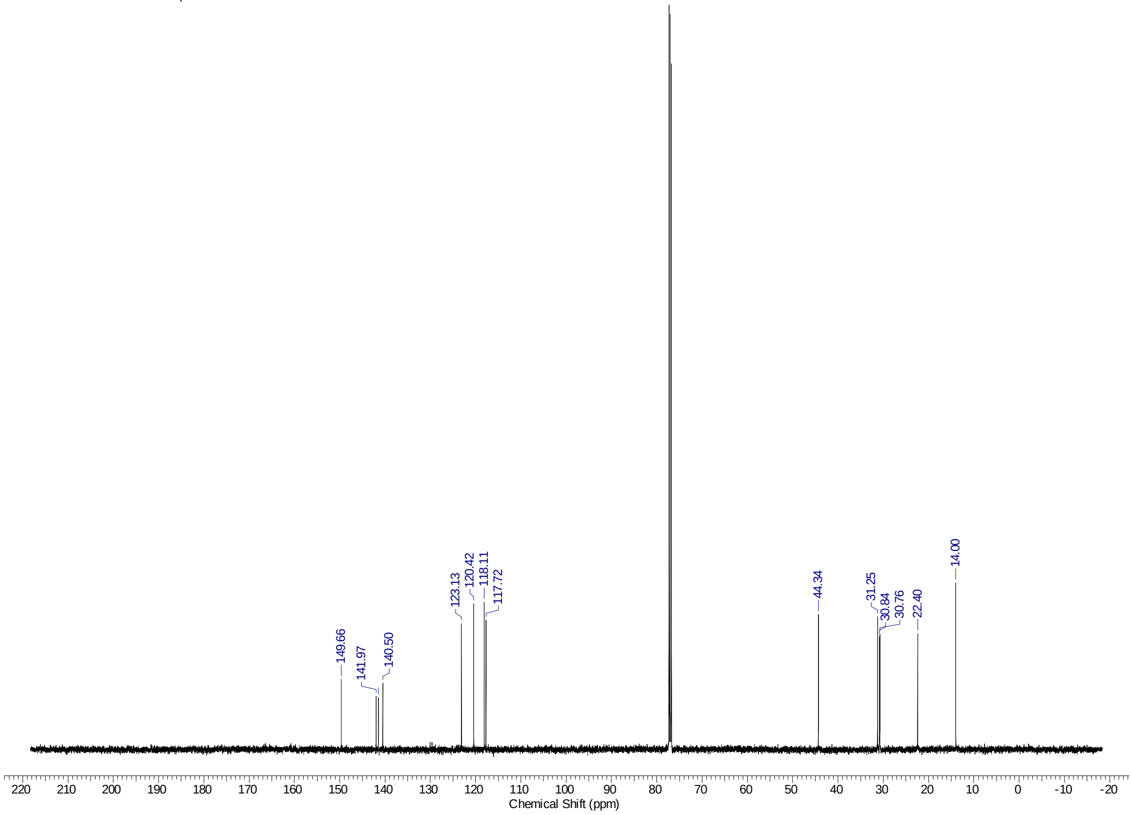

4-(3-Cyclohexylprop-2-yn-1-yl)-5-(methylsulfanyl)-2-pentylbenzo[b]thiophene S29

^{1}H NMR (400 MHz, $CDCl_3$)

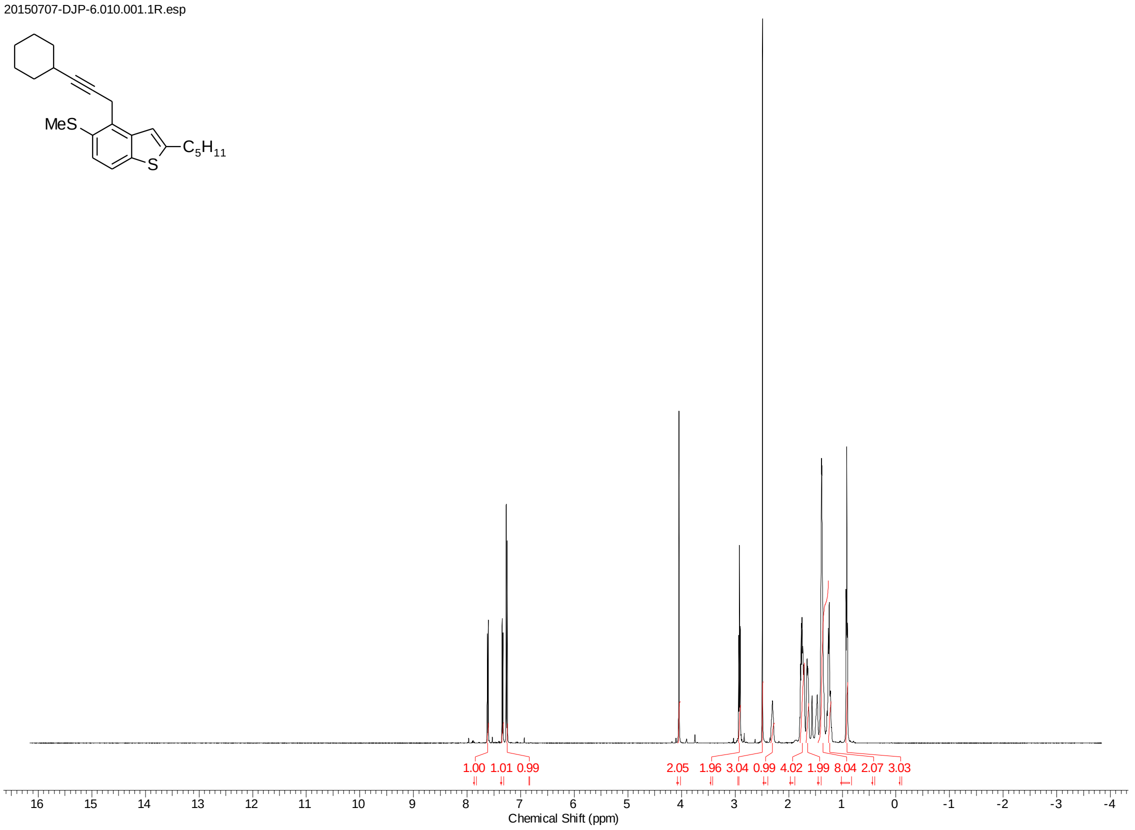

^{13}C NMR (100 MHz, $CDCl_3$)

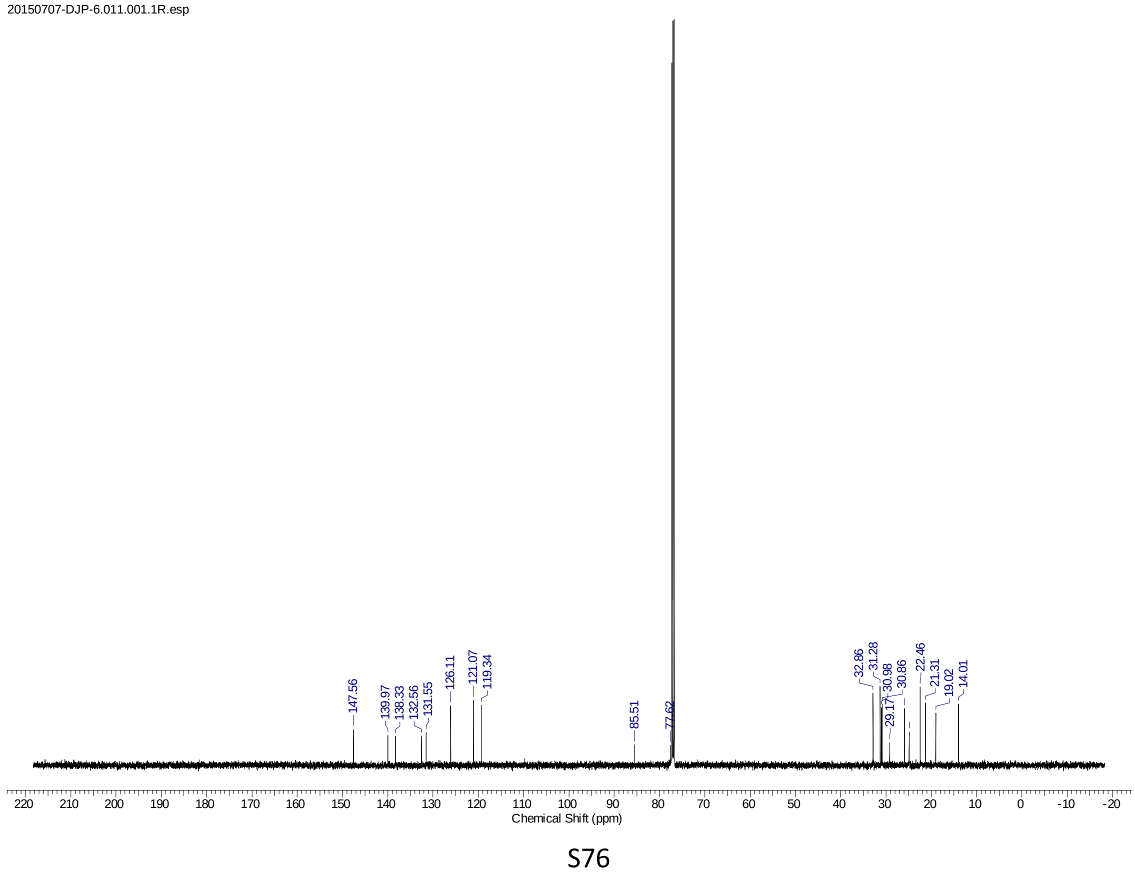

2-(Cyclohexylmethyl)-7-pentylbenzo[1,2-b:4,3-b']dithiophene 21

^{1}H NMR (400 MHz, $CDCl_3$)

20150707-DJP-8.010.001.1R.esp

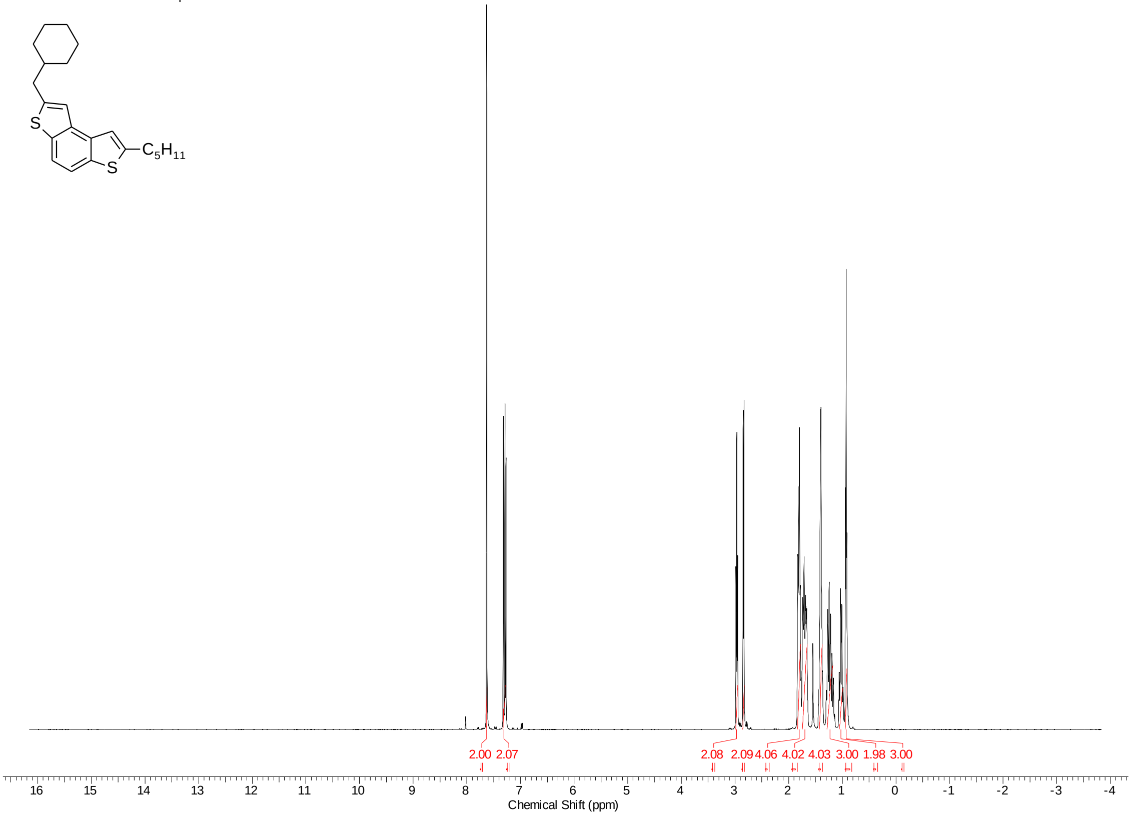

^{13}C NMR (100 MHz, $CDCl_3$)

20150707-DJP-8.011.001.1R.esp

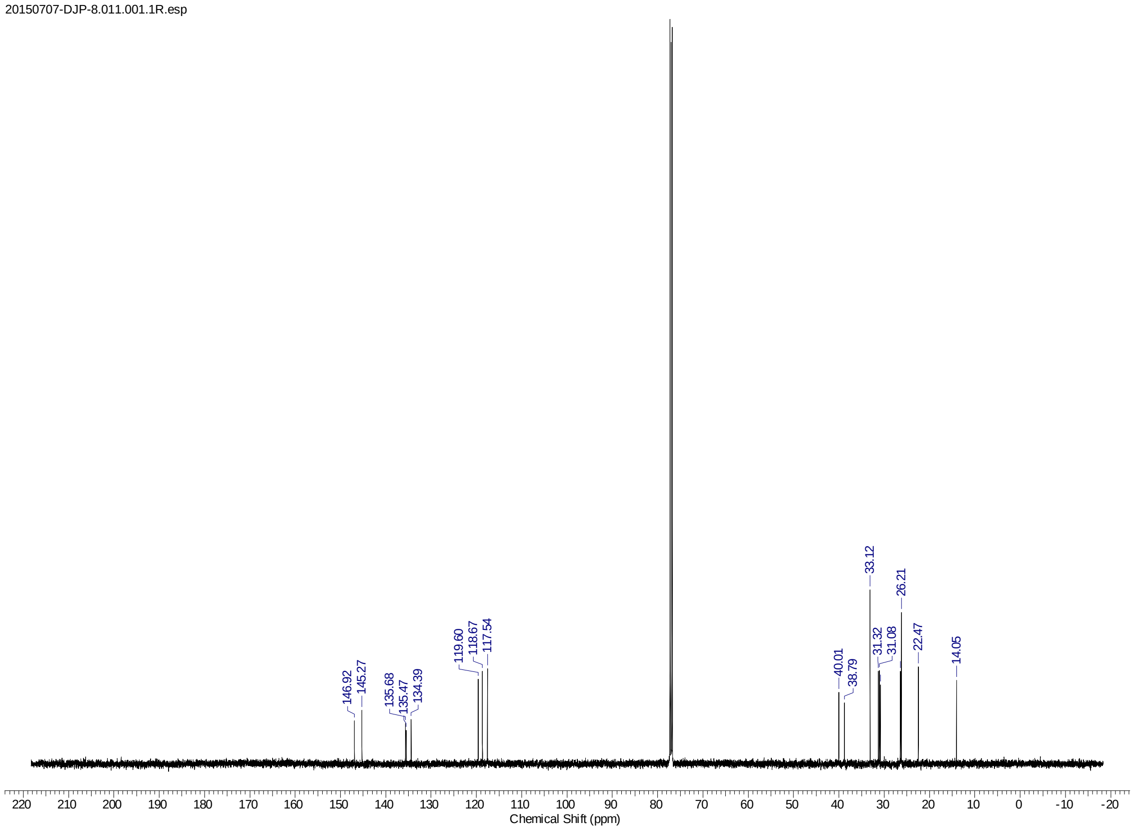

6,12-Dibutylbenzo[1,2-*b*:4,5-*b*']bis[*b*]benzothiophene 22

^{1}H NMR (400 MHz, $CDCl_3$)

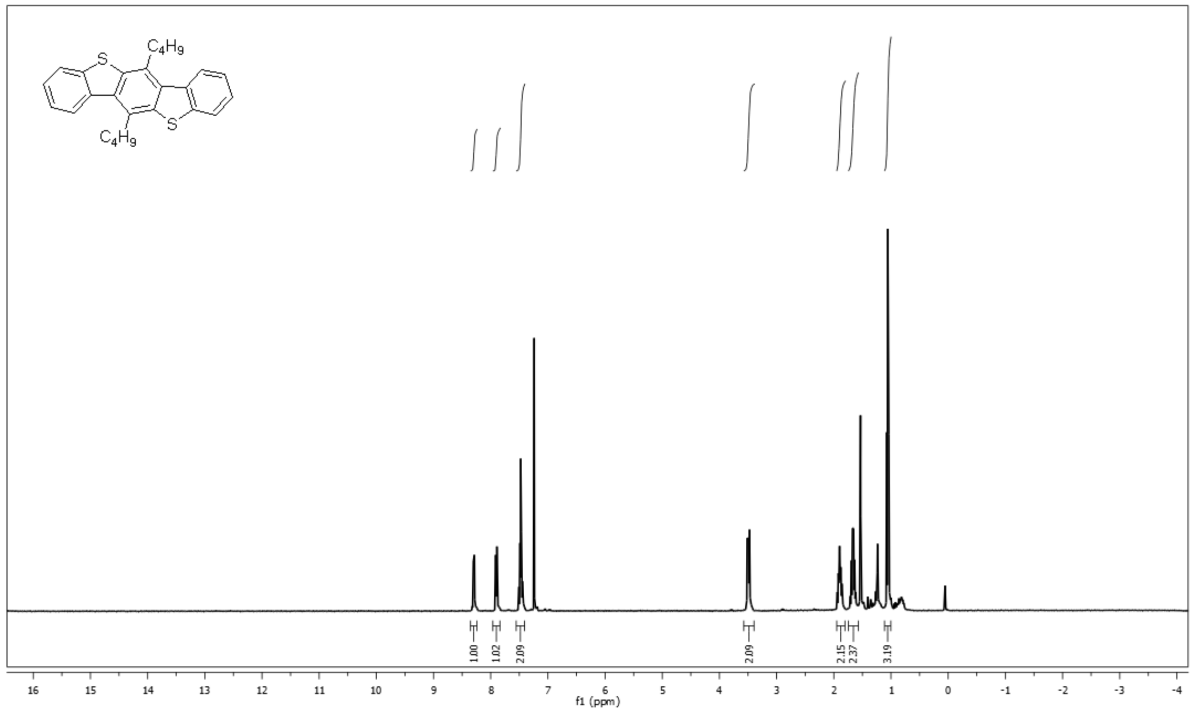

^{13}C NMR (100 MHz, $CDCl_3$)

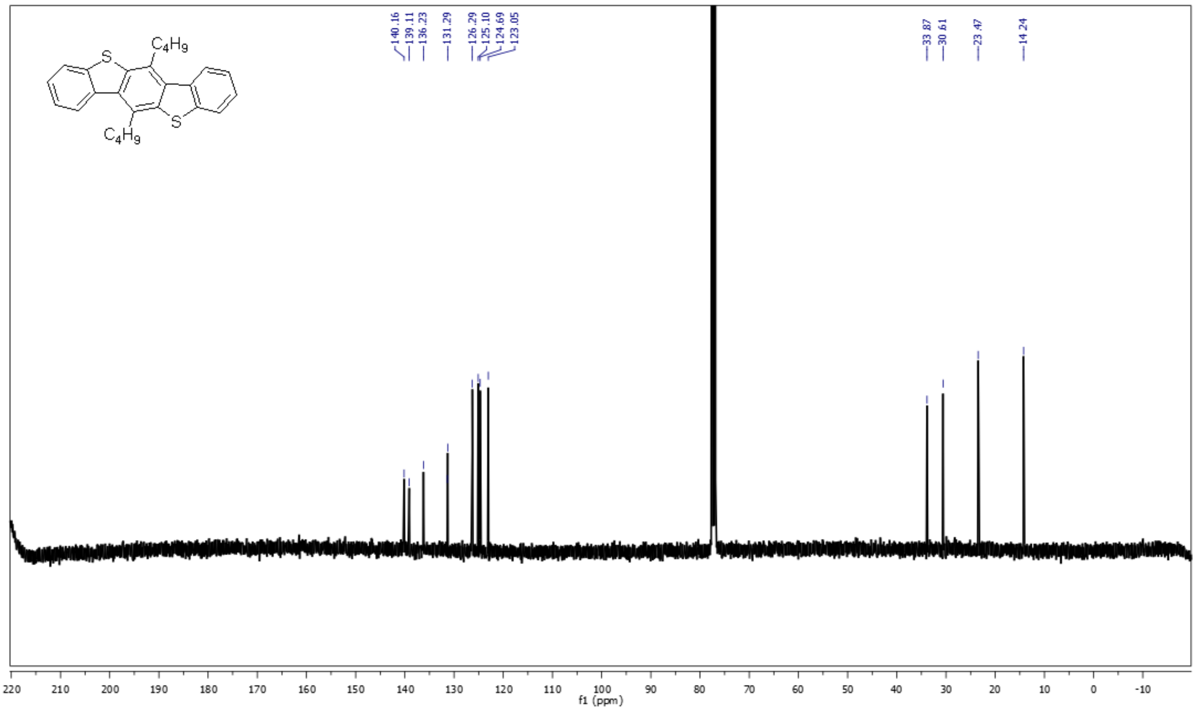

6,12-Dibutylbenzo[1,2-*b*:4,5-*b*']bis[*b*]5-methylbenzothiophene 23

^{1}H NMR (400 MHz, $CDCl_3$)

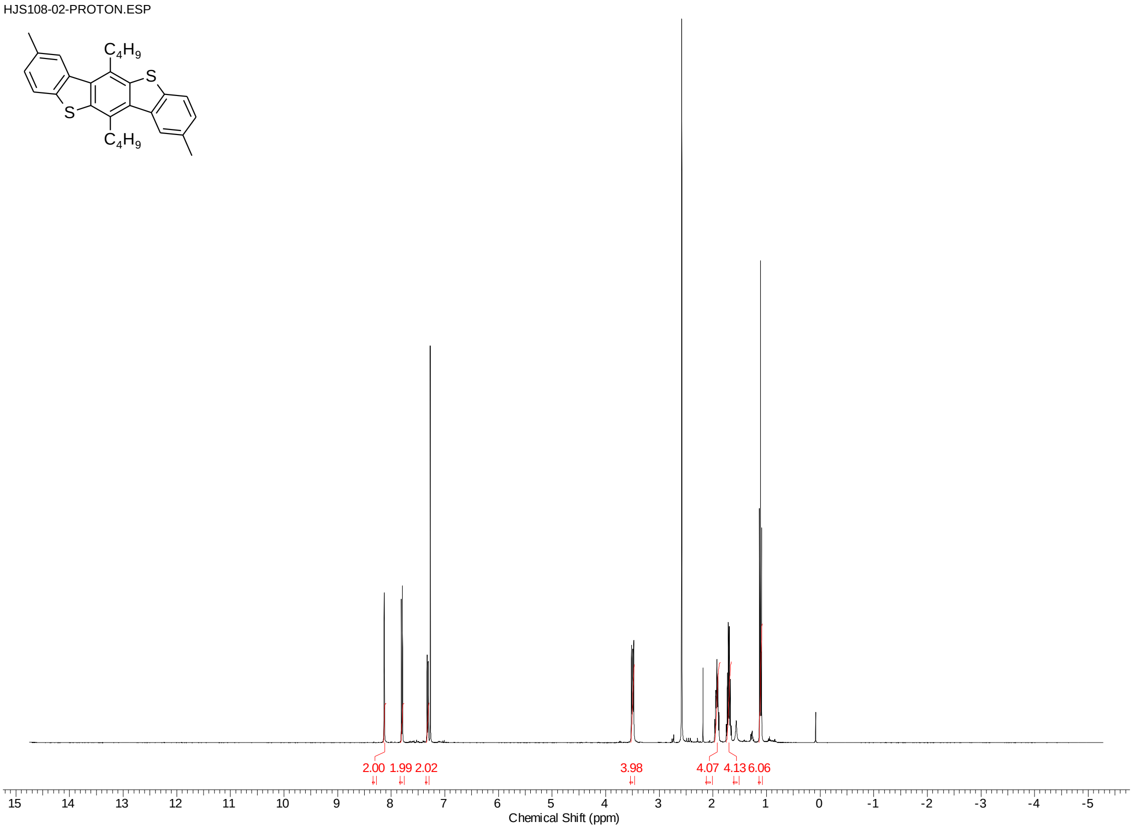

^{13}C NMR (100 MHz, $CDCl_3$)

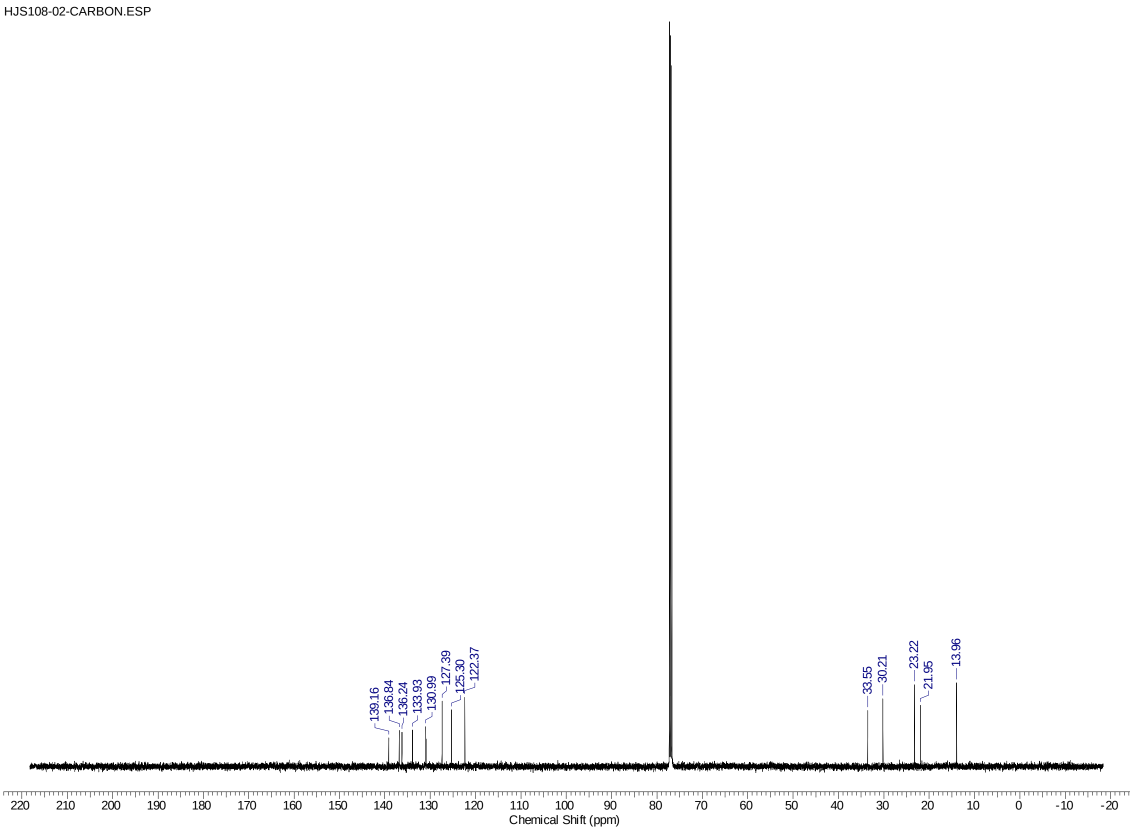

6,12-Dibutylbenzo[1,2-*b*:4,5-*b*']bis[*b*]5-methoxybenzothiophene 24

^{1}H NMR (400 MHz, $CDCl_3$)

HJS120-PROTON.ESP

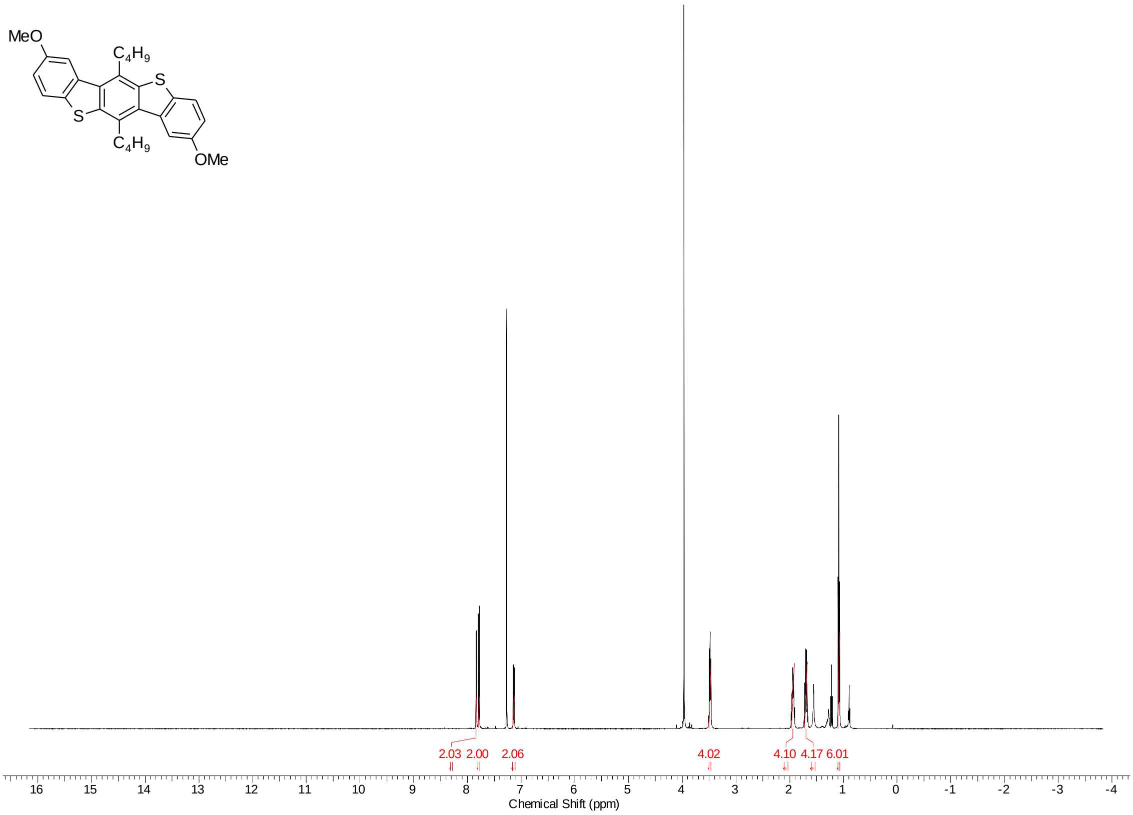

^{13}C NMR (100 MHz, $CDCl_3$)

HJS120-02-CARBON.ESP

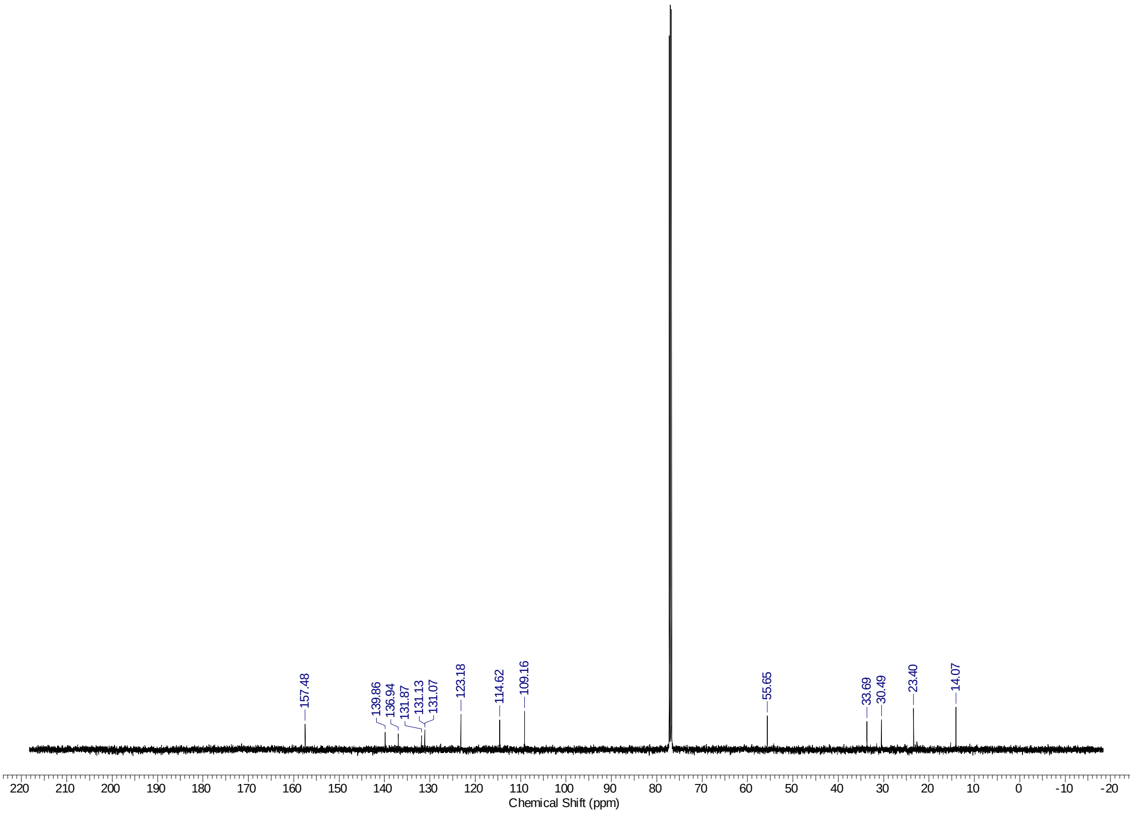

6,12-Dibutylbenzo[1,2-*b*:4,5-*b*’]bis[*b*]5-fluorobenzothiophene 25

^{1}H NMR (400 MHz, $CDCl_3$)

HJS117-02-PROTON.ESP

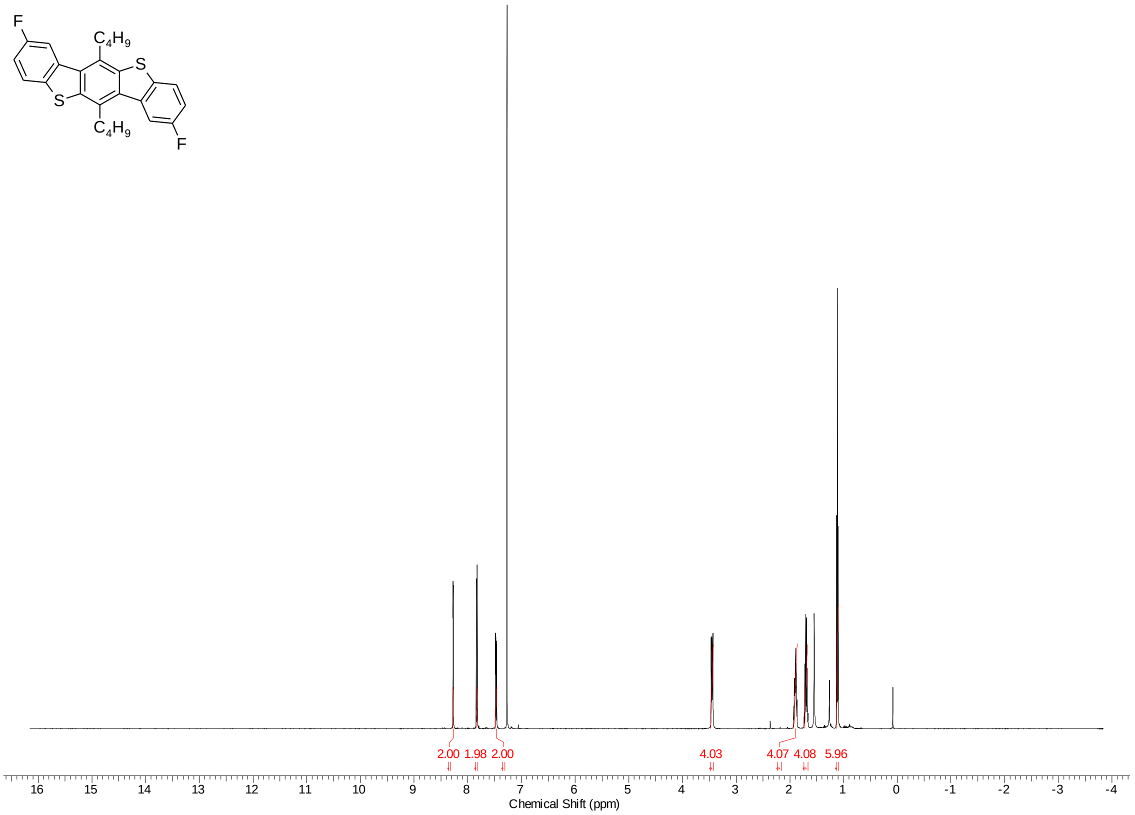

^{13}C NMR (100 MHz, $CDCl_3$)

HJS112-02-CARBON.ESP

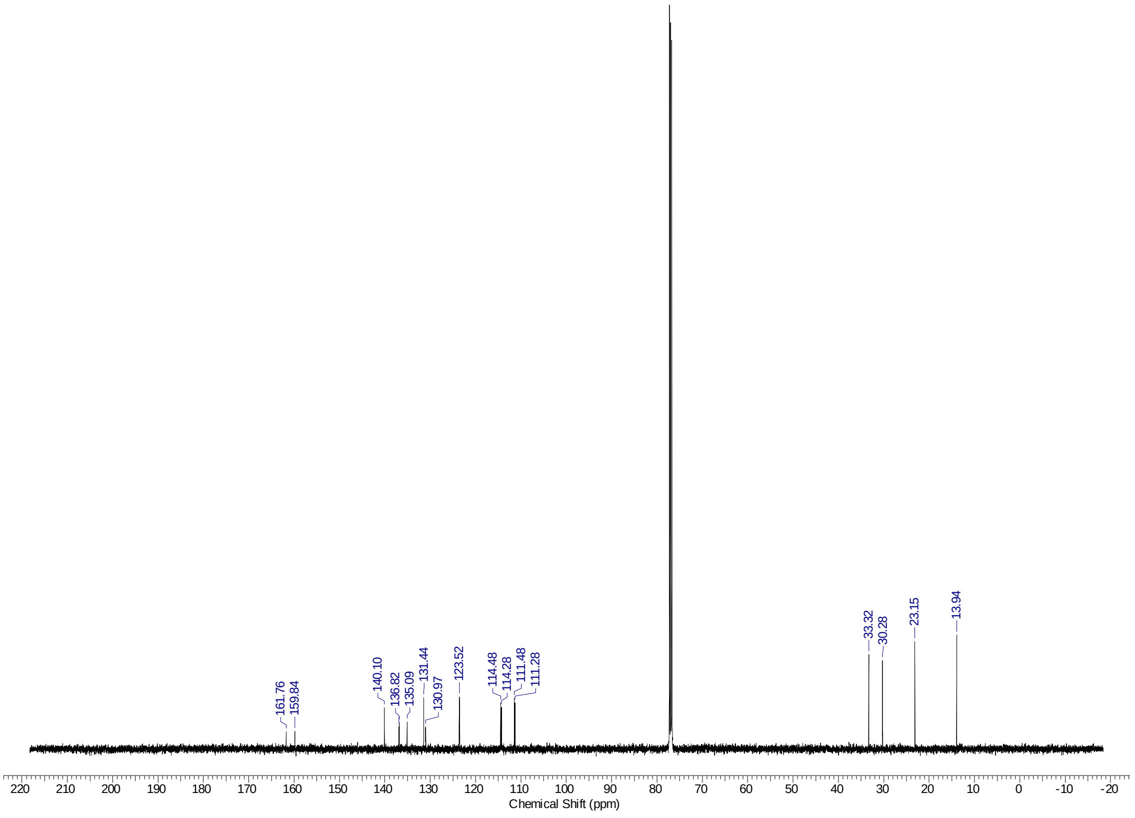

6,12-Dibutylbenzo[1,2-*b*:4,5-*b*’]bis[*b*]6-fluorobenzothiophene 26

^{1}H NMR (400 MHz, $CDCl_3$)

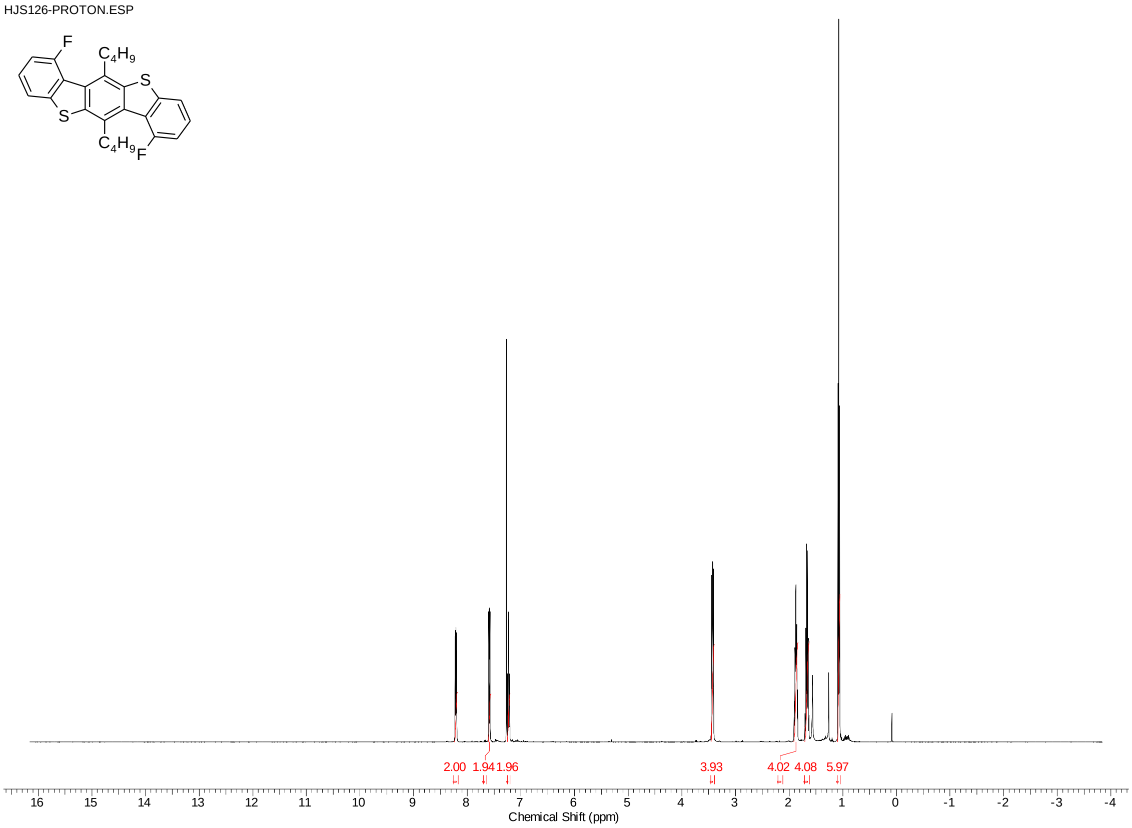

^{13}C NMR (100 MHz, $CDCl_3$)

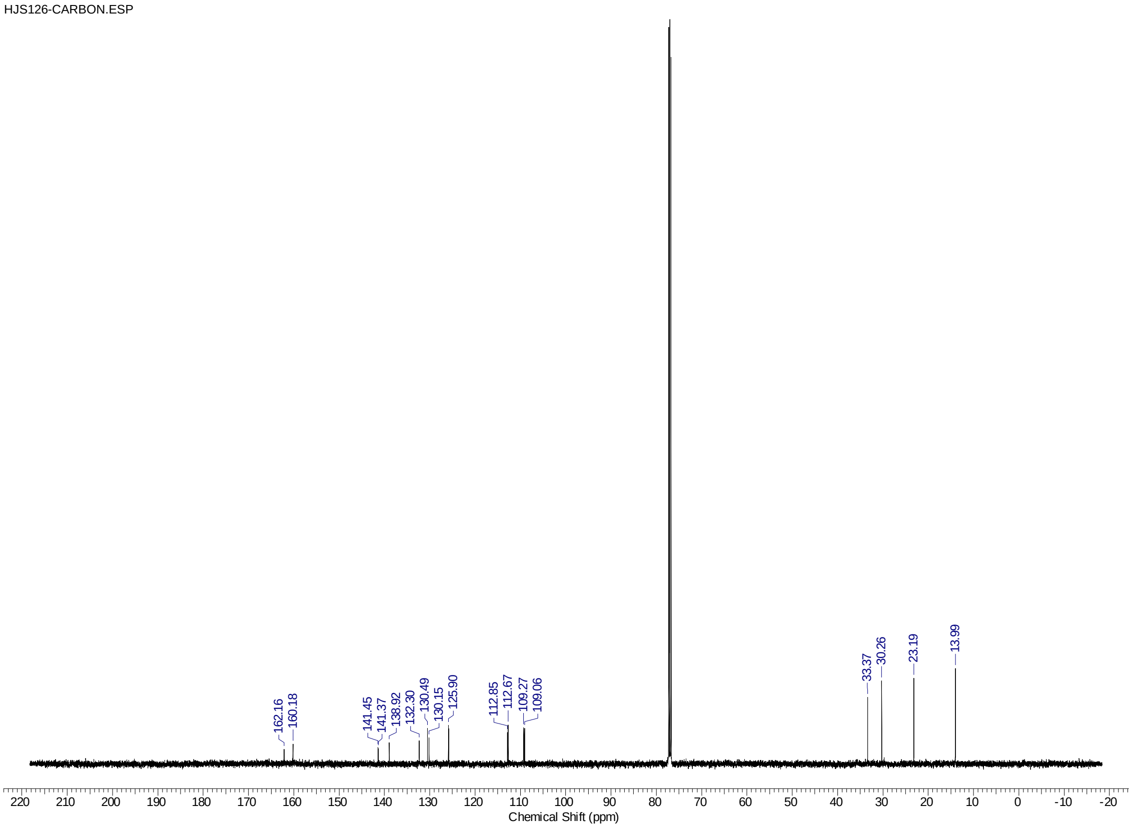

6,12-Dibutylbenzo[1,2-*b*:4,5-*b*’]bis[*b*]5-chlorobenzothiophene 27

^{1}H NMR (400 MHz, $CDCl_3$)

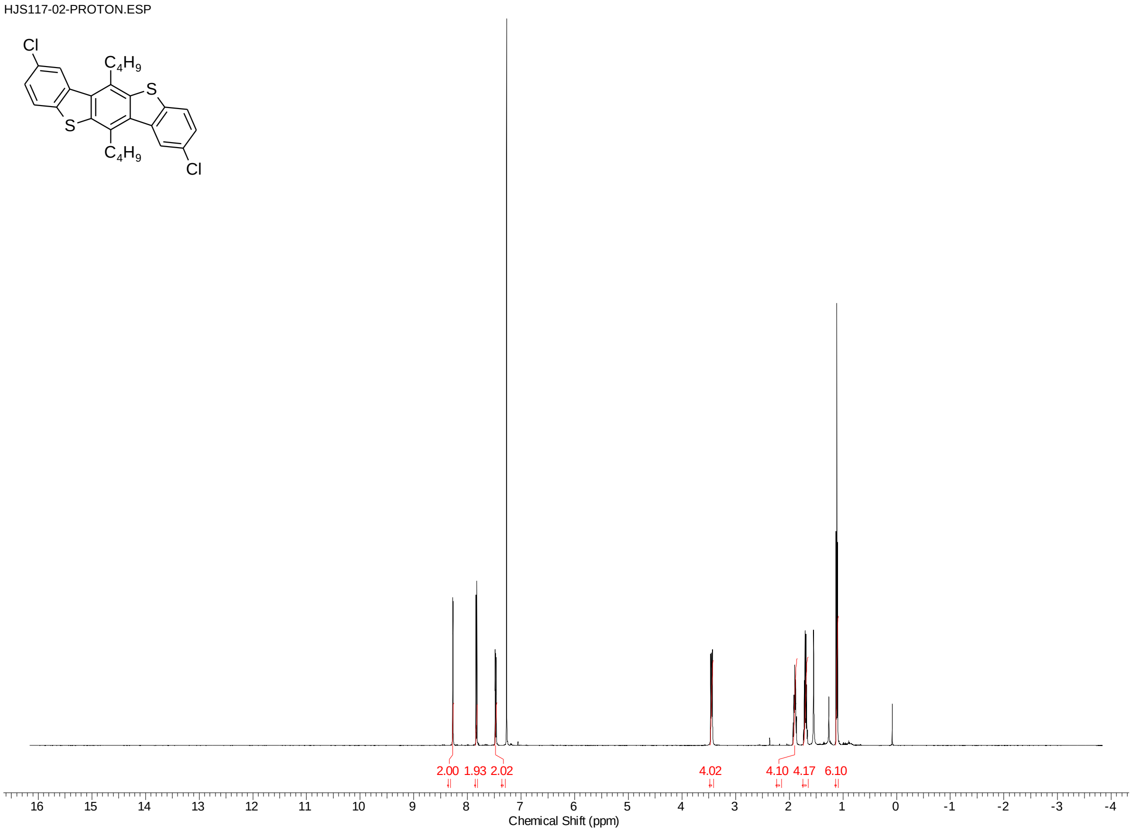

^{13}C NMR (100 MHz, $CDCl_3$)

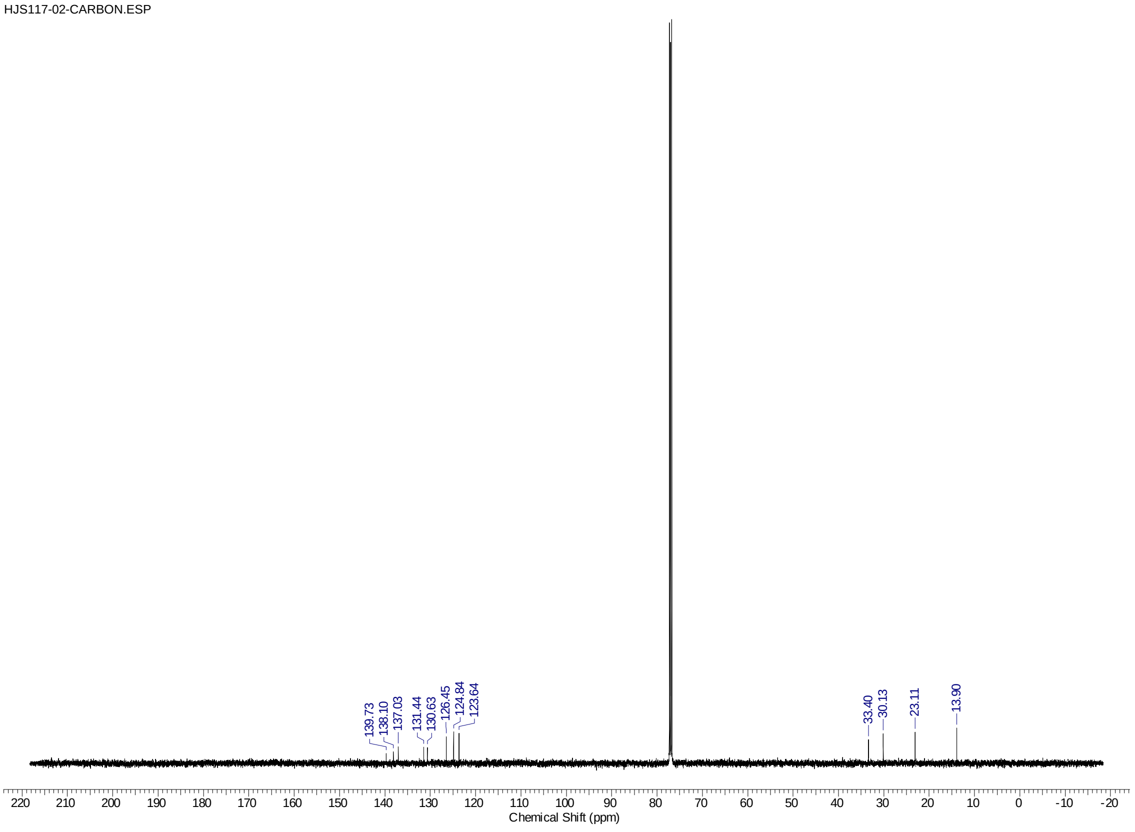

6,12-Dihexylbenzo[1,2-*b*:4,5-*b*’]bis[*b*]benzothiophene 28

^{1}H NMR (400 MHz, $CDCl_3$)

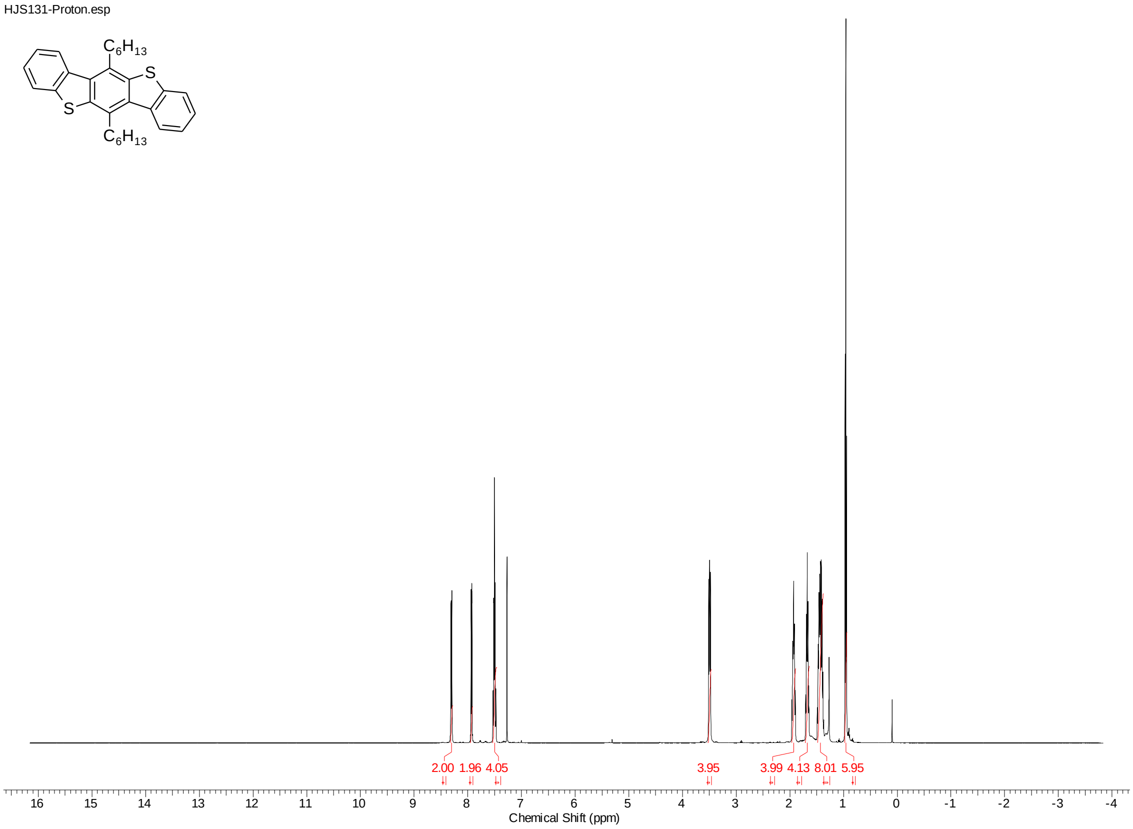

^{13}C NMR (100 MHz, $CDCl_3$)

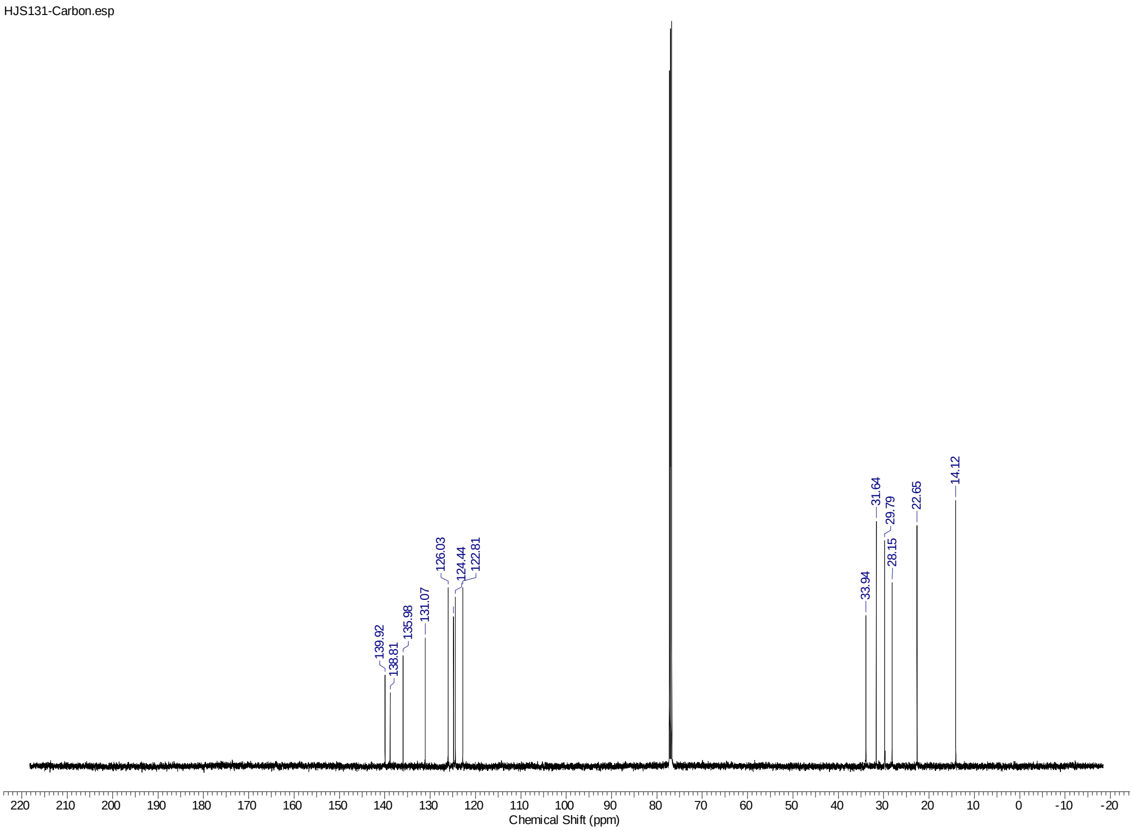

Field Effect Transistors

Field effect transistors were created for the compounds **22, 23, 11, 12, 13** and **14.** Thin films were characterised by X-ray diffraction and atomic force microscopy and transistors were created and analysed using conventional thin film transistor techniques.

Thin Film Deposition

Heavily doped silicon (n++) with 300 nm of thermally grown silicon dioxide was used as the substrate and gate electrode for all following experiments. The substrates were cleaned by sonication in acetone, propan-2-ol and methanol followed by drying under a stream of nitrogen and subjected to a UV-Ozone treatment for approximately 20 minutes.

An octadecyltrichlorosilane (OTS) monolayer was deposited in order to reduce the surface energy, passivate traps and improve thin film growth. OTS treatment was achieved by spin coating of a solution from chloroform as has been reported elsewhere.[1] OTS treated substrates were all washed with organic solvents and dried with nitrogen before being transferred to the vacuum evaporation chamber for thin film deposition.

Evaporation of organic semiconductors was performed in a modified Edwards Auto306 vacuum evaporator with a base pressure of 7×10^{-6} mbar. Nominally, between 30 and 40 nm of compounds was deposited onto substrates held at between 30 and 80 °C at a rate of 0.5 As^{-1}

To create thin film transistors the semiconductor coated substrates were transferred to a separate Edwards Auto500 vacuum evaporator (base pressure 1×10^{-7} mbar) for gold evaporation. Nominally, 50 nm of gold was deposited on top of the organic layers at a rate of 1 As^{-1} through a shadow mask, defining source and drain electrodes.

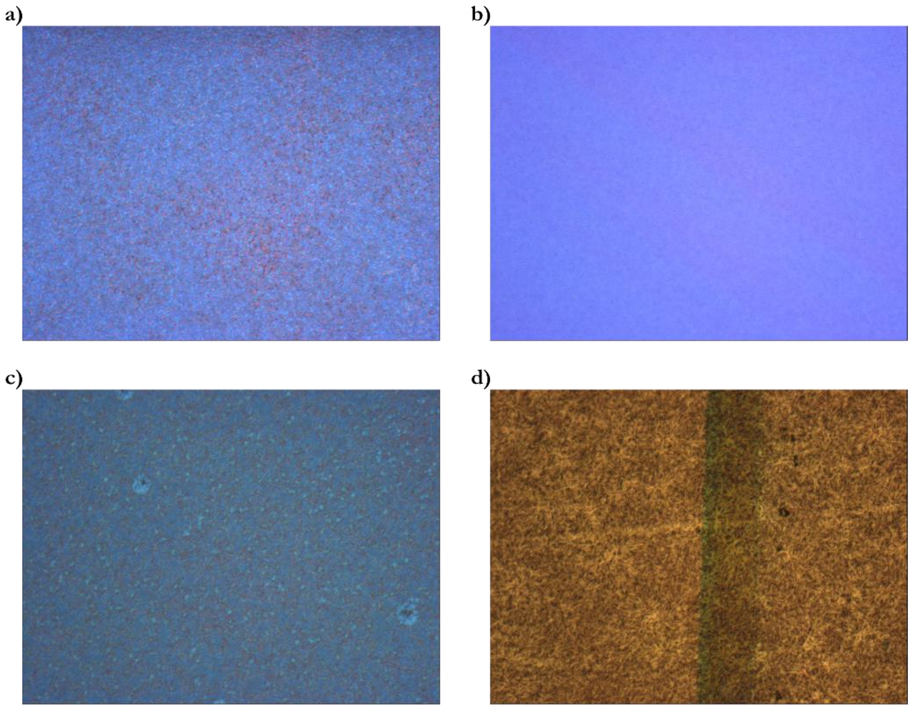

Figure 1: Optical microscopy images of evaporated thin films of a) **22** on OTS, b) **23** on OTS, c) **22** on SiO2 and d) **22** on OTS, and gold electrodes more material was deposited during this evaporation.

Thin Film Characterisation

The thin films were characterised using a Park XE100 atomic force microscope in tapping mode and a Bruker D8 discover X-ray diffractometer for out-of-plane X-ray diffraction.

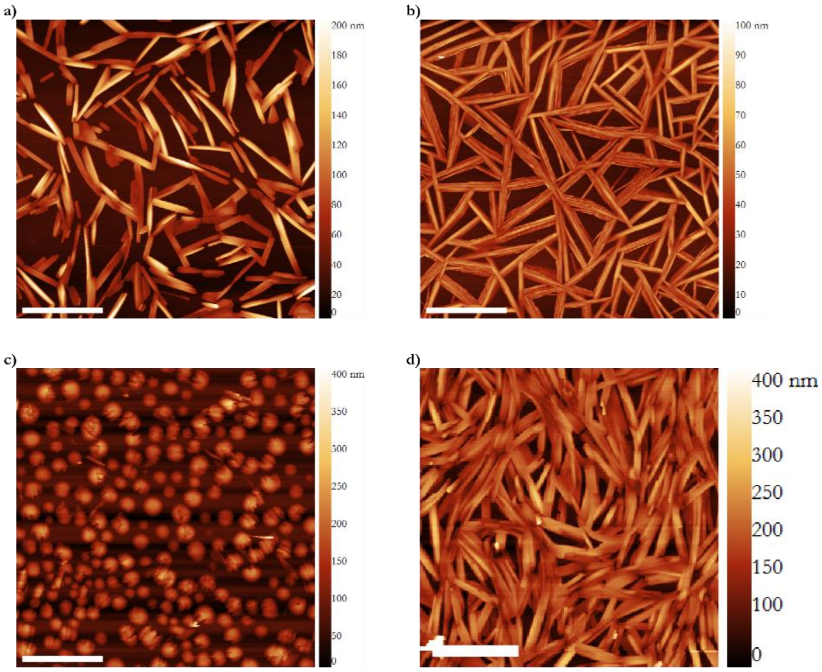

Figure 2: AFM topography images of thin films of a) **22** on OTS, b) **23** on OTS, c) **22** on SiO2 and d) **22** on OTS, and gold electrodes more material was deposited during this evaporation.

BDTs

Optical microscopy gives us an indication that the films are composed of small crystallites on both OTS treated surfaces and on bare SiO_2 (**Figure 1**). AFM topography, **Figure 2**, gives us further insight into the nature of the crystallisation. On the OTS treated substrates both compounds exhibit small needle like crystallites whereas when compound **22** is deposited on to SiO_2 it forms small nodule like structures and no thin film structure was observed in either case.

Thin film (out-of-plane) X-ray diffraction studies were conducted to look at the crystal structure of the samples. **Figure 3** shows the X-ray diffraction of samples of **22** and **23** on OTS and **22** on SiO_2. On OTS both compounds show a single large peak representative of the highly crystalline nature of the needle like crystals. The reflections for compounds **22** and **23** on OTS correspond to a d-spacing of 12.2 A and 11.2 A respectively. The d-spacings for compound **22** on SiO_2 are 11.3 A and 9.6 A confirming the difference in crystal structure

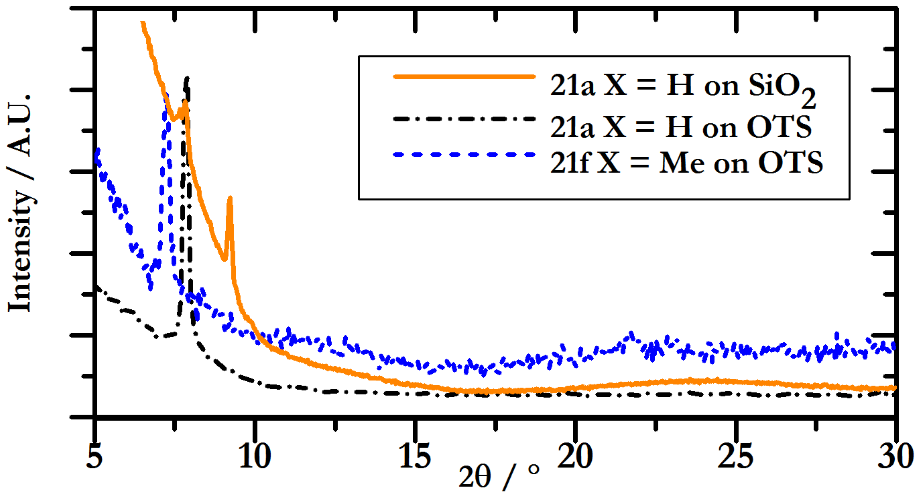

Figure 3: Out-of-plane X-ray diffraction of thin films. Compound **22** on OTS (dot-dash black), compound **23** on OTS (dash blue) and compound **22** on SiO_2 (solid orange)

a) b) c) d)

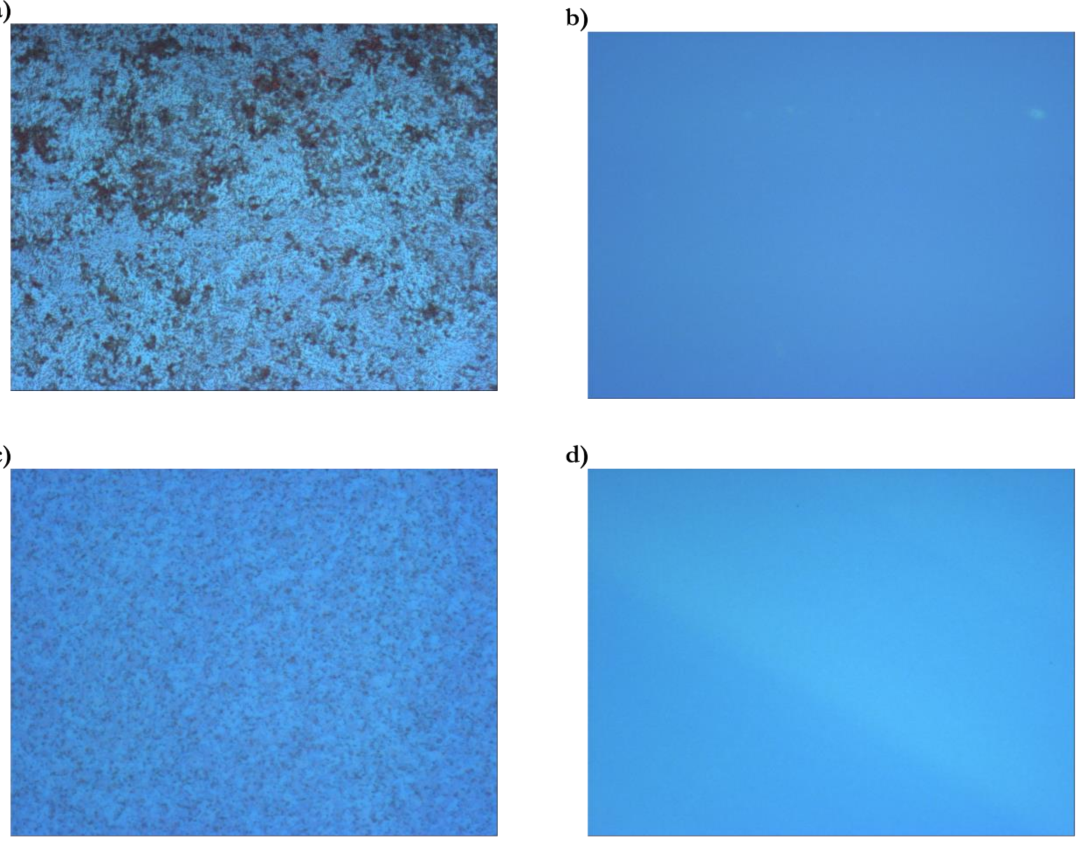

Figure 4: Optical microscopy images of evaporated thin films of a) **12**, b) **11** c) **14** and d) **13**

seen by AFM

NDTs

From the optical microscopy images (**Figure 4**) it is clear that the isomers **12** and **14** do not form complete films but instead forms small isolated crystals over the surface. However, for the isomers **11** and **13**, both the heptyl (**11**) and the ketone (**13**) form complete, smooth films. **Figure 5** confirms this incomplete coverage for isomers **12** and **13** via the AFM topography. **Figure 5a** and **c** show isomers **12 and 13** with large gaps separating crystalline material which hinders and percolation pathways for charge conduction. **Figures 5b** and **d**, however, show full crystalline thin films with little or no holes through to the substrate. As confirmation, of the thin film structure, **Figure 6** shows the out-of-plane X-ray diffraction of all 4 thin films. For compounds **12** (black) and **14** (green) there are very sharp peaks represent the very crystalline nature of the small crystallites on the surface. In the case of compounds **11** (red) and **13** (blue) the peaks are broader, indicative of a thin film crystal structure.

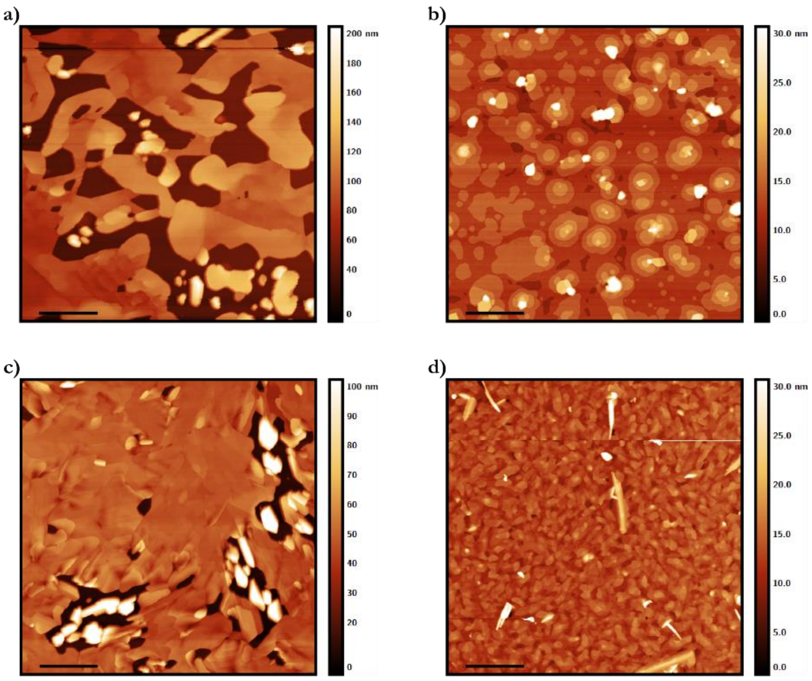

Figure 5: AFM topography images of thin films of a) **12** b) **11**, c) **14** and d) **13**.

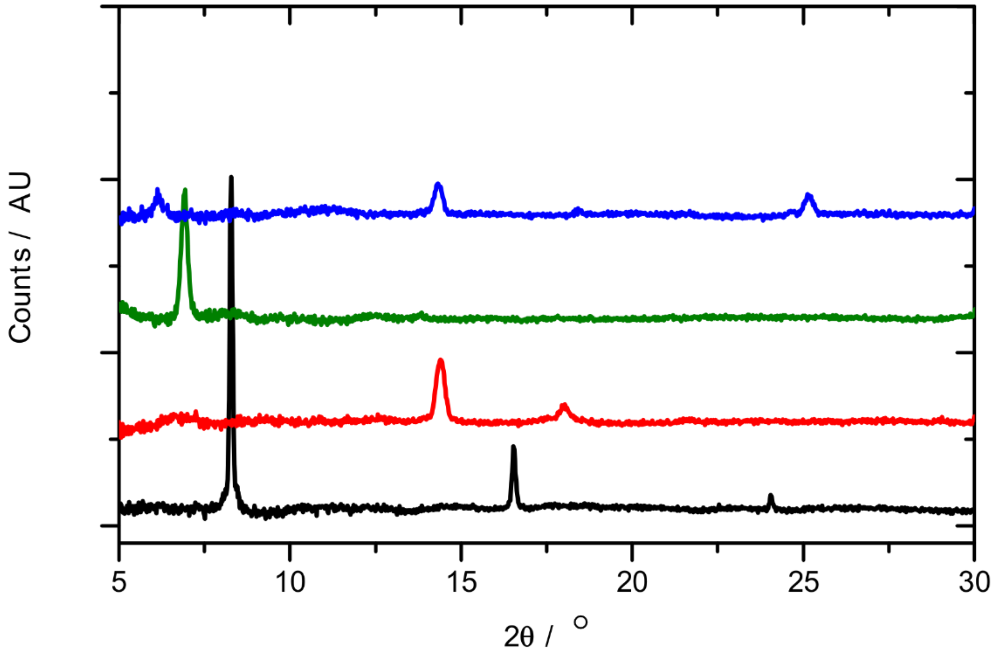

Figure 6: Out-of-plane X-ray diffraction of thin films. From bottom to top, **12** (black), **11** (red), **14** (green) and **13** (blue).

Transistor Characterisation

Transistors were created via the evaporation of gold electrodes in a top contact bottom gate configuration. The devises were then tested in the saturation regime using the standard equation.

$$I_D = C_i \frac{W}{2L} \mu_{sat} (V_G - V_T)^2$$

Where, I_D is the source-drain current, C_i is the capacitance, W the channel width, L the channel length, μ_{sat} is the saturation mobility, V_G is the swept gate voltage and V_T is the threshold voltage. The transistors had a channel width of 2000 μm and a channel length of 60 μm, the 300 nm silicon dioxide with OTS monolayer was calculated to have a capacitance of approximately 11.4 $nFcm^{-2}$.

BDTs

Only devices that were created on OTS substrates demonstrated transistor characteristics. Compound **22** showed a mobility of approximately 1 × 10^{-5} $cm^2V^{-1}s^{-1}$ an on/off current ratio of 10^4 and a very high threshold voltage. This can all be attributed to the poor percolation pathways of the needle like crystals, low coverage and poor interconnection between the grains which also meant that only 2 out of 9 devices were operational. Compound **23** demonstrated improved characteristics with and average mobility of approximately 5 × 10^{-4} $cm^2V^{-1}s^{-1}$ an on off ratio of 10^5 and threshold voltage around -20 V, 8 out of 9 devices exhibited transistor behaviour. Although exhibiting reasonable OFET characteristics the devices show poor reverse sweep performance as shown in **Figure 7c**.

It should be noted that during the deposition procedures a nominal value for the material density was used (1.23 g/cm^3). As such, in both cases the evaporation rate stated will not be an accurate representation of the actual rate. In fact, it is quite noticeable that the two compounds themselves have quite different densities as one is a hard powder (**22**) whilst the other is a more light weight solid (**23**). This leads to a mismatch even in the evaporation rate of the two compounds which would severely underestimate the rate of evaporation of compound **23**, this fits with the observation of higher coverage of needles from AFM, **Figure2b**. This may be the reason for the increased number of working devices and improved performance. Hence, it was decided to increase the coverage of compound **22** to see if that would improve the performance. **Figure 1d** shows the image of an increased coverage sample of **22**, **Figure 2d** is the AFM image of the same region showing a much increased number of needle crystals. Transfer characteristics are shown in **Figure 7d**, overall device performance is improved. A mobility of approximately 1 × 10^{-3} $cm^2V^{-1}s^{-1}$ was achieved, a threshold voltage of -10 V and an on/off ration of 10^5. Although improved performance only 2 of the 9 devices worked and reverse sweep behaviour is poor.

It should be noted that there was only enough material of **23** to do the one evaporation and more coverage was not obtained for this material.

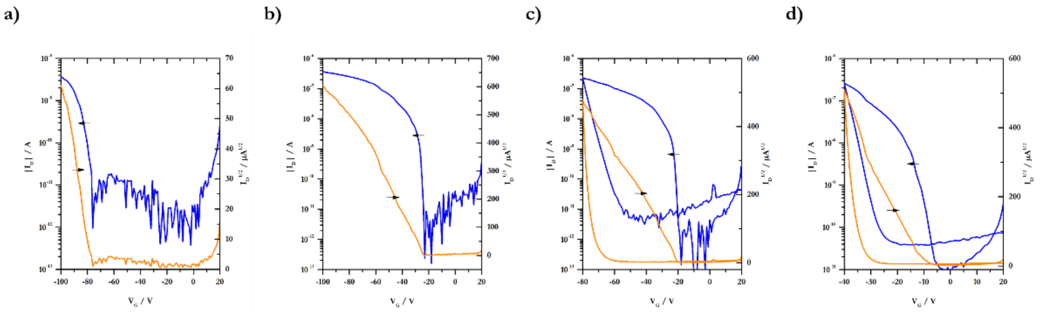

Figure 7: Transfer characteristics for a) compound **22**, b) and c) compound **23** and d) **22** with increased material. Only devices made on OTS treated substrates were observed.

NDTs

Compounds **12** and **14** showed no switching behaviour as expected from the incomplete films seen in **Figure 4**. **Figure 8a** shows a transfer curve for the compound **11**. The device shows good p-type behaviour with an average mobility of 0.21 ± 0.02 cm^2Vs^{-1}, threshold voltage of -22 ± 4 and on/off ratio of 10^7 over 9 devices. The devices do, however, show large hysteresis and memory behaviour as has been seen in similar materials before.[2] **Figure 8b** shows a transfer curve for the compound **13,** it does show limited p-type behaviour but only 10 % of devices tested worked. This poor behaviour could be attributed to the poor alignment of the

HOMO of the molecule and the work function of the gold electrodes. Devices made from compounds **14** and **13** were also tested under nitrogen as n-type devices but no switching behaviour was observed. The results from compounds **12** and **11** are consistent with that previously reported for these materials.[3]

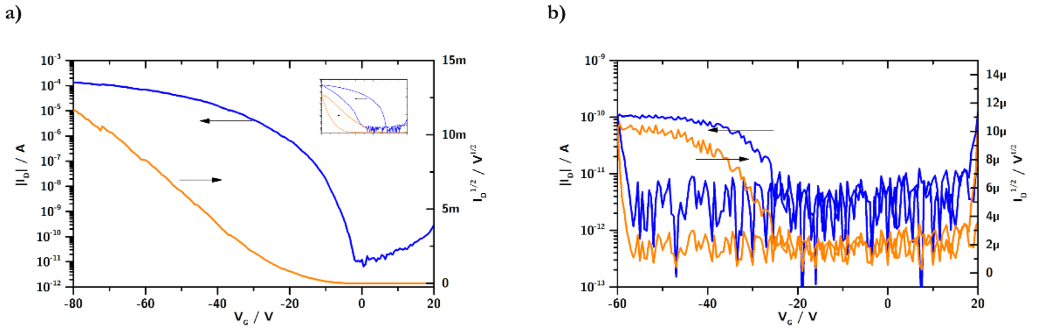

Figure 8: Transfer characteristics for a) compound **11**, inset hysteresis, b) compound **13**.

References

[1] Y. Ito, A. A. Virkar, S. Mannsfeld, J. H. Oh, M. Toney, J. Locklin, Z. Bao, *J. Am. Chem. Soc.* **2009**, *131*, 9396.

[2] M. Mushrush, A. Facchetti, M. Lefenfeld, H. E. Katz, T. J. Marks, *J. Am. Chem. Soc.* **2003**, *125*, 9414.

[3] S. Shinamura, I. Osaka, E. Miyazaki, A. Nakao, M. Yamagishi, J. Takeya, K. Takimiya, *J. Am. Chem. Soc.* **2011**, *133*, 5024.

UV/Vis Spectra and Cyclic Voltammetry

Experimental Details

UV-Vis absorption spectra were recorded on a Varian Cary 5000 UV-Vis- NIR spectrophotometer in dichloromethane at room temperature.

Cyclic voltammetry was performed in dichloromethane solution scanning at 100 mV s^{-1} on a BASI Epsilon electrochemical workstation with a three-electrode cell, $Ag/AgNO_3$ as reference electrode, platinum wire as counter electrode and working electrode, in nitrogen-purged, 0.1 M solution of tetrabutylammonium hexafluorophosphate as a supporting electrolye at room temperature.

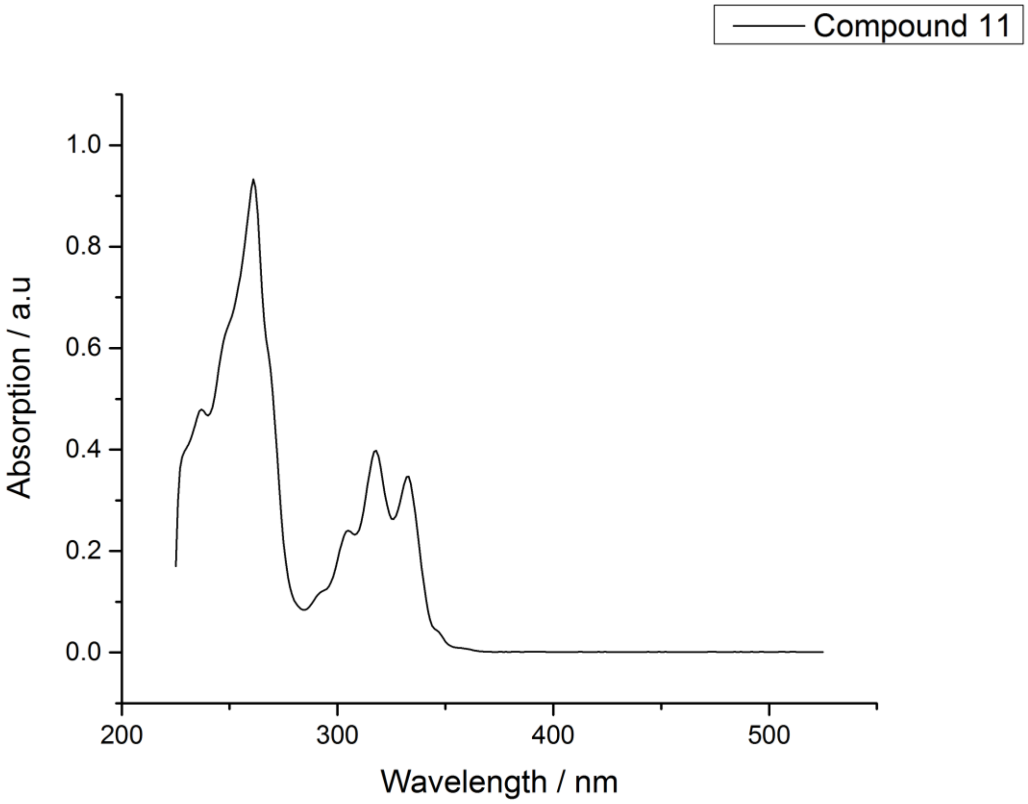

Absorption spectra of Compound 11

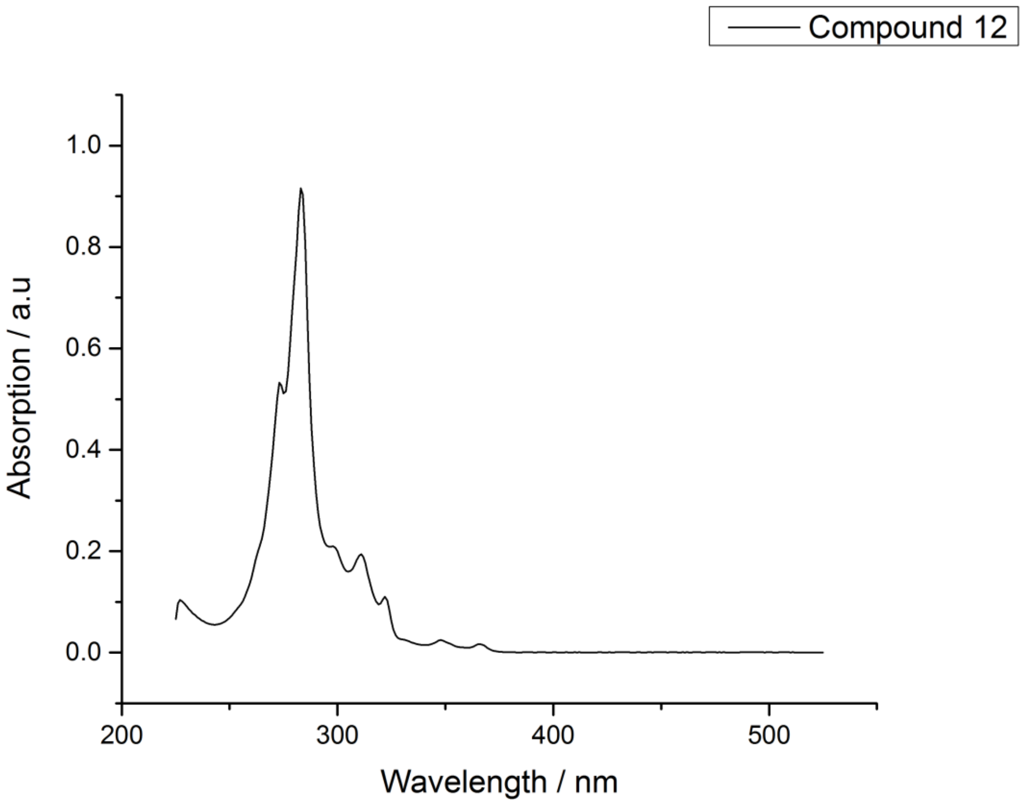

Absorption spectra of Compound 12

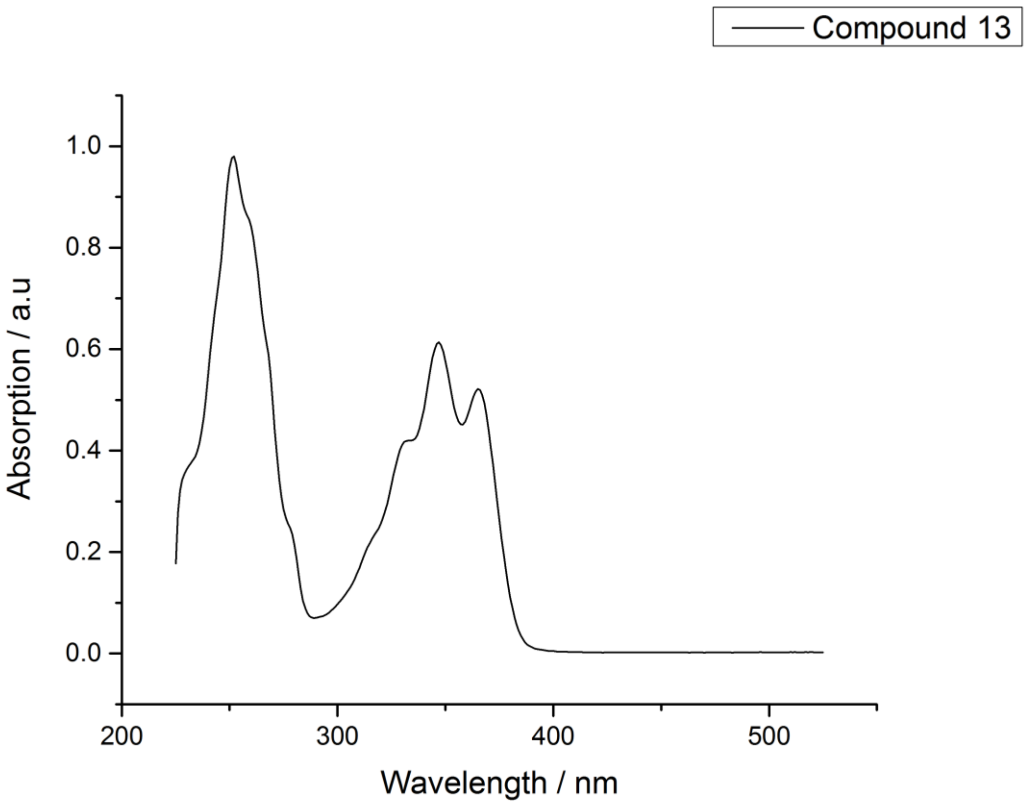

Absorption spectra of Compound 13

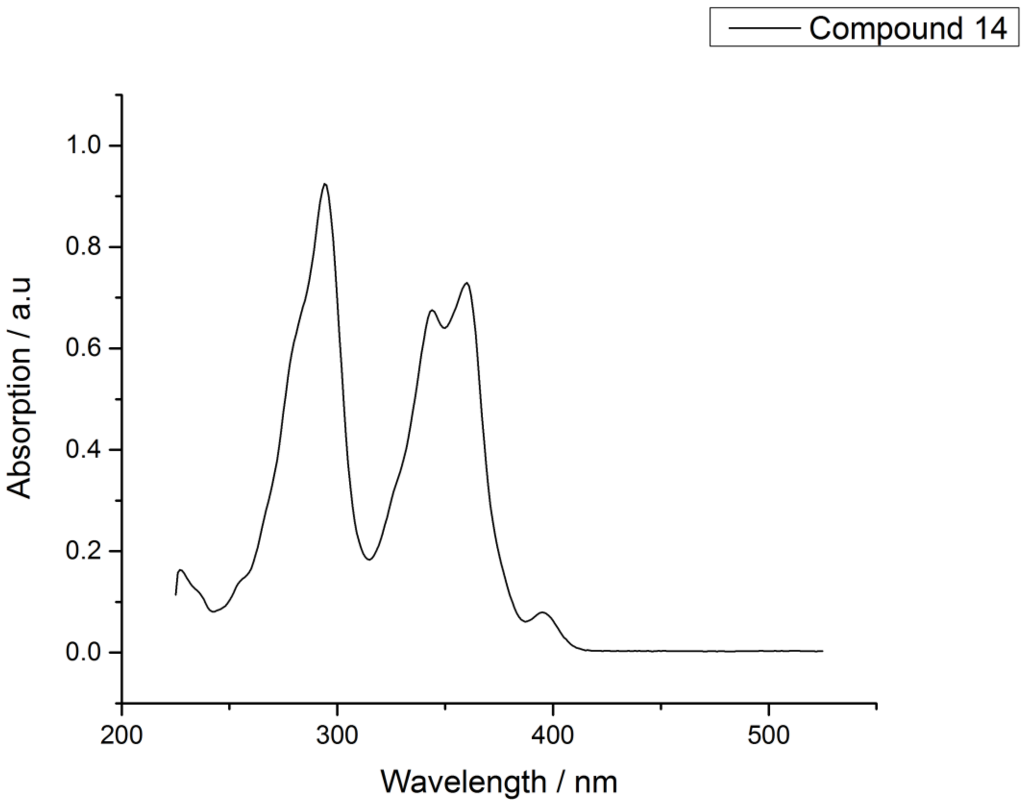

Absorption spectra of Compound 14

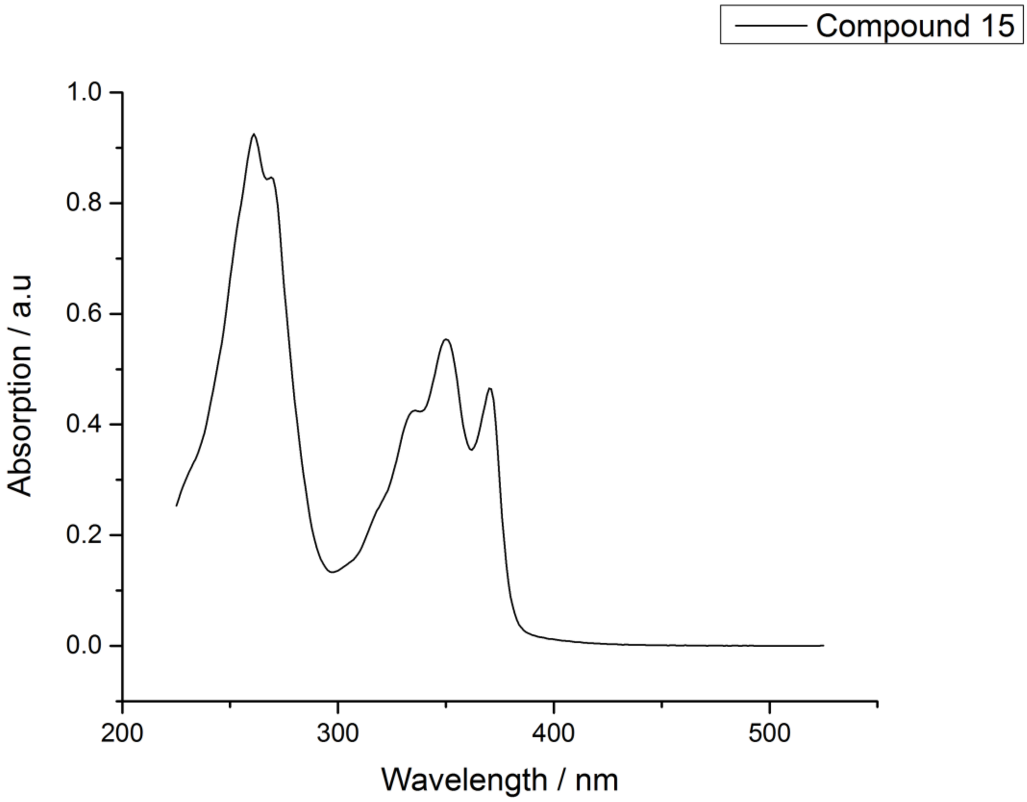

Absorption spectra of Compound 15

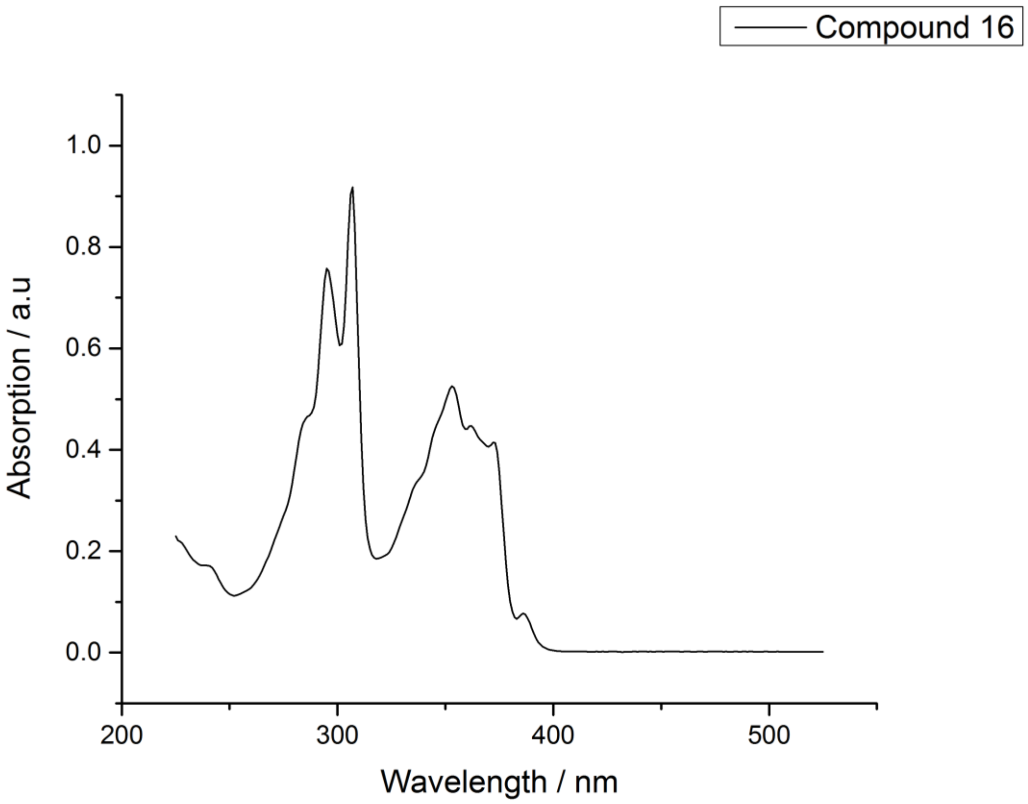

Absorption spectra of Compound 16

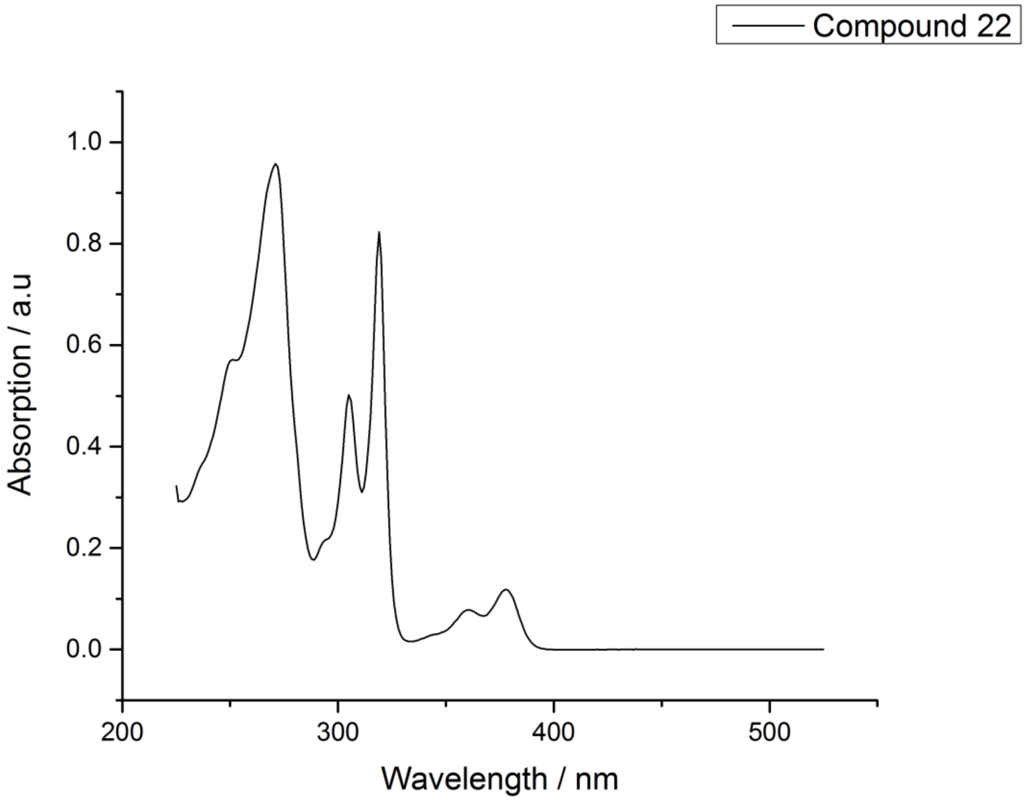

Absorption spectra of Compound 22

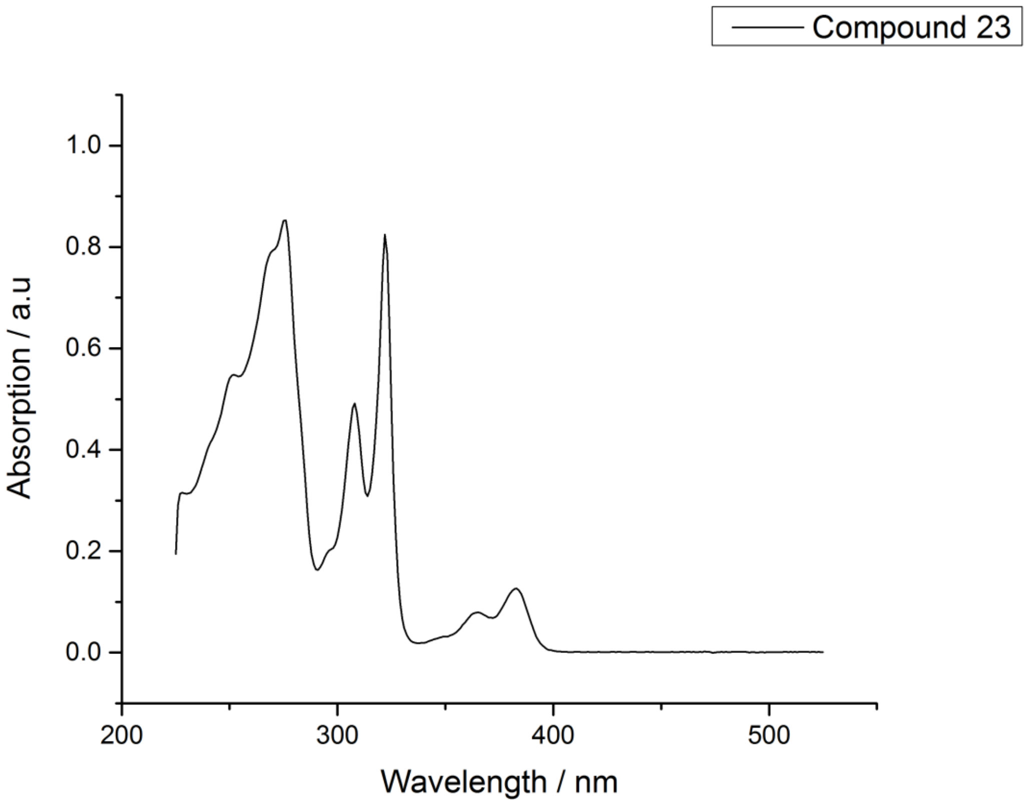

Absorption spectra of Compound 23

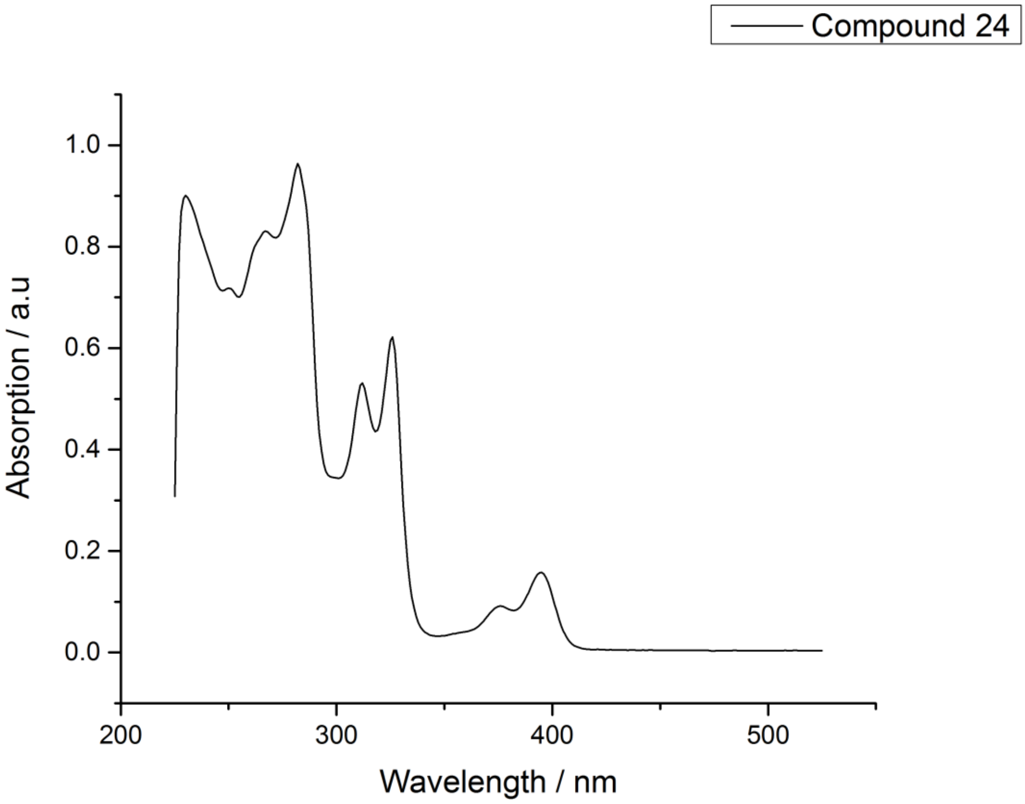

Absorption spectra of Compound 24

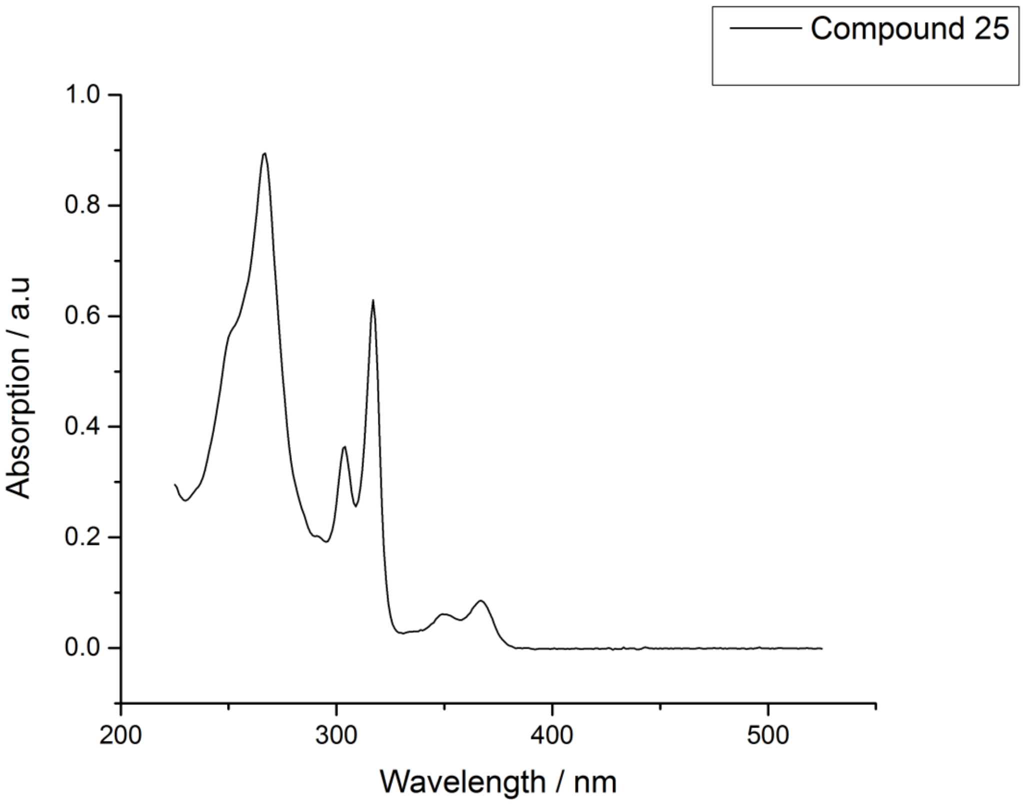

Absorption spectra of Compound 25

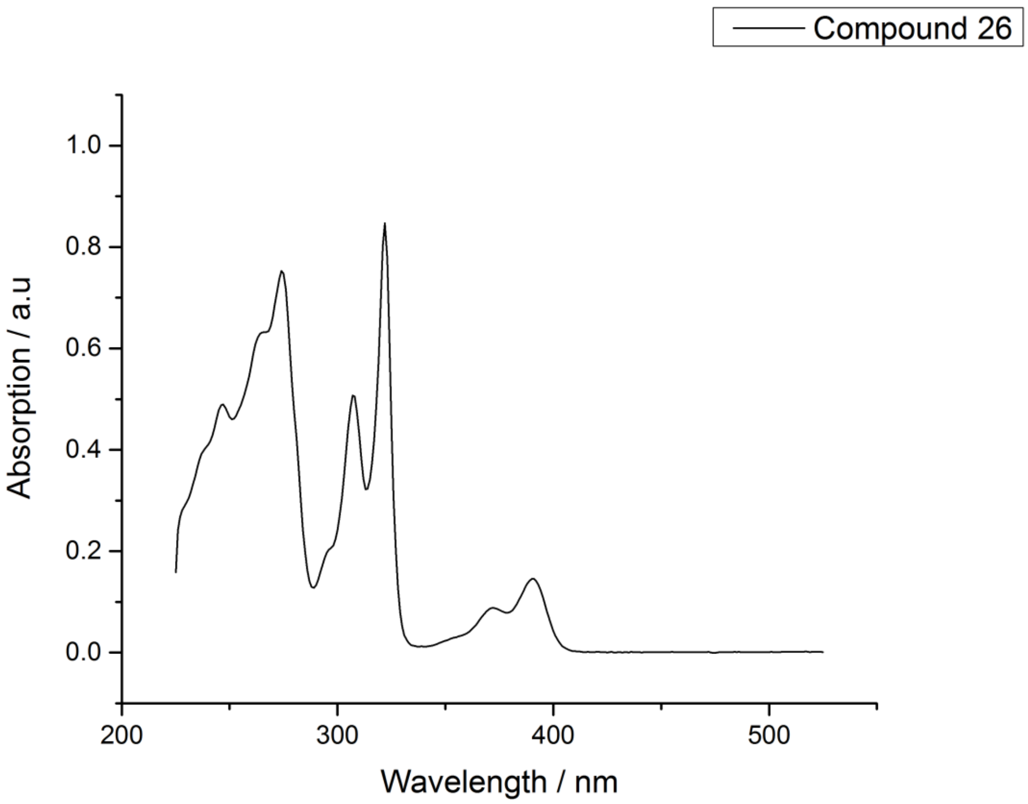

Absorption spectra of Compound 26

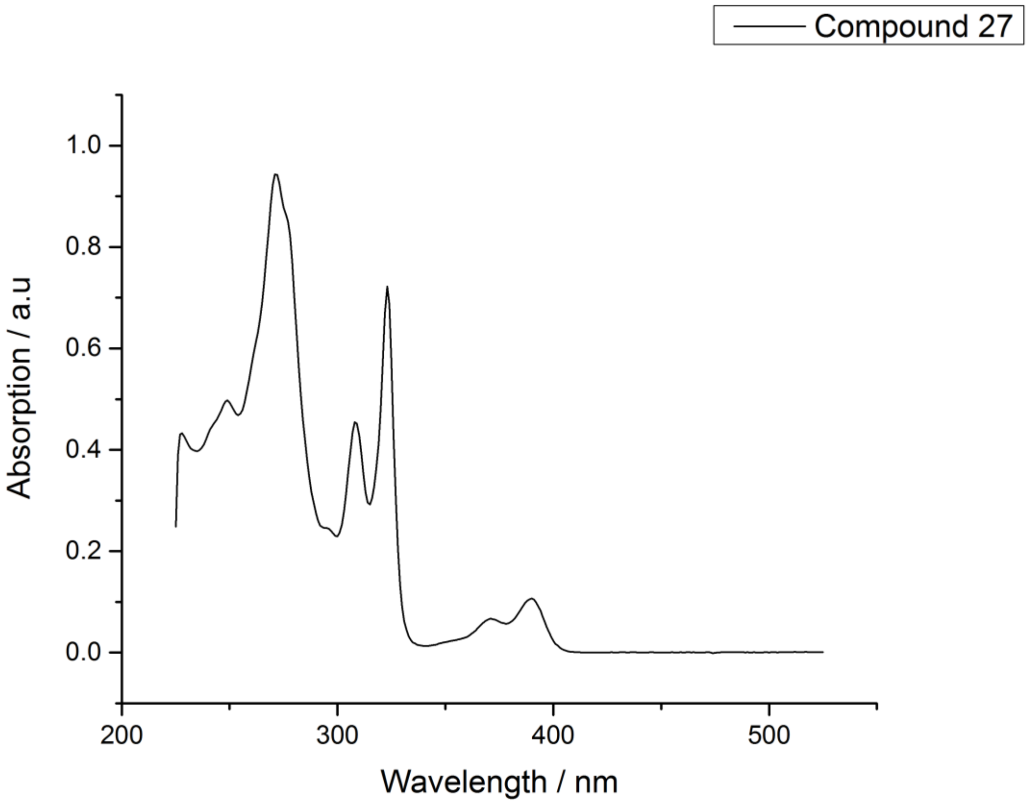

Absorption spectra of Compound 27

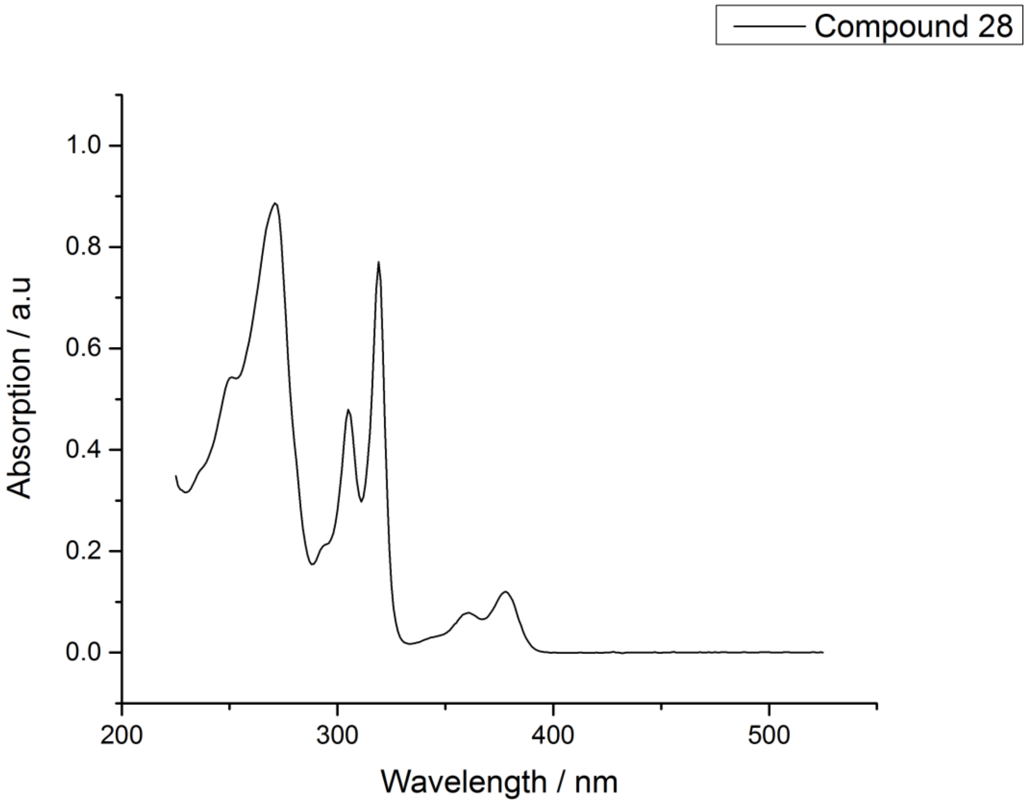

Absorption spectra of Compound 28

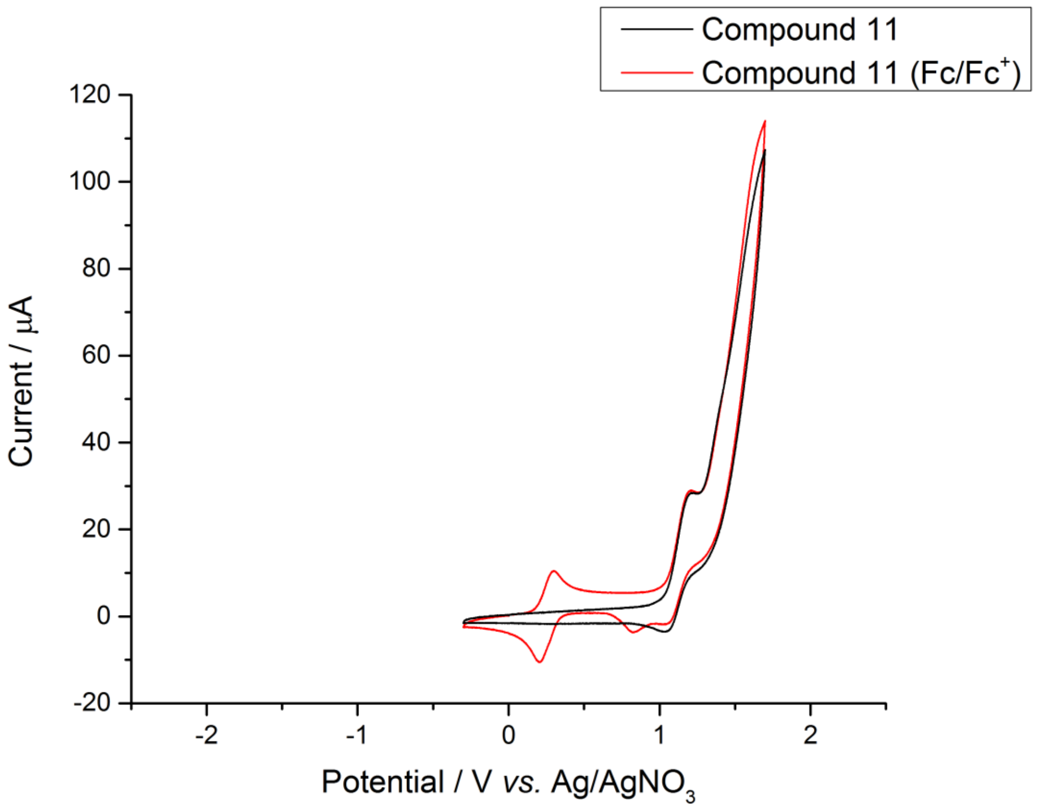

Cyclic Voltammetry of Compound 11 in DCM

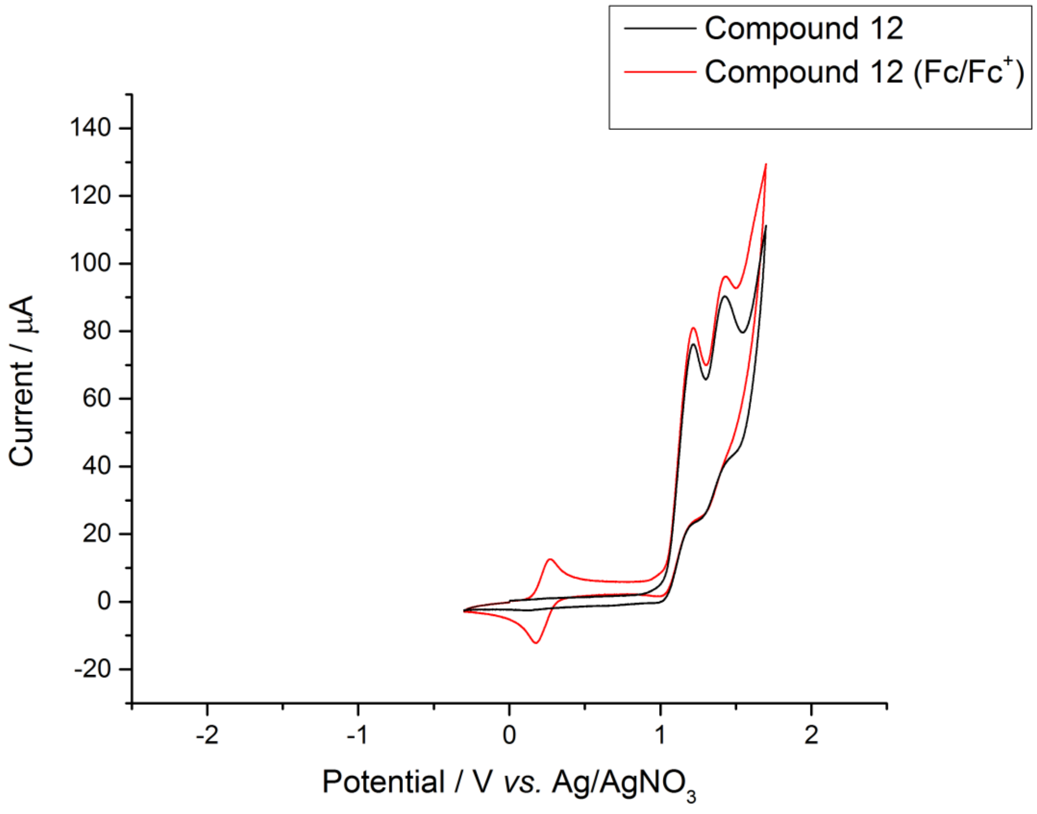

Cyclic Voltammetry of Compound 12 in DCM

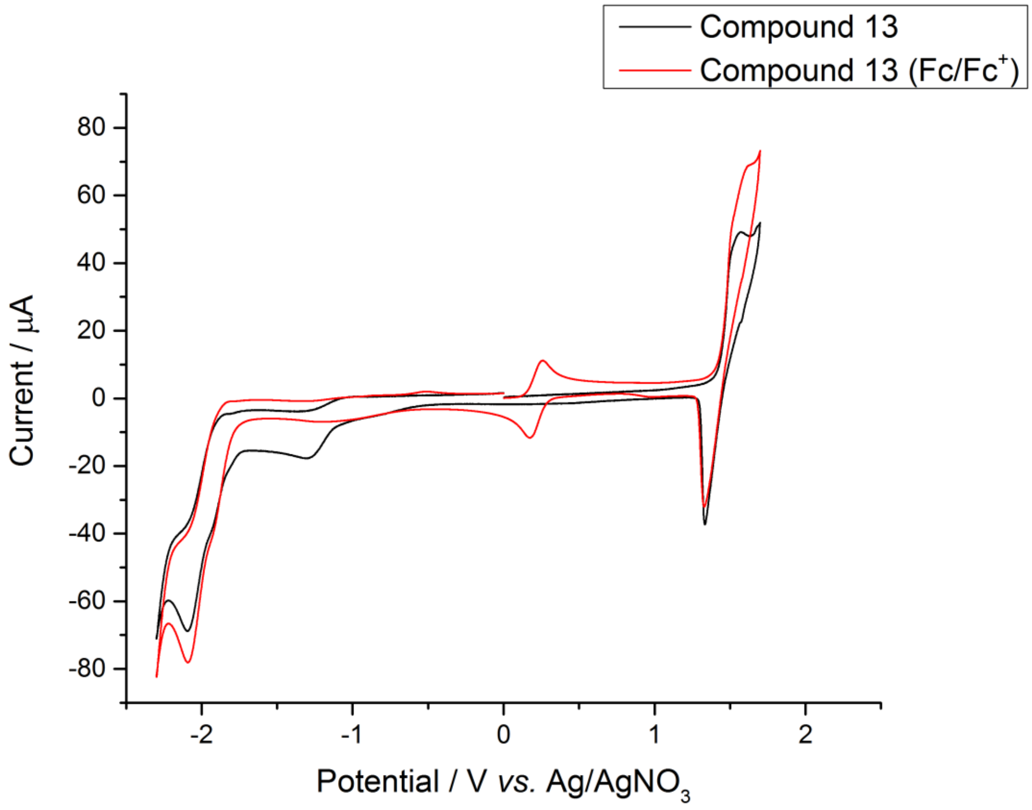

Cyclic Voltammetry of Compound 13 in DCM

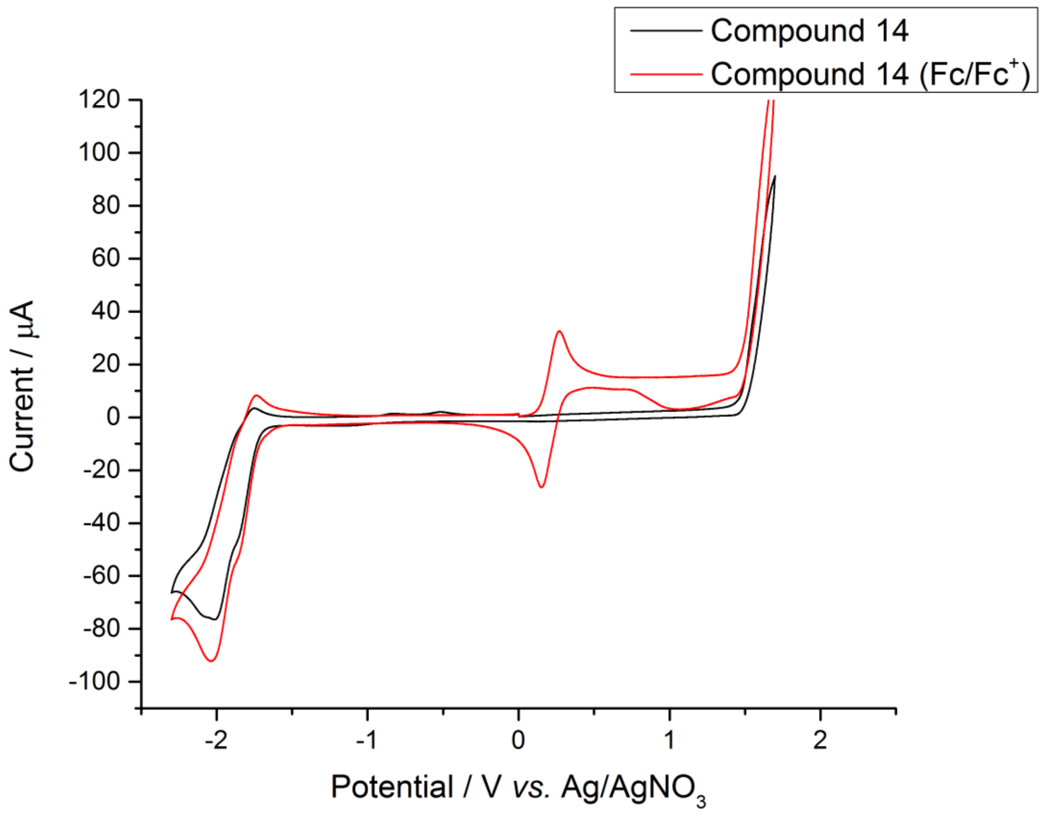

Cyclic Voltammetry of Compound 14 in DCM

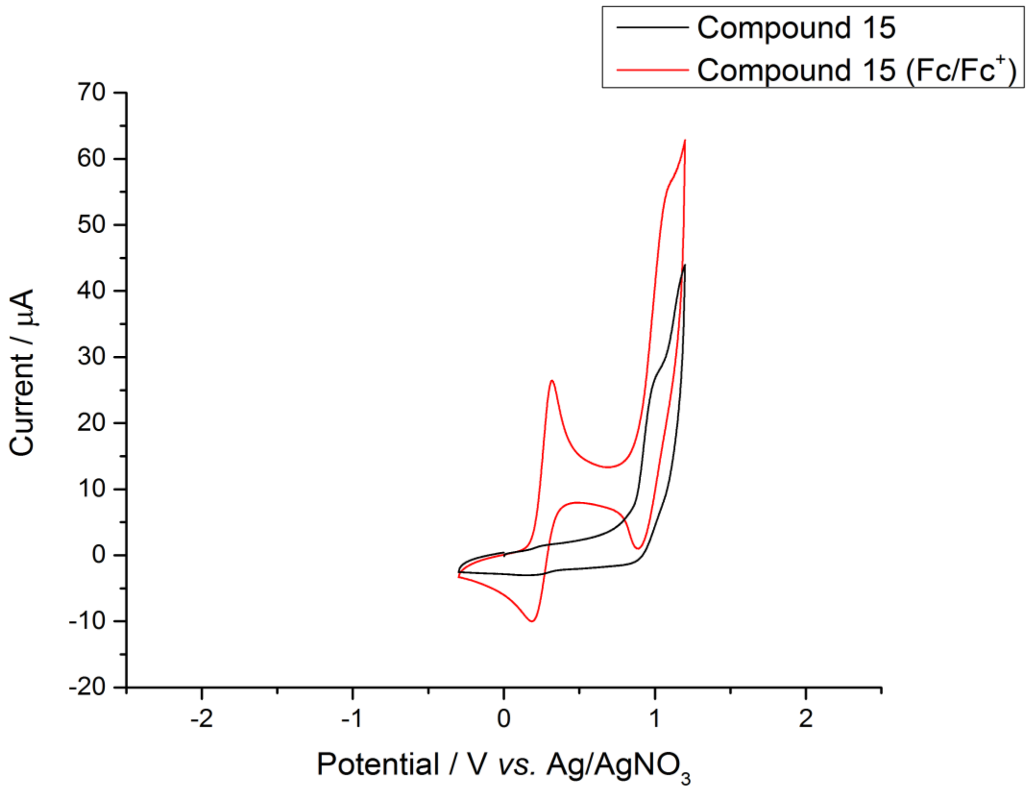

Cyclic Voltammetry of Compound 15 in DCM

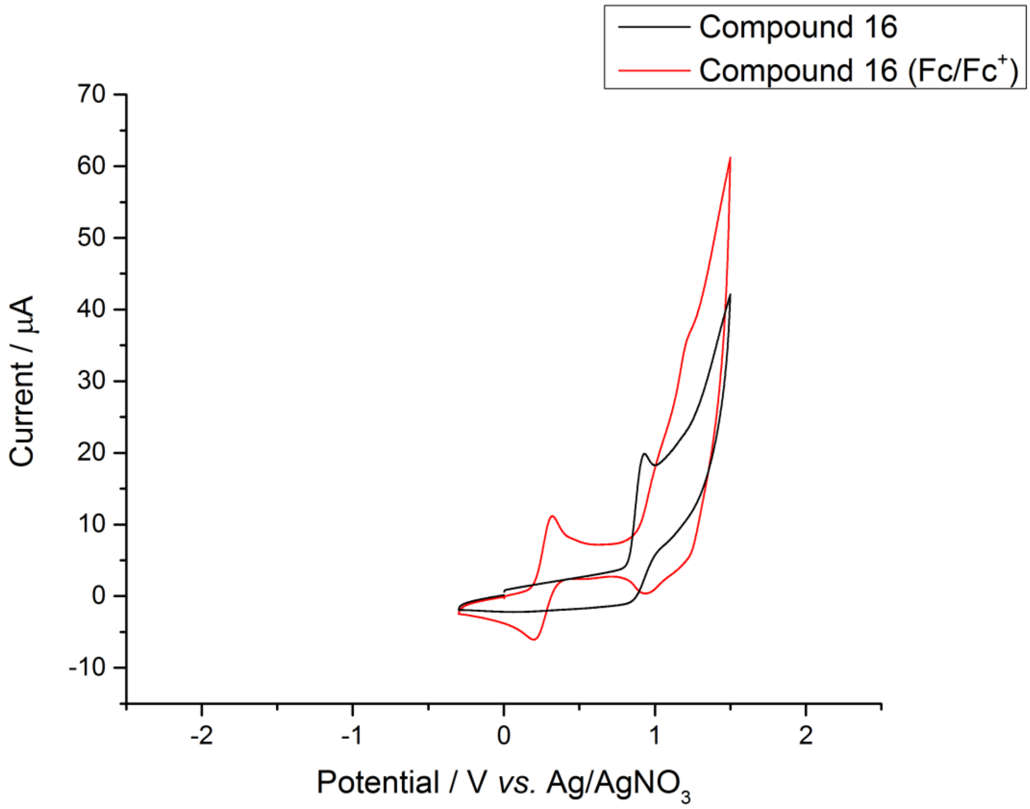

Cyclic Voltammetry of Compound 16 in DCM

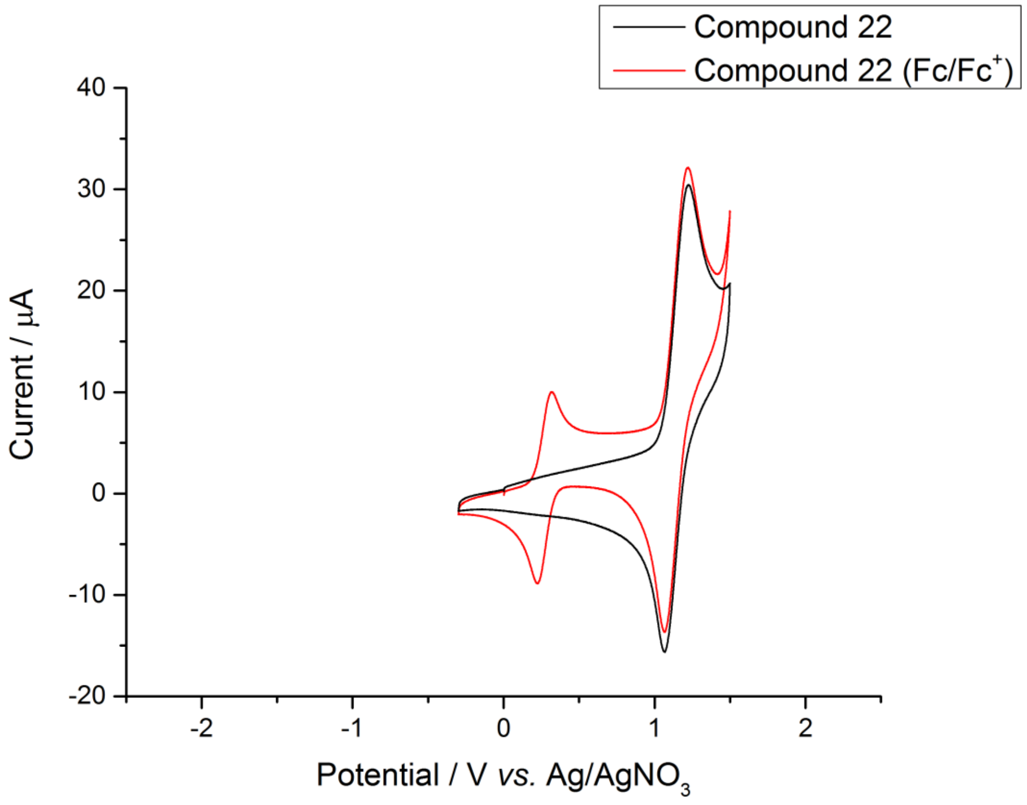

Cyclic Voltammetry of Compound 22 in DCM

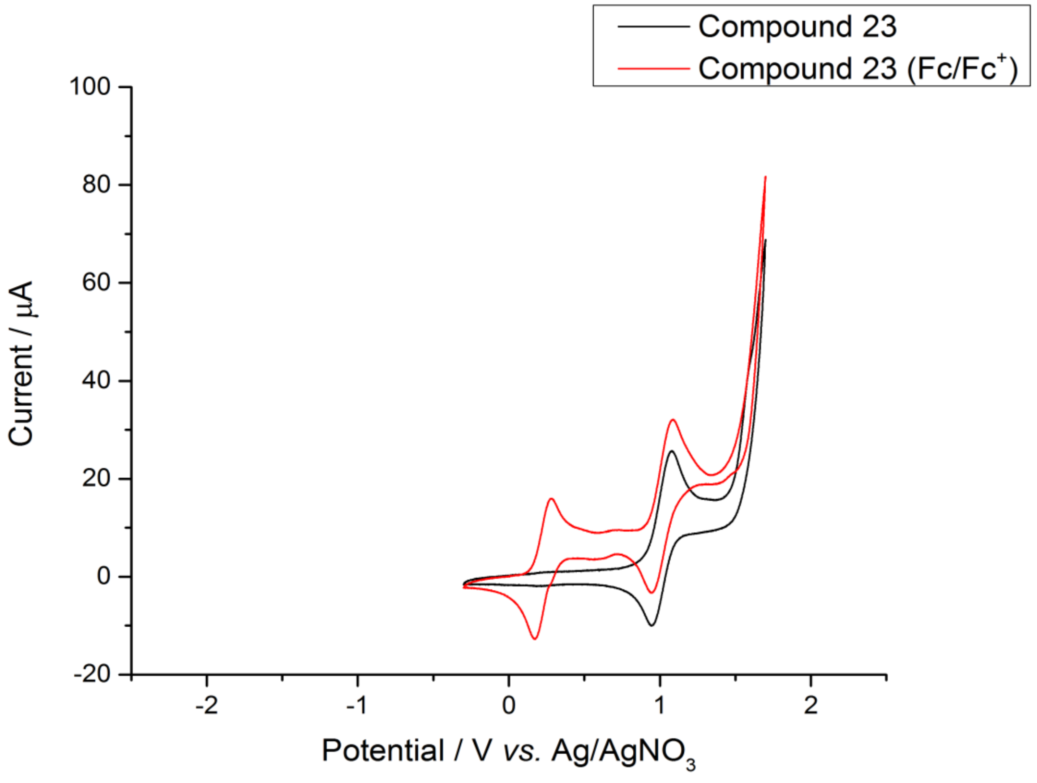

Cyclic Voltammetry of Compound 23 in DCM

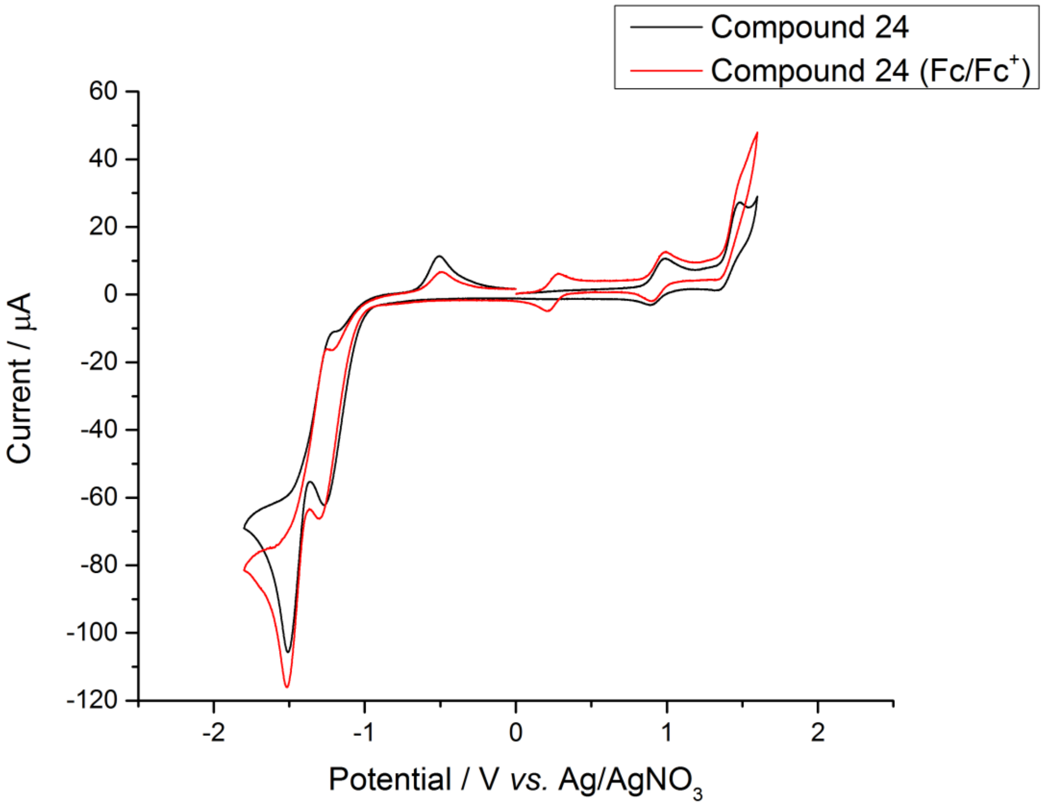

Cyclic Voltammetry of Compound 24 in DCM

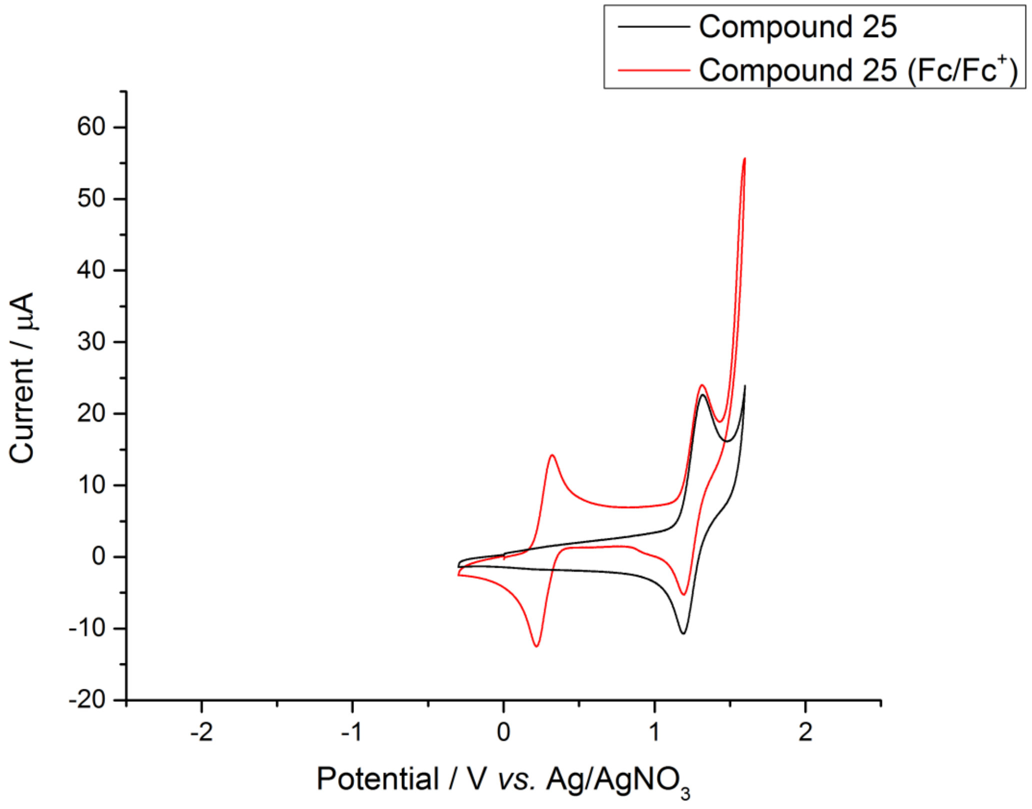

Cyclic Voltammetry of Compound 25 in DCM

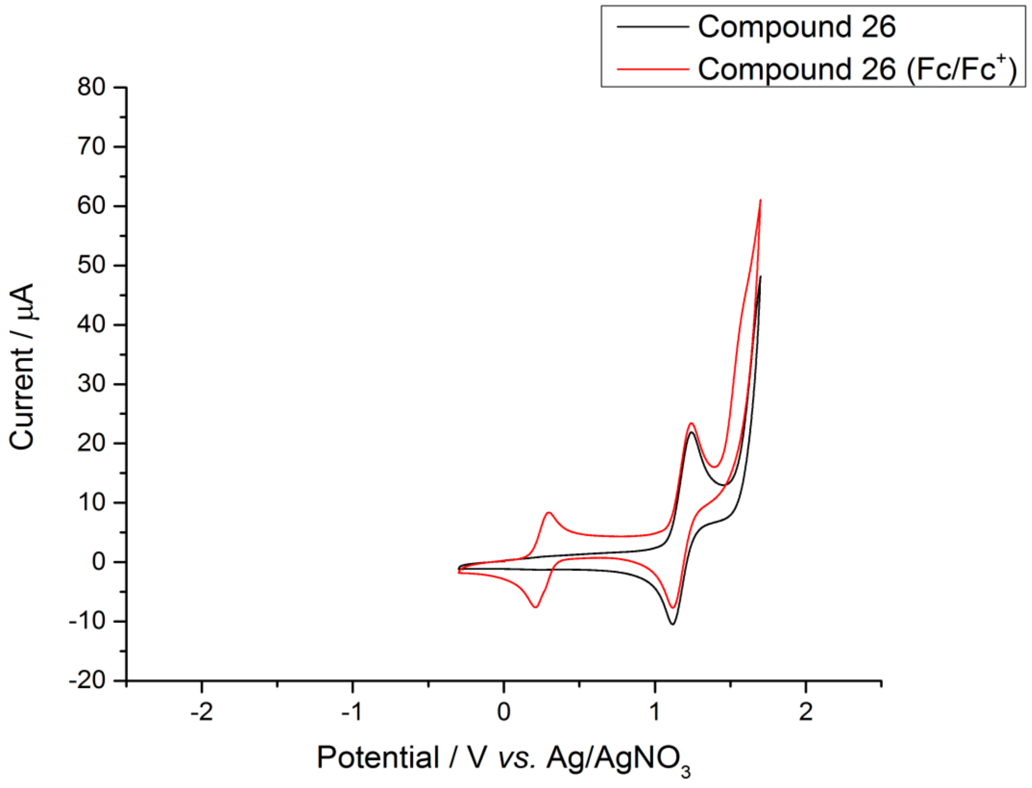

Cyclic Voltammetry of Compound 26 in DCM

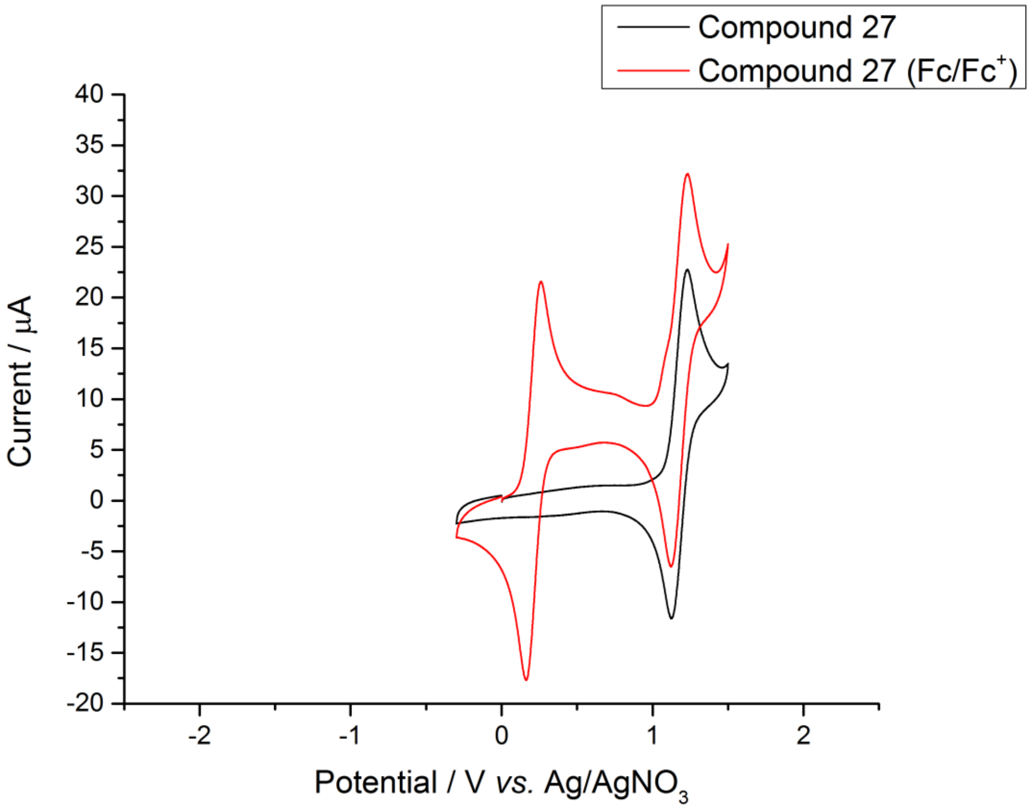

Cyclic Voltammetry of Compound 27 in DCM

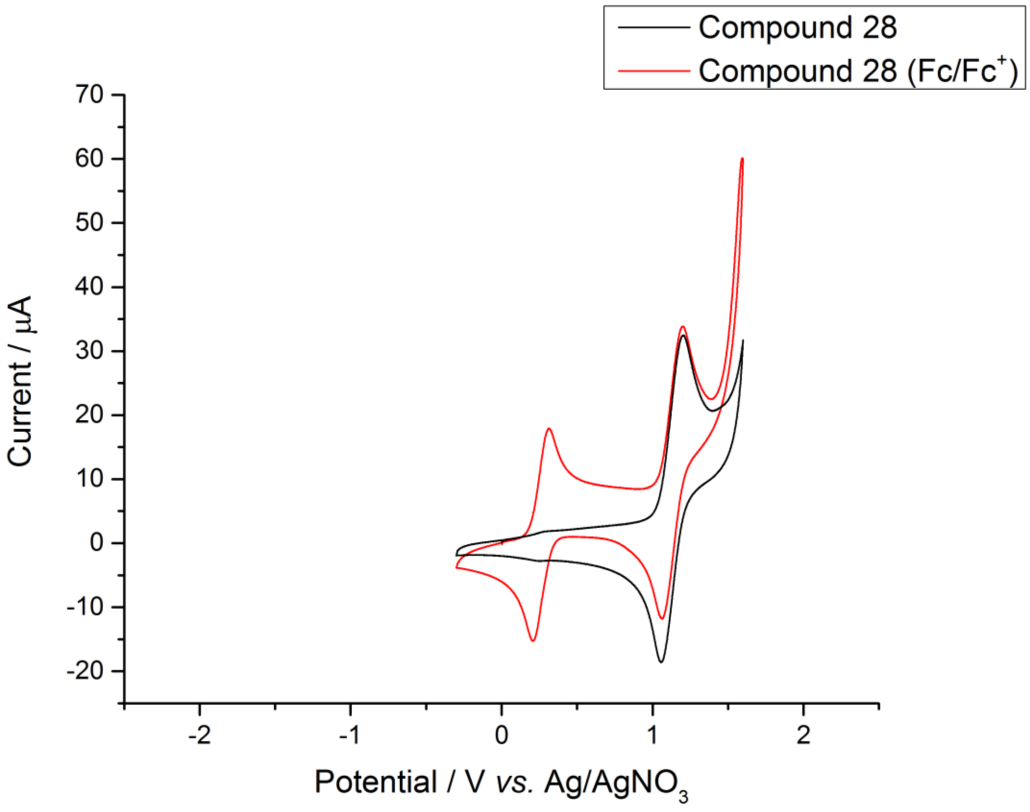

Cyclic Voltammetry of Compound 28 in DCM

X-Ray Structures and CCDC Numbers

Compound 11

CCDC 1415330

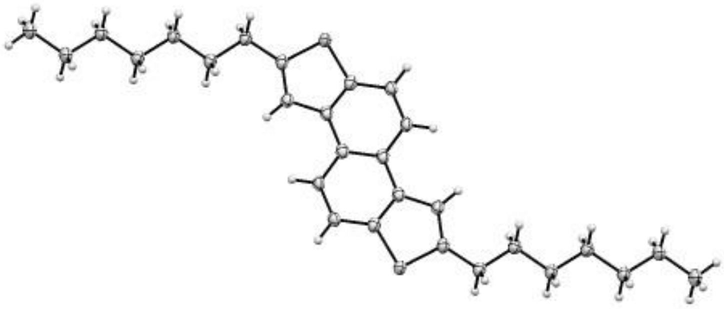

Compound 12

CCDC 1415331

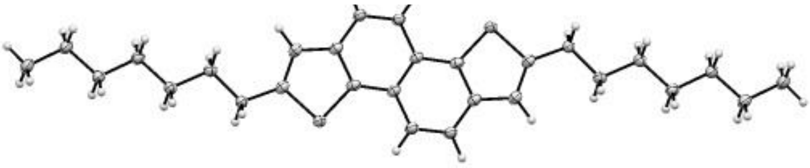

Compound 13

CCDC 1415332

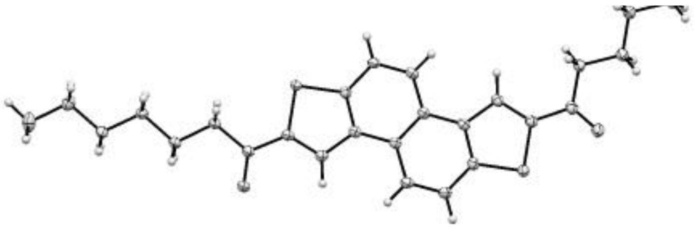

Compound 14

CCDC 1415333

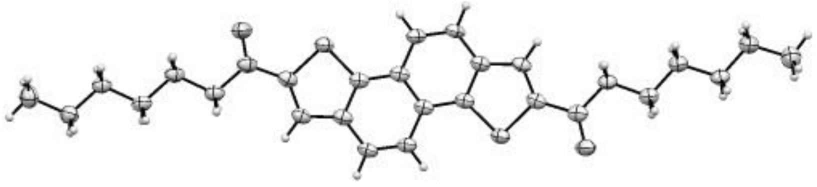

Compound 22

CCDC 1415334

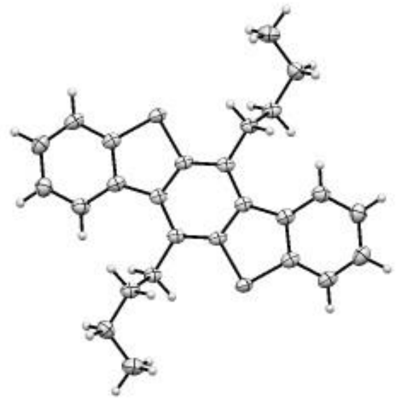

Supplement: Supplementary file 1 [file SC-007-C5SC03823E-s001.pdf]
